# The relationship between processed meat, red meat, and risk of types of cancer: A Mendelian randomization study

**DOI:** 10.3389/fnut.2022.942155

**Published:** 2022-09-20

**Authors:** Kaiwen Wu, Lei Liu, Tao Shu, Aoshuang Li, Demeng Xia, Xiaobin Sun

**Affiliations:** ^1^School of Medicine, Southwest Jiaotong University, Department of Gastroenterology, The Third People's Hospital of Chengdu, The Affiliated Hospital of Southwest Jiaotong University, Chengdu, China; ^2^Medical Research Center, Third People's Hospital of Chengdu, Affiliated Hospital of Southwest Jiaotong University, Chengdu, China; ^3^Luodian Clinical Drug Research Center, Shanghai Baoshan Luodian Hospital, Shanghai University, Shanghai, China

**Keywords:** processed meat, red meat, cancer, Mendelian randomization, genome-wide association studies

## Abstract

**Background:**

Observational studies have suggested processed and red meat may increase the risk of cancer. However, the causal effects and direction between them were still unclear. We conducted two-sample Mendelian randomization (MR) analysis to evaluate the causal effect of processed meat and red meat on the risk of nine common types of cancer, namely, lung, ovarian, endometrial, breast, kidney, gastric, prostate, skin, and oropharyngeal cancer.

**Methods:**

Genome-wide association studies (GWAS) for processed meat and red meat (pork, beef, and mutton) were obtained from the UK Biobank. GWAS of types of cancer in this study were extracted from the genetic consortia and the FinnGen consortium. The inverse variance weighted (IVW) was carried out as the main method for two-sample MR analysis. Sensitivity analyses were used to assess the robustness of the results.

**Results:**

Genetically predicted processed meat intake was causally associated with increased risk of lung cancer (OR [odds ratio] = 1.923, 95% CI = 1.084–3.409, *P* = 0.025). There is no convincing evidence for the associations between genetically determined processed meat, red meat, and the risk of other cancers we studied.

**Conclusion:**

Our results suggested that intake of processed meat may increase the risk of lung cancer. These findings provided no evidence to support that consumption of processed and red meat has a large effect on the risk of other cancers we studied. Further research is needed to clarify the results.

## Background

Cancer is the main cause of morbidity and mortality in the world. According to the research of the International Agency for Research on Cancer (IARC), it was estimated that there were approximately 20 million new cancer cases and nearly 10 million cancer deaths globally in 2020, which had become the main health burden of all countries (1). Previous studies found that dietary factors were associated with cancer risk, especially red and processed meat intake may be an important risk factor for many types of cancer (2).

Red meat (pork, beef, mutton, etc.) is an important source of protein, vitamins, amino acids, minerals, and other nutrients (3). Processed meat refers to improving the taste of meat or increasing the shelf life through several processes such as salting, curing, fermentation, and smoking (4). In recent decades, the consumption of meat has been increasing all over the world. However, it has been reported that high consumption of red and processed meat may increase the risk of cancer (5).

Myoglobin, hemoglobin, and cytochrome which have high levels in red meat were transformed into denatured protein hemes, hemichromes, and hemochromes during cooking and other processing. Oxidative reactions catalyzed by hemoglobin and iron can damage various components of biological systems, such as lipids, proteins, nucleic acids, and other substances. Free radical damage caused by oxidative stress can lead to cancer (6). The IARC working group has shown that consumption of red meat may increase the chance of colorectal cancer, pancreatic cancer, and prostate cancer, while consuming processed meat may increase the possibility of colorectal cancer and gastric cancer (7).

According to previously published systematic reviews and meta-analyses, red and processed meat consumption may lead to an increased risk of cancer (8–11). However, there are still many studies showing that the consumption of processed and red meat may not be linked to higher cancer risk (12–16). Observational studies evaluating the relationship between processed meat, red meat, and the risk of cancers have reported inconsistent results, most likely due to sampling size limitations. Furthermore, observational epidemiological studies are susceptible to confounding and reverse causation (17). Whether there is a causal relationship between the intake of processed or red meat and cancer remains unclear (18). Compared with the observational studies, randomized controlled trials (RCTs) on the consumption of red meat and processed meat could potentially help establish the causal relationship (19). A recent RCT on this topic showed that processed meat intake was not associated with the risk of cancers, and red meat intake could increase the risk of breast cancer (20). However, it is worth noting that volunteers included in the study had more health-conscious behaviors and higher educational levels compared to the general population, which will inevitably bias the results. In addition, the number of cases of cancer at specific sites is relatively small. Therefore, the extrapolation of these results still needs to be cautious.

Mendelian randomization (MR) is a research method used in epidemiology in recent years, mainly through genetic variation to infer the causal relationship between exposure and disease outcomes based on single nucleotide polymorphisms (SNPs) (21). In MR, causal inference of exposure-outcome associations can be improved by using phenotype-related genetic variants as instrumental variables for exposure. Genetic variation follows the rules that alleles segregate randomly from parent to offspring and are determined at conception by genetic variation, so it is not easy to be disturbed by population confounding factors in traditional observational research (22). In addition, the genotypes are not affected by disease phenotypes, so inverse correlation bias can also be avoided (23). Currently, MR has been applied to studies on the causal relationship between dietary habits such as vegetable intake and cancer (24, 25). For example, Chen Jin et al. conducted a two-sample MR analysis to explore the relationship between the causal relationship between dried fruit intake and the risk of cancers. Studies have shown that the consumption of dried fruit may have a protective effect against cancer. It is suggested that health education and reasonable adjustment of dietary ratios may contribute to the primary prevention of cancer (26). There is also a high-quality MR study on the association between processed meat and the risk of cancer. Qi Feng et al. performed both observational analyses with UK Biobank and genetic analysis with MR to explore the effect of processed meat intake on the risk of colorectal cancer. The results showed that heavy consumption of processed meat independently increases the risk of colorectal cancer, and processed meat intake reduction may be an effective strategy for preventing colorectal cancer (27).

In our study, we performed a two-sample MR analysis to assess the potential causal relationship between processed red meat intake and the risk of cancers from the GWASs and UK Biobank that were publicly available.

## Methods

We used data from published studies or GWAS summaries that were openly available. Since no primary data were used in this study, ethical approval was not required. All the studies included were permitted by their academic ethics review committees, and each participant signed written informed consent.

### Exposure and outcome measures

**Figure 1:**
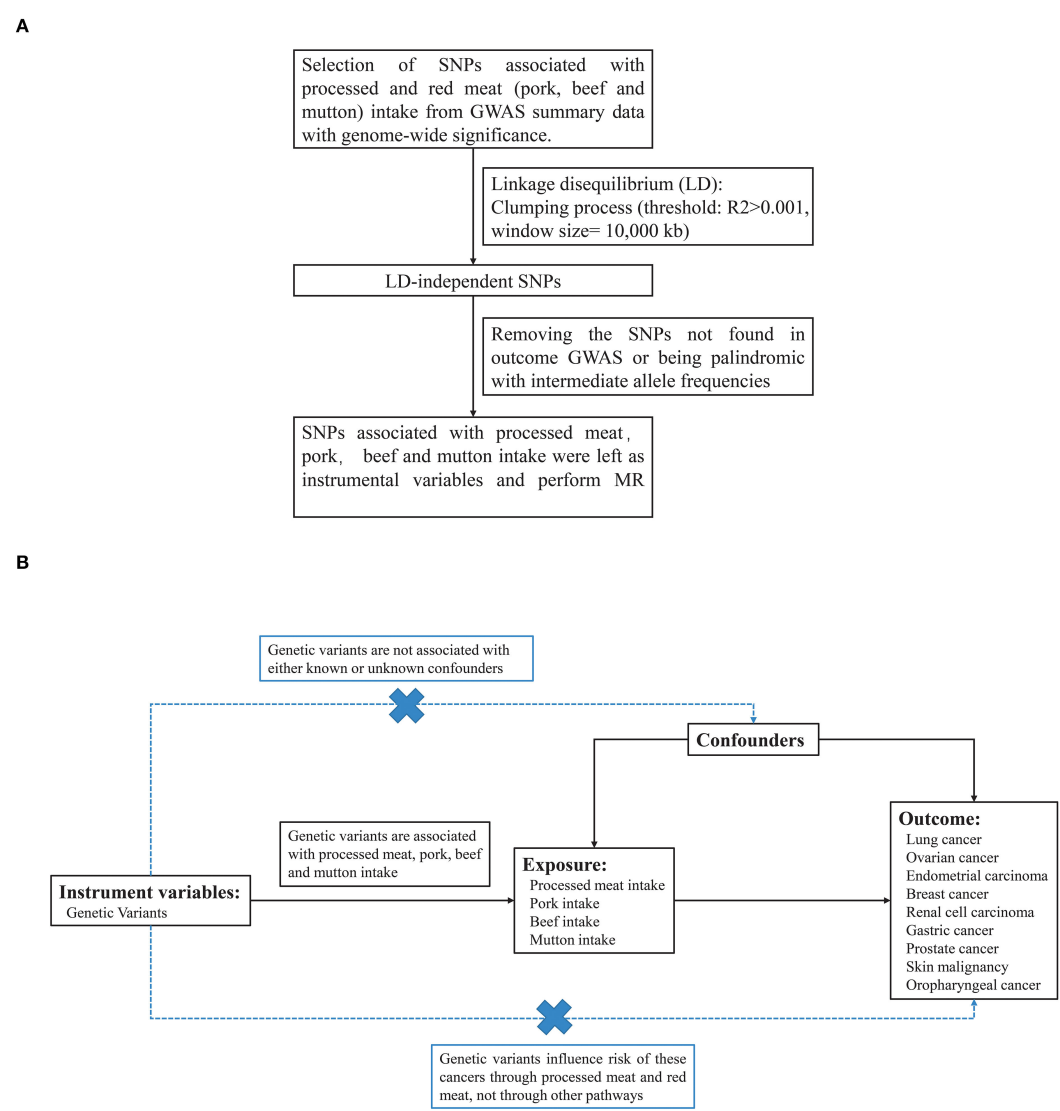
**(A)** The design of MR analysis in our study. **(B)** The flow chart about the process of screening for SNPs associated with exposure.

Dietary exposures (processed meat, pork, beef, and mutton) were obtained from the UK BioBank cohort with 461,981, 460,162, 461,053, and 460,006 individuals of European ancestry, respectively. To minimize the effects of linkage disequilibrium (LD), single nucleotide polymorphisms (SNP) that passed the generally accepted genome-wide significance threshold (*P* < 5 × 10^−8^, R^2^>0.001 within a 10,000 kb window) for exposures were chosen as instrumental variables (Figure 1A). The detailed information on these independent, genome-wide SNPs was shown in Supplementary material 1. F statistics and proportion of variance explained (PVE) were computed to test whether a weak instrument bias was present.

We use large-scale GWAS data for nine types of cancer as outcome factors. Breast cancer data were obtained from GWAS meta-analysis from Breast Cancer Association Consortium (BCAC) studies involving people of European ancestry (46,785 cases and 42,892 controls). Data for prostate cancer was derived from a genome-wide association analysis of 79,148 patients and 61,106 controls of European ancestry by the Prostate Cancer Association Group to Investigate Cancer-Associated Alterations (PRACTICAL) in the Genome Consortium. For lung cancer, we used data from the International Lung Cancer Consortium, consisting of 11,348 cases and 15,861 controls of European descent. GWAS data for ovarian cancer were acquired from the Ovarian Cancer Alliance Consortium, which included 25,509 patients of European ancestry. Genome-wide association analysis results for gastric cancer, renal cell carcinoma, and skin malignancy were all derived from European ancestry data in FinnGen Biobank analysis (Table 1). Our study only utilized the results of published GWAS and did not involve individual-level data. All exposure and outcome summary data were downloaded from the IEU OpenGWAS project (https://gwas.mrcieu.ac.uk/).

**Table 1:** Number of cancer cases and controls in the Mendelian randomization study on the association of processed meat and red meat intake with risk of site-specific cancer.

**Outcome**	**Data source**	**Cases (n)**	**Controls (n)**	**Population**
Lung cancer	ILCCO	11,348	15,861	European
Ovarian cancer	OCAC	25,509	40,941	European
Endometrial carcinoma	Consortium (Tracy et al.)	12,906	108,979	European
Breast cancer	BCAC	46,785	42,892	European
Kidney cancer	The FinnGen consortium	971	174,006	European
Gastric cancer	The FinnGen consortium	633	174,006	European
Prostate cancer	PRACTICAL	79,148	61,106	European
Skin malignancy	The FinnGen consortium	10,384	208,408	European
Oropharyngeal cancer	Consortium (Corina et al.)	2,497	2,928	European

ILCCO, International Lung Cancer Consortium; OCAC, Ovarian Cancer Alliance Consortium; BCAC, Breast Cancer Association Consortium; PRACTICAL, Prostate Cancer Association Group to Investigate Cancer-Associated Alterations in the Genome Consortium.

### Mendelian randomization

The MR analysis was carried out using the TwoSampleMR R package and the **“**MR-PRESSO**”** R package (version 0.4.13, http://github.com/MRCIEU/TwoSampleMR). All of our studies were based on a two-sample MR framework, which obtained SNP-exposure (processed meat intake, pork intake, beef intake, and mutton intake) associations and SNP-outcome (lung cancer, ovarian cancer, endometrial carcinoma, breast cancer, renal cell carcinoma, gastric cancer, prostate cancer, skin malignancy, and oropharyngeal cancer) associations from different cohorts to estimate the causal effects of exposure on the outcome. In total, six MR methods were used to estimate the effect of genetically predicted exposure on cancers namely the main analysis method inverse variance weighted (IVW), and other five additional analysis methods, Mendelian randomization pleiotropy residual sum and outlier (MR-PRESSO), maximum likelihood, MR Egger, weighted median, and penalized weighted media. For the IVW method, we used a random-effects model when the results were heterogeneous, and a fixed-effects model was used when there was no heterogeneity. The maximum likelihood method was performed by estimating the causal effects of the effect of SNPs on exposure and outcome by direct maximization of the likelihood (28). The MR-PRESSO method was used to detect outlier variables in IVW analysis by comparing the actual distance of the genetic variants to the expected distance from the regression, assuming the absence of horizontal pleiotropy and evaluating the causal estimates after removing outliers (29). The MR–Egger approach utilizes InSIDE to perform a weighted linear regression of exposure results but is susceptible to IVs (30). In addition, the weighted median method can significantly improve the detection ability of causal effects and reduce type I errors (31).

### Pleiotropy and sensitivity analyses

To test for heterogeneity, MR Egger and IVW were carried out. The SNP-exposure association and the SNP-outcome association estimates were involved in MR Egger. Using the slope of the weighted regression line, we estimated the causal effect of exposure on the outcome, independent of horizontal pleiotropy. An estimate of the causal effect of exposure on outcome was provided by the slope of the weighted regression line and was not affected by horizontal pleiotropy. In the MR-Egger test, the intercept assesses the mean pleiotropy of genetic variation, with values greater or less than zero indicating possible bias in IVW estimates. The sensitivity of the results was analyzed using the leave-one-out method. The SNPs were sequentially removed one at a time to examine whether the individual SNPs with potentially large horizontal pleiotropic effects could affect MR estimate.

## Result

### Selection of instrumental variables

We used the summary GWAS data from UK Biobank for each processed meat, pork, beef, and mutton as exposures and risk for 9 types of cancer as the outcome in different studies (Figure 1B). Two-sample MR analysis was performed to explore the causal relationship between processed/red meat and cancer. Supplementary material 1 showed the SNP information of four exposures (intake of processed meat, pork, beef and mutton), consisting of the name, chromosome location, genes, function, effect allele (EA), other alleles, and effect allele frequency (EAF). We calculated the F statistic for instrumental variable selection and F statistics were >10, which indicated that we have effectively avoided the bias caused by weak instrumental variables (Supplementary material 1) (32).

### The causal effect of processed red meat and cancer

**Figure 2:**
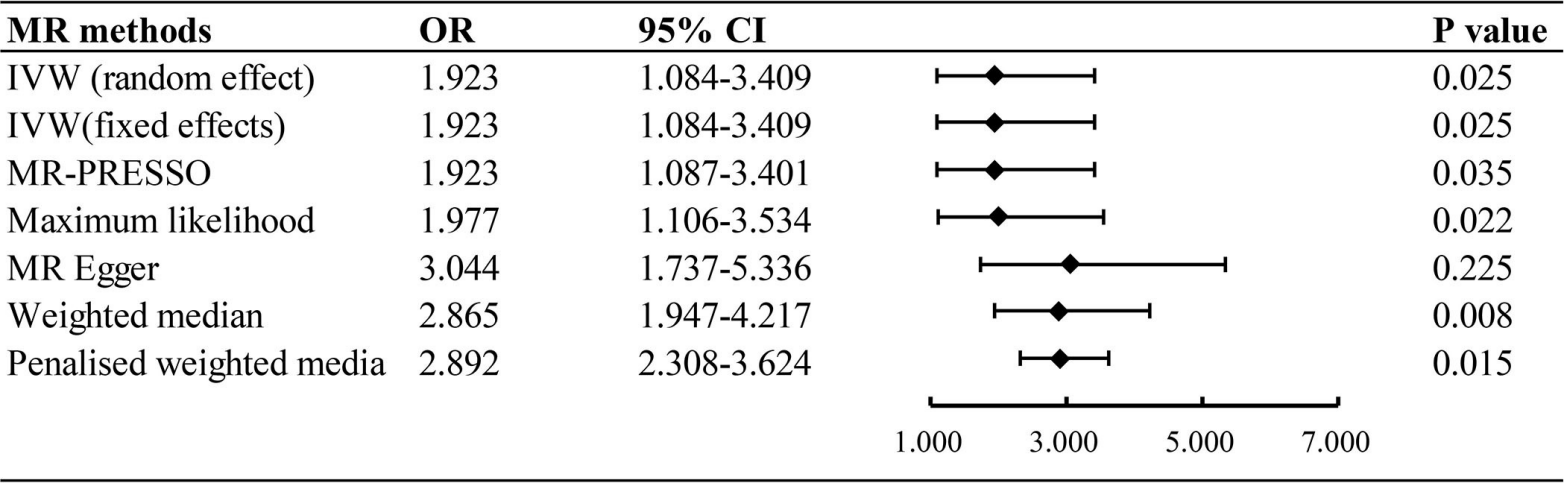
Associations of genetically predicted processed meat intake with risk of lung cancer. OR, odds ratio; CI, confidence interval.

The inverse variance weighted (random effect and fixed effect), maximum likelihood, MR Egger, weighted median, and penalized weighted media were used to estimate causal associations between genetically predicted processed/red meat and the risk of 9 types of cancer. It showed that processed meat was associated with an increased risk odds of lung cancer (IVW: OR = 1.923, 95% CI = 1.084–3.409, *P* = 0.025) (Figure 2). A higher processed meat intake was not associated with the risk of ovarian cancer, endometrial carcinoma, breast cancer, renal cell carcinoma, gastric cancer, prostate cancer, skin malignancy, and oropharyngeal cancer. Consumption of red meat did not significantly increase the risk of cancer (Supplementary material 2).

### Sensitivity analyses

The horizontal pleiotropy between SNPs and outcomes was assessed by MR-Egger regression, which showed no evidence of horizontal pleiotropy (Supplementary material 4). The funnel plots showed a symmetric pattern of effect size variation around the point estimates, indicating no apparent horizontal pleiotropy (Supplementary material 3). The results of the leave-one-out sensitivity analyses demonstrated that no potentially influential SNPs drive the causal link and the stability of our conclusion (Supplementary material 3).

## Discussion

In this study, a two-sample MR analysis was performed using the instrumental variables of large-scale GWAS to assess the causal relationship between processed/red meat and cancers using genetic data from populations of European descent. In our MR analysis, genetic predisposition to processed meat consumption was associated with a higher risk of lung cancer, with an OR of 1.923 [95% CI, 1.084–3.409; *P* = 0.025]. Results from a two-sample MR analysis suggested that processed meat consumption was not associated with the risk of ovarian cancer, endometrial carcinoma, breast cancer, renal cell carcinoma, gastric cancer, prostate cancer, skin malignancy, and oropharyngeal cancer. In this study, no strong evidence was found to support associations between red meat intake and the risk of types of cancer.

There was growing evidence that high levels of red meat intake, and processed meat consumption were linked to an increased risk of types of cancer (33, 34). A large observational study involving more than 470,000 people with a follow-up of 11.4 years showed a reduced risk of colorectal cancer and breast cancer in people who consumed less red meat (35). World Cancer Research Fund/American Institute for Cancer Research (WCRF/AICR) also advised that limiting red meat intake and avoiding consumption of processed meat may modestly reduce the risk of cancer (36). Giuseppe et al. (37) compiled 24 meta-analyses of the association of red meat and 39 processed meat consumption with the risk of cancer published between 2005 and 2015. The results indicated an increased risk of cancer in subjects consuming large amounts of red and processed meat. It is possible that high-temperature cooked meats can produce N-nitroso compounds (NOCs). Heterocyclic amines formed in meat smoking can become carcinogens after metabolic activation (38). Heterocyclic aromatic amines (HAAs) can be derived from high-temperature cooked red meat (39). The rich heme in red meat can catalyze the production of NOC and lipid peroxidation products (LPO). These carcinogens combine with DNA to form DNA adducts, which interfere with DNA replication and repair, and cause gene mutations during cell division, inducing the occurrence of cancer (40). The 2-amino-1-methyl-6-phenylimidazo[4,5-b] pyridine (PhIP), a heterocyclic amine widely present in processed and red meat, induced cancer through cytochrome P450-mediated DNA damage and metabolic activation of mutagens (41, 42).

In MR analysis, it failed to detect significant associations of genetic predisposition to processed/ red meat with most of the cancers studied (*P* > 0.05). This was consistent with the conclusions of some guidelines and observational studies (43–47). A meta-analysis of 6 million participants in 56 cohort studies also found evidence of low quality that with the reduction of unprocessed red meat, the total cancer mortality would decrease. An intake reduction of three servings of processed meat per week was not associated with a lower incidence of cancer of the mouth, stomach, small bowel, liver, pancreas, endometrial, or prostate. Although studies have shown that reducing the intake of processed meat can reduce the risk of esophageal, colorectal, and breast cancer, the certainty of the evidence was very low due to the observation design and inaccuracy. In addition, there was low-certainty evidence that reducing the intake of red meat was associated with a very small overall reduction in cancer mortality (48). According to the Nutritional Recommendations (NutriRECS) Consortium's report, there was a low and very low quality that meat consumption could lead to potential adverse health outcomes. The probability of esophageal cancer, colorectal cancer, and breast cancer caused by high consumption of processed meat was not significantly different from that of low consumption (49). A definitive causal relationship requires more in-depth mechanism studies and RCT studies in the future.

The Mendelian randomization can avoid bias from unmeasured confounding and avoid bias from reverse causation and offer some protection against biases that can be conceptualized as reverse causation (50, 51). Our study tried to avoid some problems of confounding factors and reverse causality, but there were still some limitations. First, this is a pooled analysis of individual studies, due to the lack of original data, we could not conduct a patient-level analysis. Second, like all MR studies, horizontal pleiotropy, as a common issue, is difficult to avoid. Although some MR methods such as the leave-one-out method and MR-Egger were used to test, which indicated our results were not affected by pleiotropy, the possibility of bias could not be ruled out. Third, our results suggested a potential causal relationship between processed/red meat and types of cancer, the analysis presented here does not provide evidence for specific mechanisms of tumorigenesis. Fourth, wide CIs was observed under the MR-Egger method in MR analyses, which may hint at low potency. However, the MR-Egger method is often underpowered in studies and other Mendelian analyses were qualitatively consistent with the primary analysis of the inverse-variance weighted method. The last but not least, although using a single European population to investigate the causal relationship between processed/red meat and cancer can minimize population stratification bias, it might not be generalizable to other populations.

## Conclusion

In conclusion, there is an obvious positive causal relationship between the genetically predicted processed meat and lung cancer. We did not find a causal relationship between processed, red meat, and other studied cancers. Observational studies had previously suggested an association between processed/red meat and cancer. Although traditional epidemiological studies can help us preliminarily understand the correlation between meat consumption and cancer, traditional epidemiological studies are influenced by confounding factors, such as social and demographic components. In addition, unrecognized bias may lead to inaccurate results. Further MR studies may be needed to assess the relationship between meat consumption and important risk factors for cancer.

## Data availability statement

All summary statistics based on association data are available free of charge. The data of processed meat intake (ID: ukb-b-6324), pork intake (ID: ukb-b-5640), beef intake (ID: ukb-b-2862), mutton intake (ID: ukb-b-14179), lung cancer (ID: ieu-a-966), prostate cancer (ID: ieu-b-85), oral cavity and pharyngeal cancer (ID: ieu-b-89), breast cancer (ID: ieu-a-1130), ovarian cancer (ID: ieu-a-1120), endometrial cancer (ID: ebi-a-GCST006464), skin malignancy (ID: finn-b-C3_SKIN), kidney cancer (ID: finn-b-C3_KIDNEY_NOTRENALPELVIS) and gastric cancer (ID:finn-b-C3_STOMACH_EXALLC) can be obtained from https://gwas.mrcieu.ac.uk/.

## Author contributions

XS and DX contributed to the study's conception and design. Material preparation, data collection, and analysis were performed by KW, TS, and AL. The first draft of the manuscript was written by KW and LL. All authors commented on previous versions of the manuscript and read and approved the final manuscript.

## Funding

This work was supported by the Foundation of Science and Technology, Department of Sichuan Province (2020YJ0485).

## Conflict of interest

The authors declare that the research was conducted in the absence of any commercial or financial relationships that could be construed as a potential conflict of interest.

## Publisher's note

All claims expressed in this article are solely those of the authors and do not necessarily represent those of their affiliated organizations, or those of the publisher, the editors and the reviewers. Any product that may be evaluated in this article, or claim that may be made by its manufacturer, is not guaranteed or endorsed by the publisher.

## Supplementary material

Supplementary material 1 Instrumental SNPs from processed meat and red meat (pork, beef, and mutton) GWASs.

Supplementary material 2 MR estimates from each method of the causal effect of exposure on cancers.

Supplementary material 3 Leave-one-out analysis, funnel plot, and MR effect size for processed meat, pork, beef, and mutton intake on cancer.

Supplementary material 4 Heterogeneity and level pleiotropy test in the Mendelian randomization analyses.
