# Supplementary material for: The relationship between processed meat, red meat, and risk of types of cancer: A Mendelian randomization study
Source: Front Nutr. 2022 Sep 20;9:942155. doi: 10.3389/fnut.2022.942155 (PMC9530935; doi:10.3389/fnut.2022.942155)
Supplement: Supplementary material 1 — Instrumental SNPs from processed meat and red meat (pork, beef, and mutton) GWASs. [file Data_Sheet_1.ZIP › Supplementary material 3.pdf]

Figure S1.Leave-one-out analysis, funnel plot and MR effect size for processed meat, pork, beef and mutton intake on lung cancer.

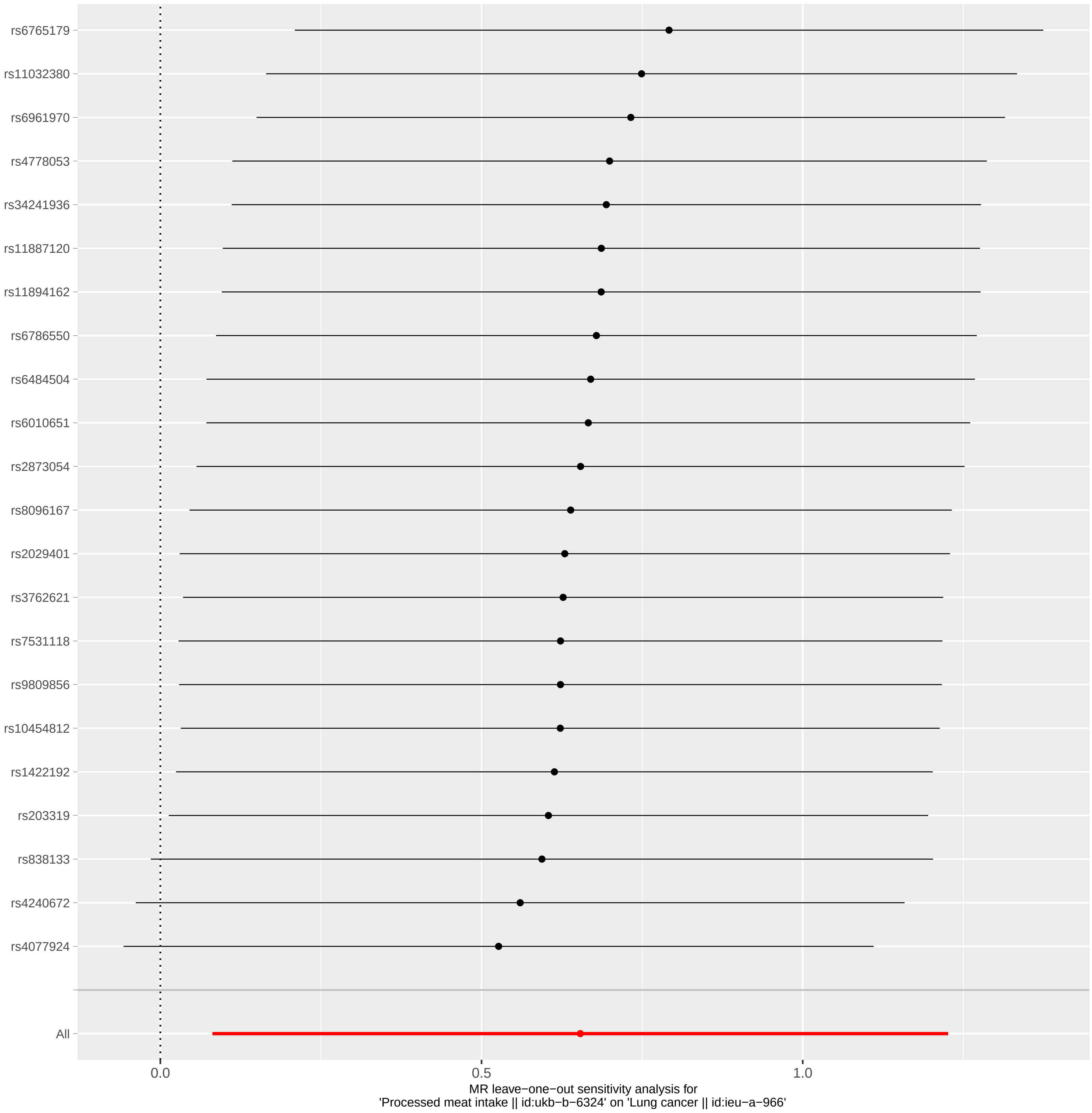

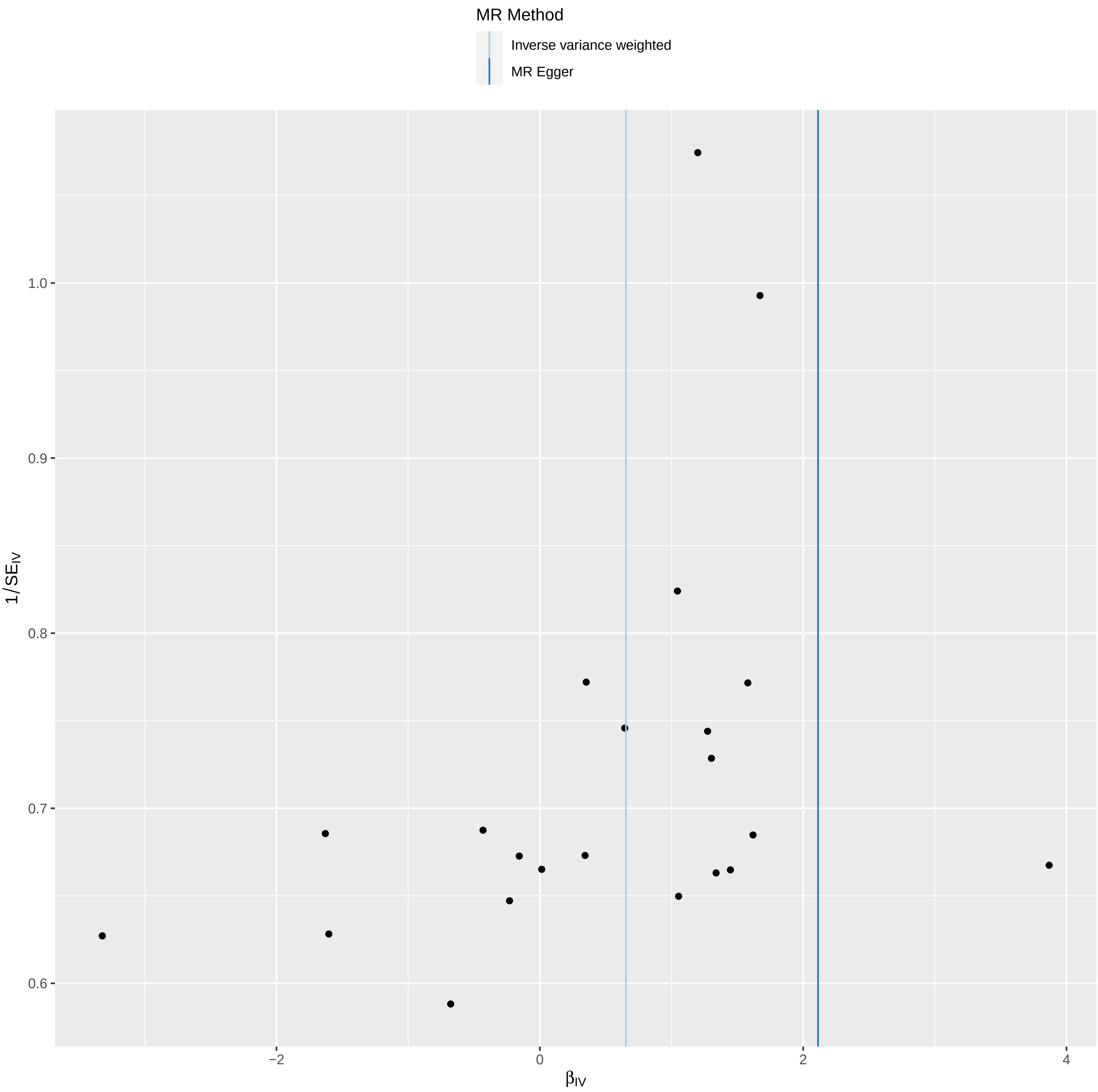

MR Method
Inverse variance weighted
MR Egger
1.0
0.9
0.8
0.7
0.6
−2
0
2
4
$1/SE_{IV}$
β_{IV}

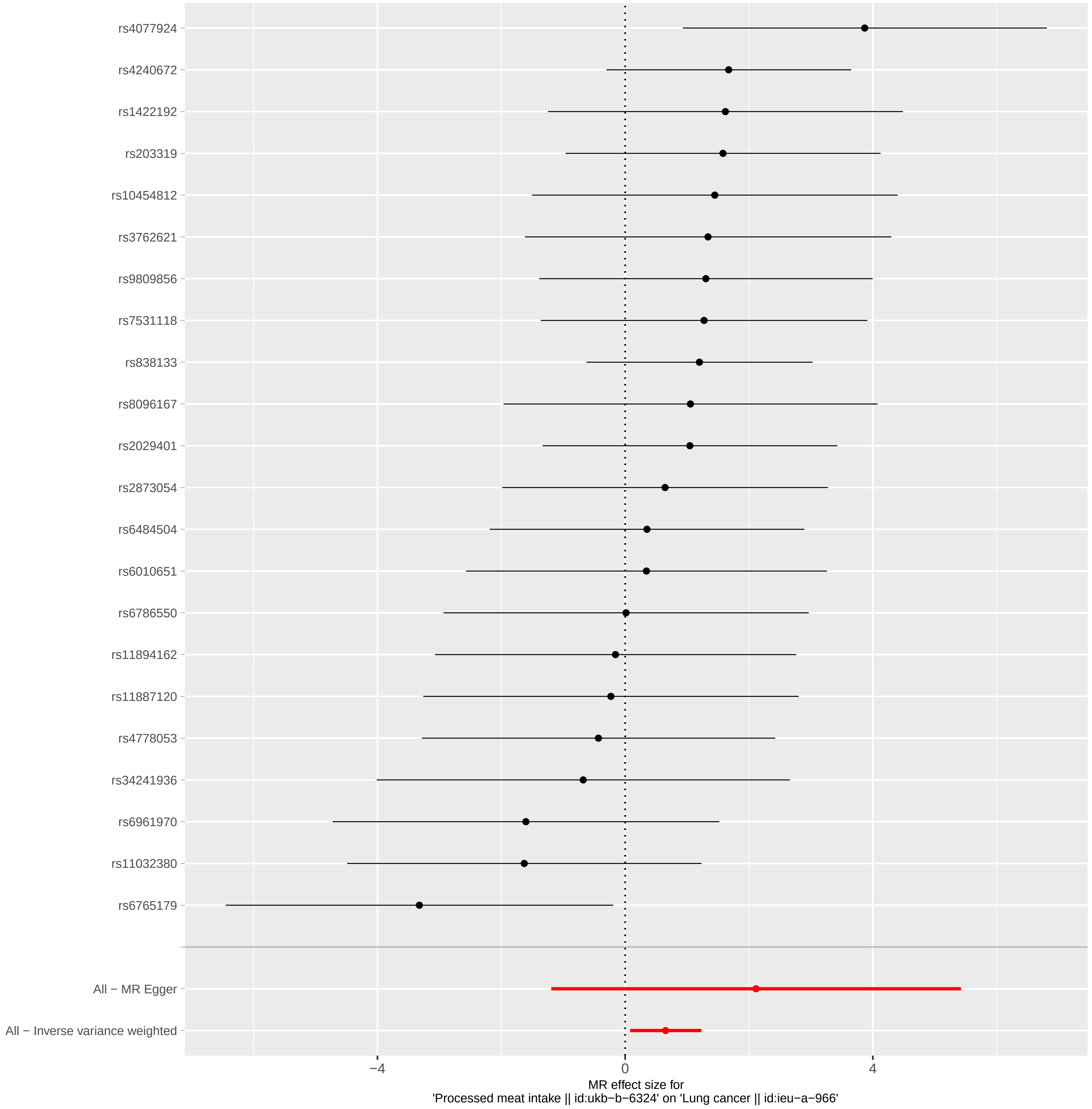

rs4077924
rs4240672
rs1422192
rs203319
rs10454812
rs3762621
rs9809856
rs7531118
rs838133
rs8096167
rs2029401
rs2873054
rs6484504
rs6010651
rs6786550
rs11894162
rs11887120
rs4778053
rs34241936
rs6961970
rs11032380
rs6765179
All − MR Egger
All − Inverse variance weighted
−4
0
4
MR effect size for
'Processed meat intake || id:ukb−b−6324' on 'Lung cancer || id:ieu−a−966'

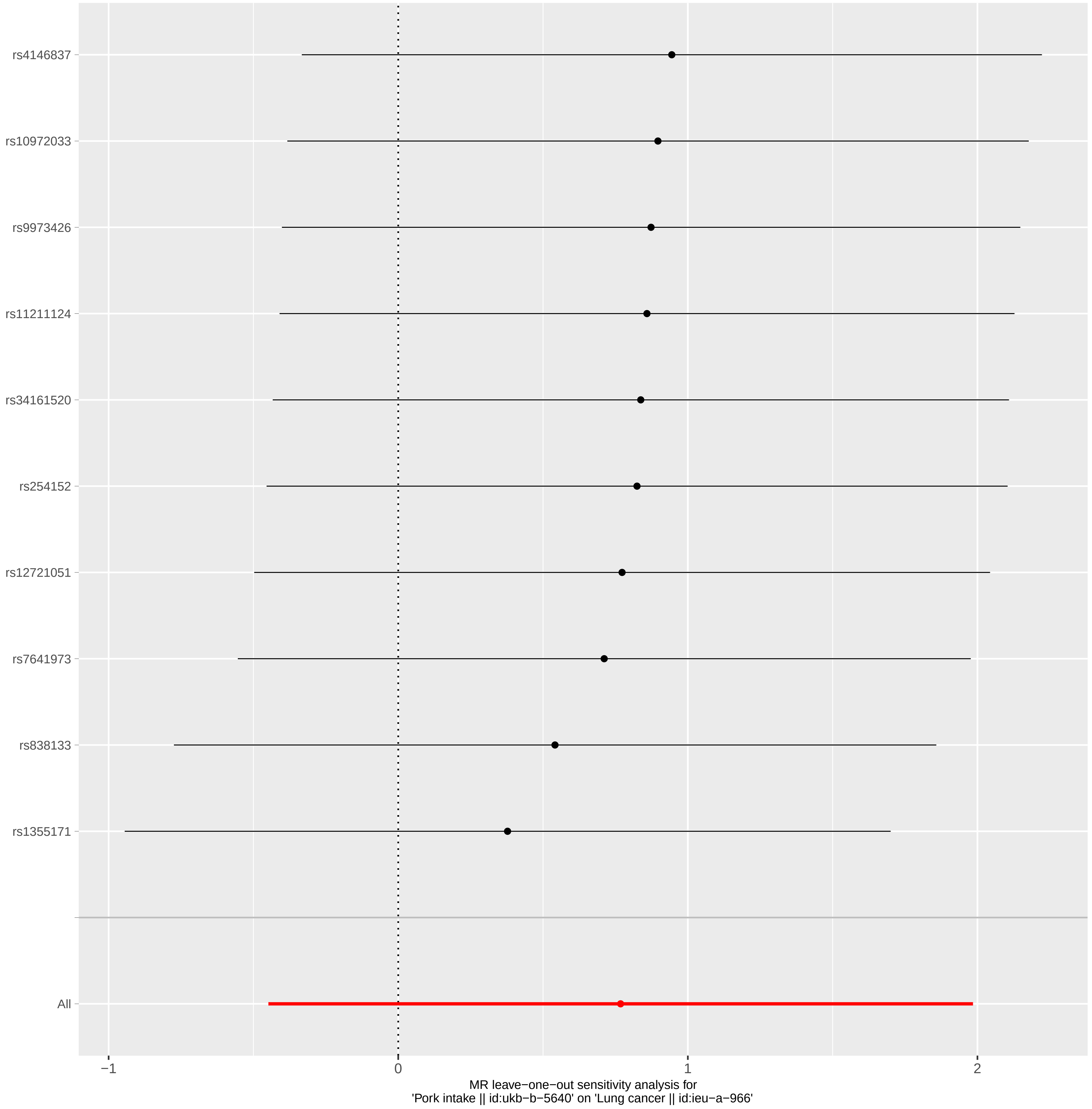
rs4146837
rs10972033
rs9973426
rs11211124
rs34161520
rs254152
rs12721051
rs7641973
rs838133
rs1355171
All
−1
0
1
2
MR leave−one−out sensitivity analysis for
'Pork intake || id:ukb−b−5640' on 'Lung cancer || id:ieu−a−966'

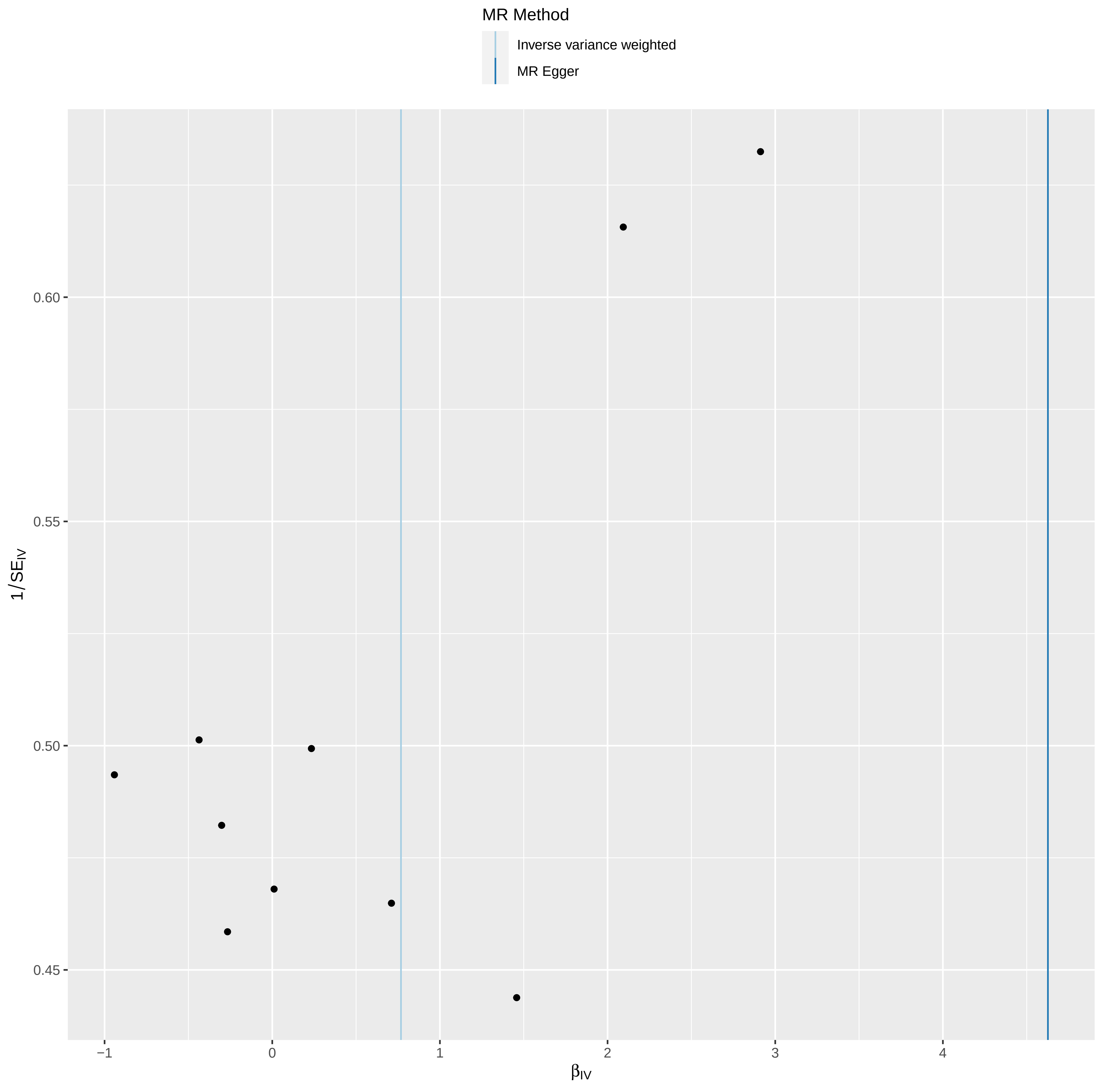
MR Method
Inverse variance weighted
MR Egger
0.60
0.55
0.50
0.45
1/SE$_{IV}$
−1
0
1
2
3
4
β$_{IV}$

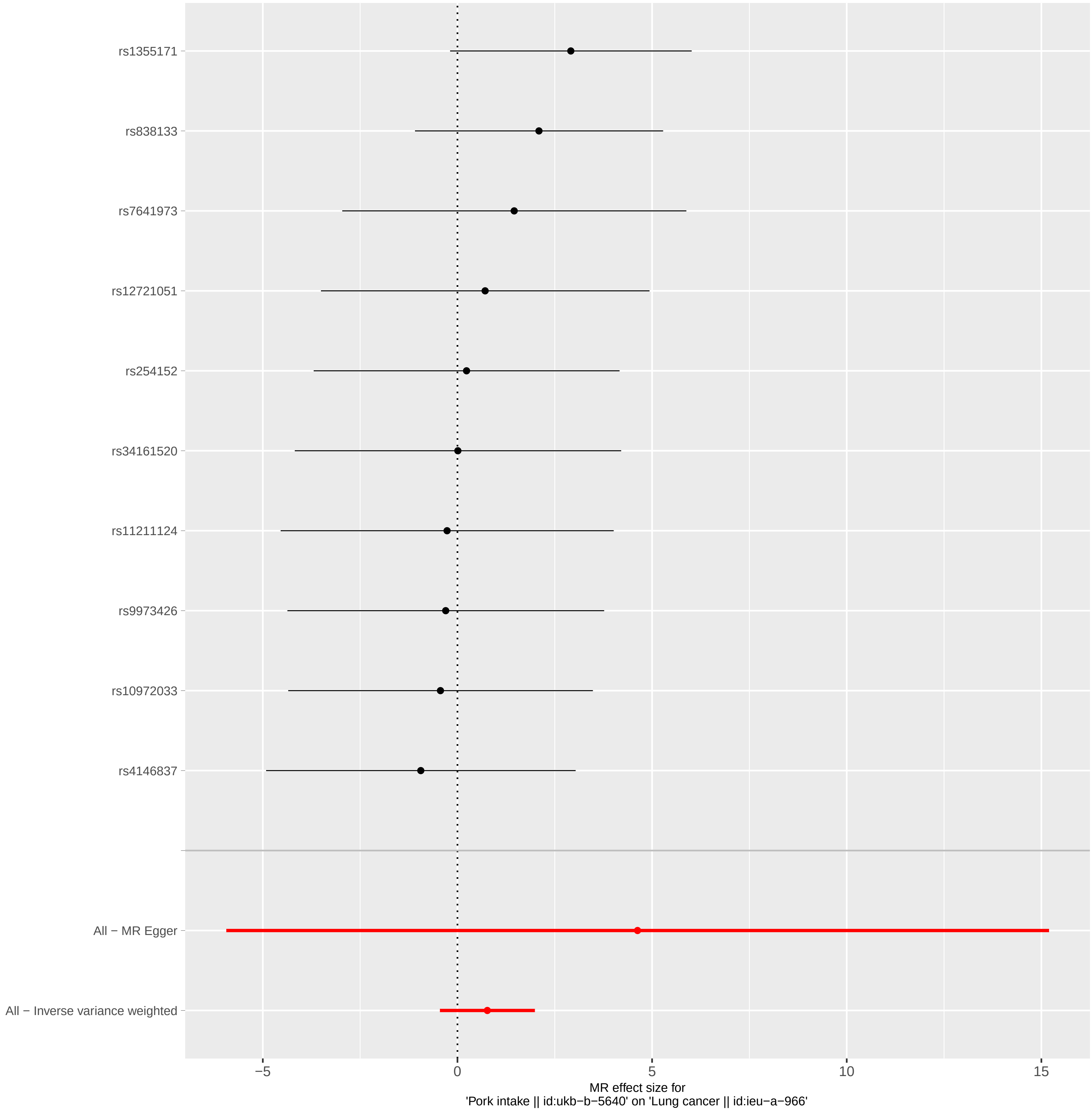
rs1355171
rs838133
rs7641973
rs12721051
rs254152
rs34161520
rs11211124
rs9973426
rs10972033
rs4146837
All − MR Egger
All − Inverse variance weighted
−5
0
5
10
15
MR effect size for
'Pork intake || id:ukb−b−5640' on 'Lung cancer || id:ieu−a−966'

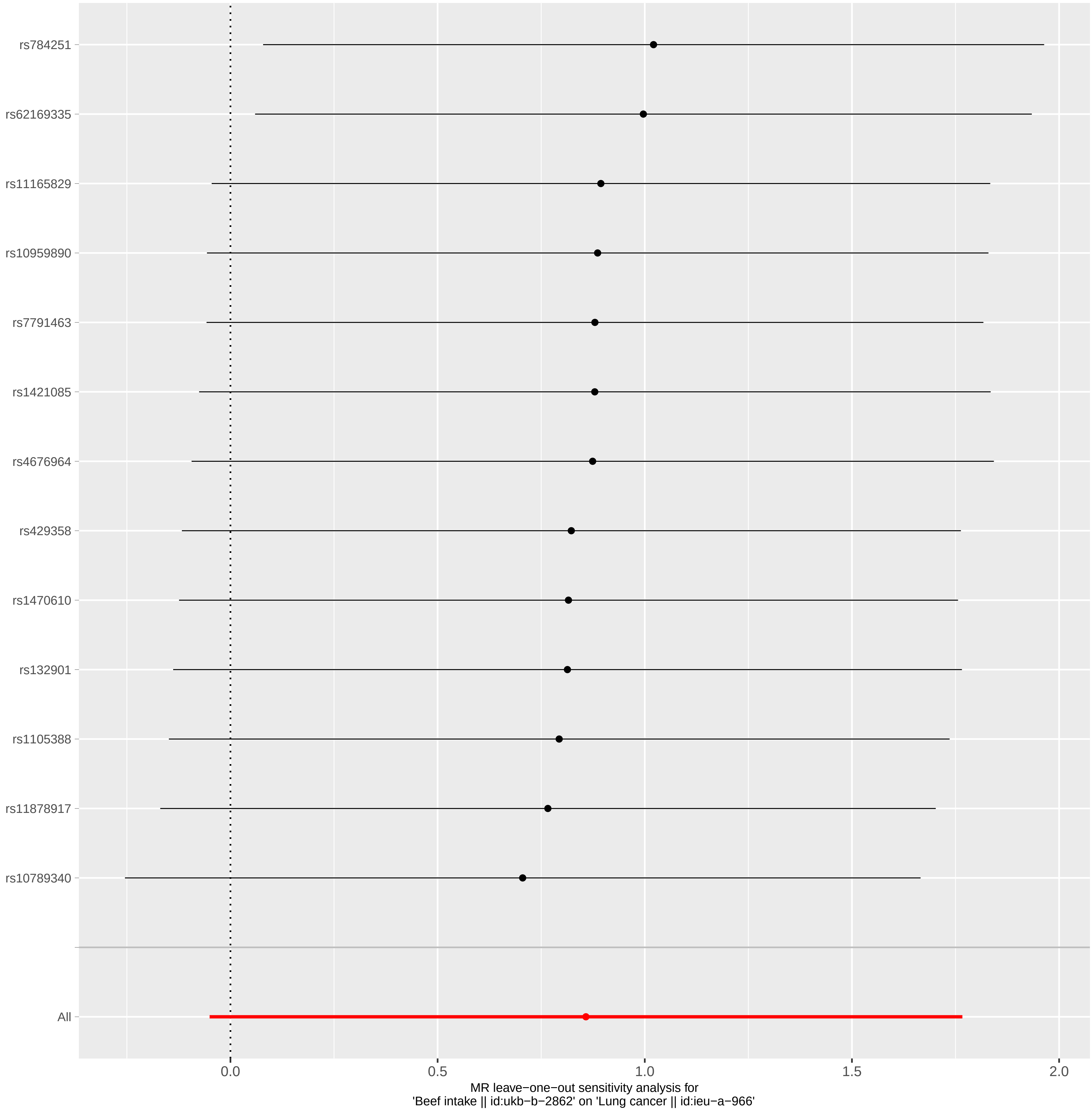

rs784251
rs62169335
rs11165829
rs10959890
rs7791463
rs1421085
rs4676964
rs429358
rs1470610
rs132901
rs1105388
rs11878917
rs10789340
All
0.0
0.5
1.0
1.5
2.0
MR leave−one−out sensitivity analysis for
'Beef intake || id:ukb−b−2862' on 'Lung cancer || id:ieu−a−966'

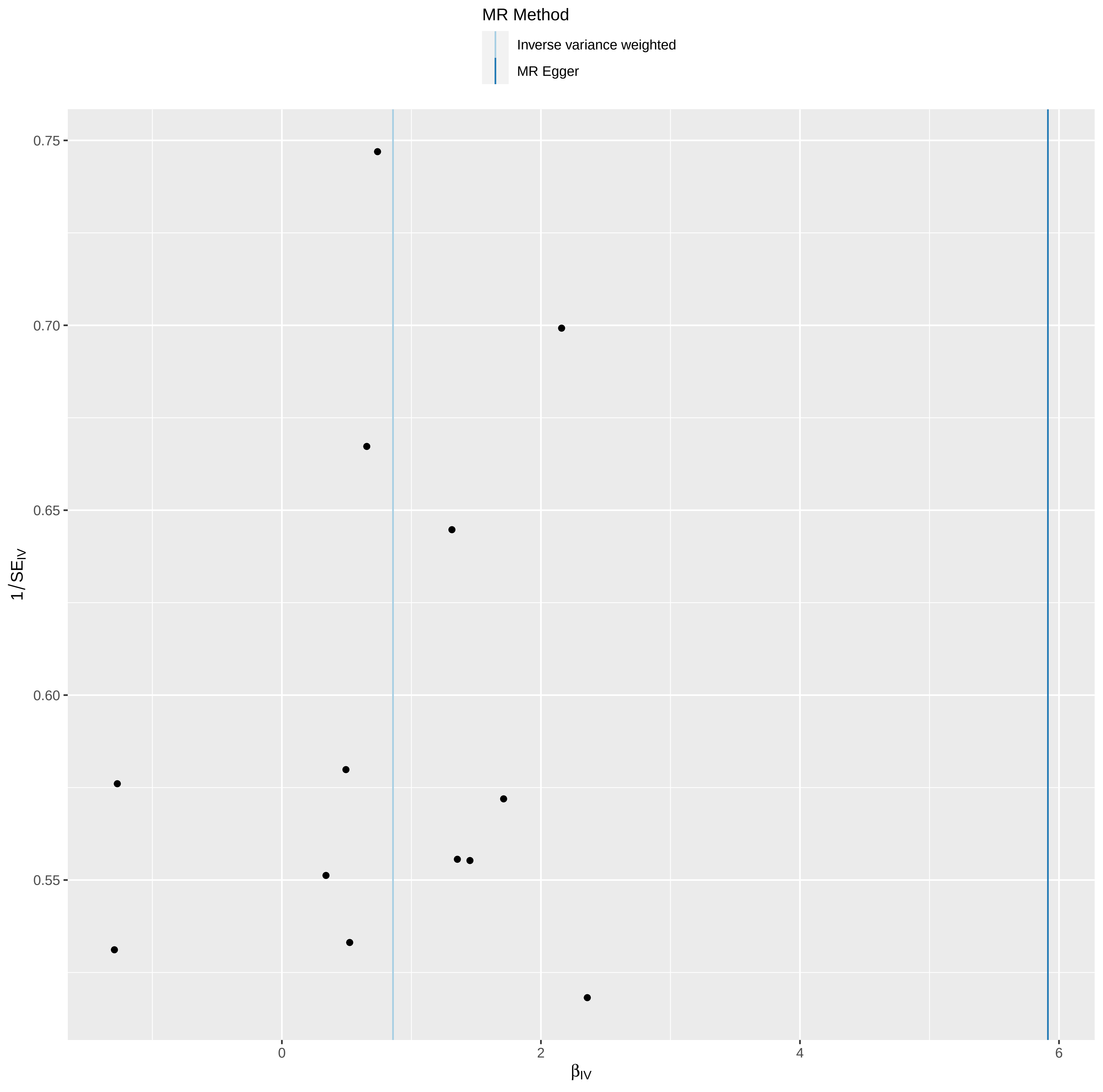

MR Method
Inverse variance weighted
MR Egger
0.75
0.70
0.65
0.60
0.55
$1/SE_{IV}$
0
2
4
6
β_{IV}

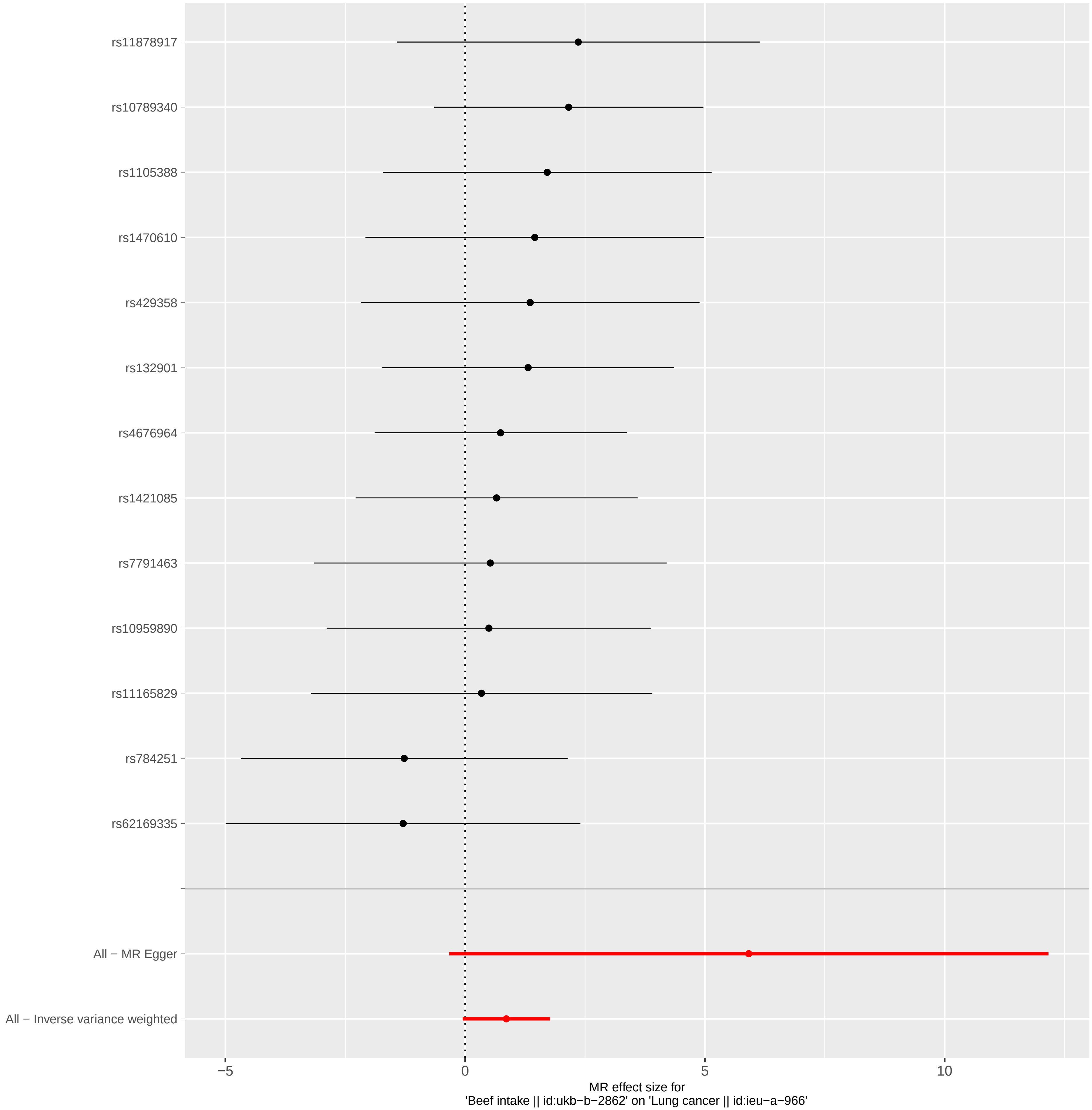

rs11878917
rs10789340
rs1105388
rs1470610
rs429358
rs132901
rs4676964
rs1421085
rs7791463
rs10959890
rs11165829
rs784251
rs62169335
All − MR Egger
All − Inverse variance weighted
−5
0
5
10
MR effect size for
'Beef intake || id:ukb−b−2862' on 'Lung cancer || id:ieu−a−966'

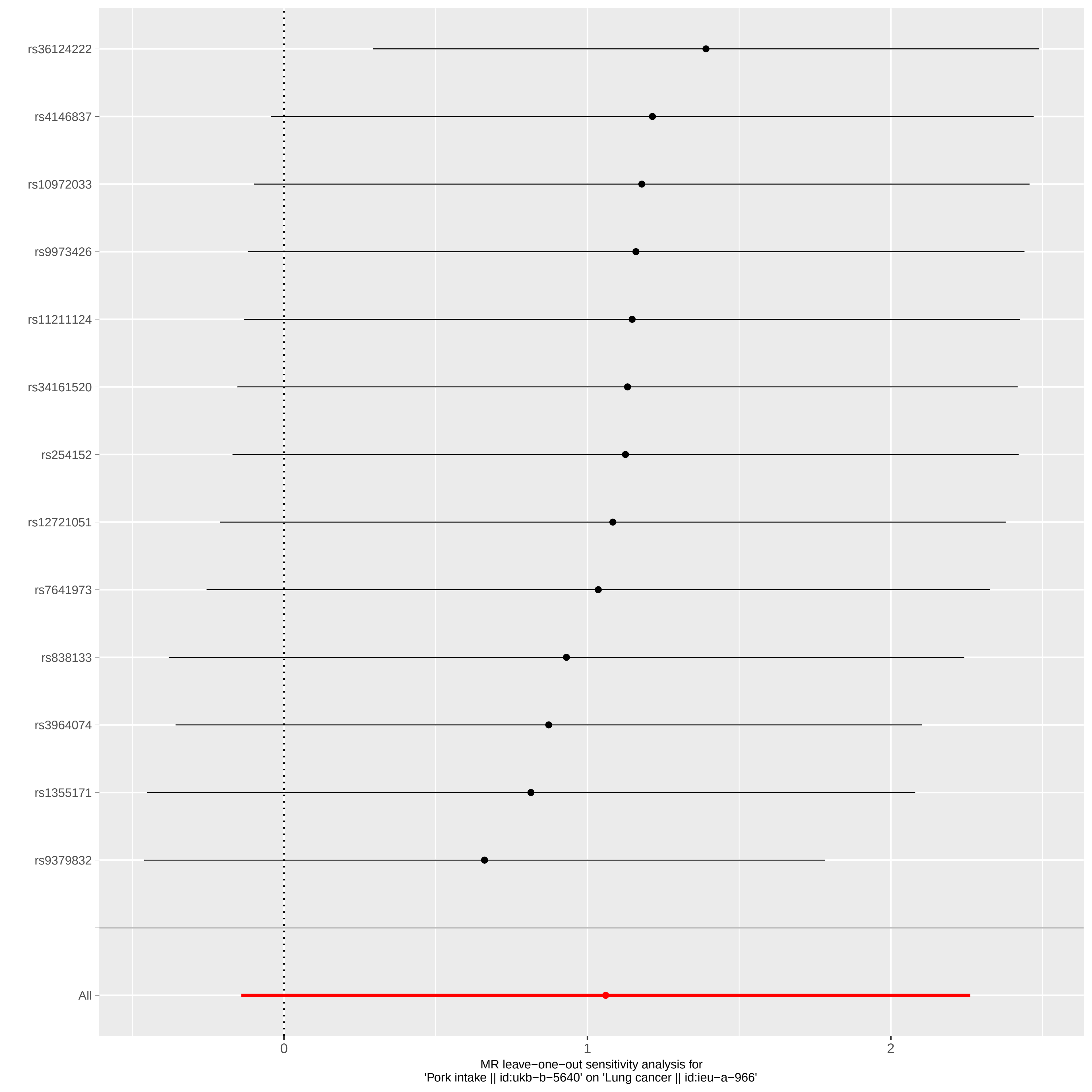
rs36124222
rs4146837
rs10972033
rs9973426
rs11211124
rs34161520
rs254152
rs12721051
rs7641973
rs838133
rs3964074
rs1355171
rs9379832
All
0
1
2
MR leave−one−out sensitivity analysis for
'Pork intake || id:ukb−b−5640' on 'Lung cancer || id:ieu−a−966'

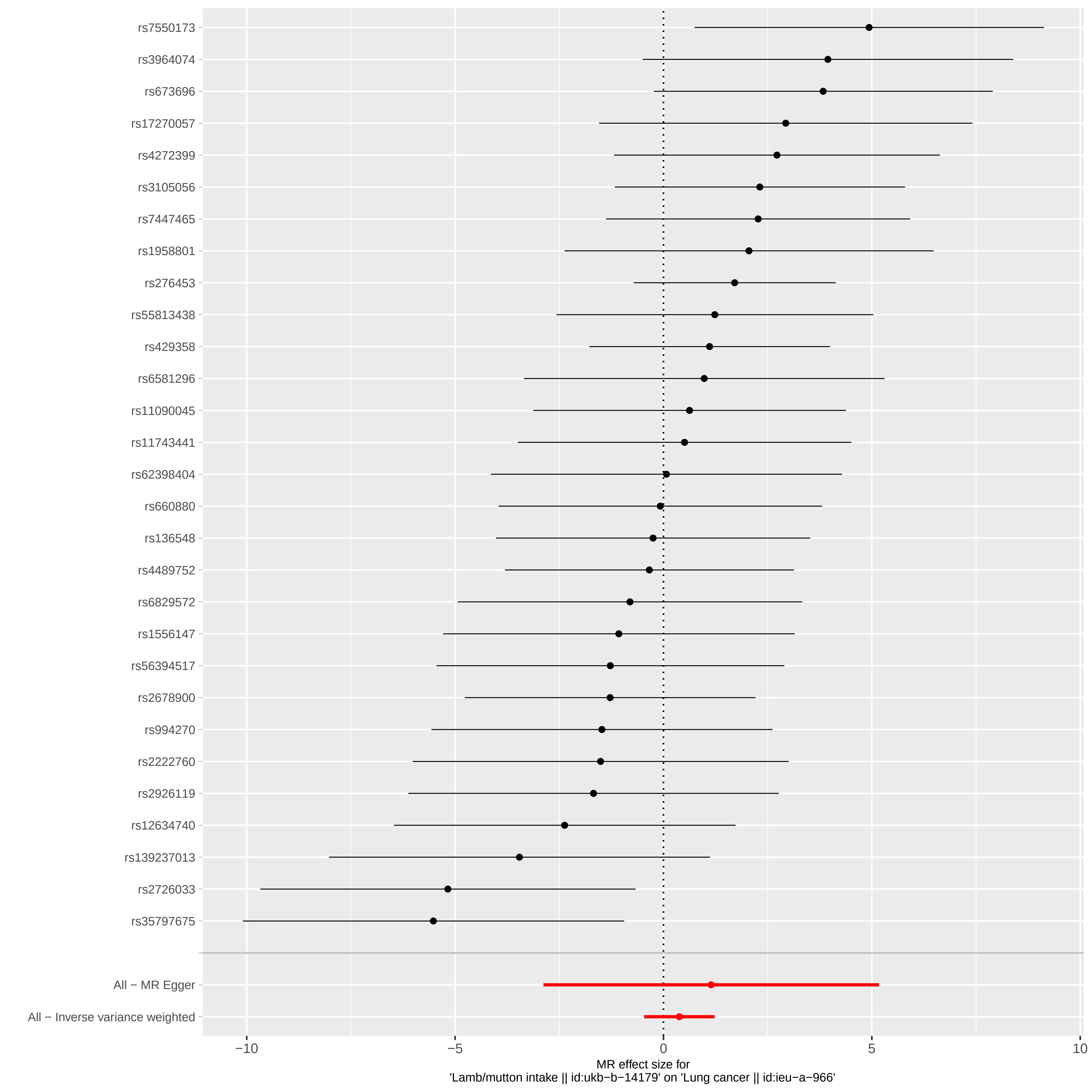
rs7550173
rs3964074
rs673696
rs17270057
rs4272399
rs3105056
rs7447465
rs1958801
rs276453
rs55813438
rs429358
rs6581296
rs11090045
rs11743441
rs62398404
rs660880
rs136548
rs4489752
rs6829572
rs1556147
rs56394517
rs2678900
rs994270
rs2222760
rs2926119
rs12634740
rs139237013
rs2726033
rs35797675
All − MR Egger
All − Inverse variance weighted
−10
−5
0
5
10
MR effect size for
'Lamb/mutton intake || id:ukb−b−14179' on 'Lung cancer || id:ieu−a−966'

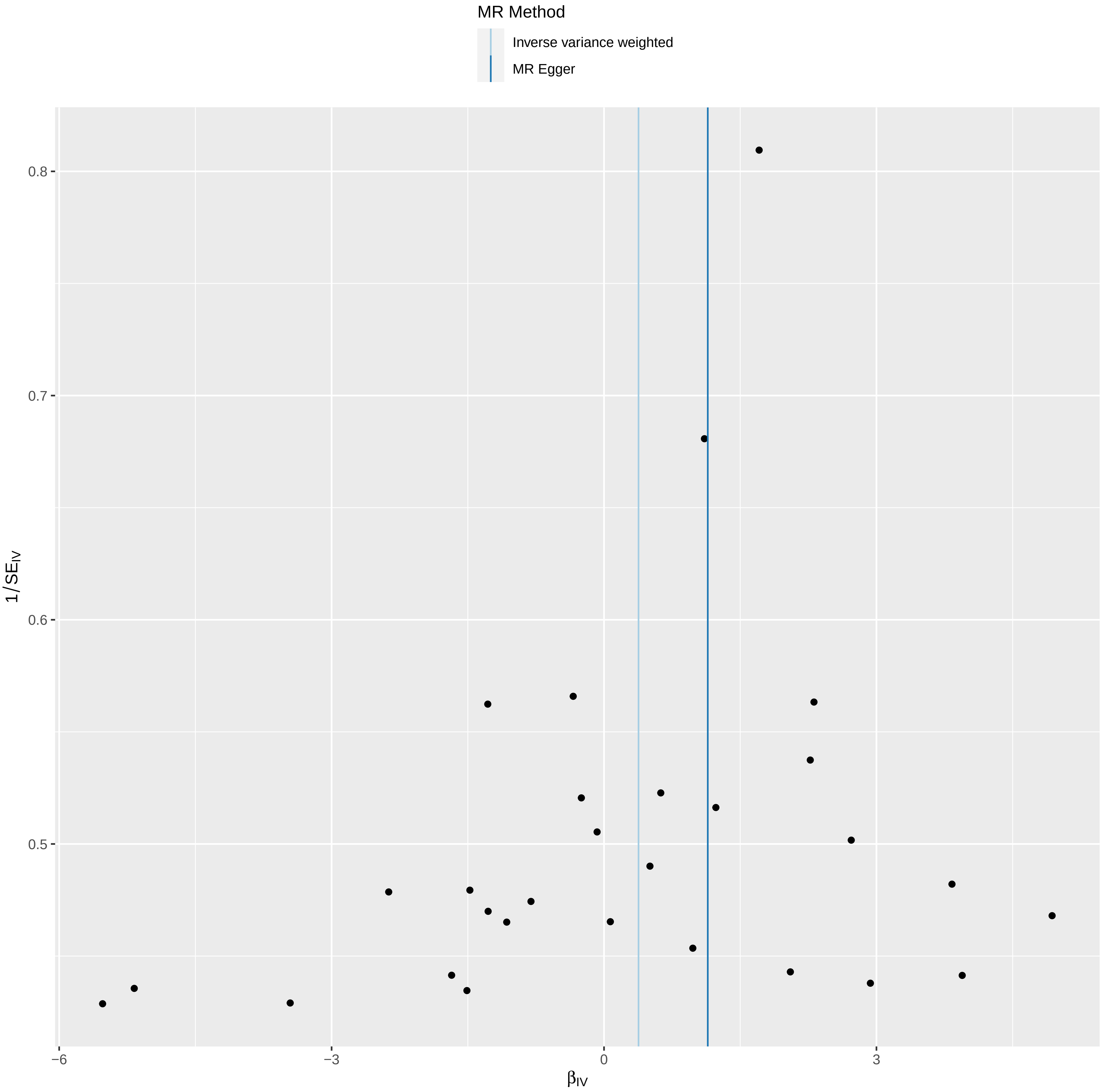
MR Method
Inverse variance weighted
MR Egger
0.8
0.7
0.6
0.5
1/SE_{IV}
−6
−3
0
3
$β_{IV}$

Figure S2. Leave-one-out analysis, funnel plot and MR effect size for processed meat, pork, beef and mutton intake on ovarian cancer.

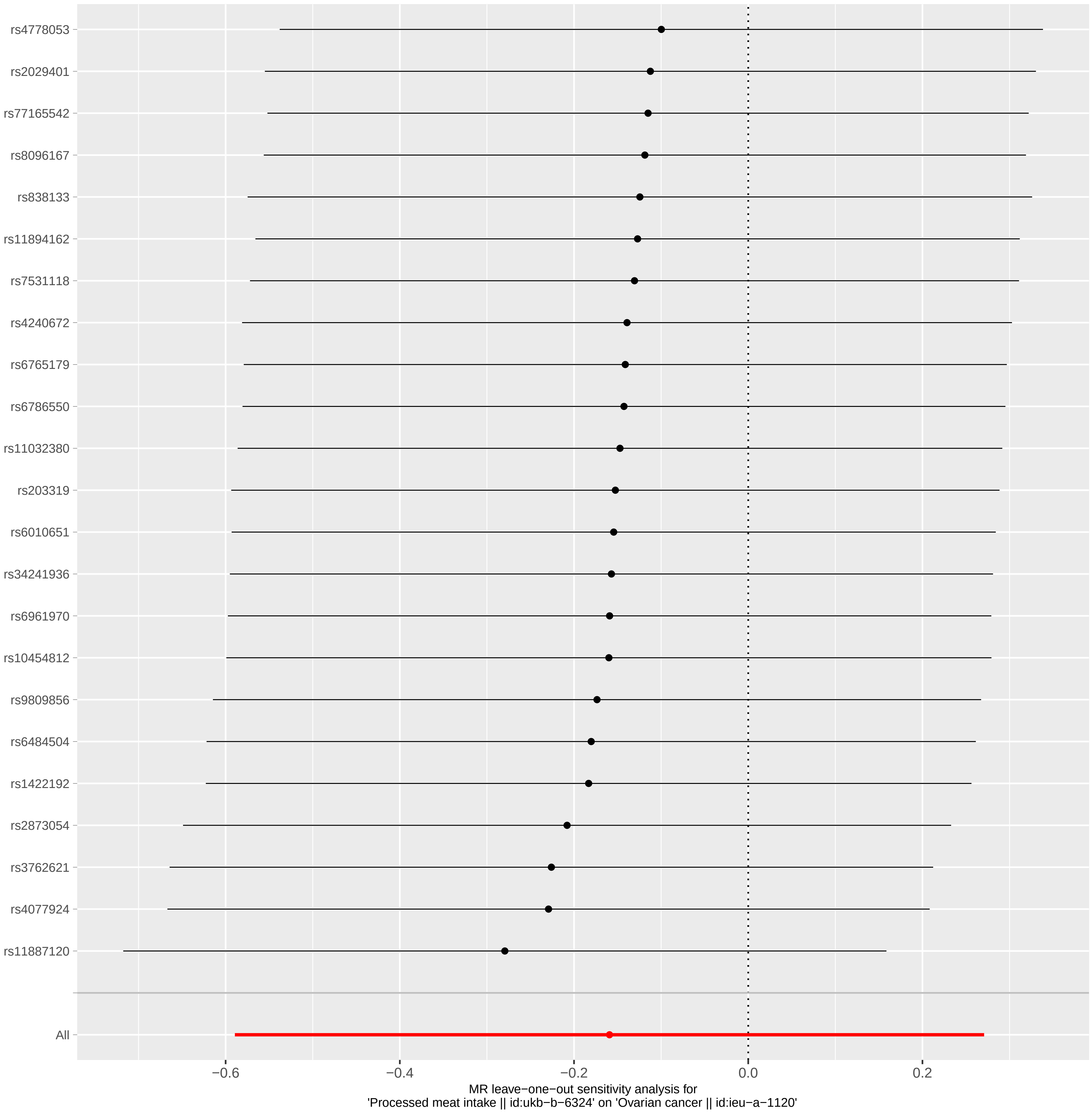

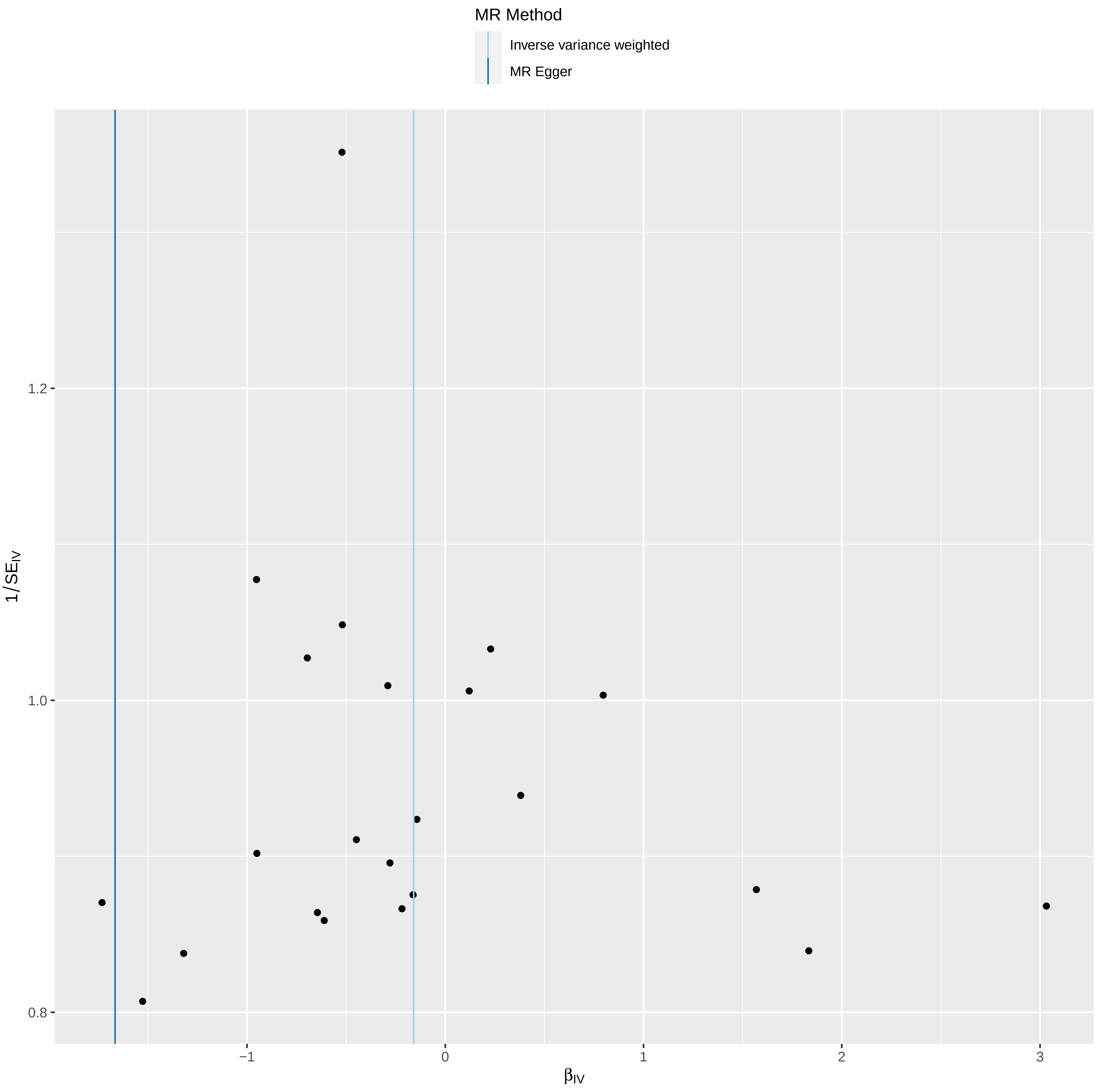

MR Method
Inverse variance weighted
MR Egger
1.2
1.0
0.8
1/SE_IV
−1
0
1
2
3
β_IV

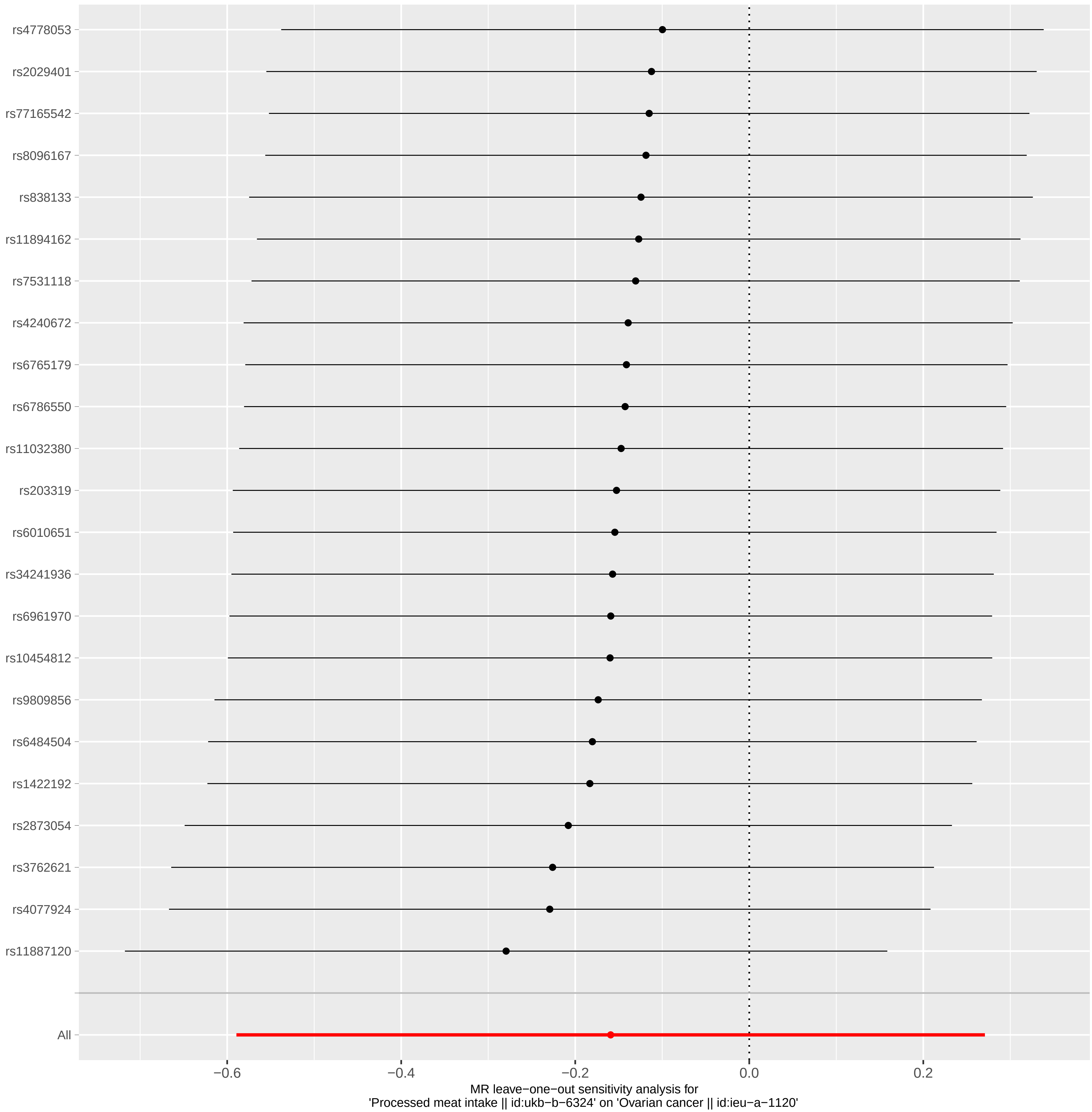
rs4778053
rs2029401
rs77165542
rs8096167
rs838133
rs11894162
rs7531118
rs4240672
rs6765179
rs6786550
rs11032380
rs203319
rs6010651
rs34241936
rs6961970
rs10454812
rs9809856
rs6484504
rs1422192
rs2873054
rs3762621
rs4077924
rs11887120
All
−0.6
−0.4
−0.2
0.0
0.2
MR leave−one−out sensitivity analysis for
'Processed meat intake || id:ukb−b−6324' on 'Ovarian cancer || id:ieu−a−1120'

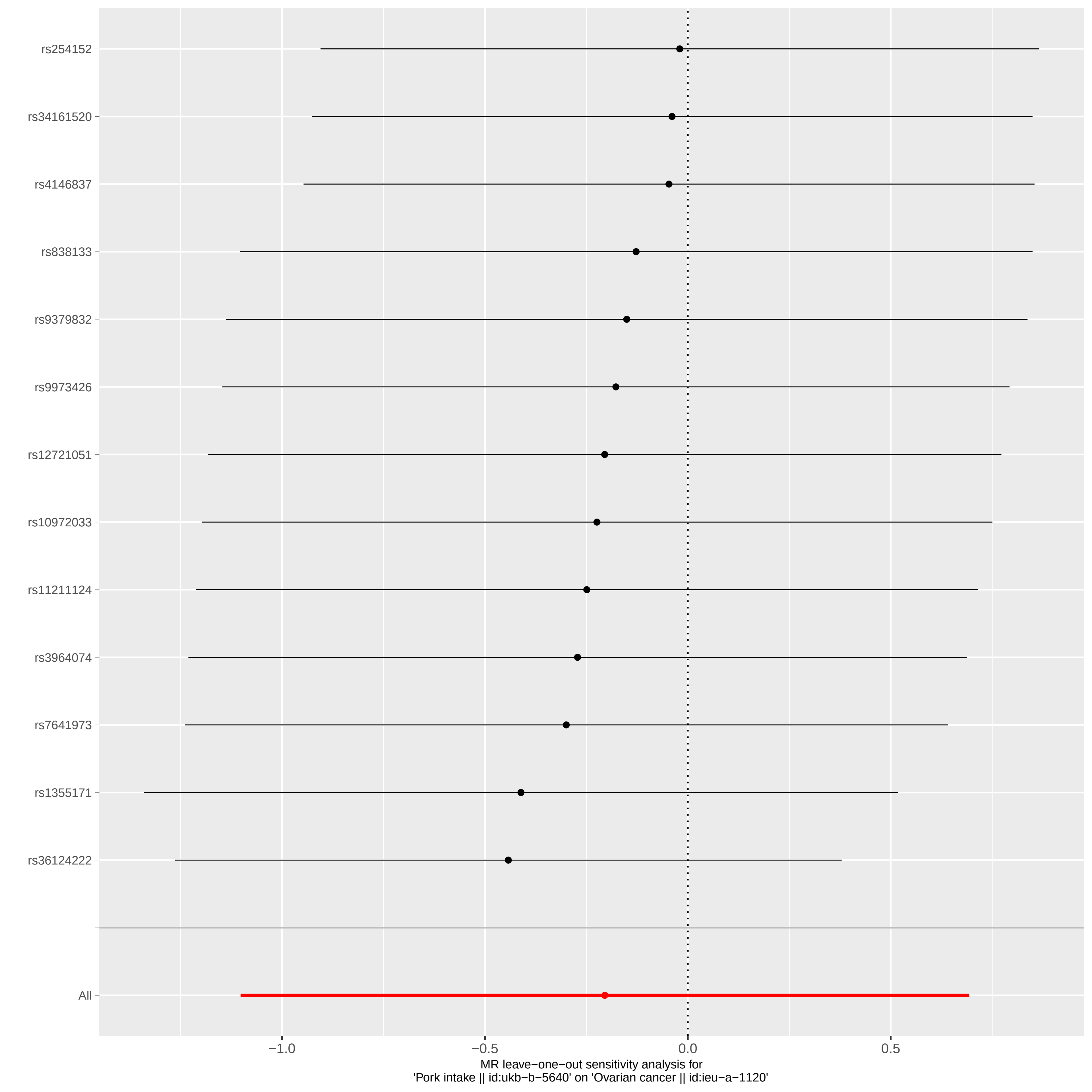

rs254152
rs34161520
rs4146837
rs838133
rs9379832
rs9973426
rs12721051
rs10972033
rs11211124
rs3964074
rs7641973
rs1355171
rs36124222
All
−1.0
−0.5
0.0
0.5
MR leave−one−out sensitivity analysis for
'Pork intake || id:ukb−b−5640' on 'Ovarian cancer || id:ieu−a−1120'

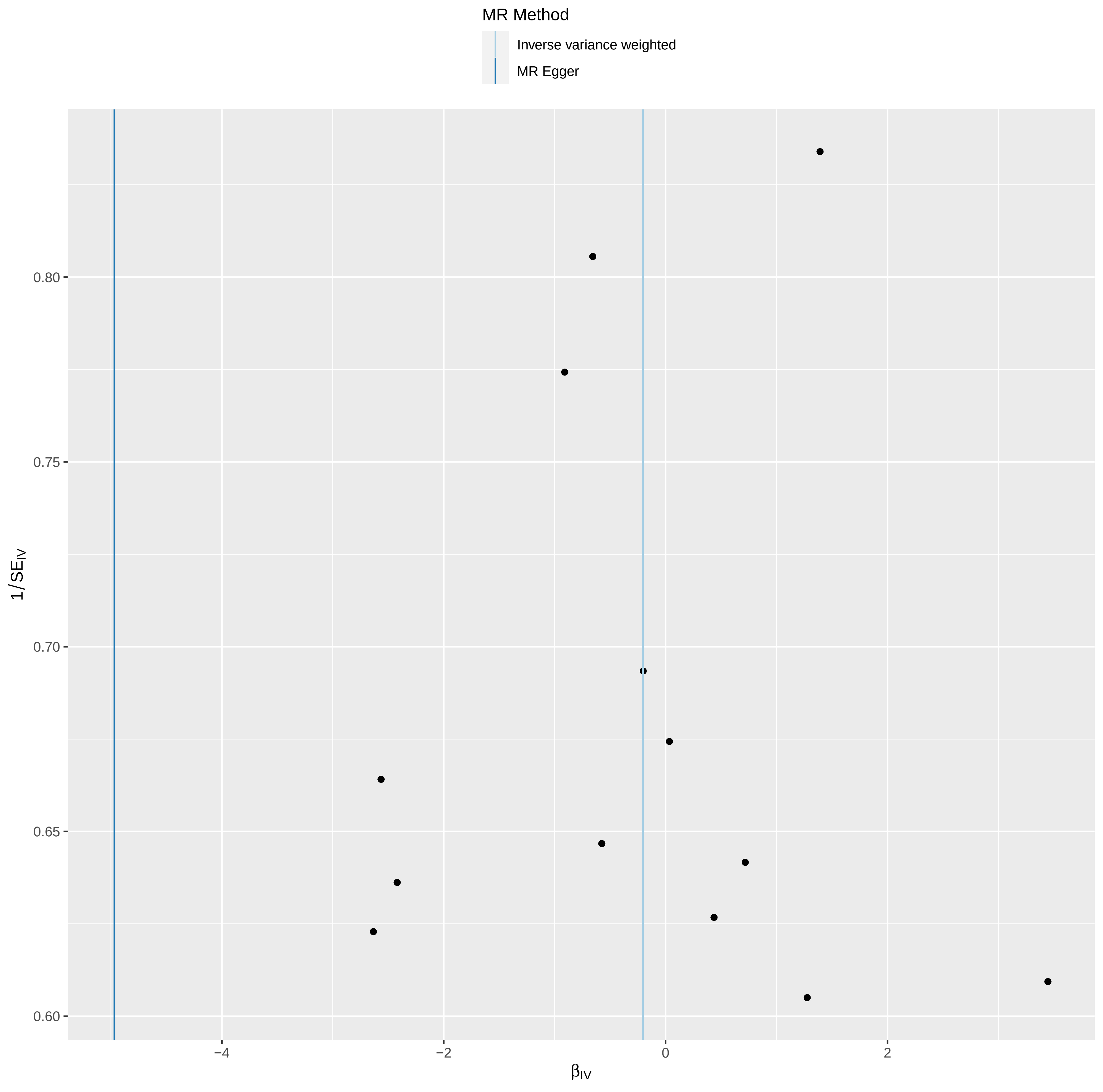

MR Method
Inverse variance weighted
MR Egger
0.80
0.75
0.70
0.65
0.60
1/SE_IV
−4
−2
0
2
β_IV

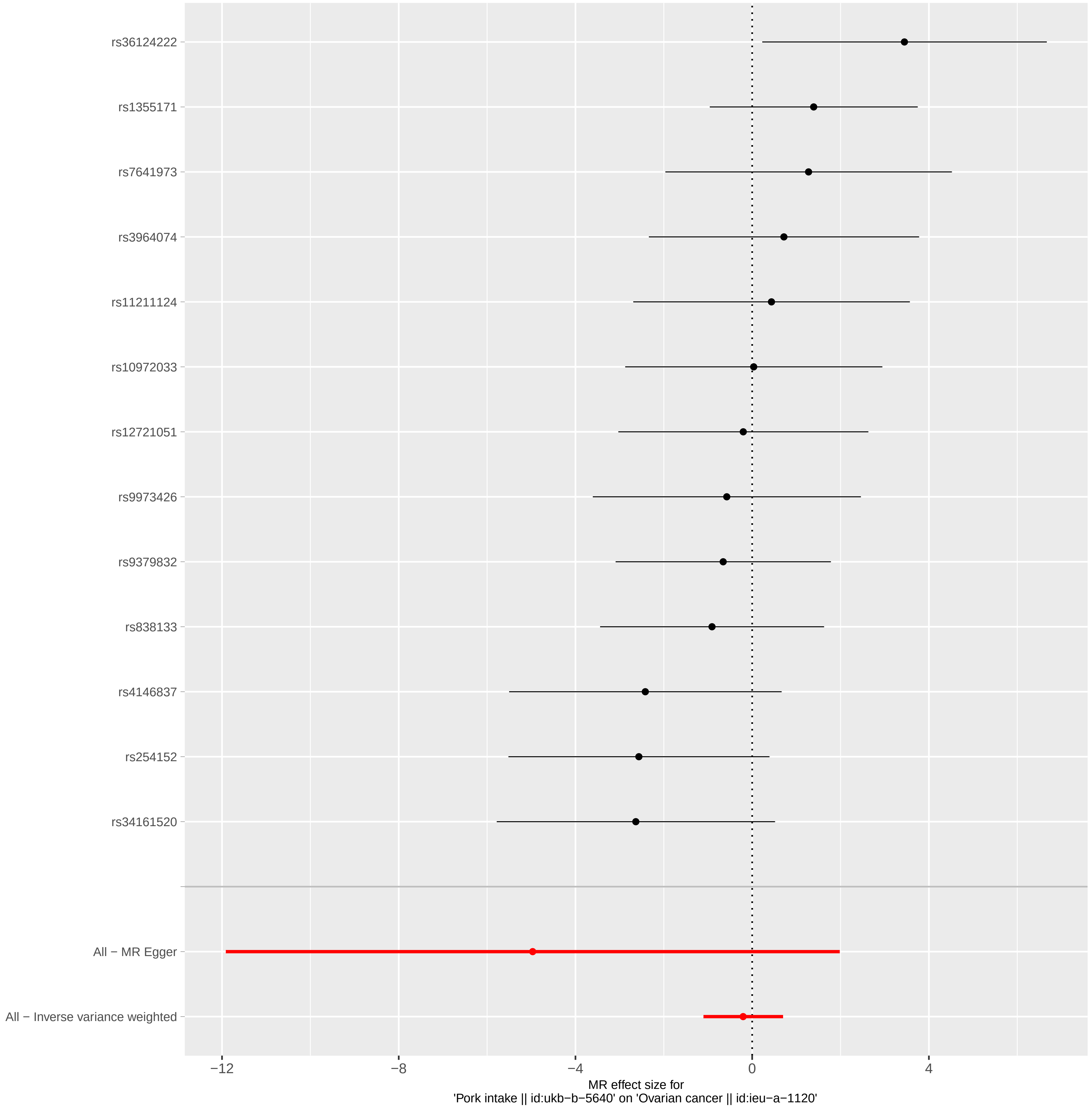
rs36124222
rs1355171
rs7641973
rs3964074
rs11211124
rs10972033
rs12721051
rs9973426
rs9379832
rs838133
rs4146837
rs254152
rs34161520
All − MR Egger
All − Inverse variance weighted
−12
−8
−4
0
4
MR effect size for
'Pork intake || id:ukb−b−5640' on 'Ovarian cancer || id:ieu−a−1120'

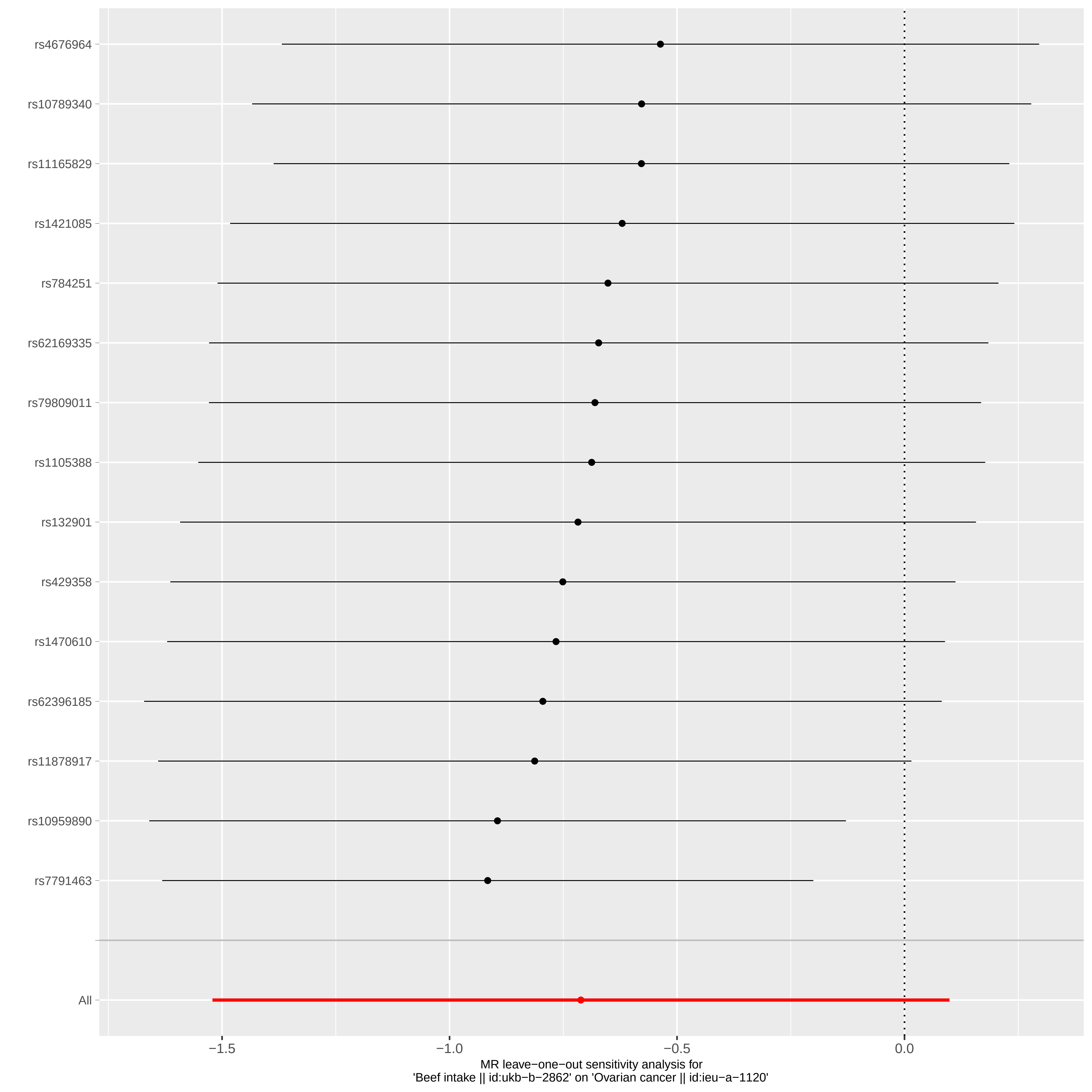
rs4676964
rs10789340
rs11165829
rs1421085
rs784251
rs62169335
rs79809011
rs1105388
rs132901
rs429358
rs1470610
rs62396185
rs11878917
rs10959890
rs7791463
All
−1.5
−1.0
−0.5
0.0
MR leave−one−out sensitivity analysis for
'Beef intake || id:ukb−b−2862' on 'Ovarian cancer || id:ieu−a−1120'

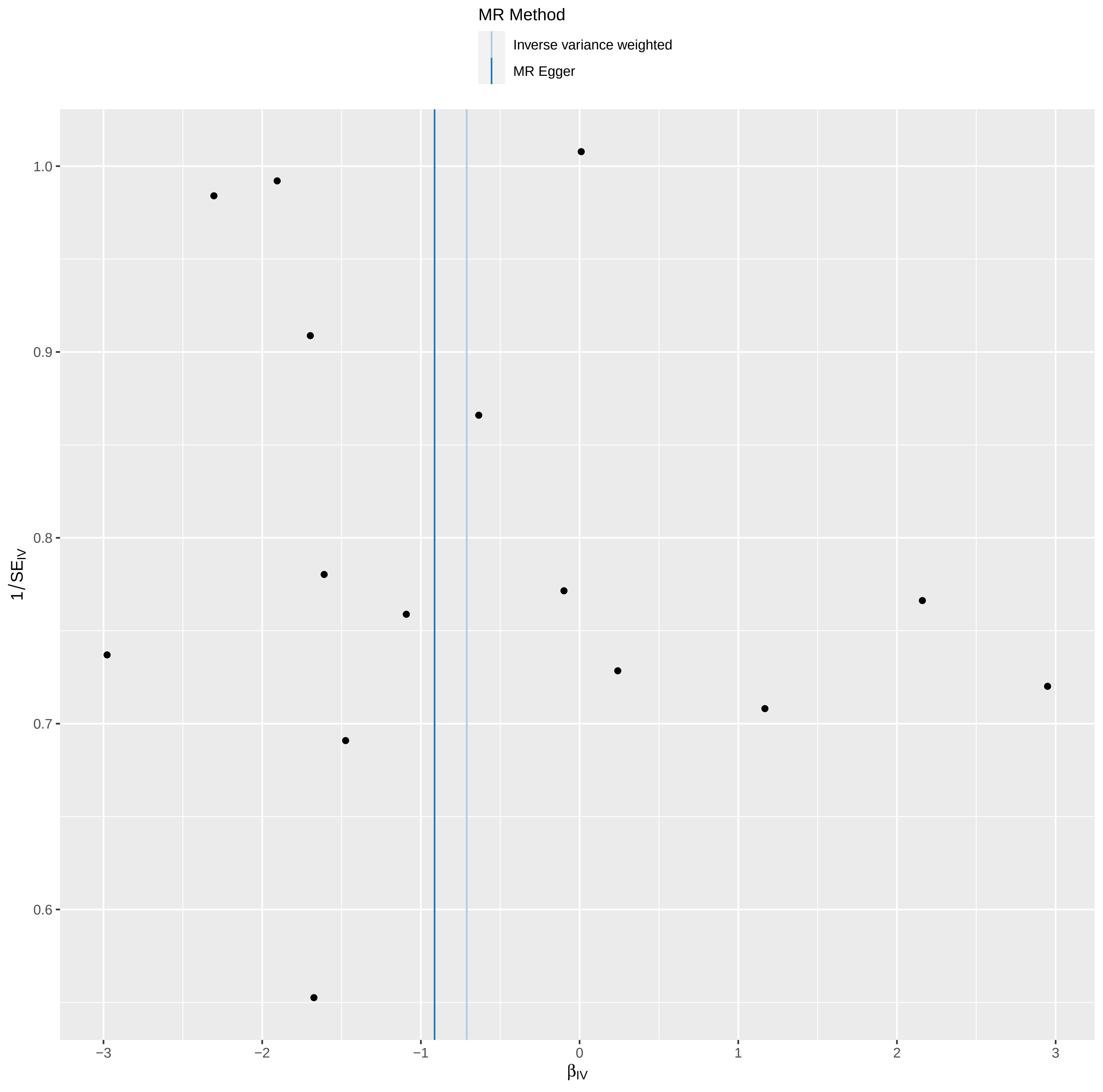
MR Method
Inverse variance weighted
MR Egger
1.0
0.9
0.8
0.7
0.6
1/SE_IV
−3
−2
−1
0
1
2
3
β_IV

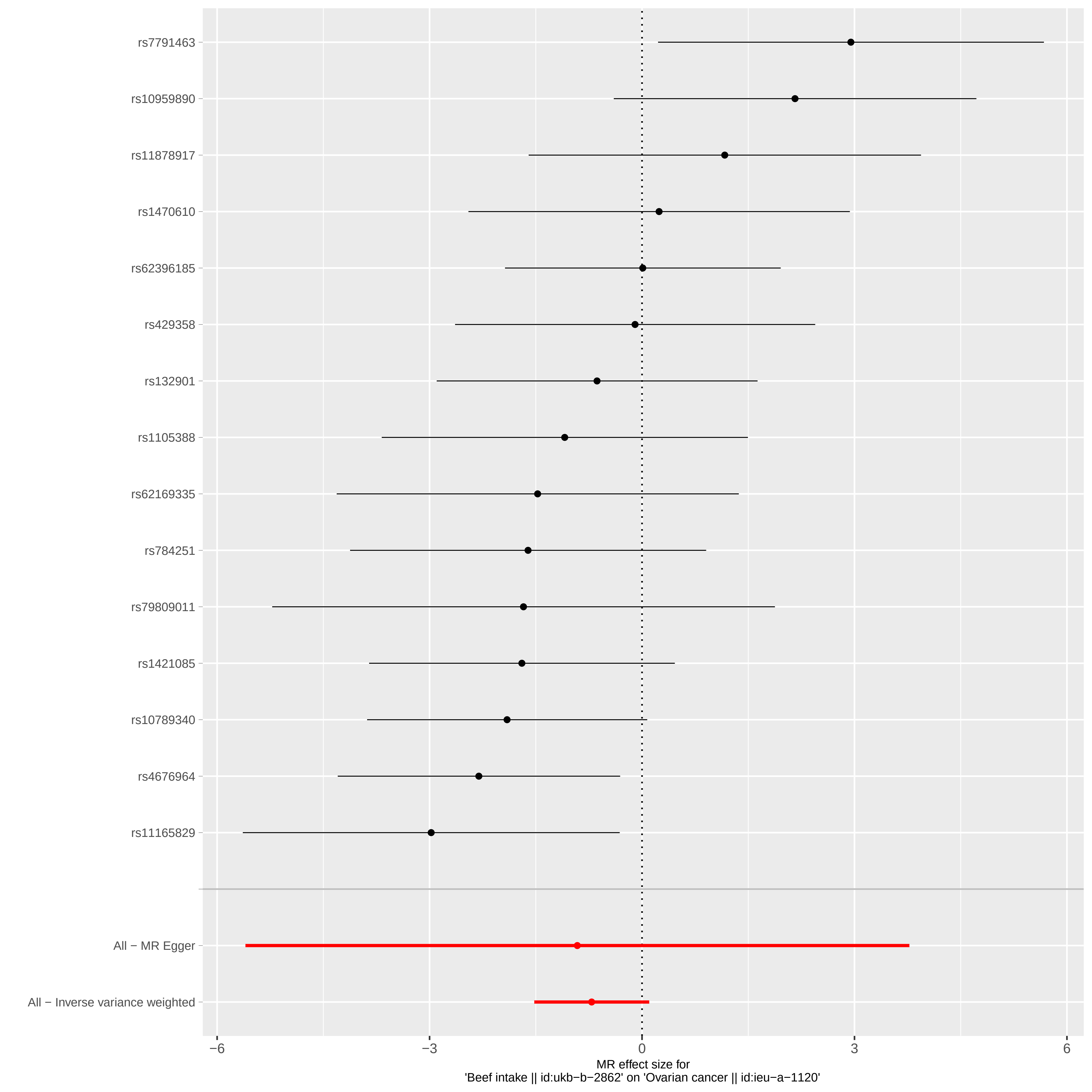
rs7791463
rs10959890
rs11878917
rs1470610
rs62396185
rs429358
rs132901
rs1105388
rs62169335
rs784251
rs79809011
rs1421085
rs10789340
rs4676964
rs11165829
All − MR Egger
All − Inverse variance weighted
−6
−3
0
3
6
MR effect size for
'Beef intake || id:ukb−b−2862' on 'Ovarian cancer || id:ieu−a−1120'

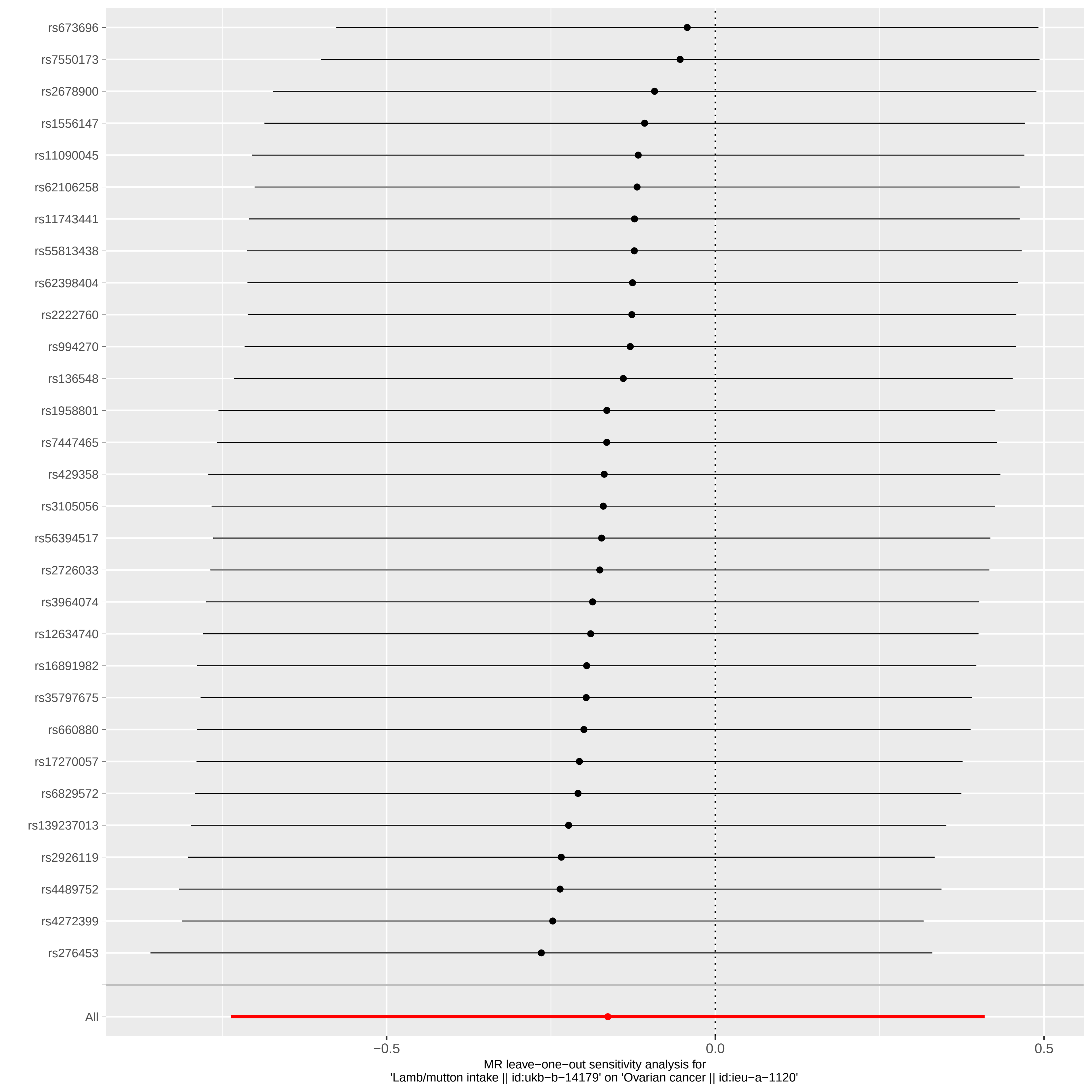
rs673696
rs7550173
rs2678900
rs1556147
rs11090045
rs62106258
rs11743441
rs55813438
rs62398404
rs2222760
rs994270
rs136548
rs1958801
rs7447465
rs429358
rs3105056
rs56394517
rs2726033
rs3964074
rs12634740
rs16891982
rs35797675
rs660880
rs17270057
rs6829572
rs139237013
rs2926119
rs4489752
rs4272399
rs276453
All
−0.5
0.0
0.5
MR leave−one−out sensitivity analysis for
'Lamb/mutton intake || id:ukb−b−14179' on 'Ovarian cancer || id:ieu−a−1120'

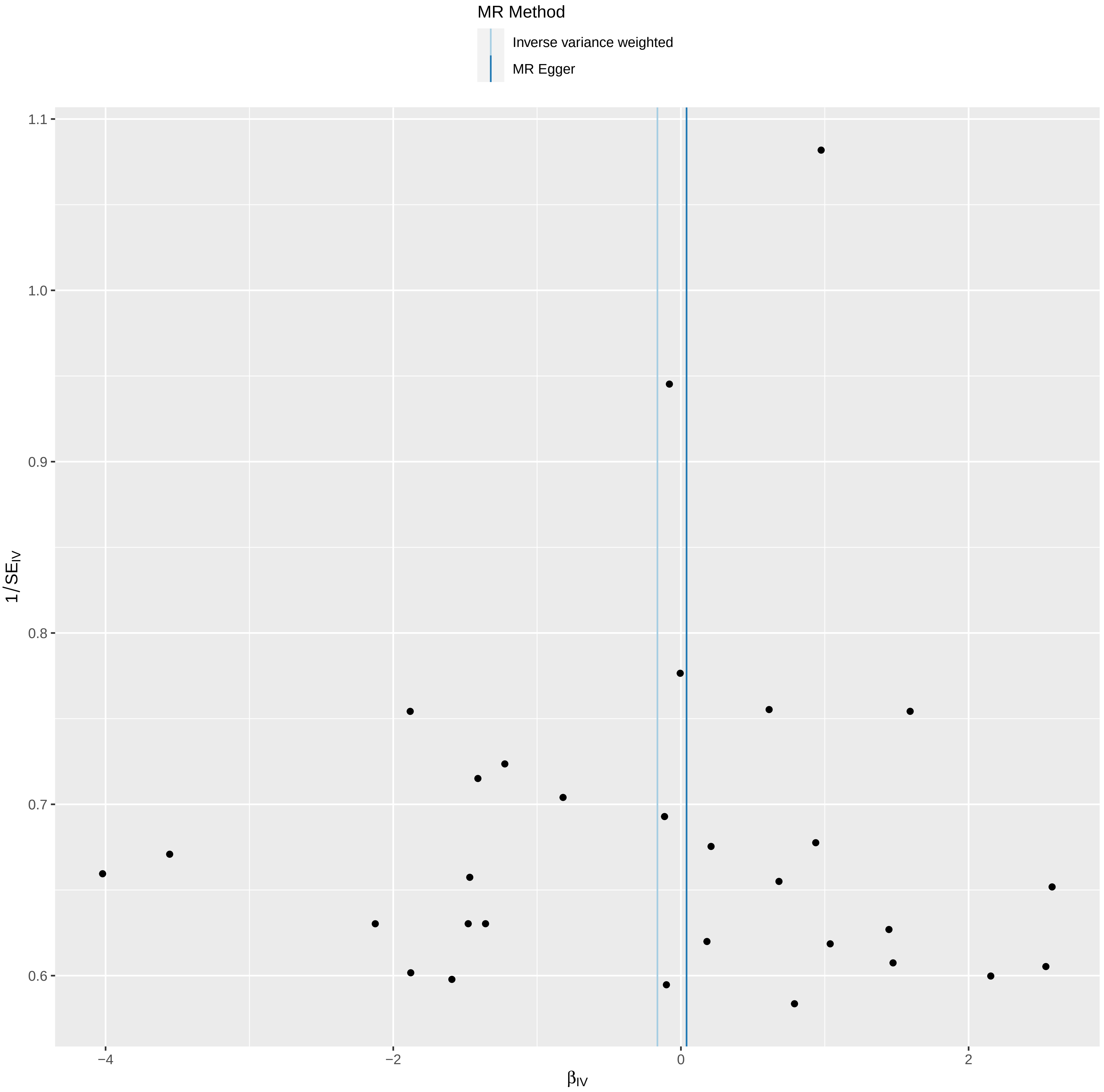

MR Method
Inverse variance weighted
MR Egger
1.1
1.0
0.9
0.8
0.7
0.6
1/SE_IV
−4
−2
0
2
β_IV

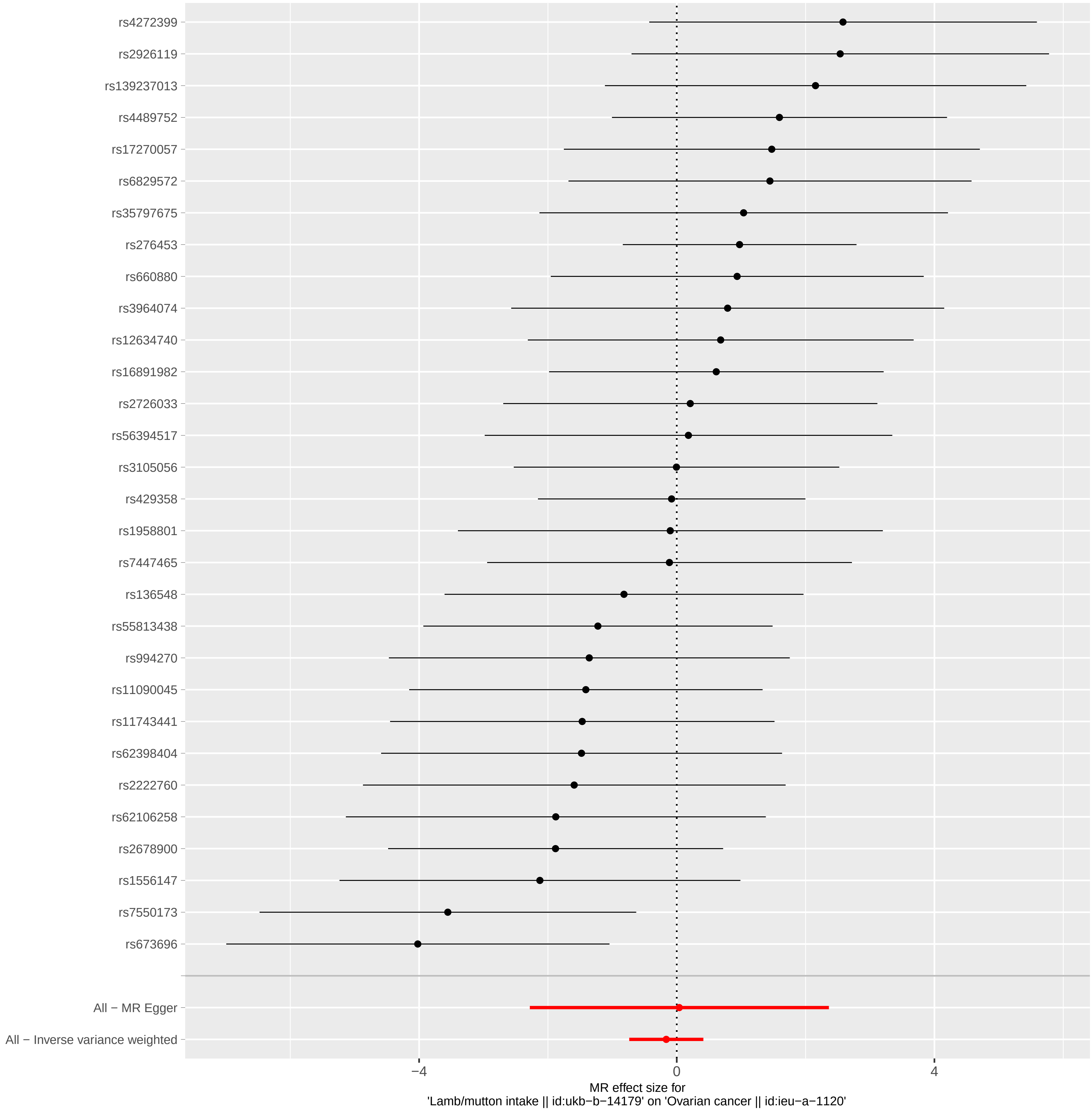

rs4272399
rs2926119
rs139237013
rs4489752
rs17270057
rs6829572
rs35797675
rs276453
rs660880
rs3964074
rs12634740
rs16891982
rs2726033
rs56394517
rs3105056
rs429358
rs1958801
rs7447465
rs136548
rs55813438
rs994270
rs11090045
rs11743441
rs62398404
rs2222760
rs62106258
rs2678900
rs1556147
rs7550173
rs673696
All − MR Egger
All − Inverse variance weighted
−4
0
4
MR effect size for
'Lamb/mutton intake || id:ukb−b−14179' on 'Ovarian cancer || id:ieu−a−1120'

Figure S3. Leave-one-out analysis, funnel plot and MR effect size for processed meat, pork, beef and mutton intake on endometrial carcinoma.

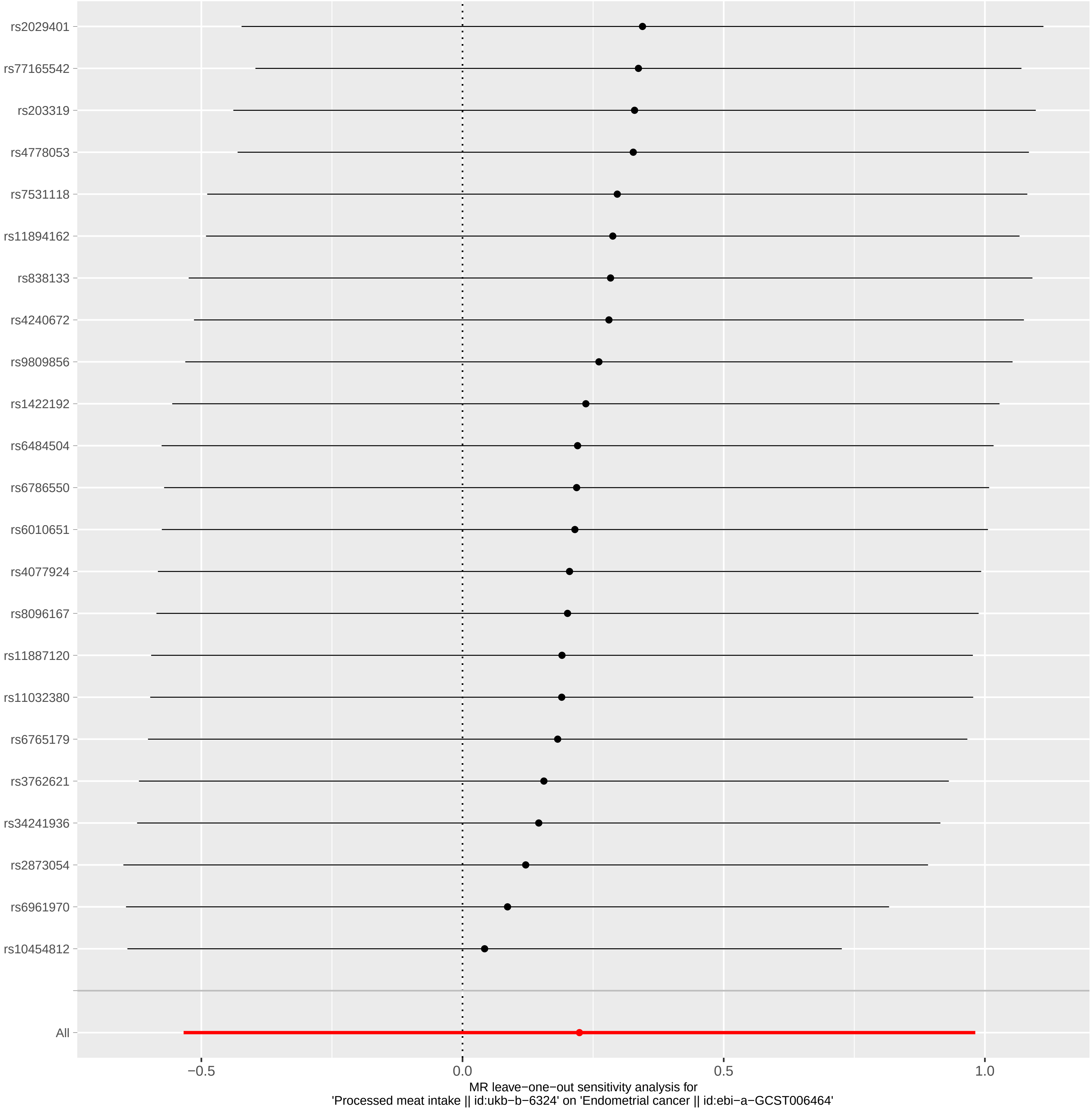

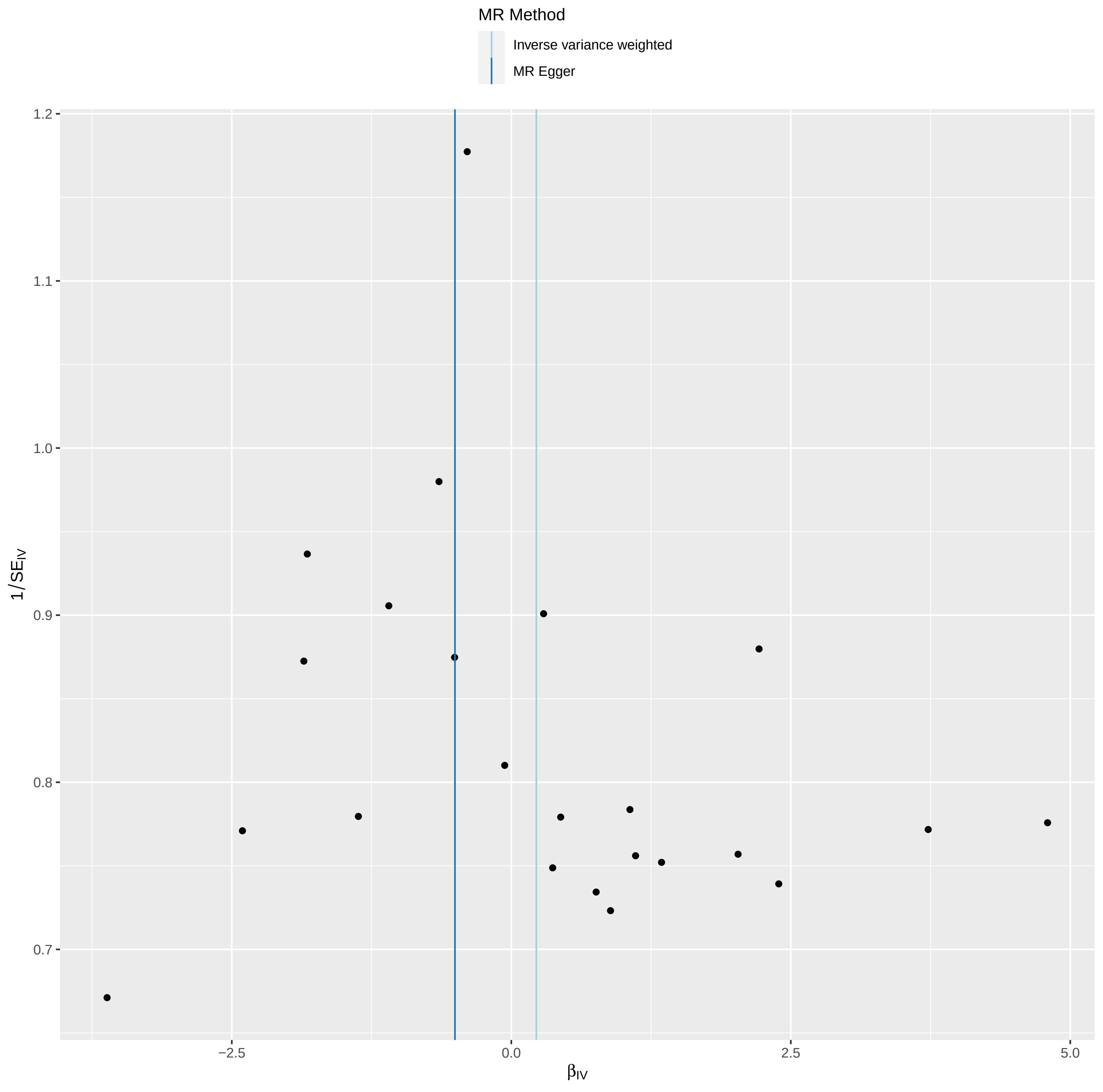

MR Method
Inverse variance weighted
MR Egger
1.2
1.1
1.0
0.9
0.8
0.7
1/SE_IV
−2.5
0.0
2.5
5.0
β_IV

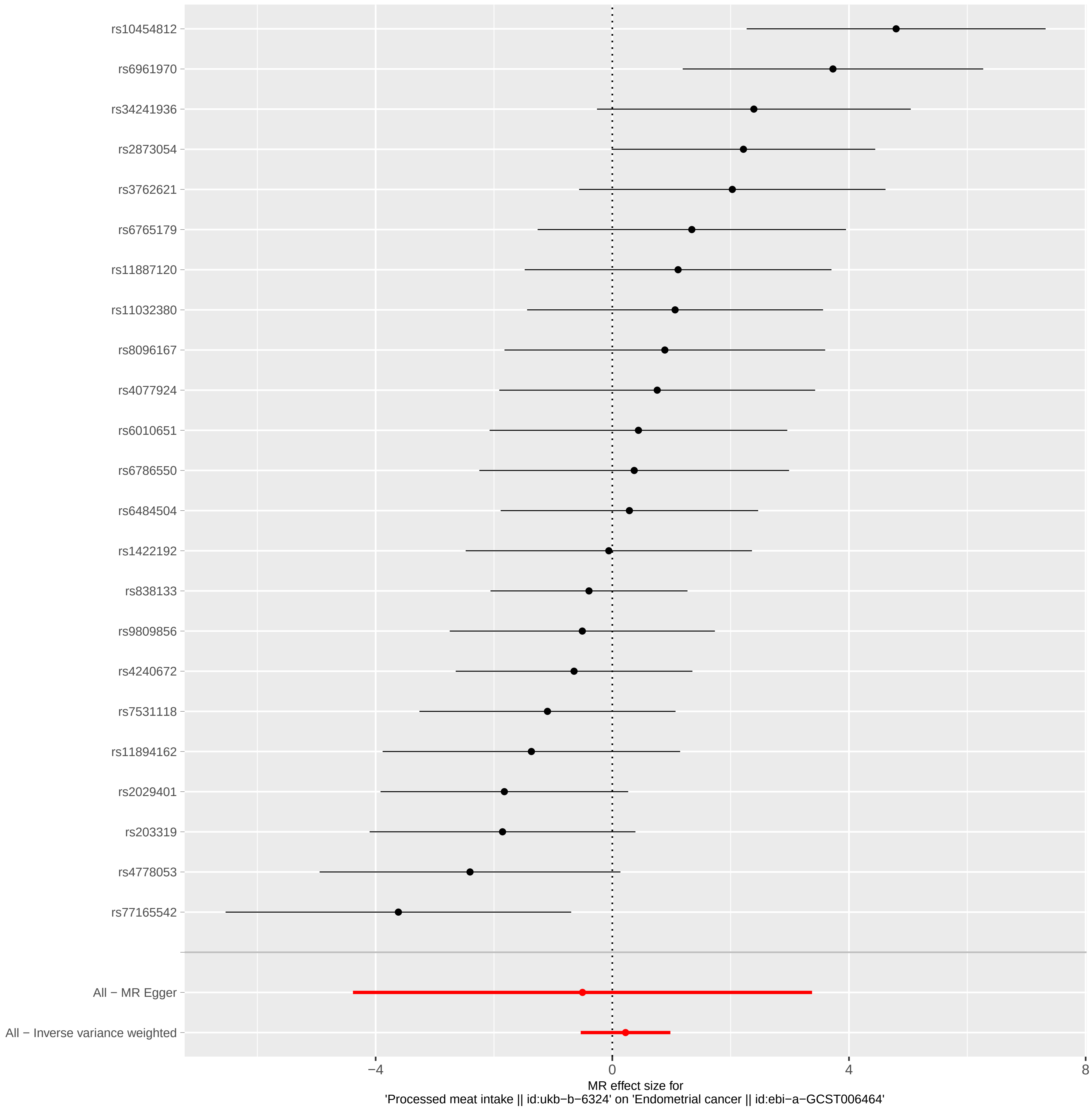

rs10454812
rs6961970
rs34241936
rs2873054
rs3762621
rs6765179
rs11887120
rs11032380
rs8096167
rs4077924
rs6010651
rs6786550
rs6484504
rs1422192
rs838133
rs9809856
rs4240672
rs7531118
rs11894162
rs2029401
rs203319
rs4778053
rs77165542
All − MR Egger
All − Inverse variance weighted
−4
0
4
8
MR effect size for
'Processed meat intake || id:ukb−b−6324' on 'Endometrial cancer || id:ebi−a−GCST006464'

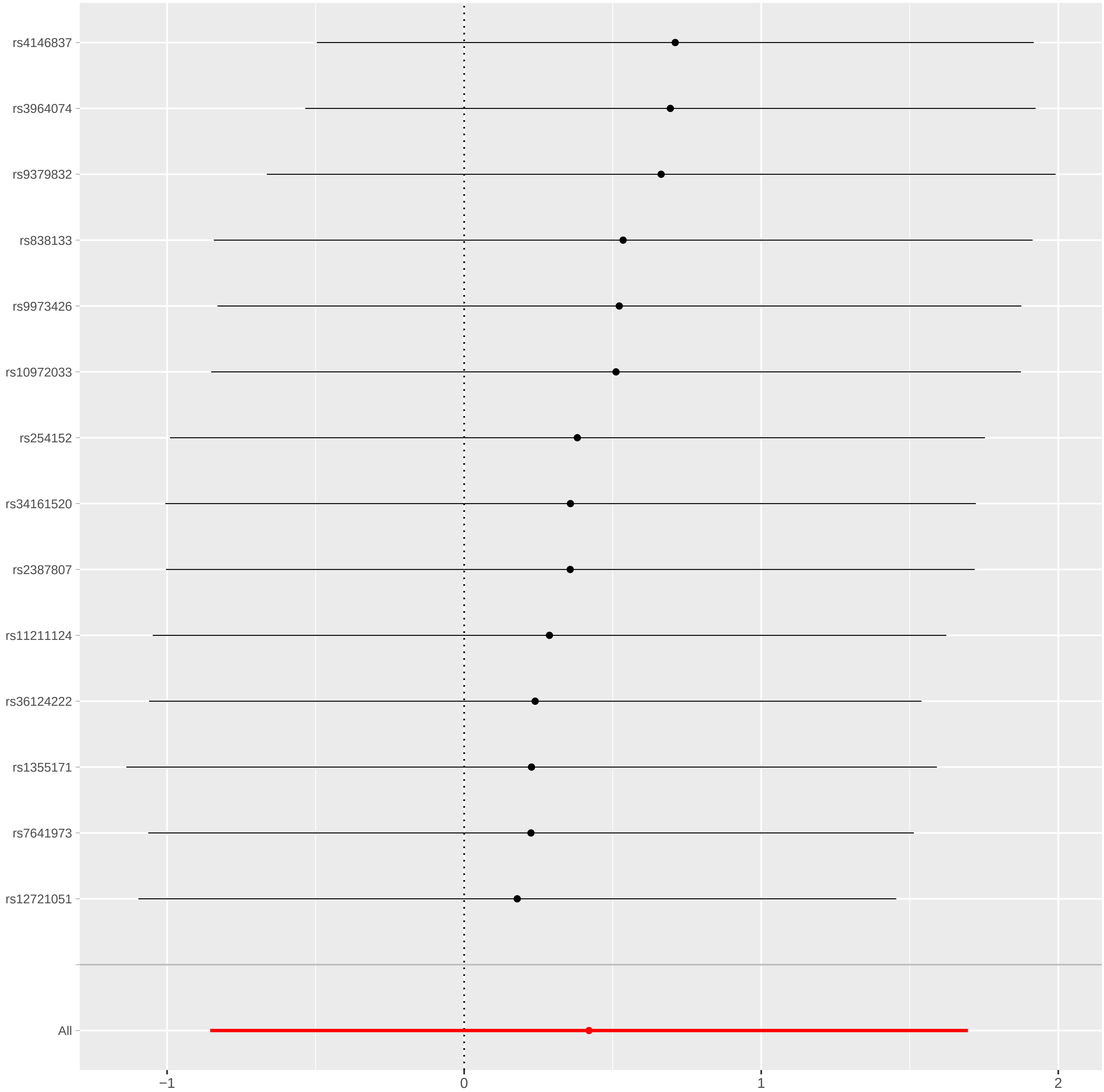

MR leave−one−out sensitivity analysis for
'Pork intake || id:ukb−b−5640' on 'Endometrial cancer || id:ebi−a−GCST006464'

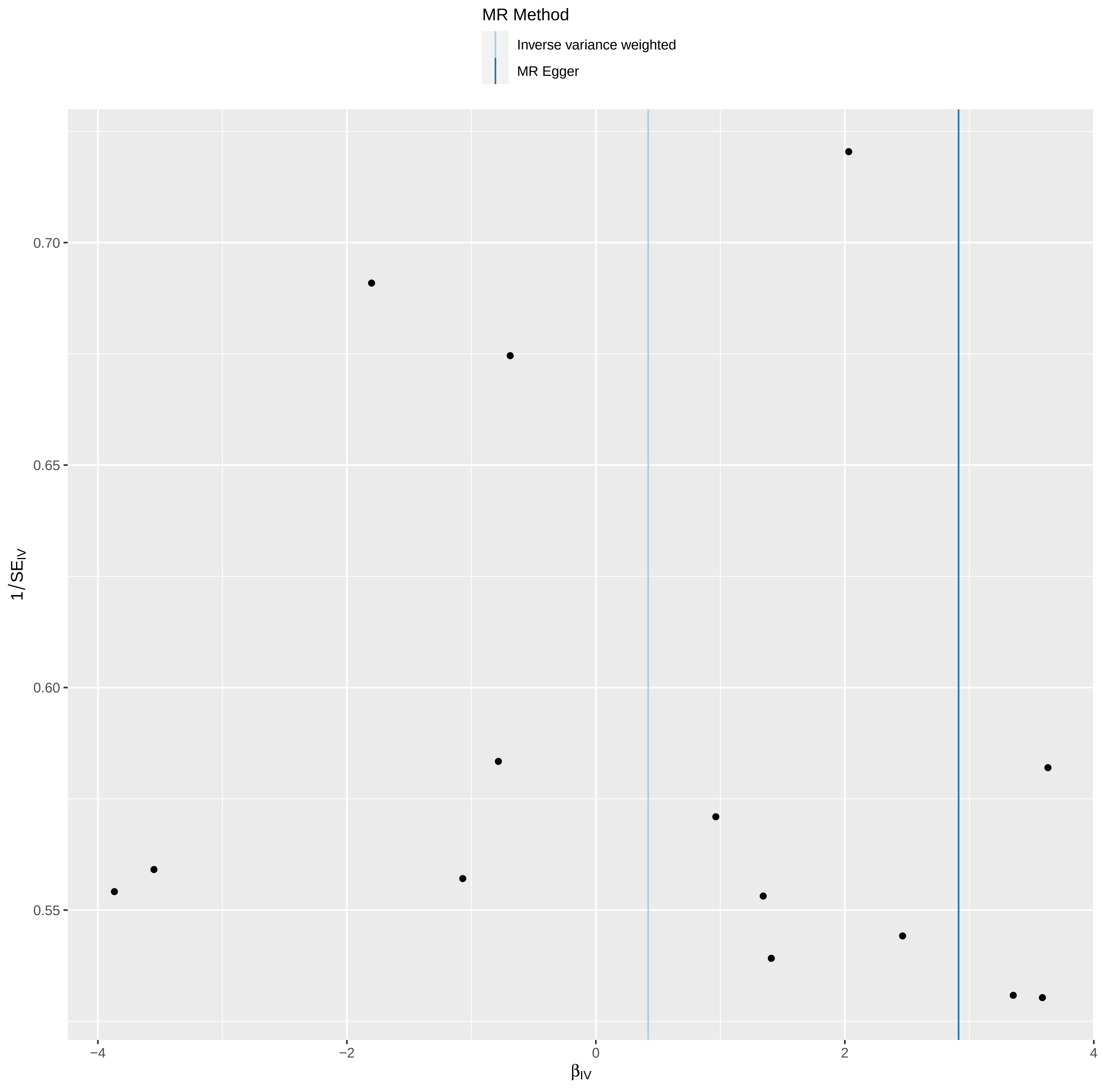
MR Method
Inverse variance weighted
MR Egger
0.70
0.65
0.60
0.55
1/SE_IV
−4
−2
0
2
4
β_IV

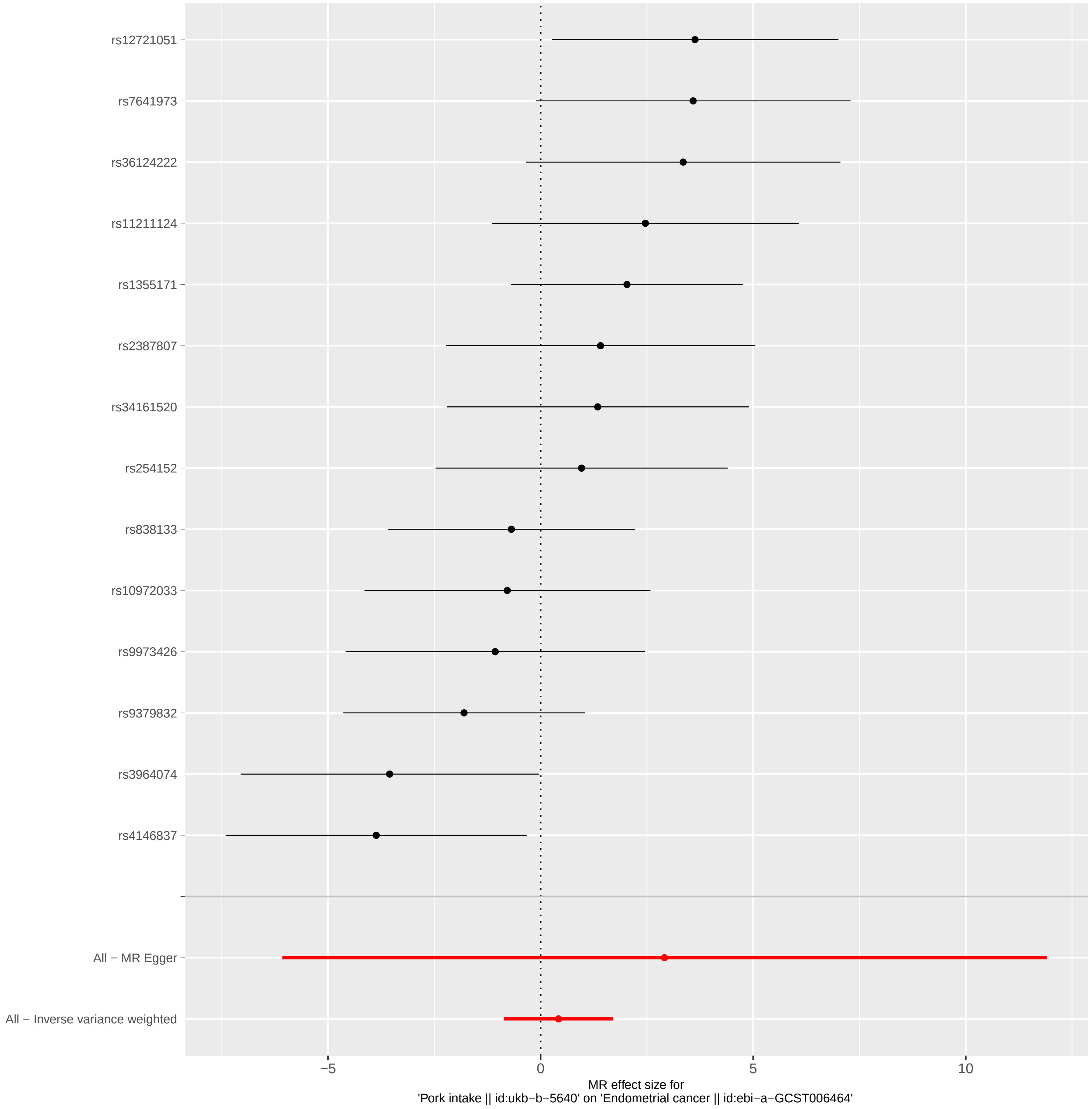

rs12721051
rs7641973
rs36124222
rs11211124
rs1355171
rs2387807
rs34161520
rs254152
rs838133
rs10972033
rs9973426
rs9379832
rs3964074
rs4146837
All − MR Egger
All − Inverse variance weighted
−5
0
5
10
MR effect size for
'Pork intake || id:ukb−b−5640' on 'Endometrial cancer || id:ebi−a−GCST006464'

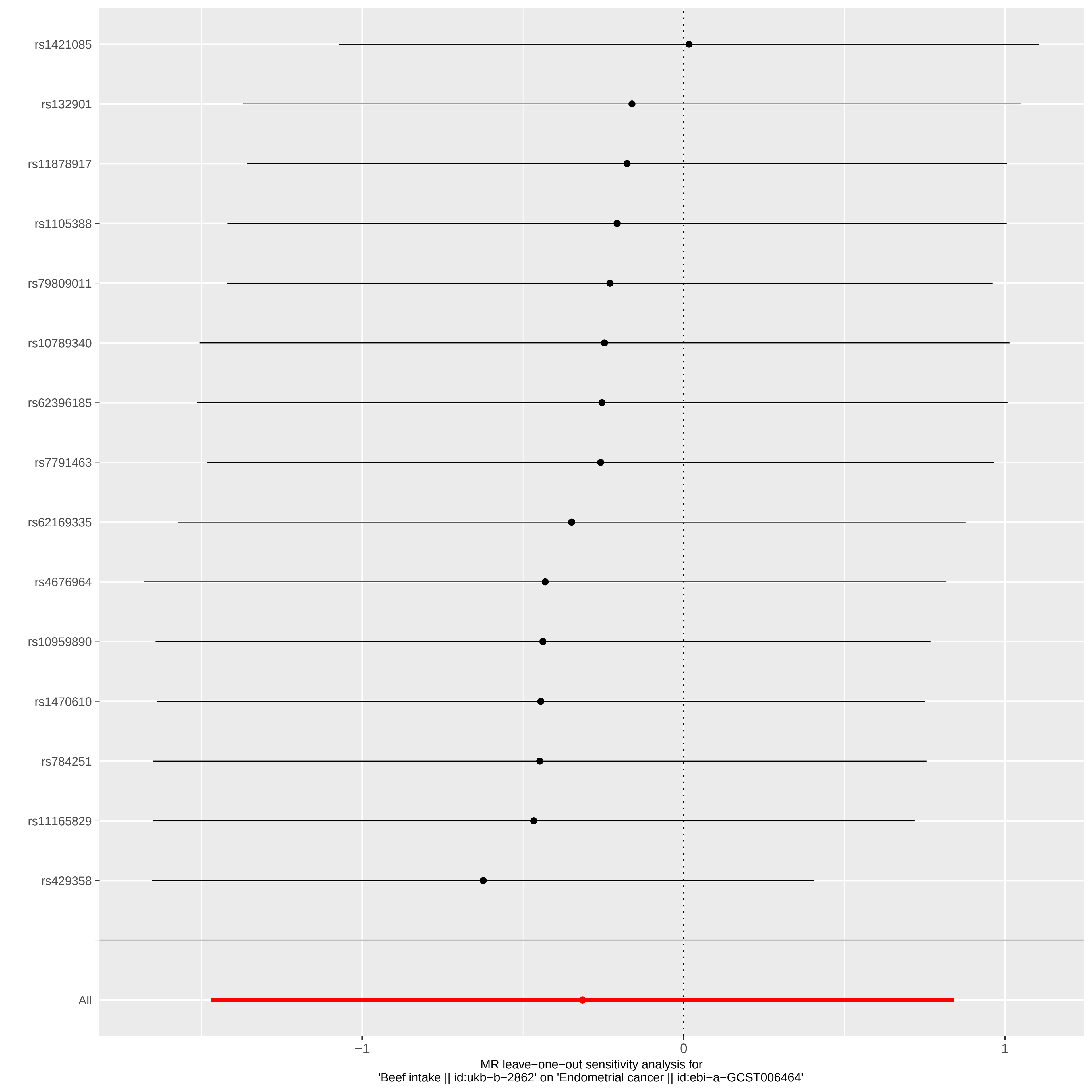
rs1421085
rs132901
rs11878917
rs1105388
rs79809011
rs10789340
rs62396185
rs7791463
rs62169335
rs4676964
rs10959890
rs1470610
rs784251
rs11165829
rs429358
All
−1
0
1
MR leave−one−out sensitivity analysis for
'Beef intake || id:ukb−b−2862' on 'Endometrial cancer || id:ebi−a−GCST006464'

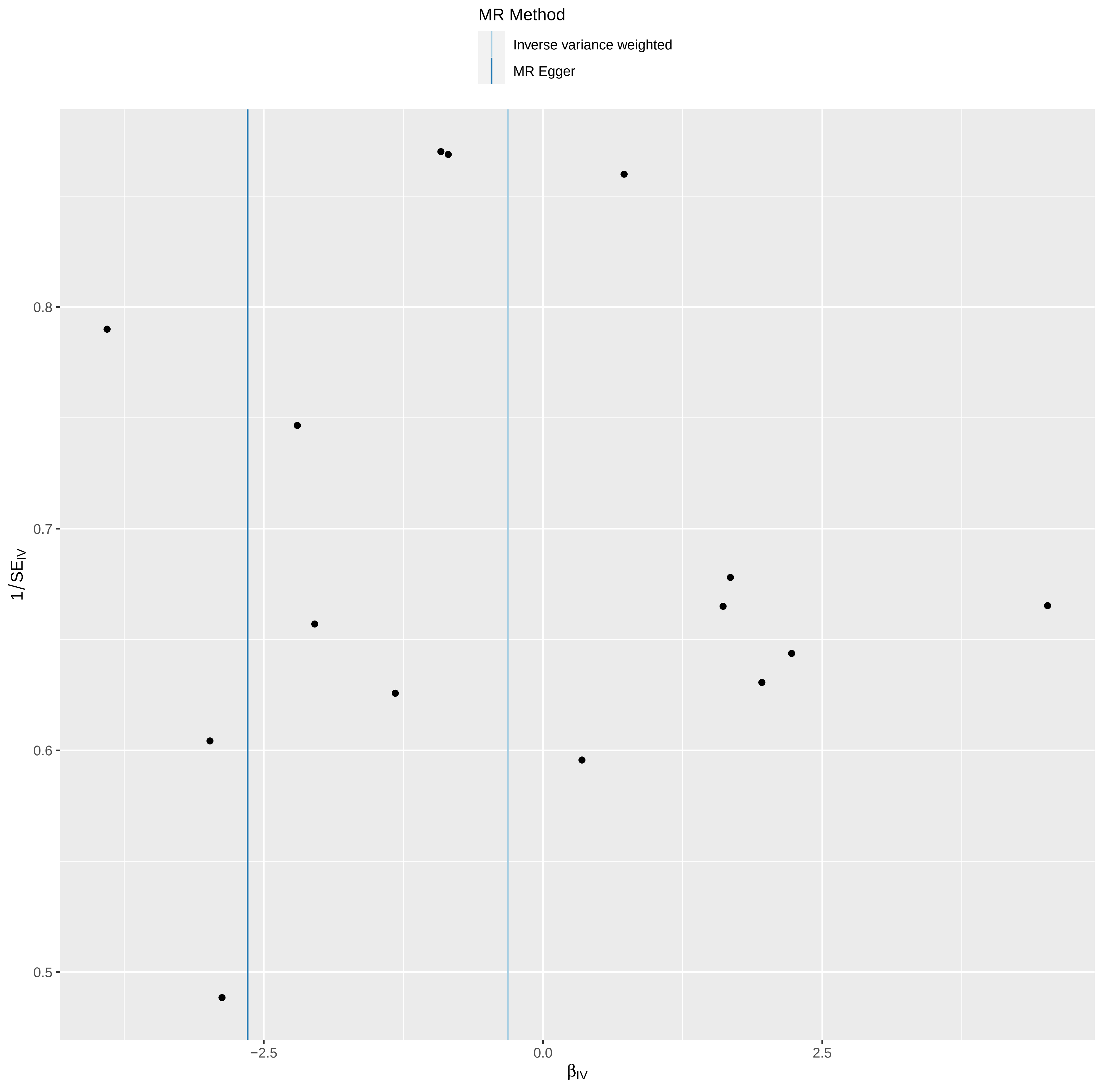

MR Method
Inverse variance weighted
MR Egger
0.8
0.7
0.6
0.5
1/SE_IV
−2.5
0.0
2.5
β_IV

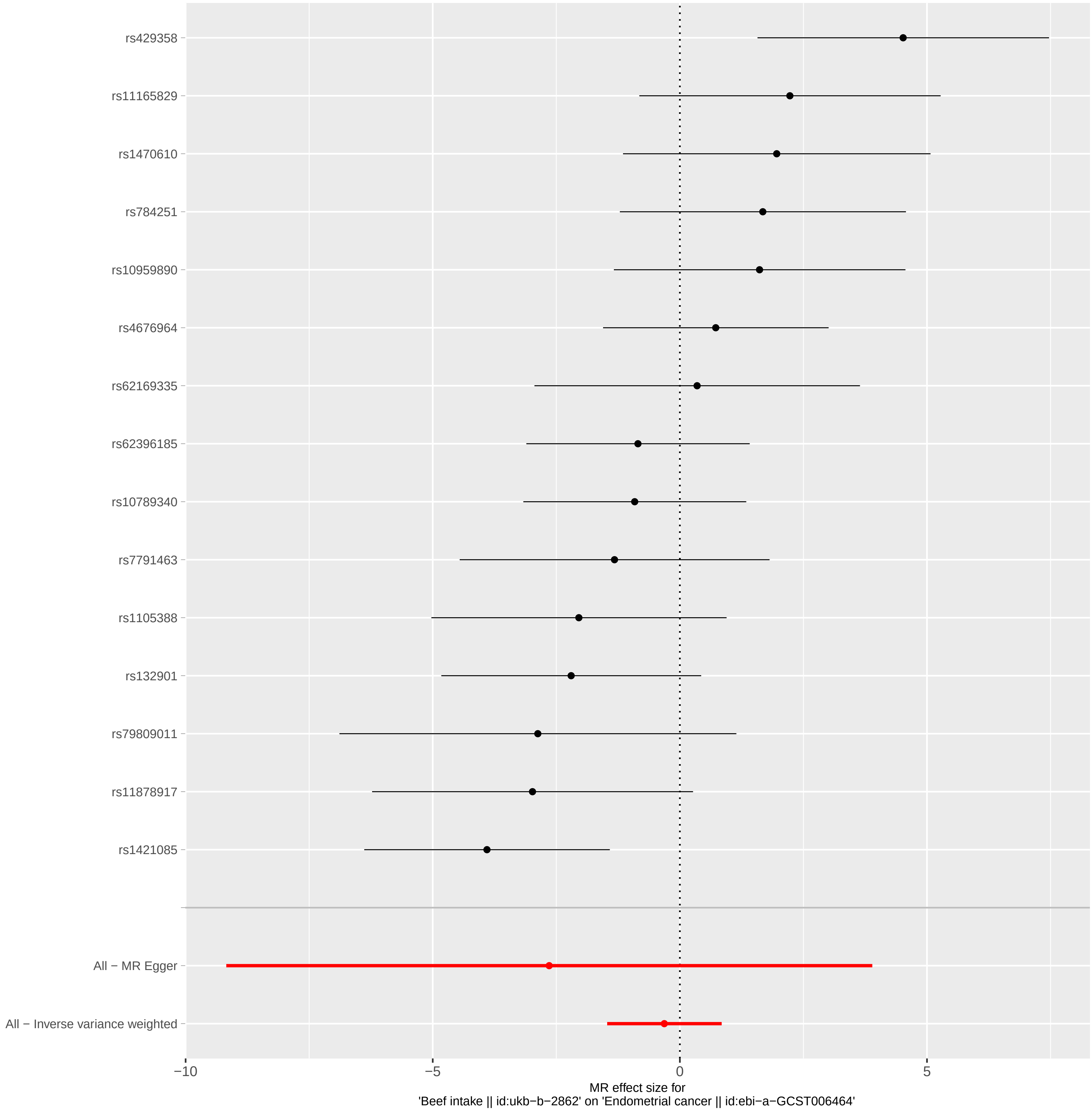
rs429358
rs11165829
rs1470610
rs784251
rs10959890
rs4676964
rs62169335
rs62396185
rs10789340
rs7791463
rs1105388
rs132901
rs79809011
rs11878917
rs1421085
All − MR Egger
All − Inverse variance weighted
−10
−5
0
5
MR effect size for
'Beef intake || id:ukb−b−2862' on 'Endometrial cancer || id:ebi−a−GCST006464'

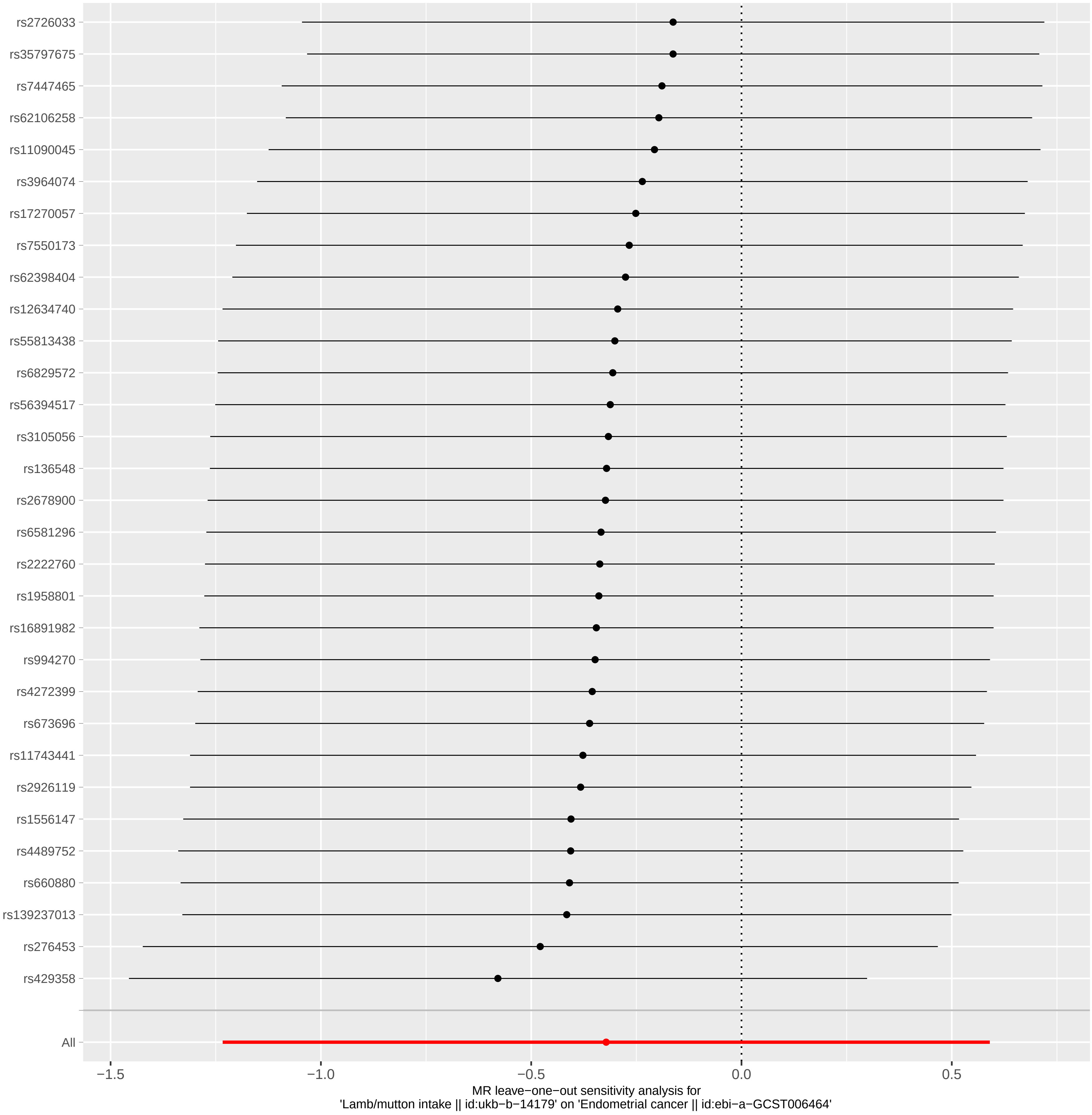

rs2726033
rs35797675
rs7447465
rs62106258
rs11090045
rs3964074
rs17270057
rs7550173
rs62398404
rs12634740
rs55813438
rs6829572
rs56394517
rs3105056
rs136548
rs2678900
rs6581296
rs2222760
rs1958801
rs16891982
rs994270
rs4272399
rs673696
rs11743441
rs2926119
rs1556147
rs4489752
rs660880
rs139237013
rs276453
rs429358
All
−1.5
−1.0
−0.5
0.0
0.5
MR leave−one−out sensitivity analysis for
'Lamb/mutton intake || id:ukb−b−14179' on 'Endometrial cancer || id:ebi−a−GCST006464'

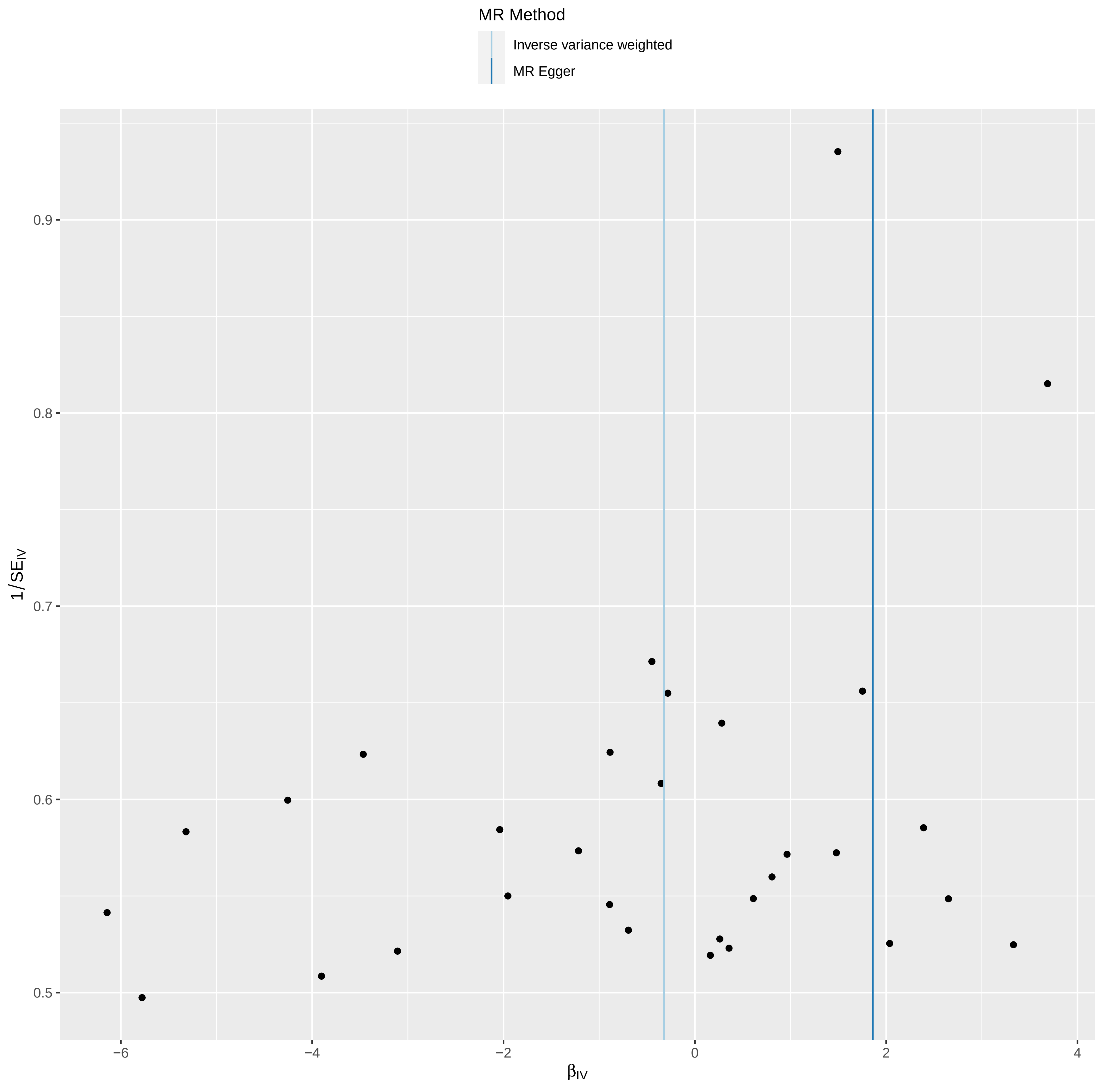
MR Method
Inverse variance weighted
MR Egger
0.9
0.8
0.7
0.6
0.5
1/SE_IV
−6
−4
−2
0
2
4
β_IV

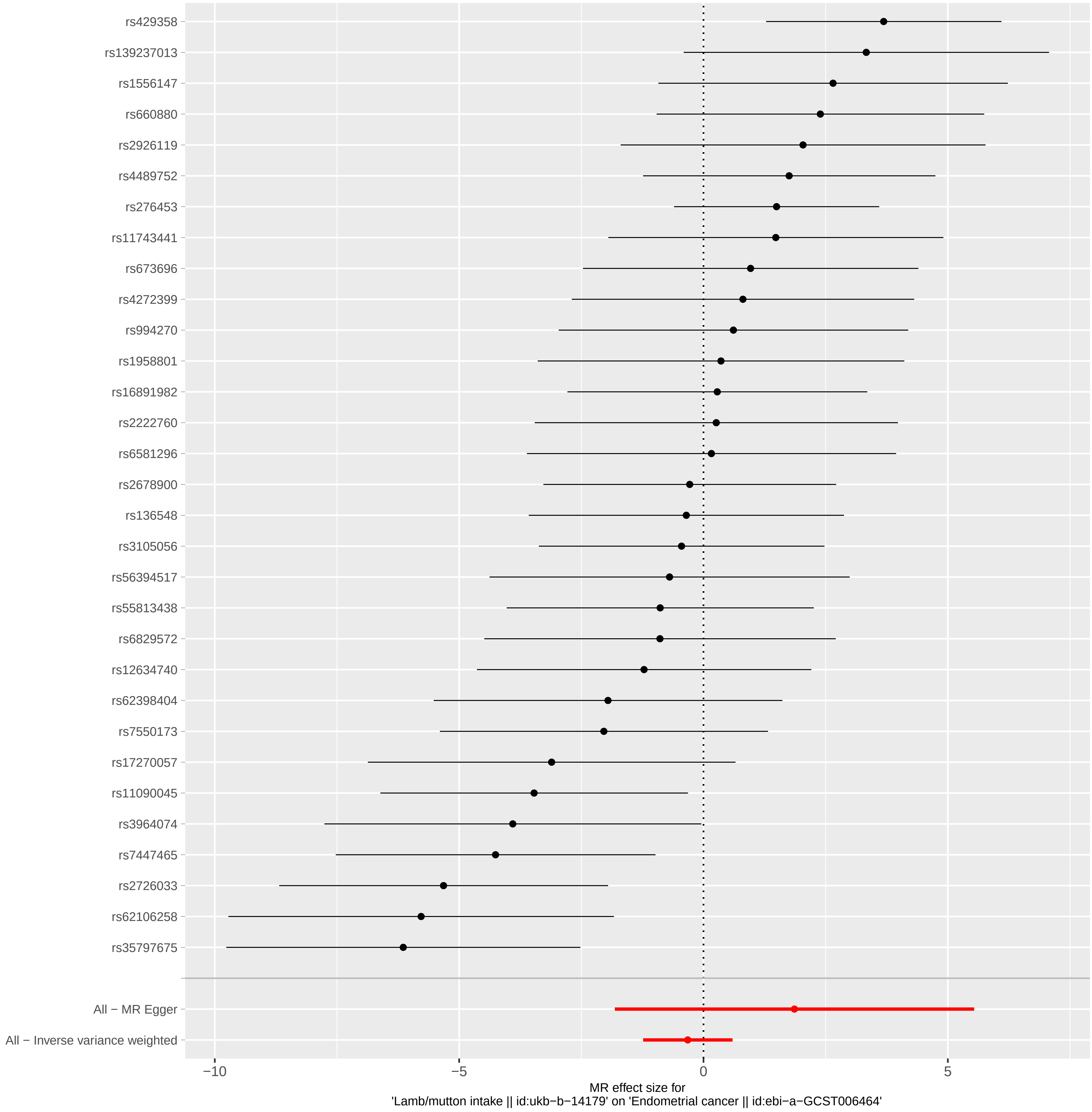
rs429358
rs139237013
rs1556147
rs660880
rs2926119
rs4489752
rs276453
rs11743441
rs673696
rs4272399
rs994270
rs1958801
rs16891982
rs2222760
rs6581296
rs2678900
rs136548
rs3105056
rs56394517
rs55813438
rs6829572
rs12634740
rs62398404
rs7550173
rs17270057
rs11090045
rs3964074
rs7447465
rs2726033
rs62106258
rs35797675
All − MR Egger
All − Inverse variance weighted
−10
−5
0
5
MR effect size for
'Lamb/mutton intake || id:ukb−b−14179' on 'Endometrial cancer || id:ebi−a−GCST006464'

Figure S4. Leave-one-out analysis, funnel plot and MR effect size for processed meat, pork, beef and mutton intake on breast carcinoma.

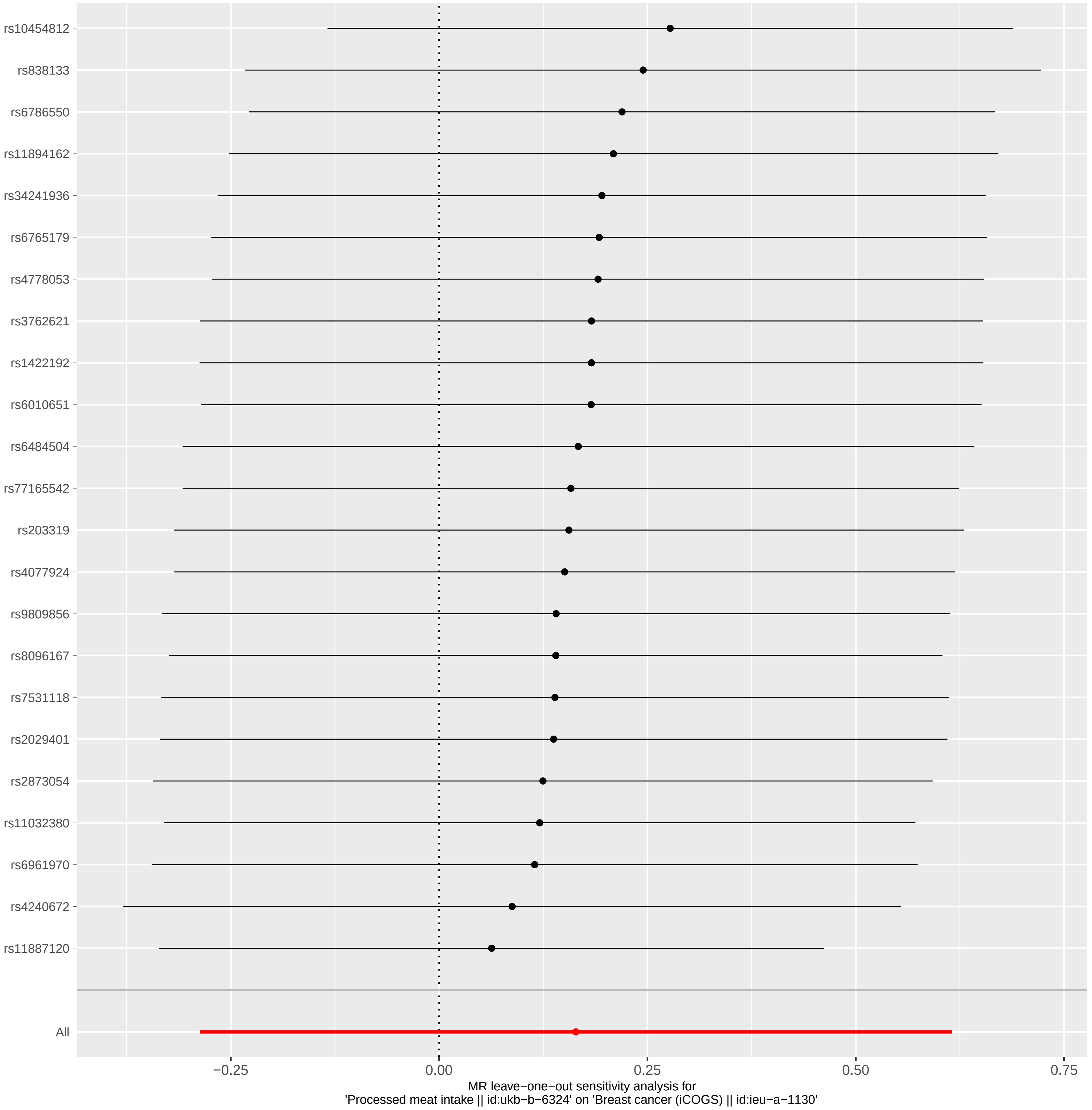

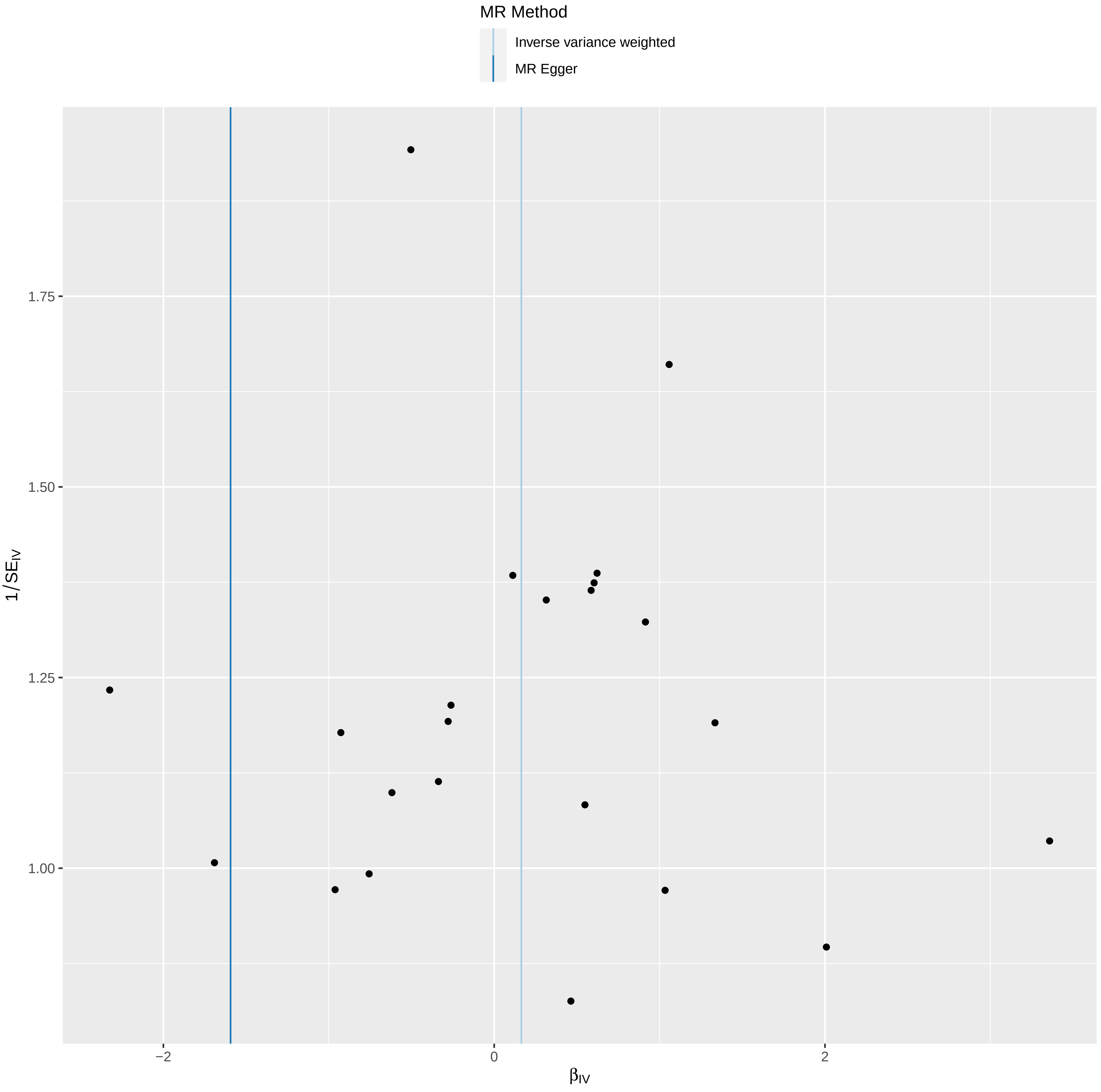
MR Method
Inverse variance weighted
MR Egger
1.75
1.50
1.25
1.00
1/SE$_{IV}$
−2
0
2
β$_{IV}$

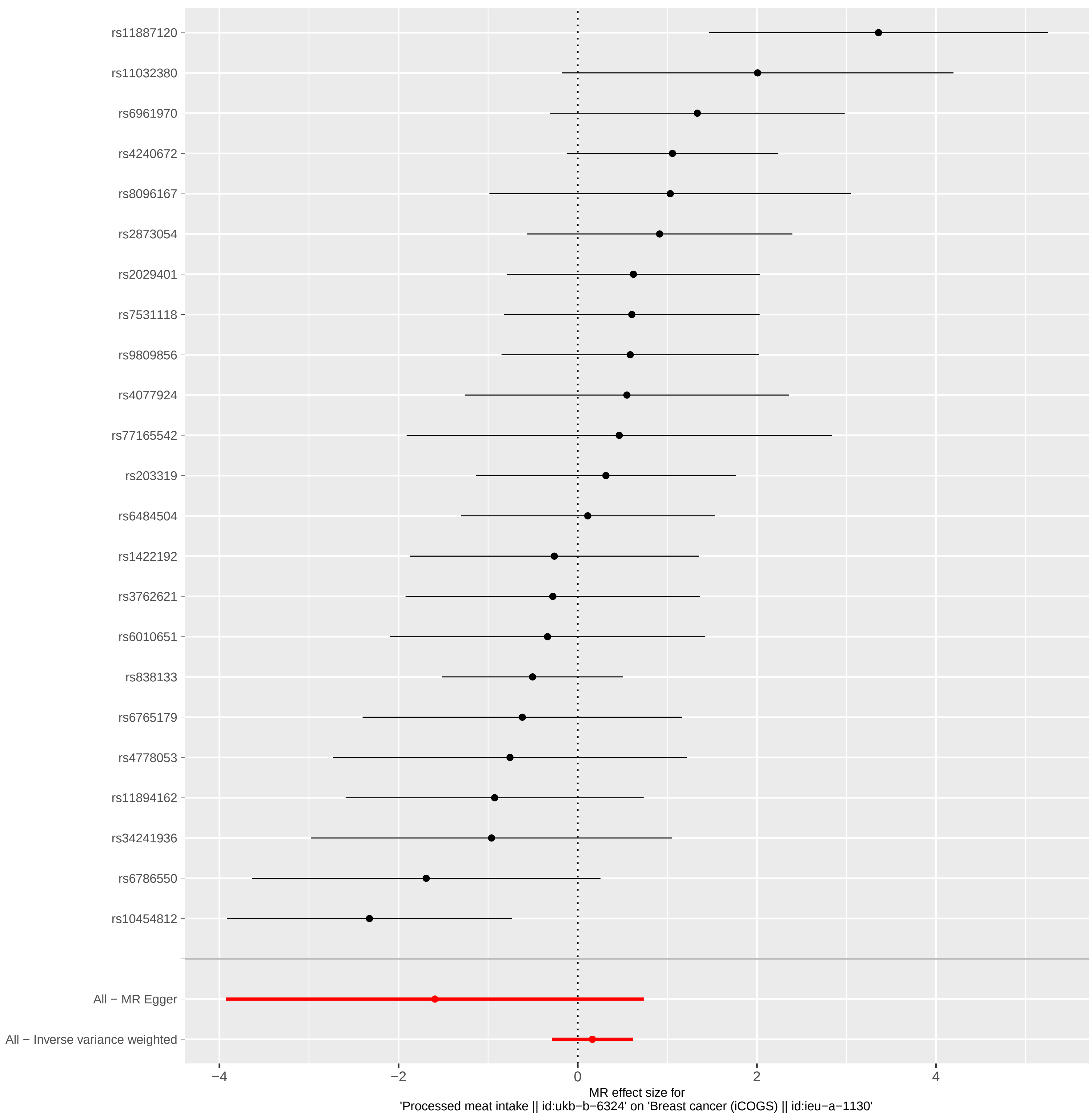
rs11887120
rs11032380
rs6961970
rs4240672
rs8096167
rs2873054
rs2029401
rs7531118
rs9809856
rs4077924
rs77165542
rs203319
rs6484504
rs1422192
rs3762621
rs6010651
rs838133
rs6765179
rs4778053
rs11894162
rs34241936
rs6786550
rs10454812
All − MR Egger
All − Inverse variance weighted
−4
−2
0
2
4
MR effect size for
'Processed meat intake || id:ukb−b−6324' on 'Breast cancer (iCOGS) || id:ieu−a−1130'

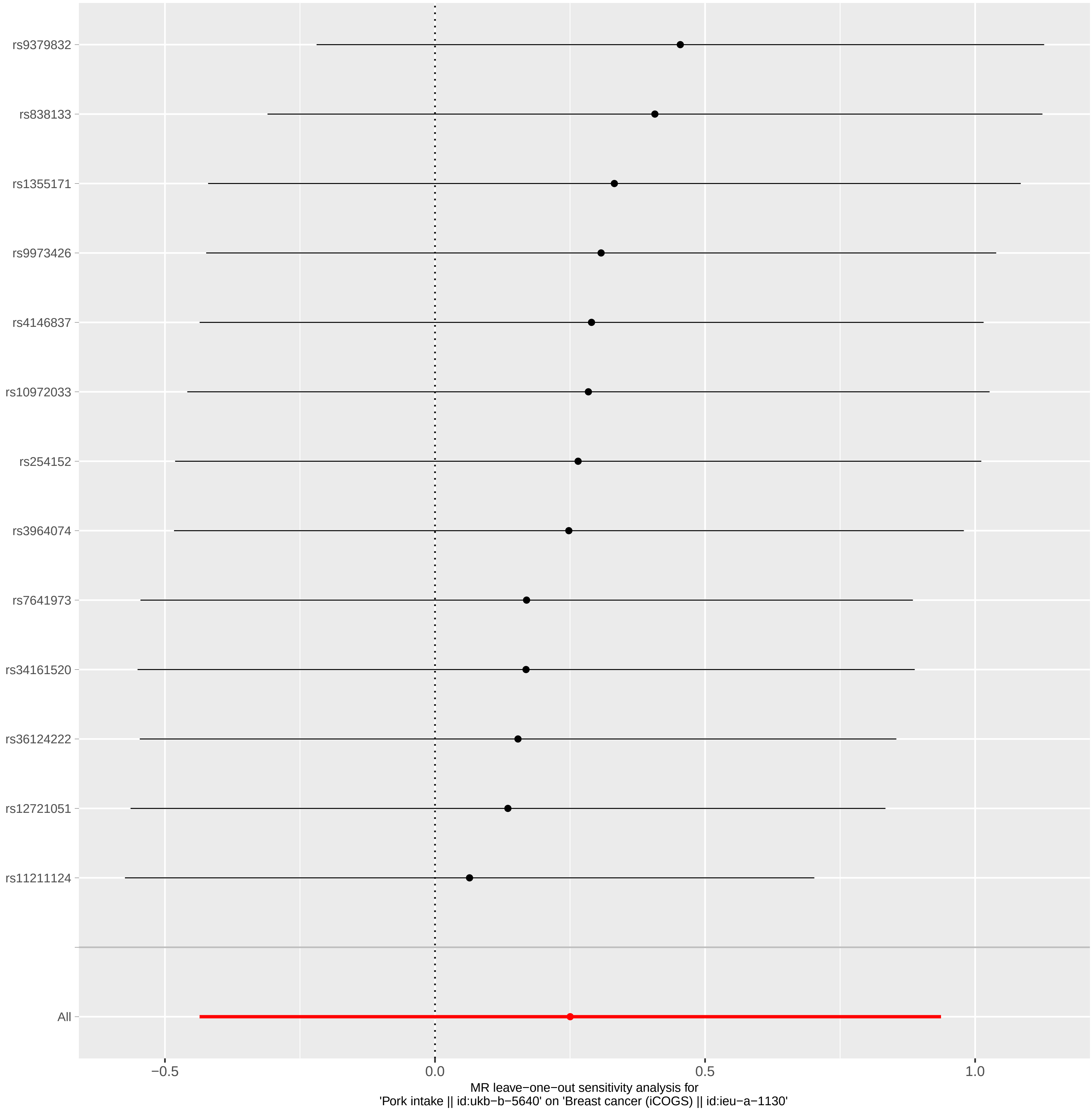
rs9379832
rs838133
rs1355171
rs9973426
rs4146837
rs10972033
rs254152
rs3964074
rs7641973
rs34161520
rs36124222
rs12721051
rs11211124
All
−0.5
0.0
0.5
1.0
MR leave−one−out sensitivity analysis for
'Pork intake || id:ukb−b−5640' on 'Breast cancer (iCOGS) || id:ieu−a−1130'

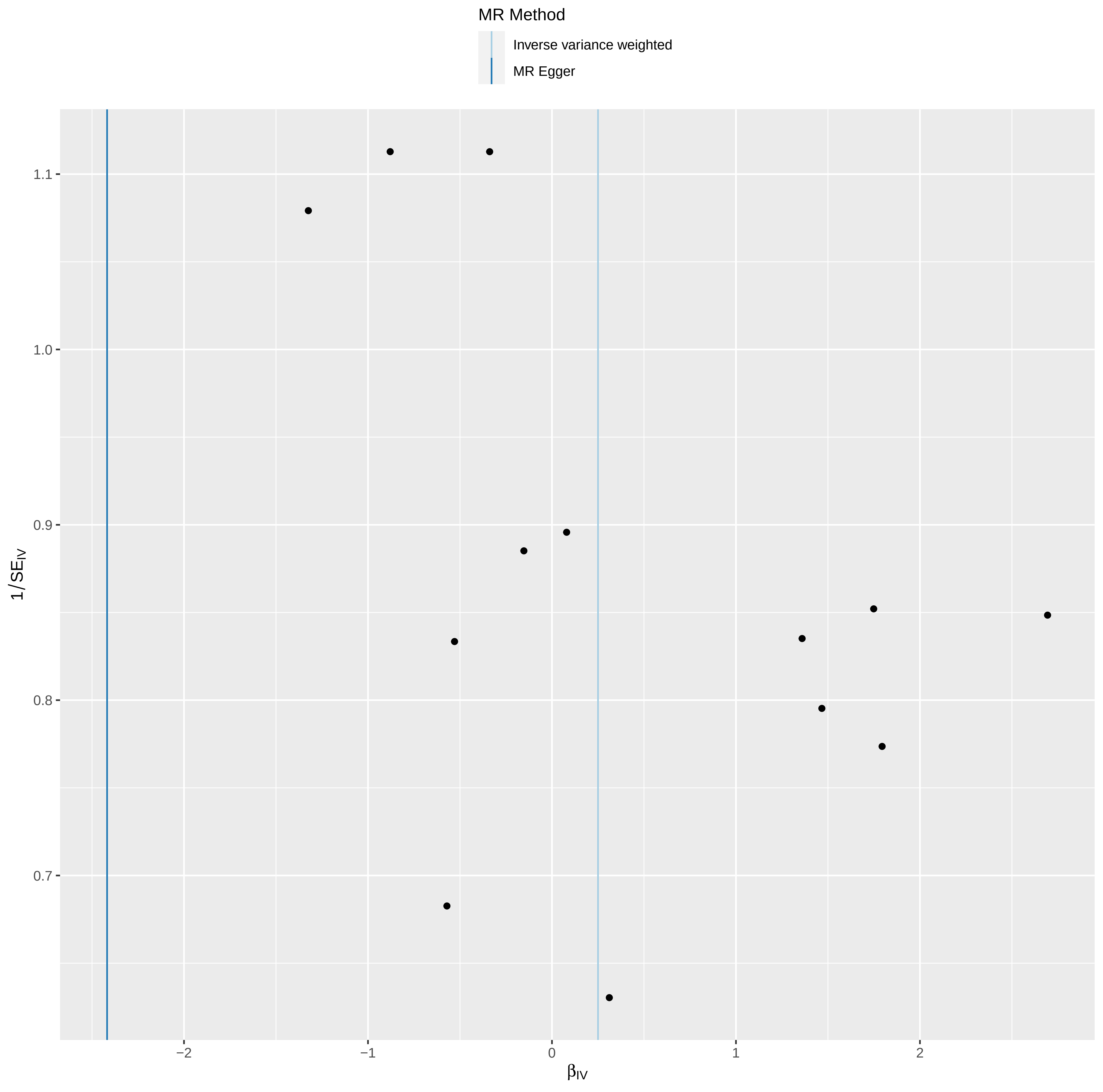

MR Method
Inverse variance weighted
MR Egger
1.1
1.0
0.9
0.8
0.7
1/SE_IV
−2
−1
0
1
2
β_IV

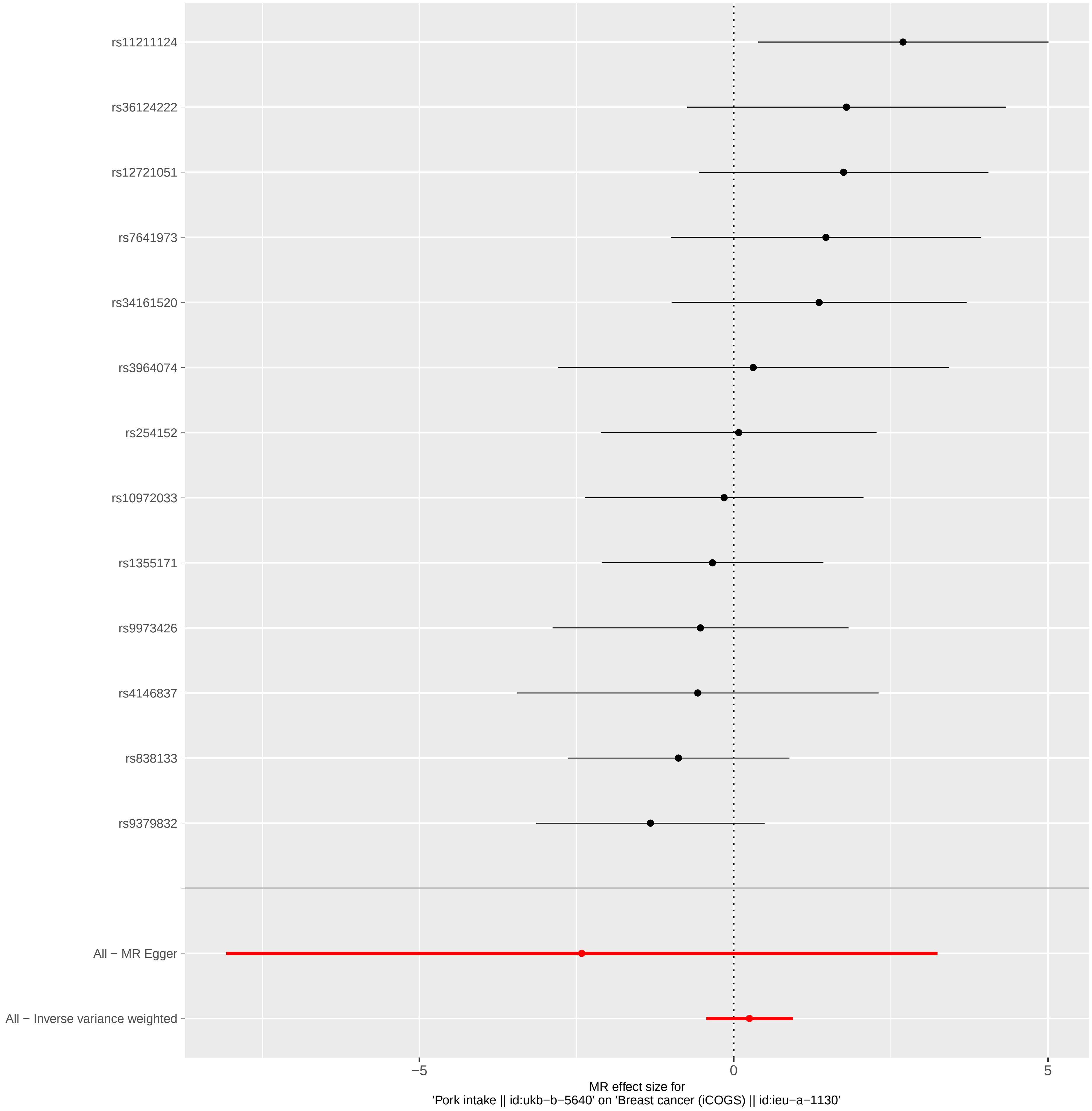

rs11211124
rs36124222
rs12721051
rs7641973
rs34161520
rs3964074
rs254152
rs10972033
rs1355171
rs9973426
rs4146837
rs838133
rs9379832
All − MR Egger
All − Inverse variance weighted
−5
0
5
MR effect size for
'Pork intake || id:ukb−b−5640' on 'Breast cancer (iCOGS) || id:ieu−a−1130'

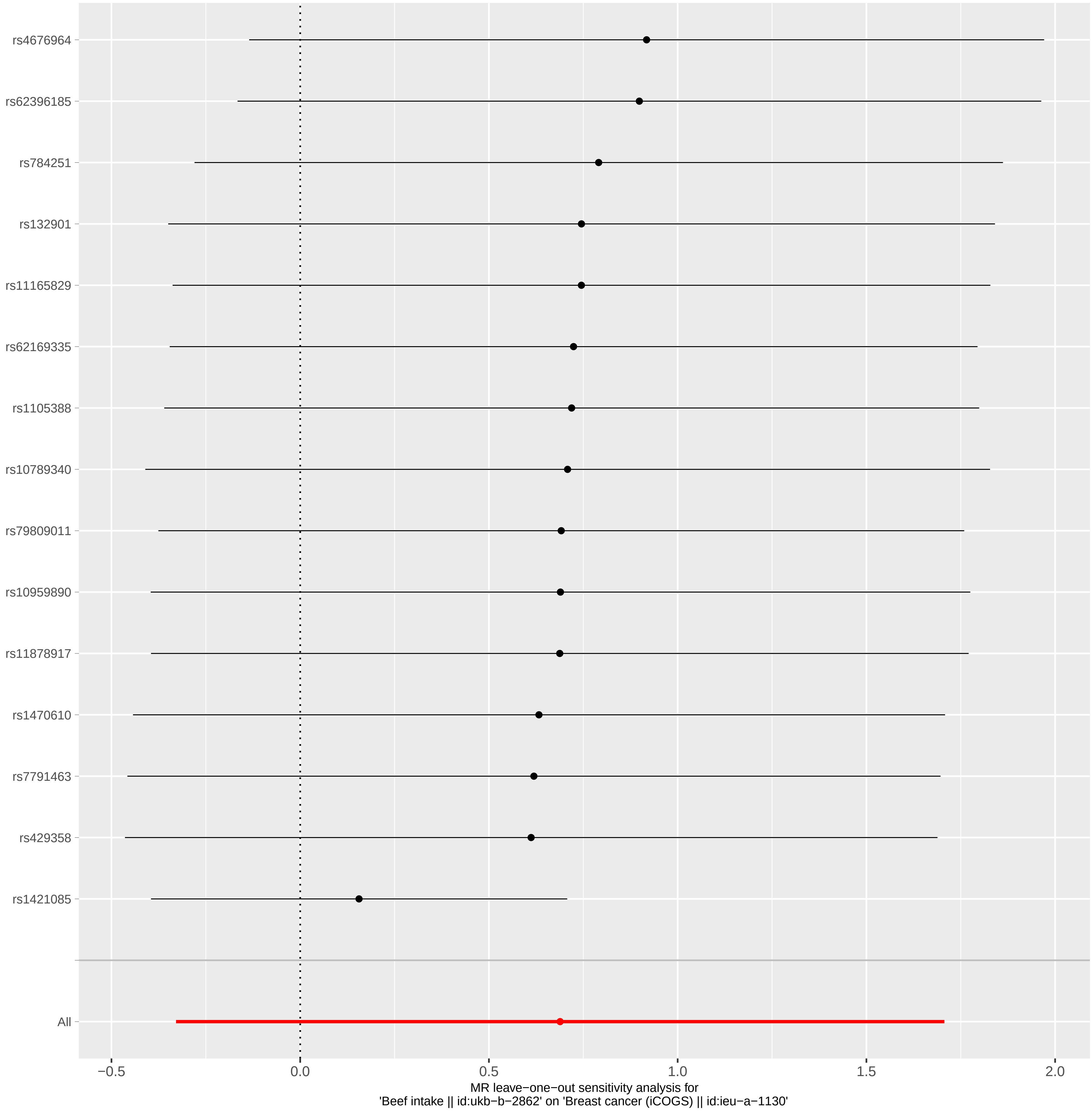
rs4676964
rs62396185
rs784251
rs132901
rs11165829
rs62169335
rs1105388
rs10789340
rs79809011
rs10959890
rs11878917
rs1470610
rs7791463
rs429358
rs1421085
All
−0.5
0.0
0.5
1.0
1.5
2.0
MR leave−one−out sensitivity analysis for
'Beef intake || id:ukb−b−2862' on 'Breast cancer (iCOGS) || id:ieu−a−1130'

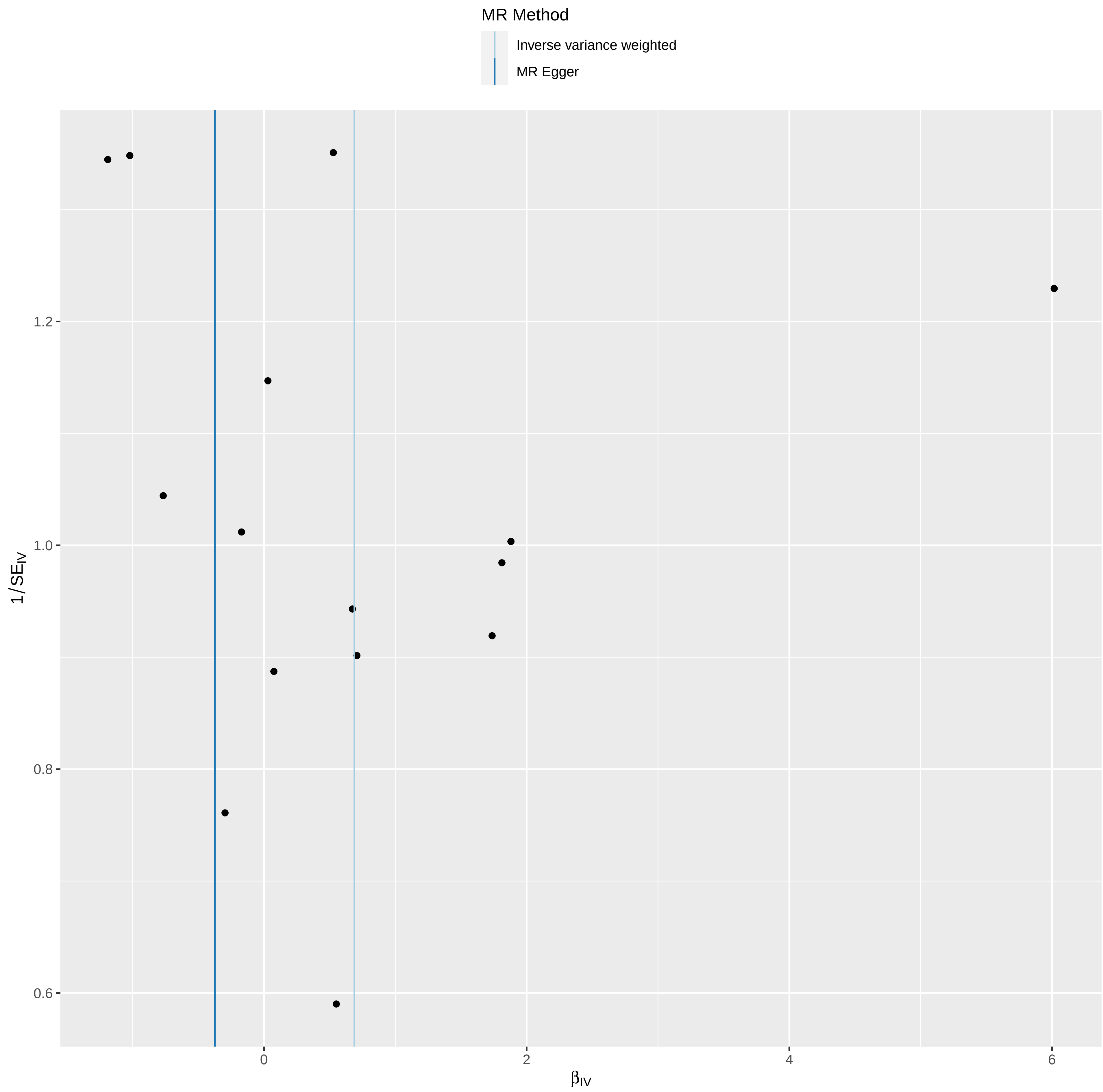

MR Method
Inverse variance weighted
MR Egger
1.2
1.0
0.8
0.6
$1/SE_{IV}$
0
2
4
6
β_{IV}

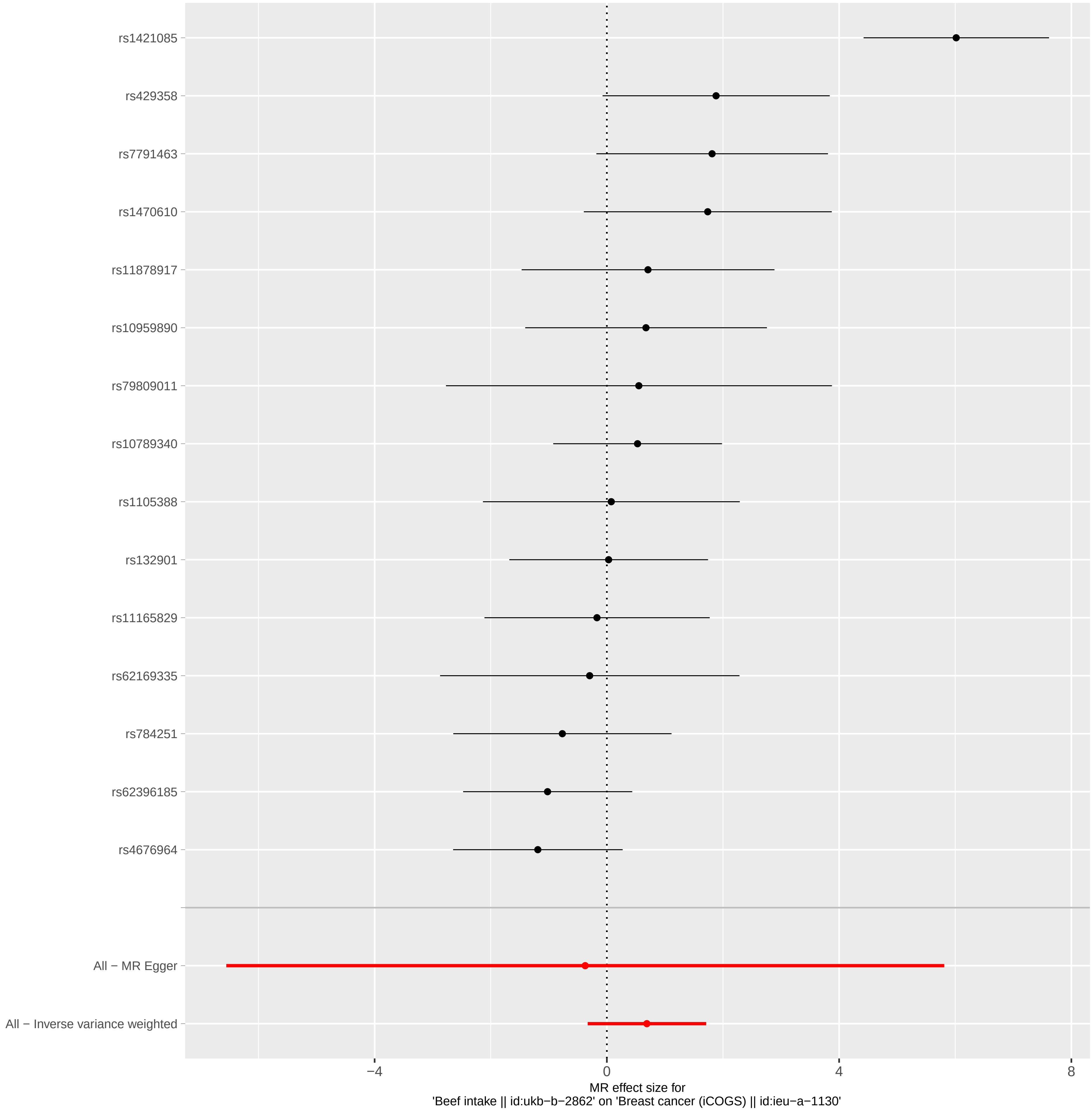
rs1421085
rs429358
rs7791463
rs1470610
rs11878917
rs10959890
rs79809011
rs10789340
rs1105388
rs132901
rs11165829
rs62169335
rs784251
rs62396185
rs4676964
All − MR Egger
All − Inverse variance weighted
−4
0
4
8
MR effect size for
'Beef intake || id:ukb−b−2862' on 'Breast cancer (iCOGS) || id:ieu−a−1130'

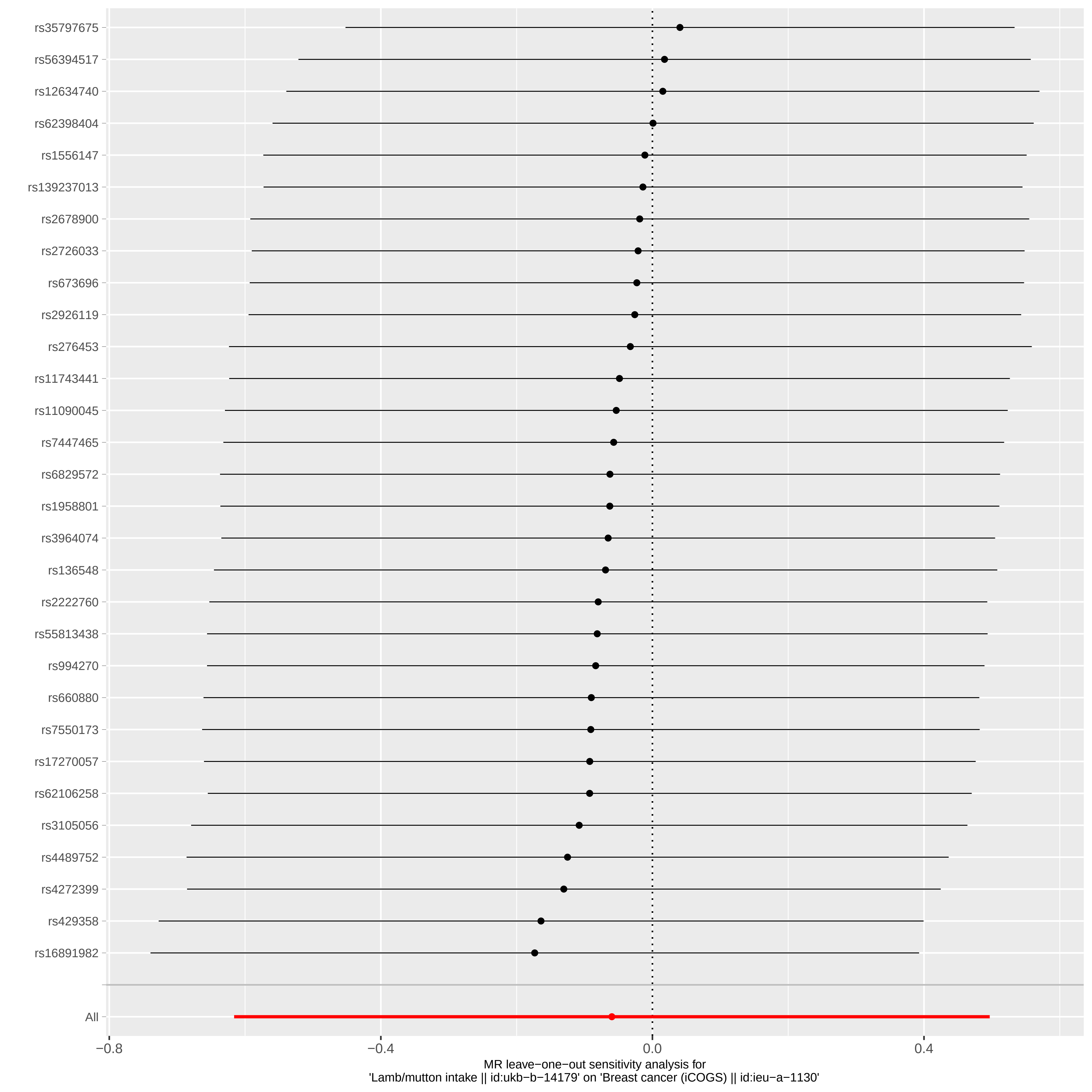
rs35797675
rs56394517
rs12634740
rs62398404
rs1556147
rs139237013
rs2678900
rs2726033
rs673696
rs2926119
rs276453
rs11743441
rs11090045
rs7447465
rs6829572
rs1958801
rs3964074
rs136548
rs2222760
rs55813438
rs994270
rs660880
rs7550173
rs17270057
rs62106258
rs3105056
rs4489752
rs4272399
rs429358
rs16891982
All
−0.8
−0.4
0.0
0.4
MR leave−one−out sensitivity analysis for
'Lamb/mutton intake || id:ukb−b−14179' on 'Breast cancer (iCOGS) || id:ieu−a−1130'

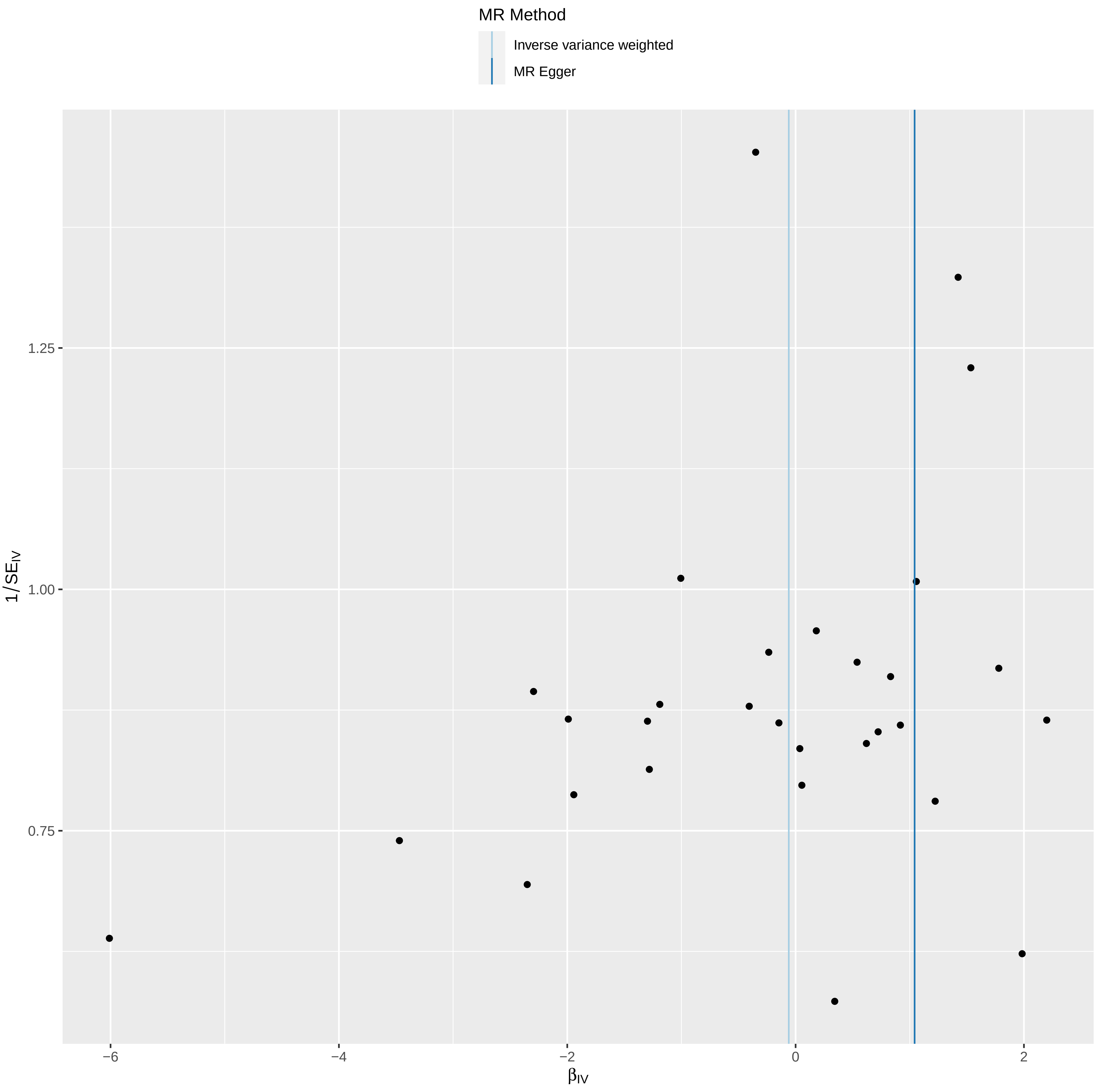
MR Method
Inverse variance weighted
MR Egger
1.25
1.00
0.75
1/SE_IV
−6
−4
−2
0
2
β_IV

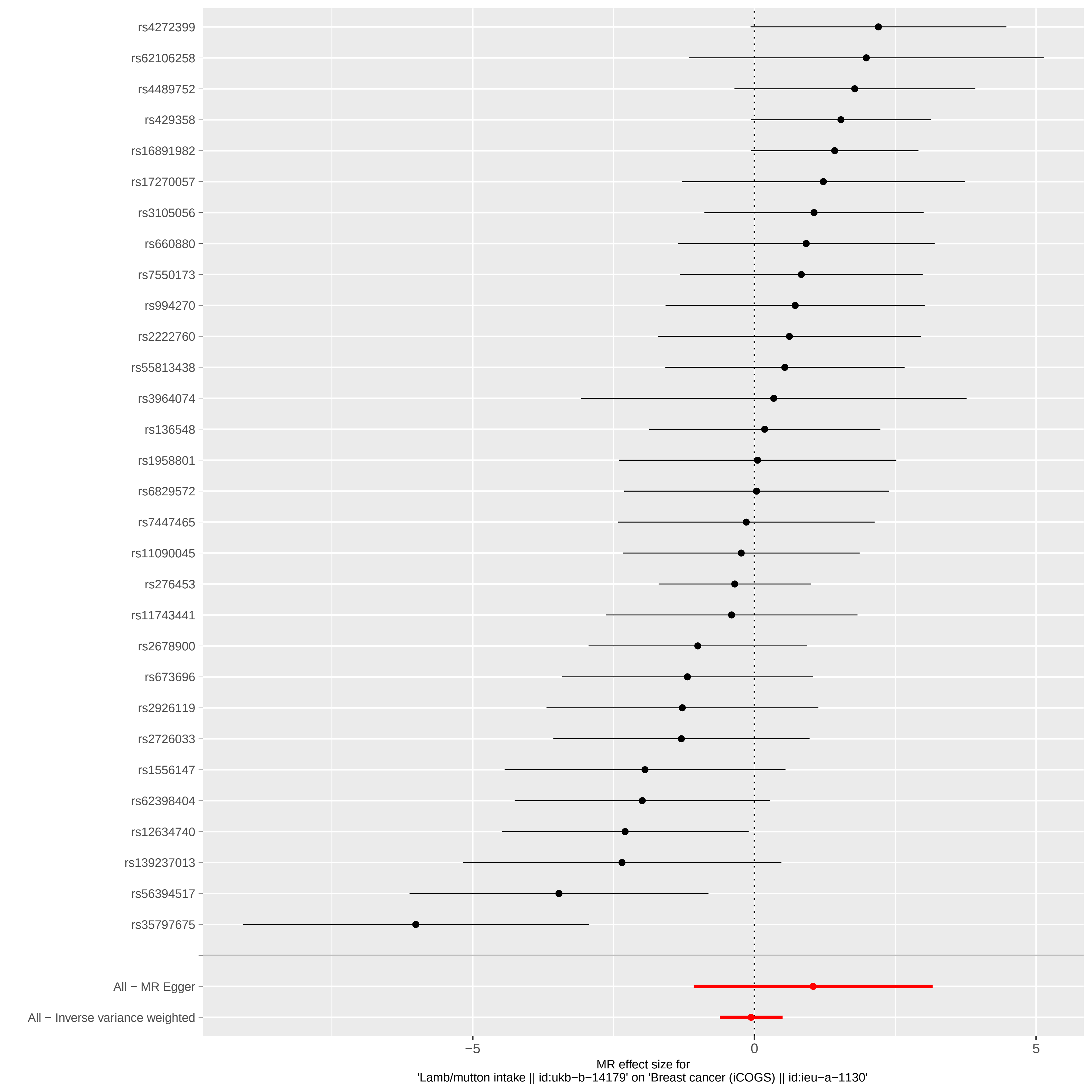
rs4272399
rs62106258
rs4489752
rs429358
rs16891982
rs17270057
rs3105056
rs660880
rs7550173
rs994270
rs2222760
rs55813438
rs3964074
rs136548
rs1958801
rs6829572
rs7447465
rs11090045
rs276453
rs11743441
rs2678900
rs673696
rs2926119
rs2726033
rs1556147
rs62398404
rs12634740
rs139237013
rs56394517
rs35797675
All − MR Egger
All − Inverse variance weighted
−5
0
5
MR effect size for
'Lamb/mutton intake || id:ukb−b−14179' on 'Breast cancer (iCOGS) || id:ieu−a−1130'

Figure S5. Leave-one-out analysis, funnel plot and MR effect size for processed meat, pork, beef and mutton intake on renal cell carcinoma.

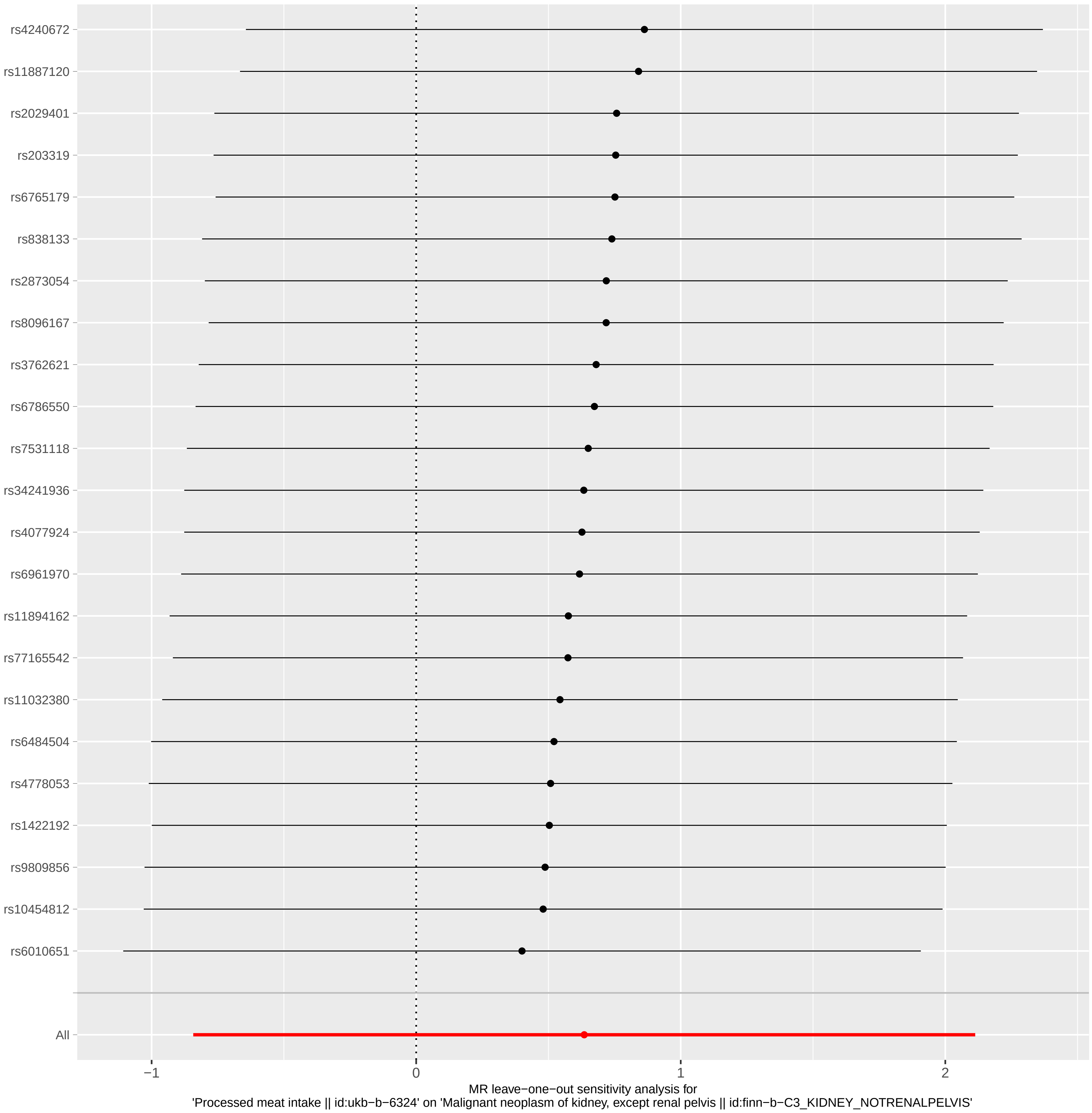

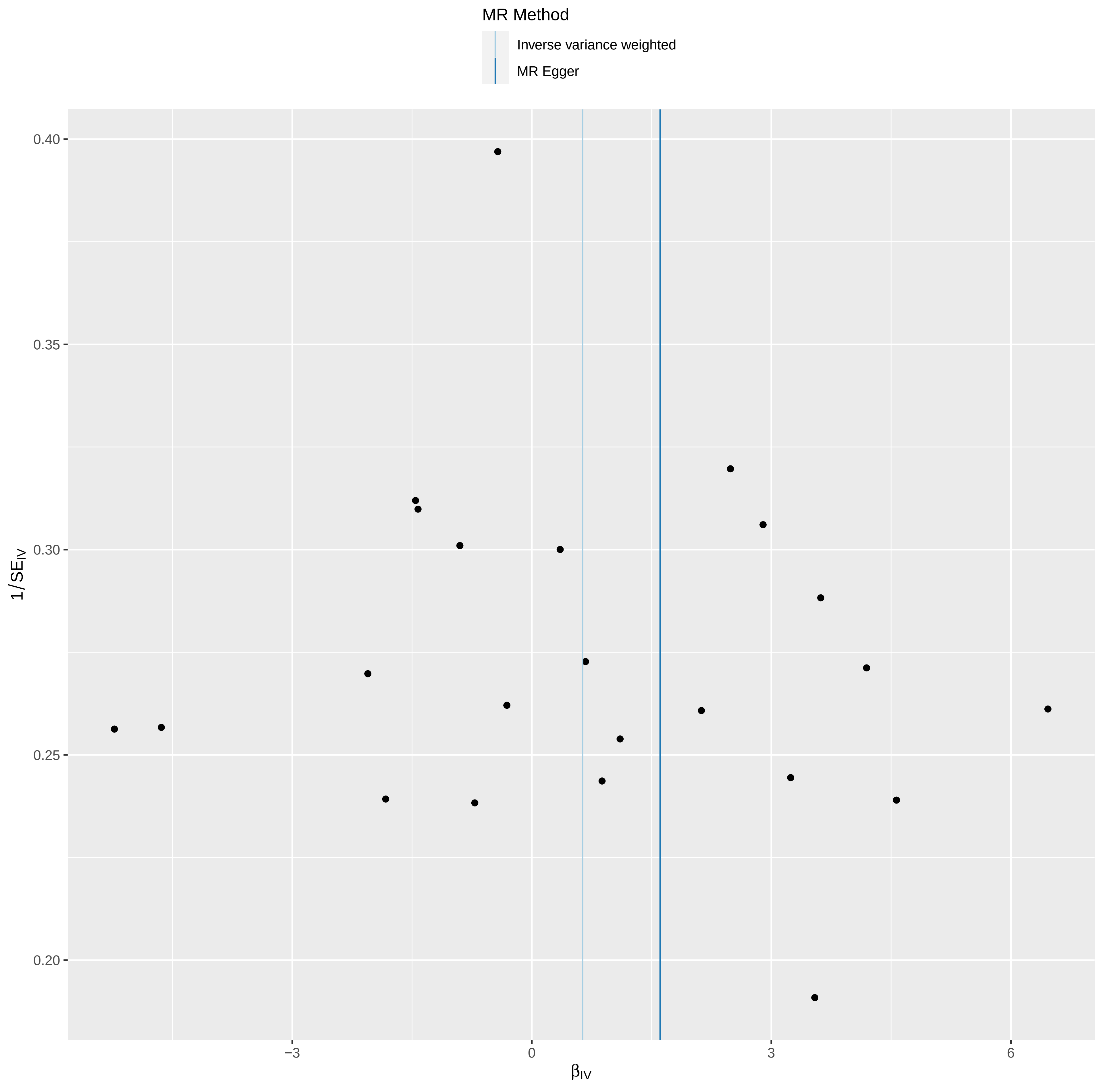
MR Method
Inverse variance weighted
MR Egger
0.40
0.35
0.30
0.25
0.20
1/SE_IV
−3
0
3
6
β_IV

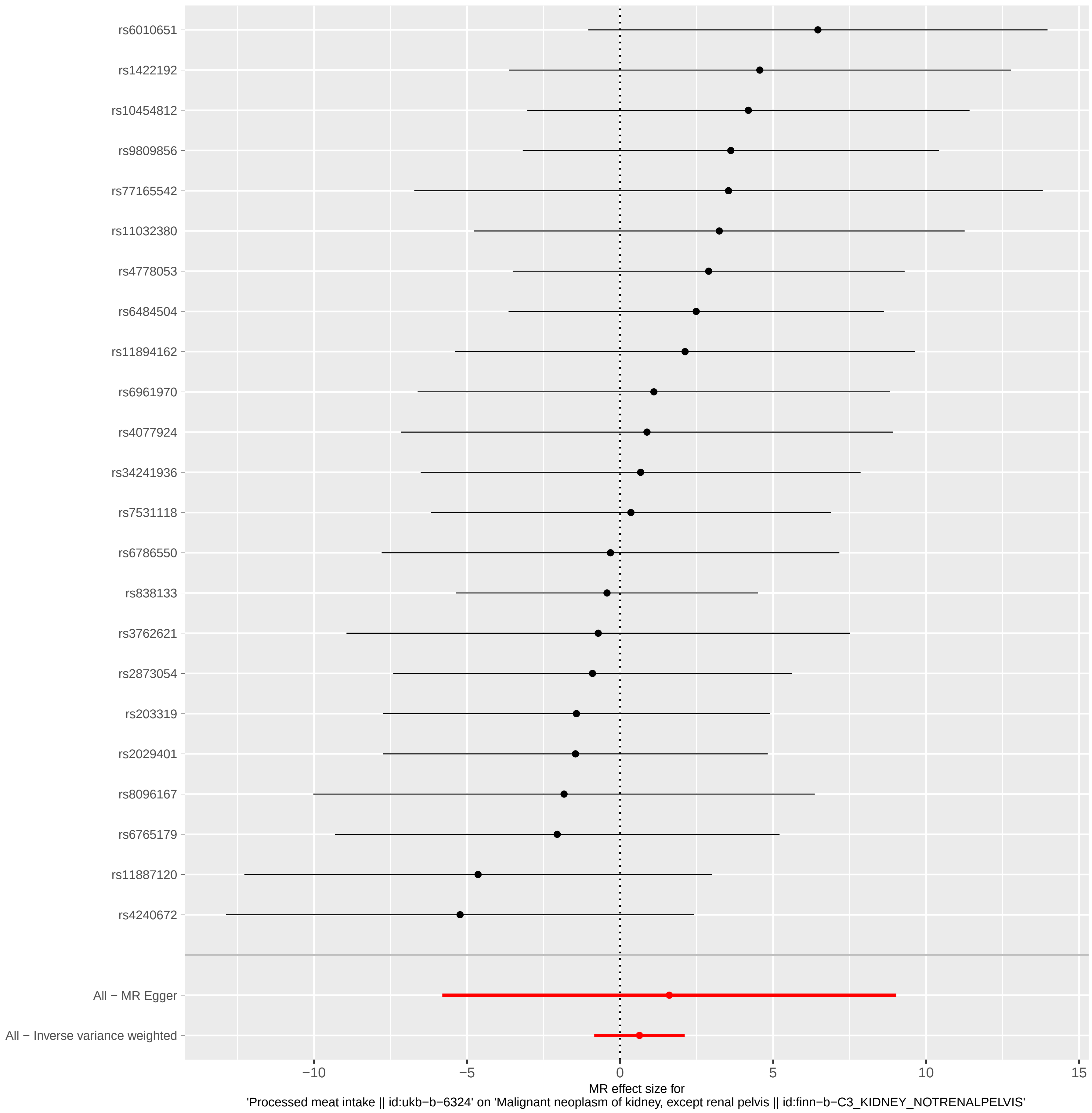
rs6010651
rs1422192
rs10454812
rs9809856
rs77165542
rs11032380
rs4778053
rs6484504
rs11894162
rs6961970
rs4077924
rs34241936
rs7531118
rs6786550
rs838133
rs3762621
rs2873054
rs203319
rs2029401
rs8096167
rs6765179
rs11887120
rs4240672
All − MR Egger
All − Inverse variance weighted
−10
−5
0
5
10
15
MR effect size for
'Processed meat intake || id:ukb−b−6324' on 'Malignant neoplasm of kidney, except renal pelvis || id:finn−b−C3_KIDNEY_NOTRENALPELVIS'

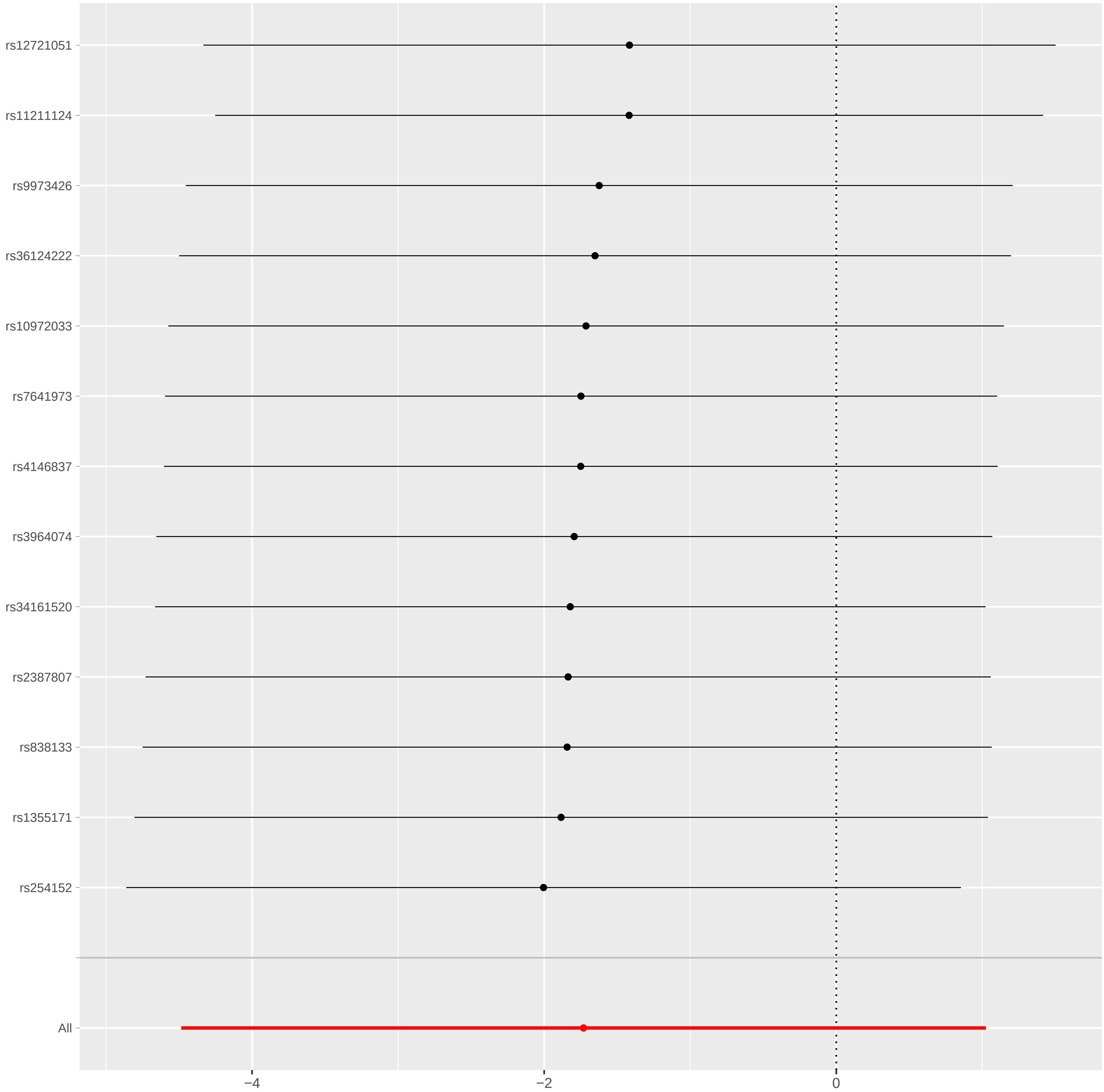

MR leave−one−out sensitivity analysis for
'Pork intake || id:ukb−b−5640' on 'Malignant neoplasm of kidney, except renal pelvis || id:finn−b−C3_KIDNEY_NOTRENALPELVIS'

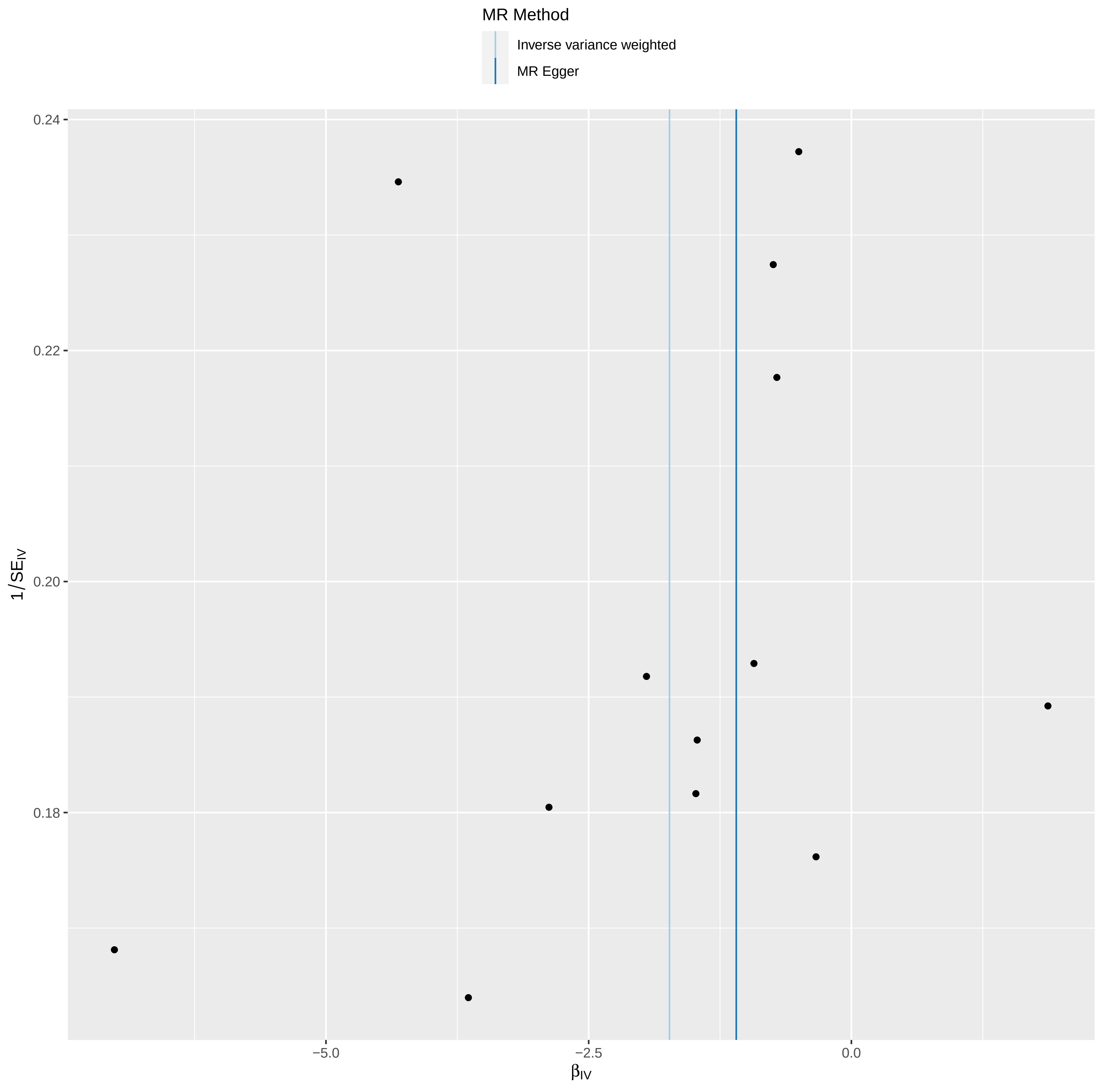

MR Method
Inverse variance weighted
MR Egger
0.24
0.22
0.20
0.18
1/SE_IV
−5.0
−2.5
0.0
β_IV

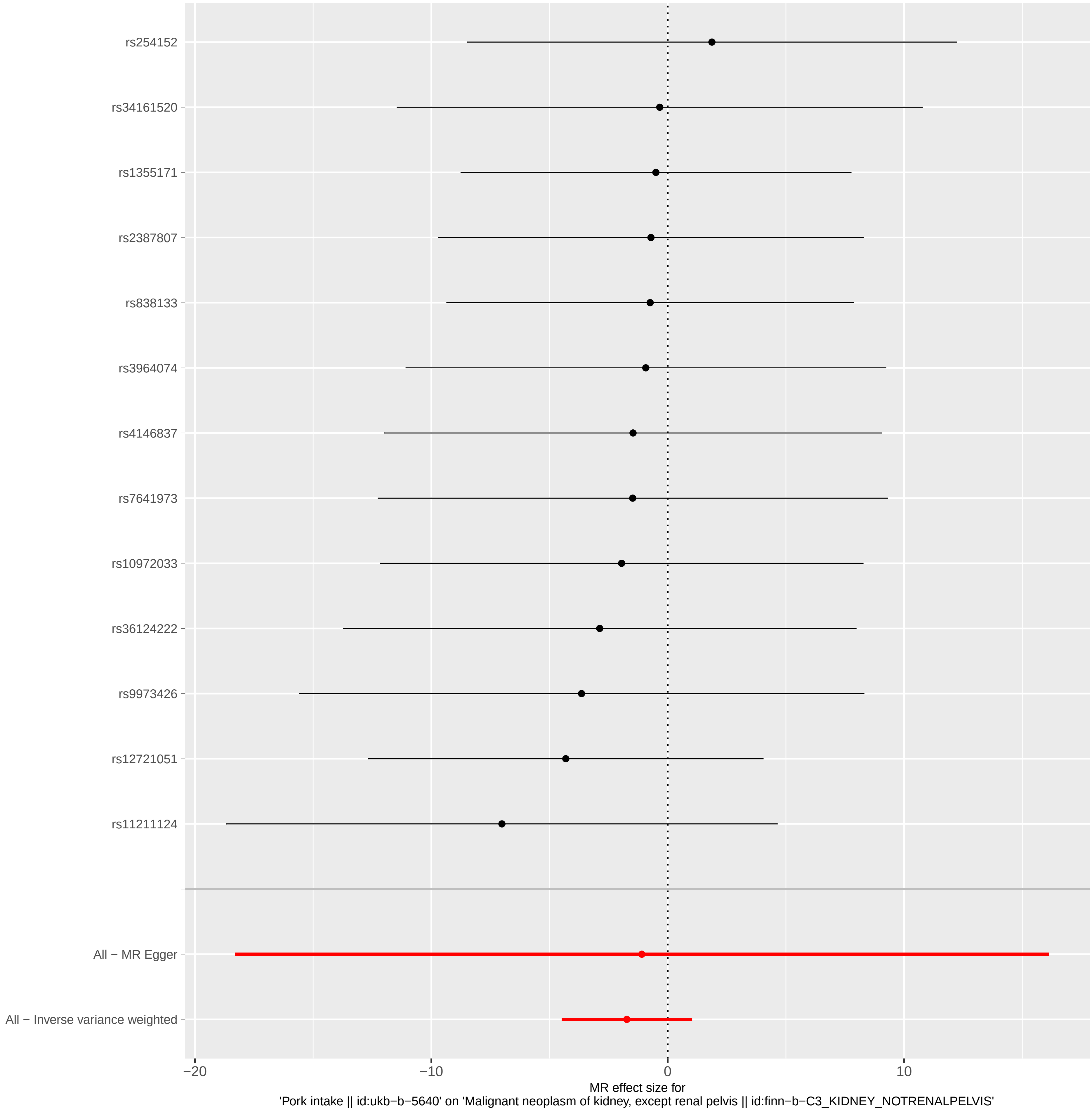

rs254152
rs34161520
rs1355171
rs2387807
rs838133
rs3964074
rs4146837
rs7641973
rs10972033
rs36124222
rs9973426
rs12721051
rs11211124
All − MR Egger
All − Inverse variance weighted
−20
−10
0
10
MR effect size for
'Pork intake || id:ukb−b−5640' on 'Malignant neoplasm of kidney, except renal pelvis || id:finn−b−C3_KIDNEY_NOTRENALPELVIS'

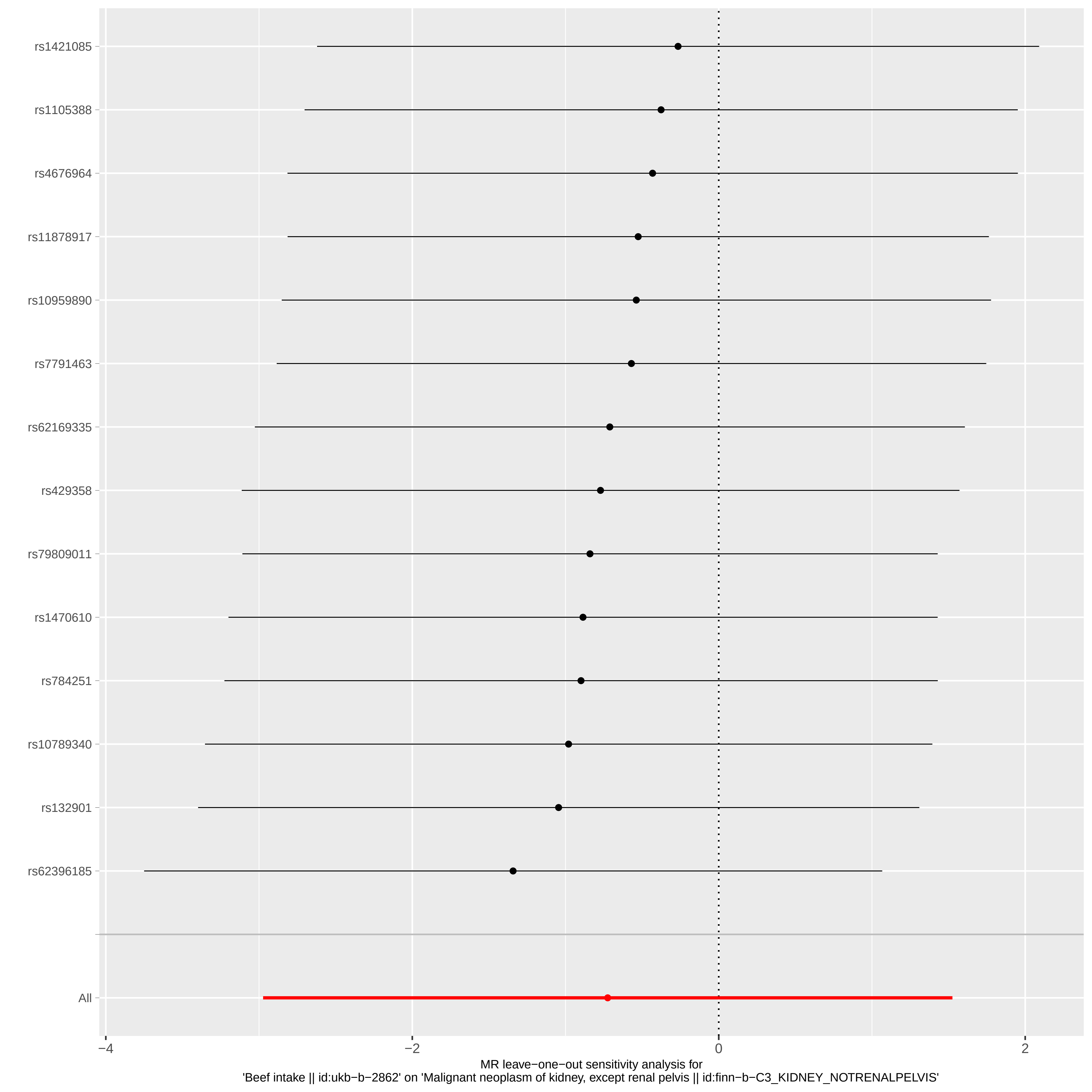
rs1421085
rs1105388
rs4676964
rs11878917
rs10959890
rs7791463
rs62169335
rs429358
rs79809011
rs1470610
rs784251
rs10789340
rs132901
rs62396185
All
−4
−2
0
2
MR leave−one−out sensitivity analysis for
'Beef intake || id:ukb−b−2862' on 'Malignant neoplasm of kidney, except renal pelvis || id:finn−b−C3_KIDNEY_NOTRENALPELVIS'

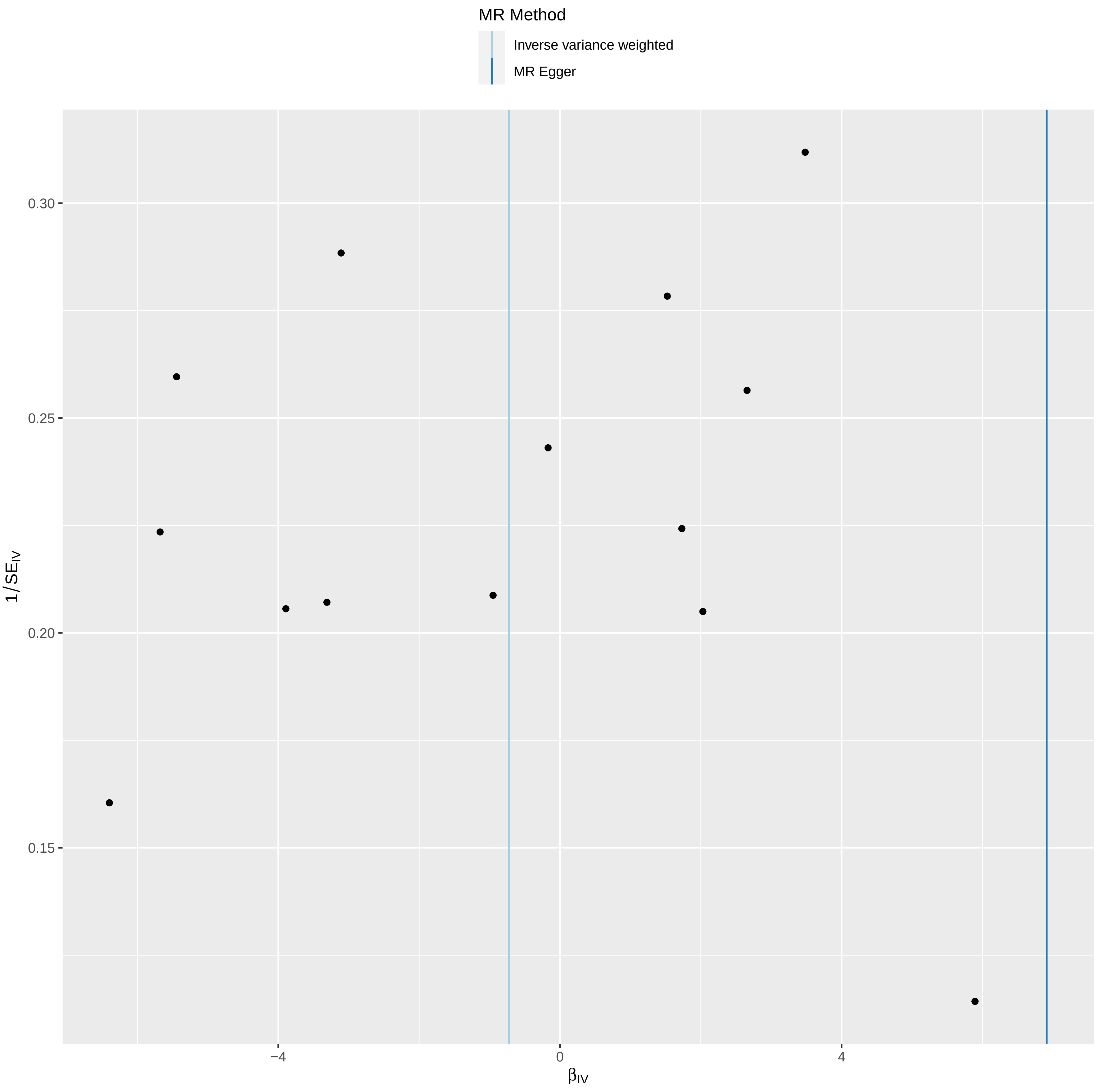

MR Method
Inverse variance weighted
MR Egger
0.30
0.25
0.20
0.15
1/SE_IV
−4
0
4
β_IV

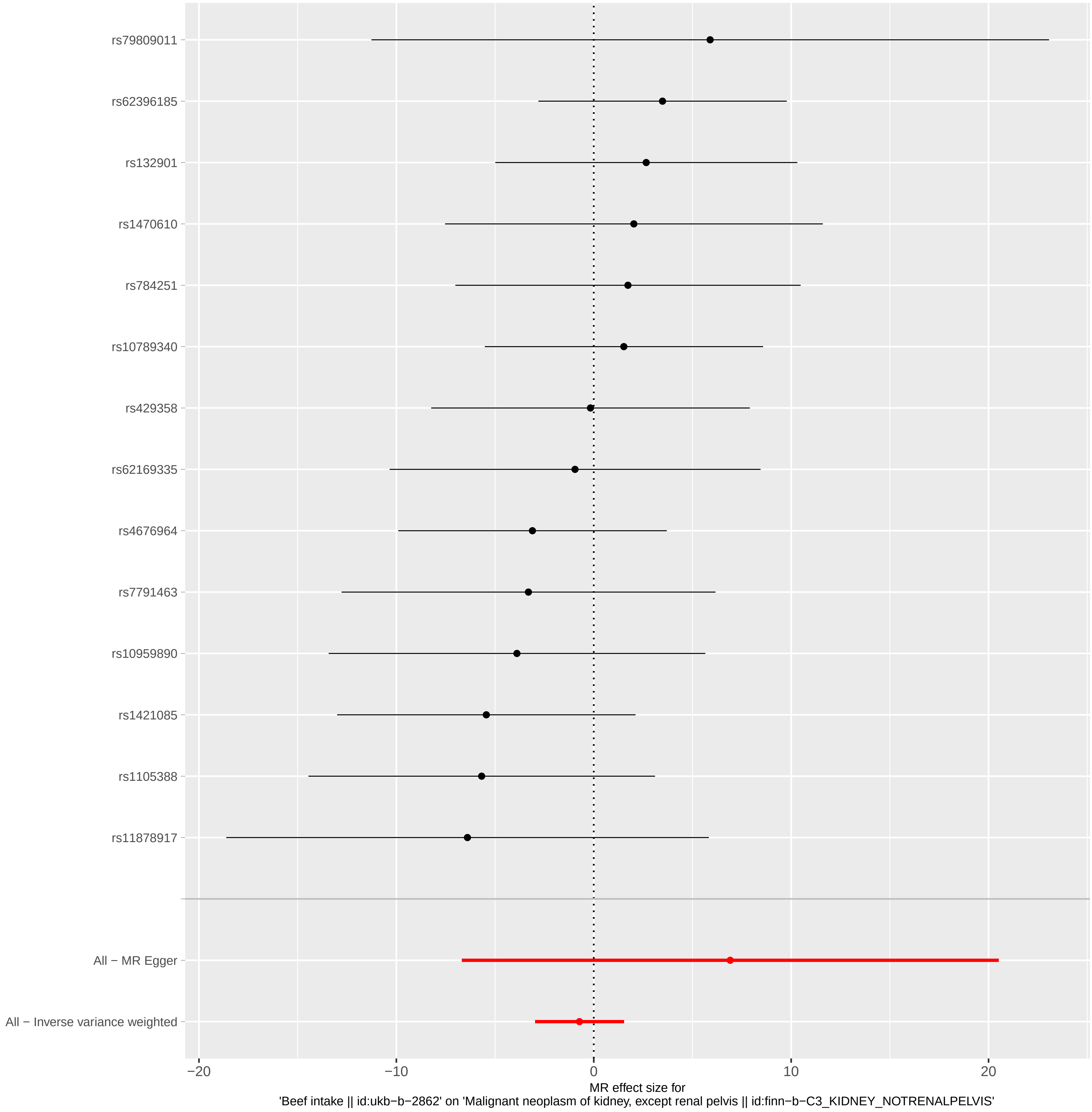

rs79809011
rs62396185
rs132901
rs1470610
rs784251
rs10789340
rs429358
rs62169335
rs4676964
rs7791463
rs10959890
rs1421085
rs1105388
rs11878917
All − MR Egger
All − Inverse variance weighted
−20
−10
0
10
20
MR effect size for
'Beef intake || id:ukb−b−2862' on 'Malignant neoplasm of kidney, except renal pelvis || id:finn−b−C3_KIDNEY_NOTRENALPELVIS'

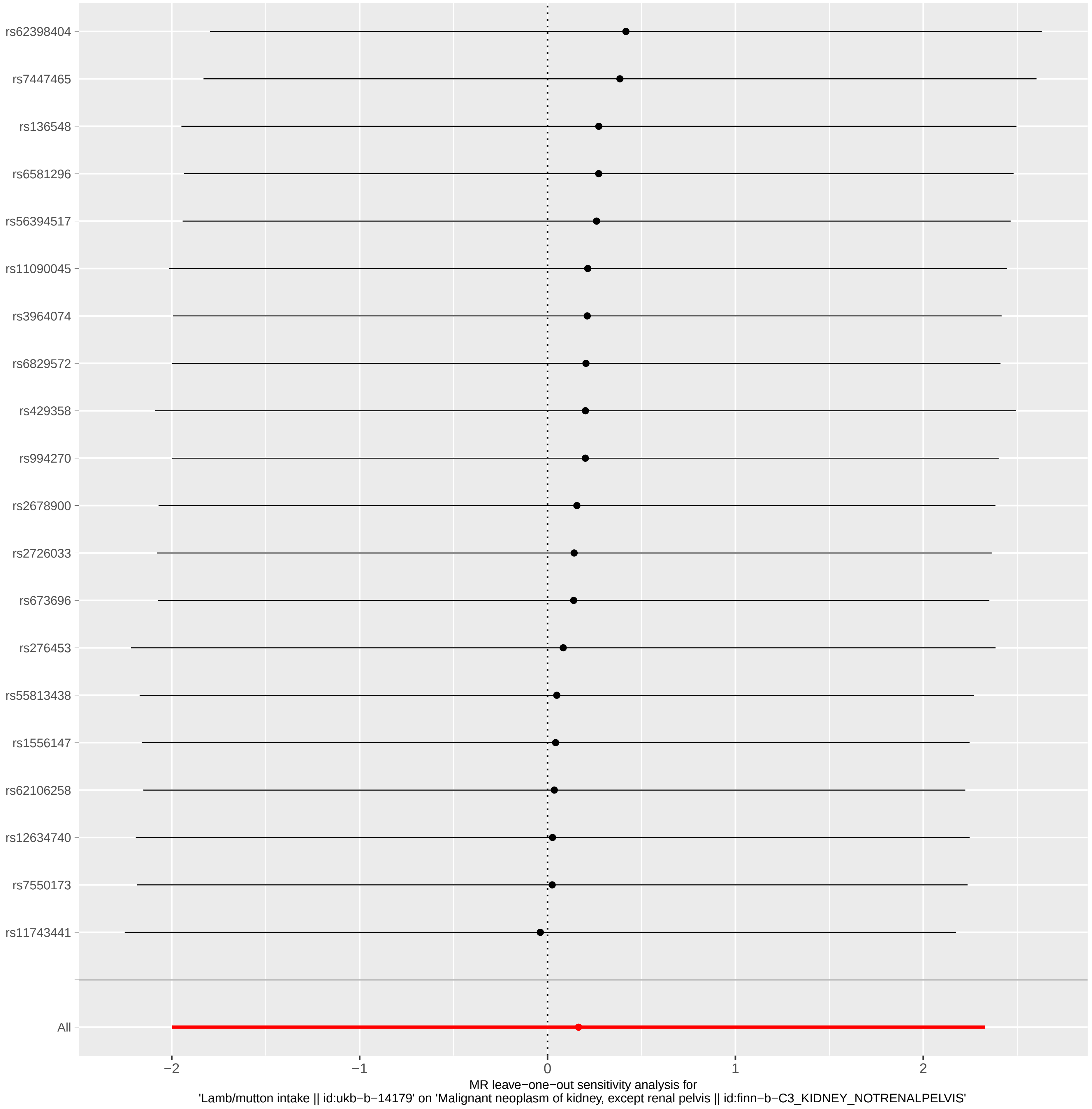
rs62398404
rs7447465
rs136548
rs6581296
rs56394517
rs11090045
rs3964074
rs6829572
rs429358
rs994270
rs2678900
rs2726033
rs673696
rs276453
rs55813438
rs1556147
rs62106258
rs12634740
rs7550173
rs11743441
All
−2
−1
0
1
2
MR leave−one−out sensitivity analysis for
'Lamb/mutton intake || id:ukb−b−14179' on 'Malignant neoplasm of kidney, except renal pelvis || id:finn−b−C3_KIDNEY_NOTRENALPELVIS'

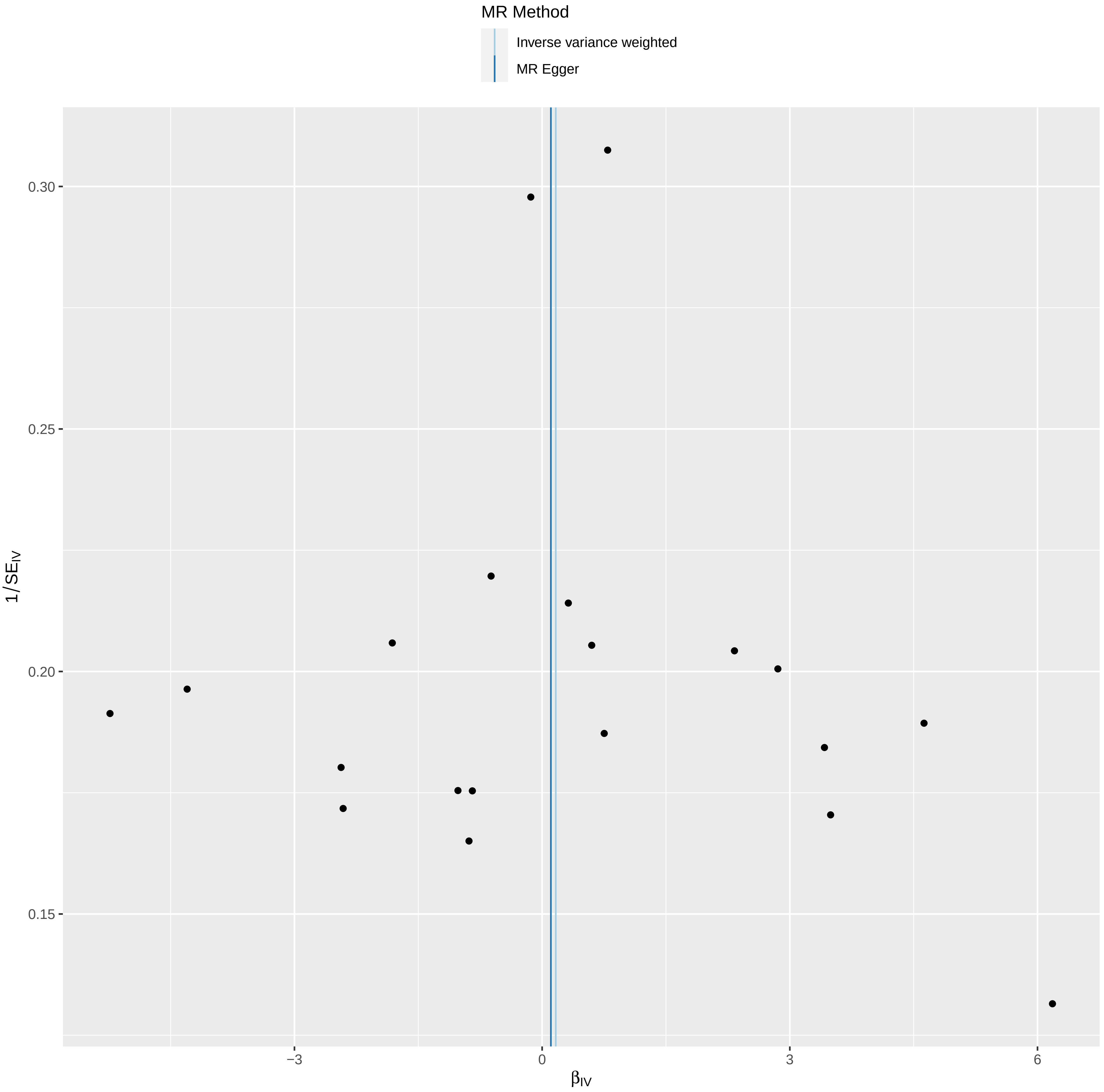

MR Method
Inverse variance weighted
MR Egger
1/SE_IV
0.30
0.25
0.20
0.15
−3
0
3
6
β_IV

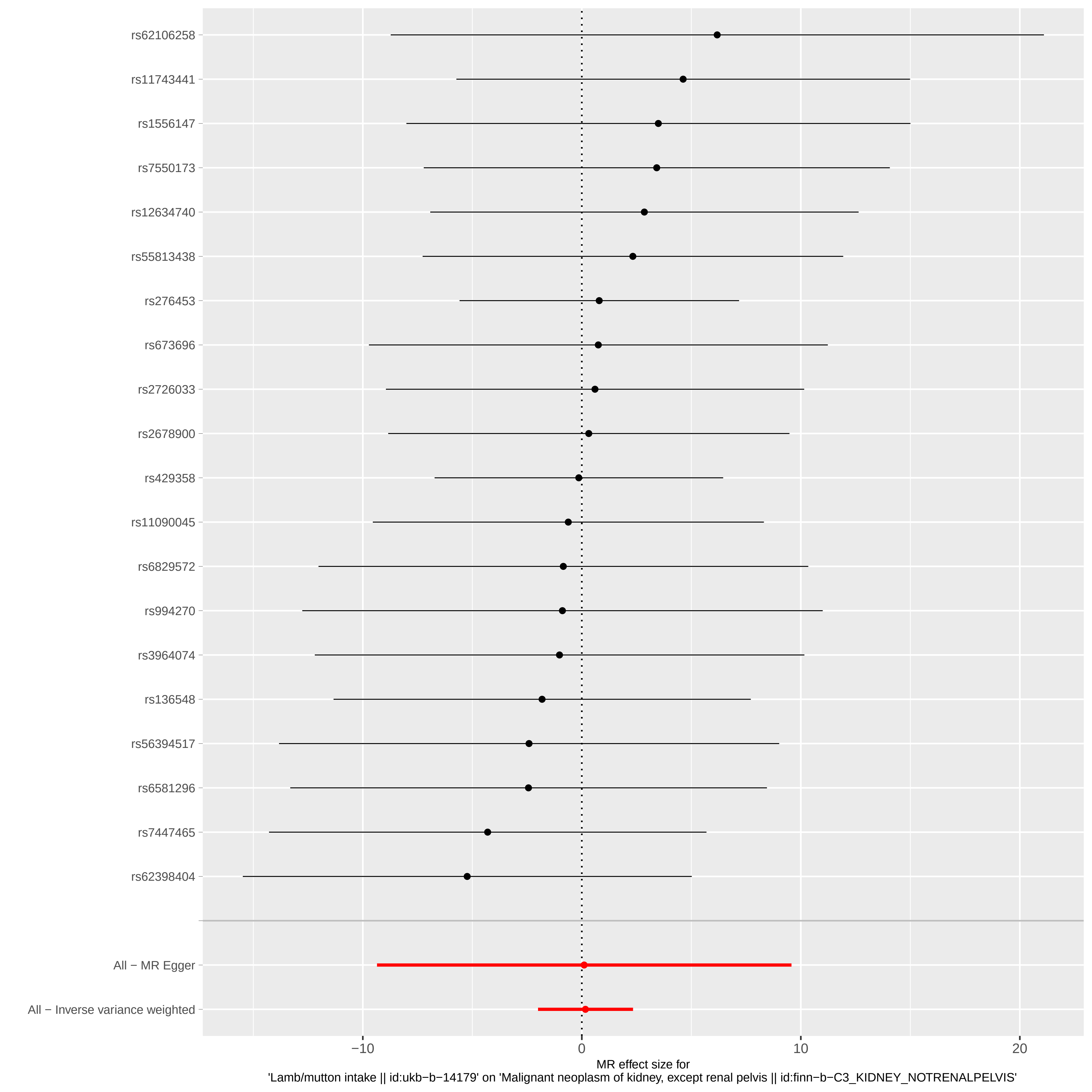
rs62106258
rs11743441
rs1556147
rs7550173
rs12634740
rs55813438
rs276453
rs673696
rs2726033
rs2678900
rs429358
rs11090045
rs6829572
rs994270
rs3964074
rs136548
rs56394517
rs6581296
rs7447465
rs62398404
All − MR Egger
All − Inverse variance weighted
−10
0
10
20
MR effect size for
'Lamb/mutton intake || id:ukb−b−14179' on 'Malignant neoplasm of kidney, except renal pelvis || id:finn−b−C3_KIDNEY_NOTRENALPELVIS'

Figure S6. Leave-one-out analysis, funnel plot and MR effect size for processed meat, pork, beef and mutton intake on gastric cancer.

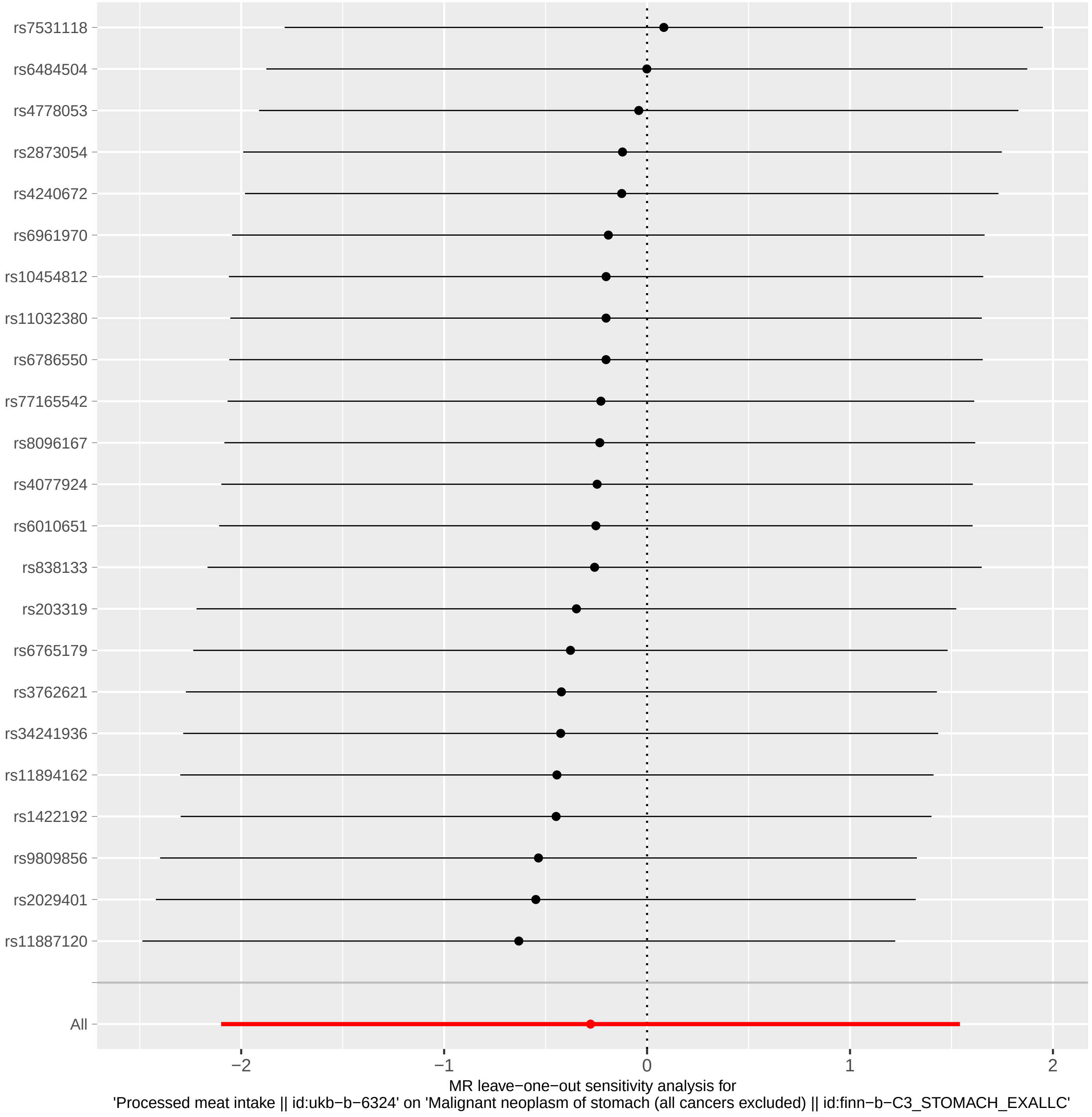

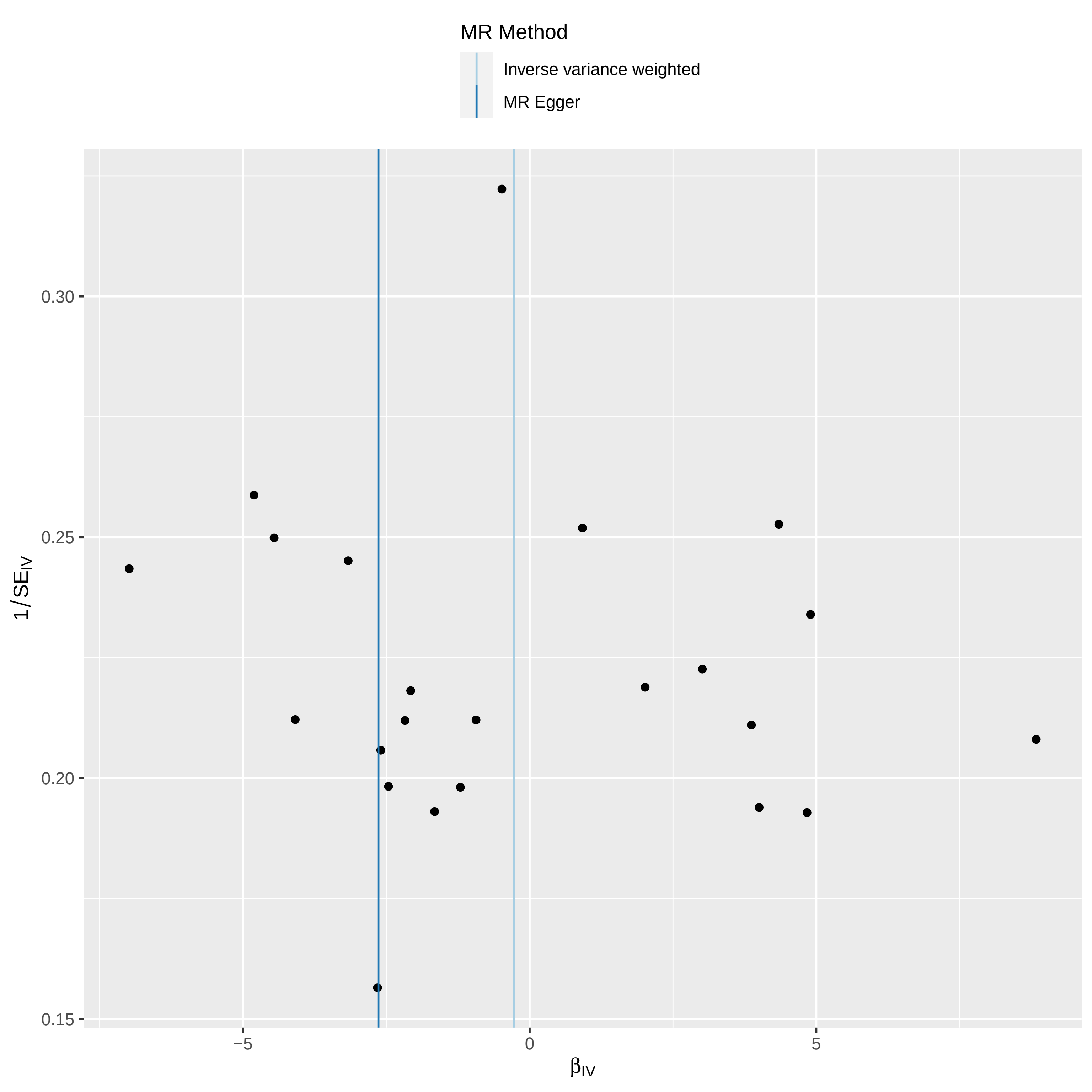
MR Method
Inverse variance weighted
MR Egger
0.30
0.25
0.20
0.15
$1/SE_{IV}$
−5
0
5
β_{IV}

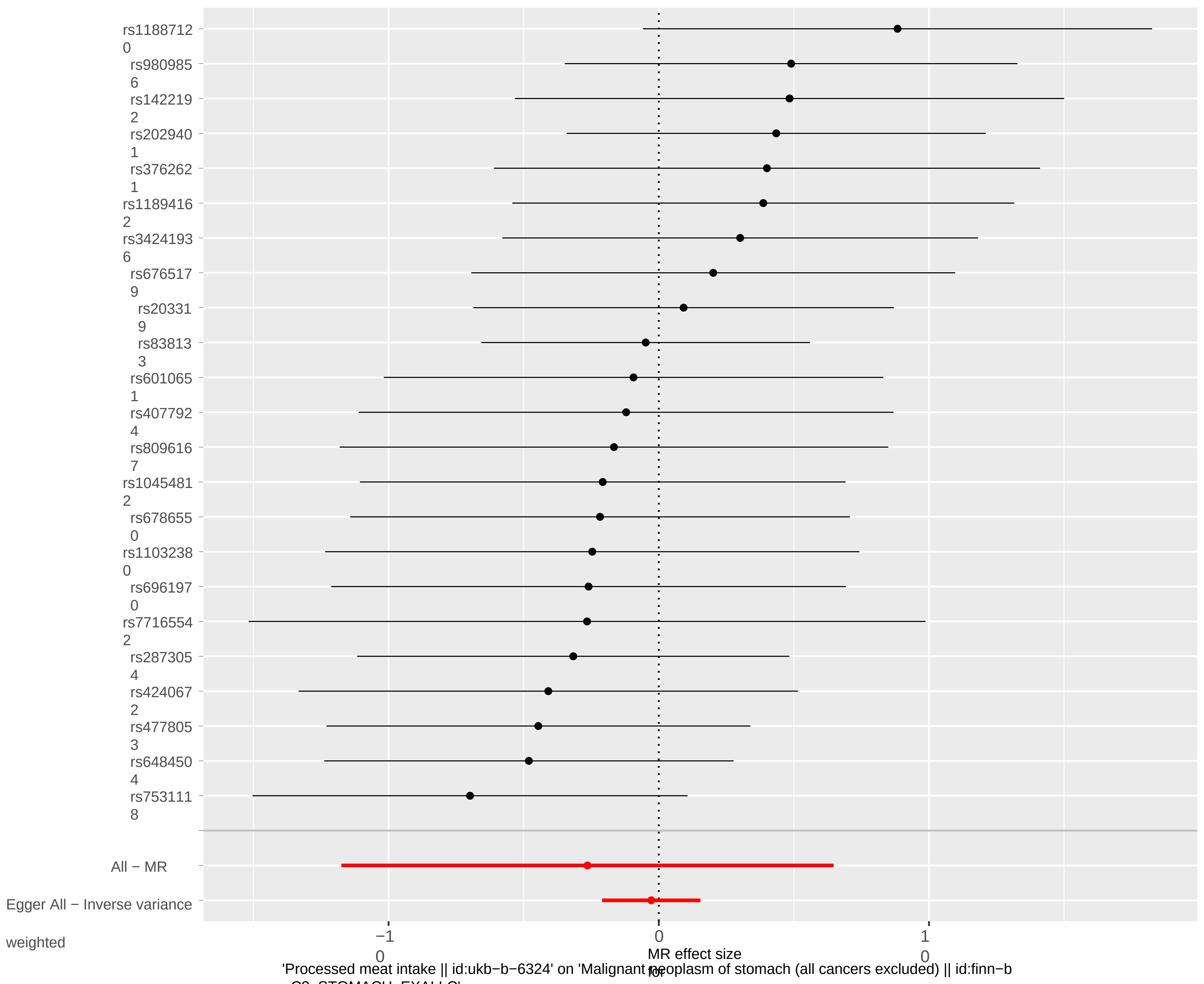
rs1188712
0
rs980985
6
rs142219
2
rs202940
1
rs376262
1
rs1189416
2
rs3424193
6
rs676517
9
rs20331
9
rs83813
3
rs601065
1
rs407792
4
rs809616
7
rs1045481
2
rs678655
0
rs1103238
0
rs696197
0
rs7716554
2
rs287305
4
rs424067
2
rs477805
3
rs648450
4
rs753111
8
All − MR
Egger All − Inverse variance
weighted
−1
0
0
1
0
MR effect size
for
'Processed meat intake || id:ukb−b−6324' on 'Malignant neoplasm of stomach (all cancers excluded) || id:finn−b

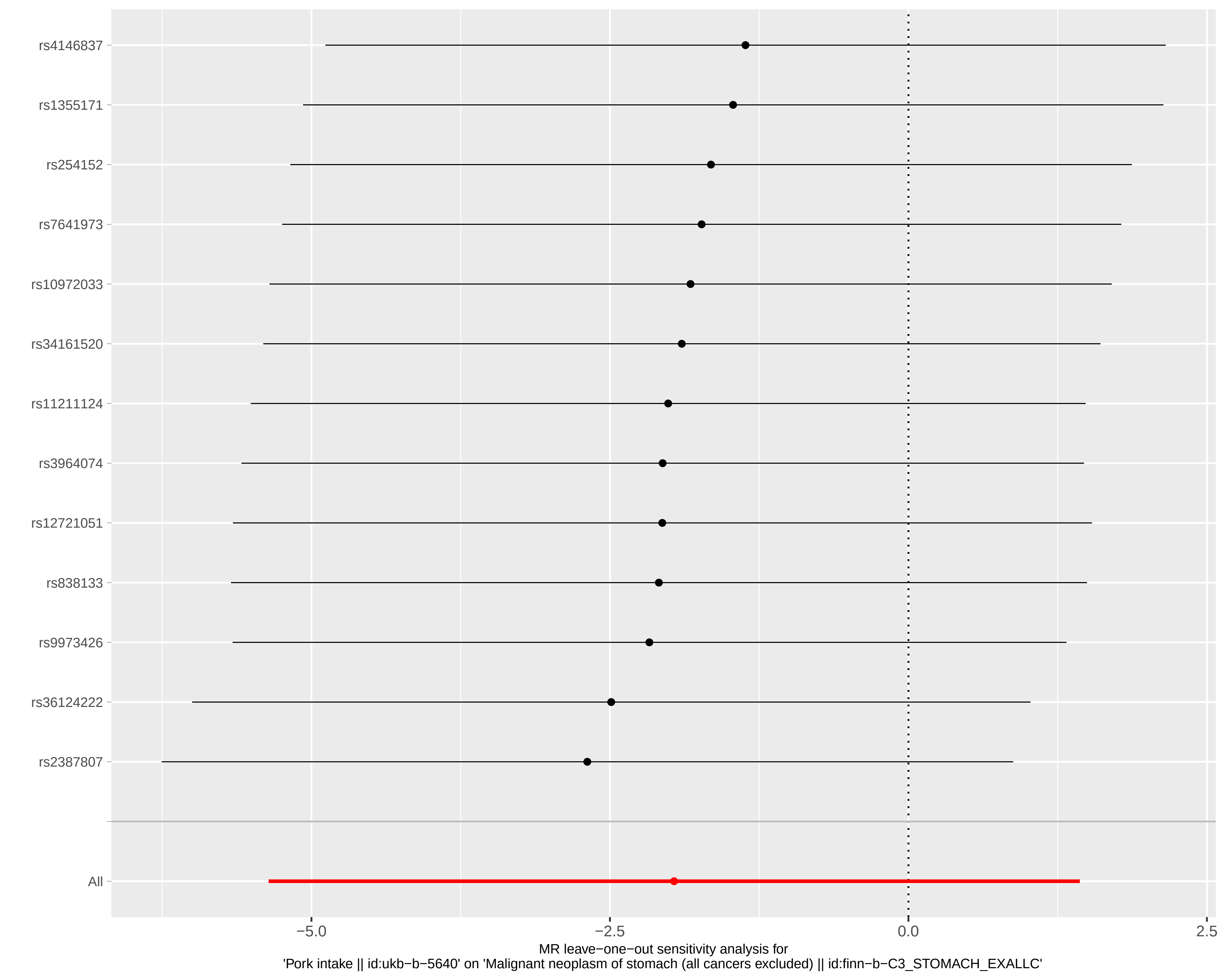
rs4146837
rs1355171
rs254152
rs7641973
rs10972033
rs34161520
rs11211124
rs3964074
rs12721051
rs838133
rs9973426
rs36124222
rs2387807
All
−5.0
−2.5
0.0
2.5
MR leave−one−out sensitivity analysis for
'Pork intake || id:ukb−b−5640' on 'Malignant neoplasm of stomach (all cancers excluded) || id:finn−b−C3_STOMACH_EXALLC'

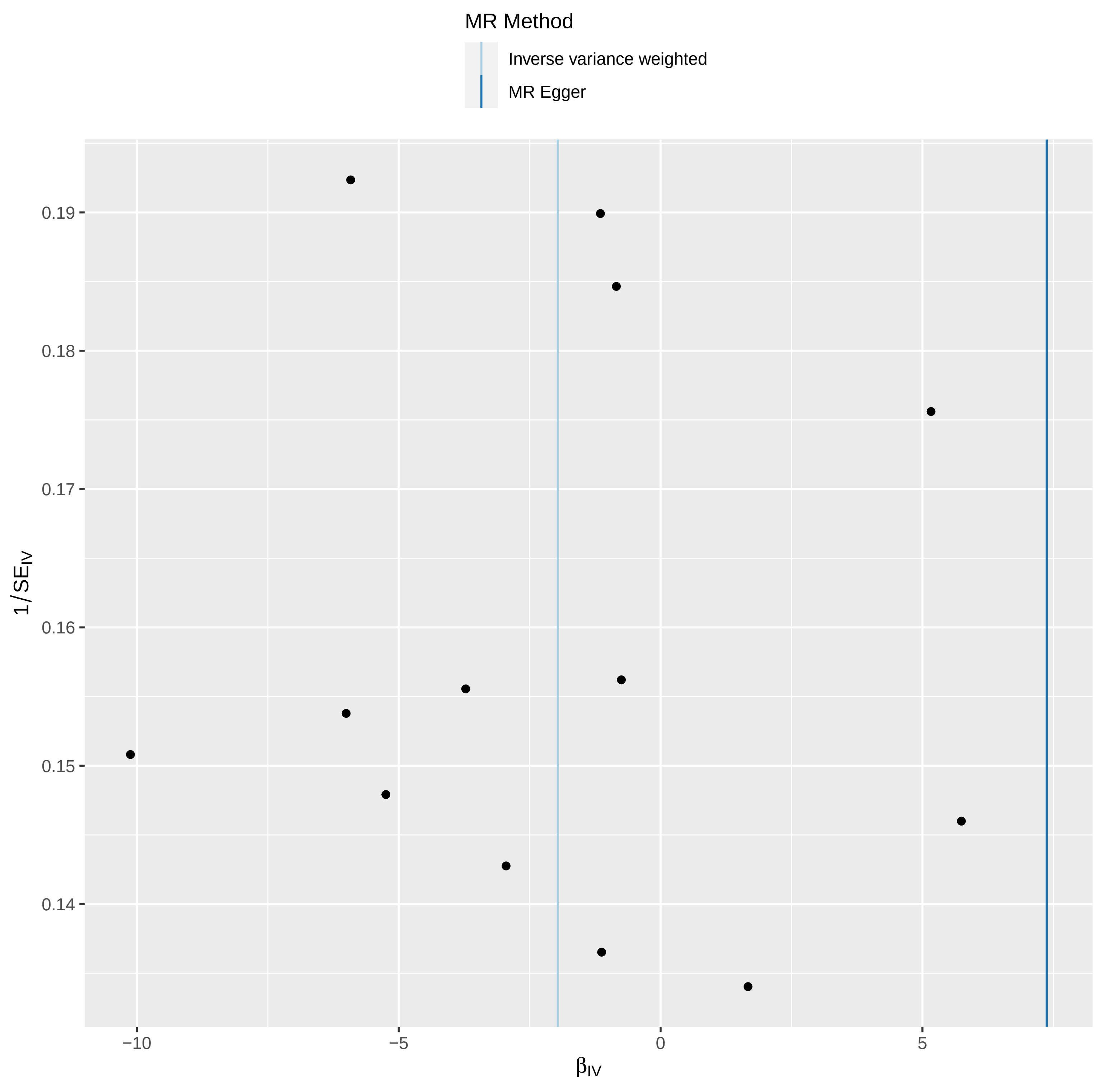
MR Method
Inverse variance weighted
MR Egger
0.19
0.18
0.17
0.16
0.15
0.14
$1/SE_{IV}$
−10
−5
0
5
β_{IV}

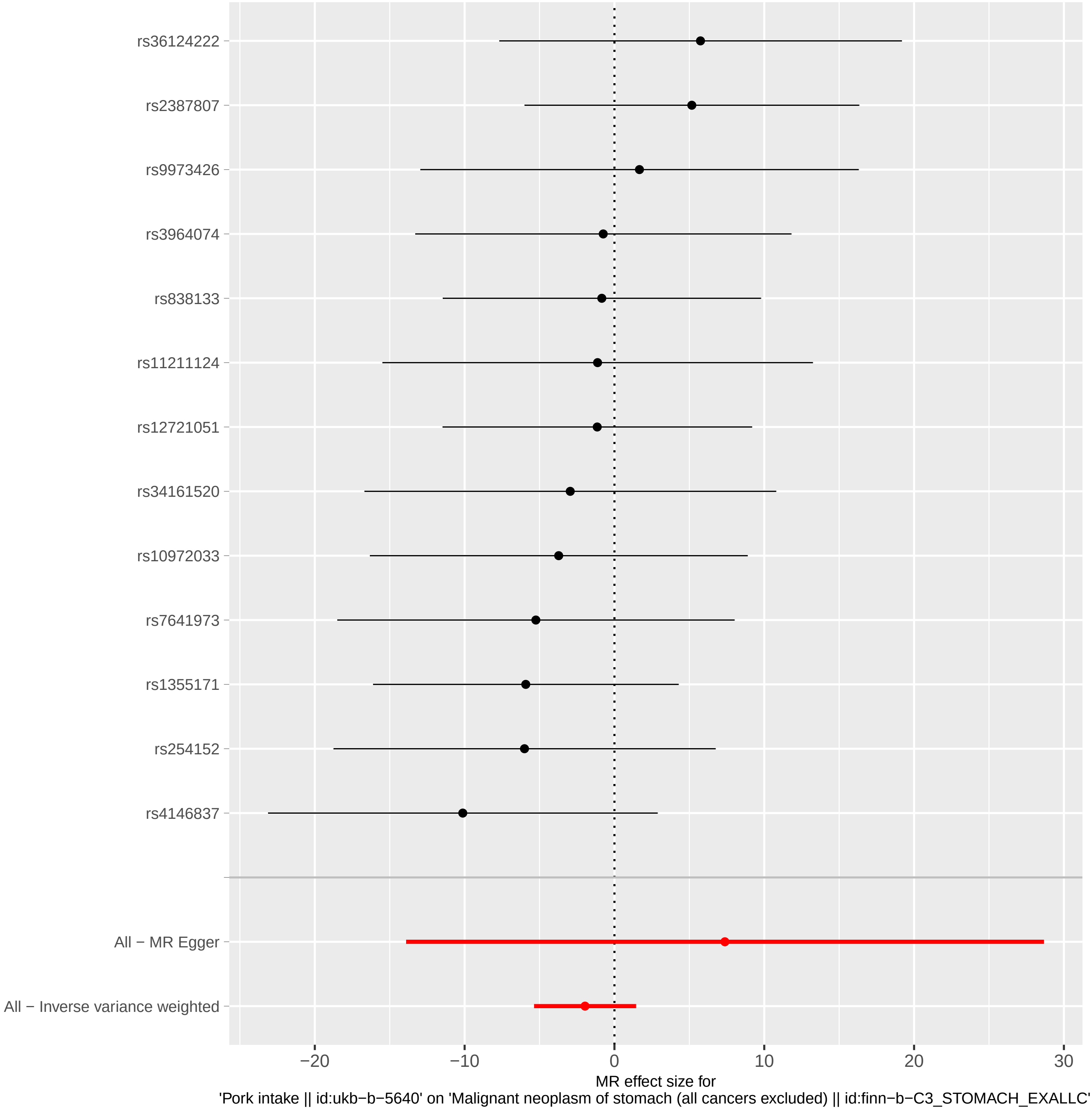
rs36124222
rs2387807
rs9973426
rs3964074
rs838133
rs11211124
rs12721051
rs34161520
rs10972033
rs7641973
rs1355171
rs254152
rs4146837
All − MR Egger
All − Inverse variance weighted
−20
−10
0
10
20
30
MR effect size for
'Pork intake || id:ukb−b−5640' on 'Malignant neoplasm of stomach (all cancers excluded) || id:finn−b−C3_STOMACH_EXALLC'

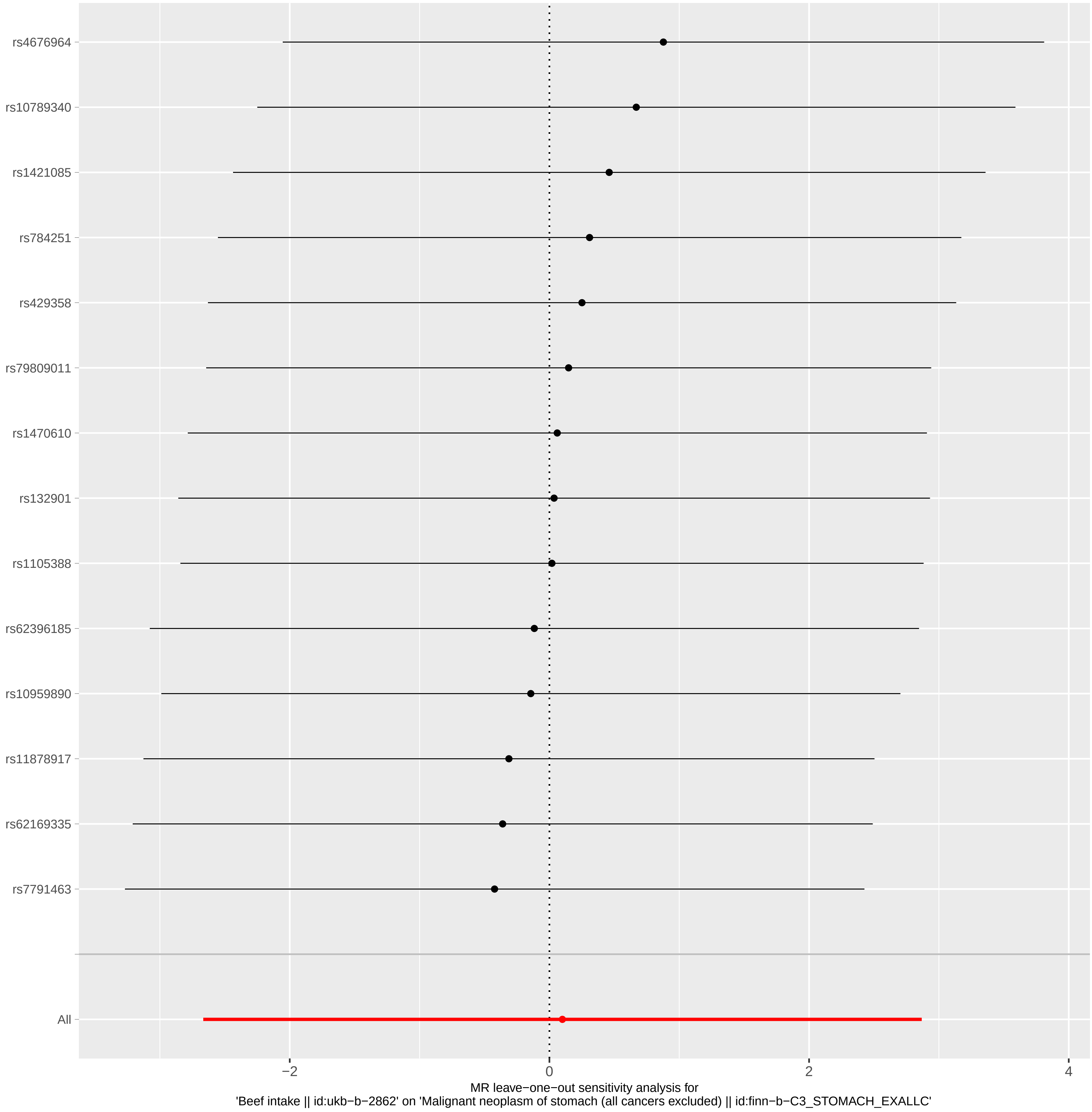
rs4676964
rs10789340
rs1421085
rs784251
rs429358
rs79809011
rs1470610
rs132901
rs1105388
rs62396185
rs10959890
rs11878917
rs62169335
rs7791463
All
−2
0
2
4
MR leave−one−out sensitivity analysis for
'Beef intake || id:ukb−b−2862' on 'Malignant neoplasm of stomach (all cancers excluded) || id:finn−b−C3_STOMACH_EXALLC'

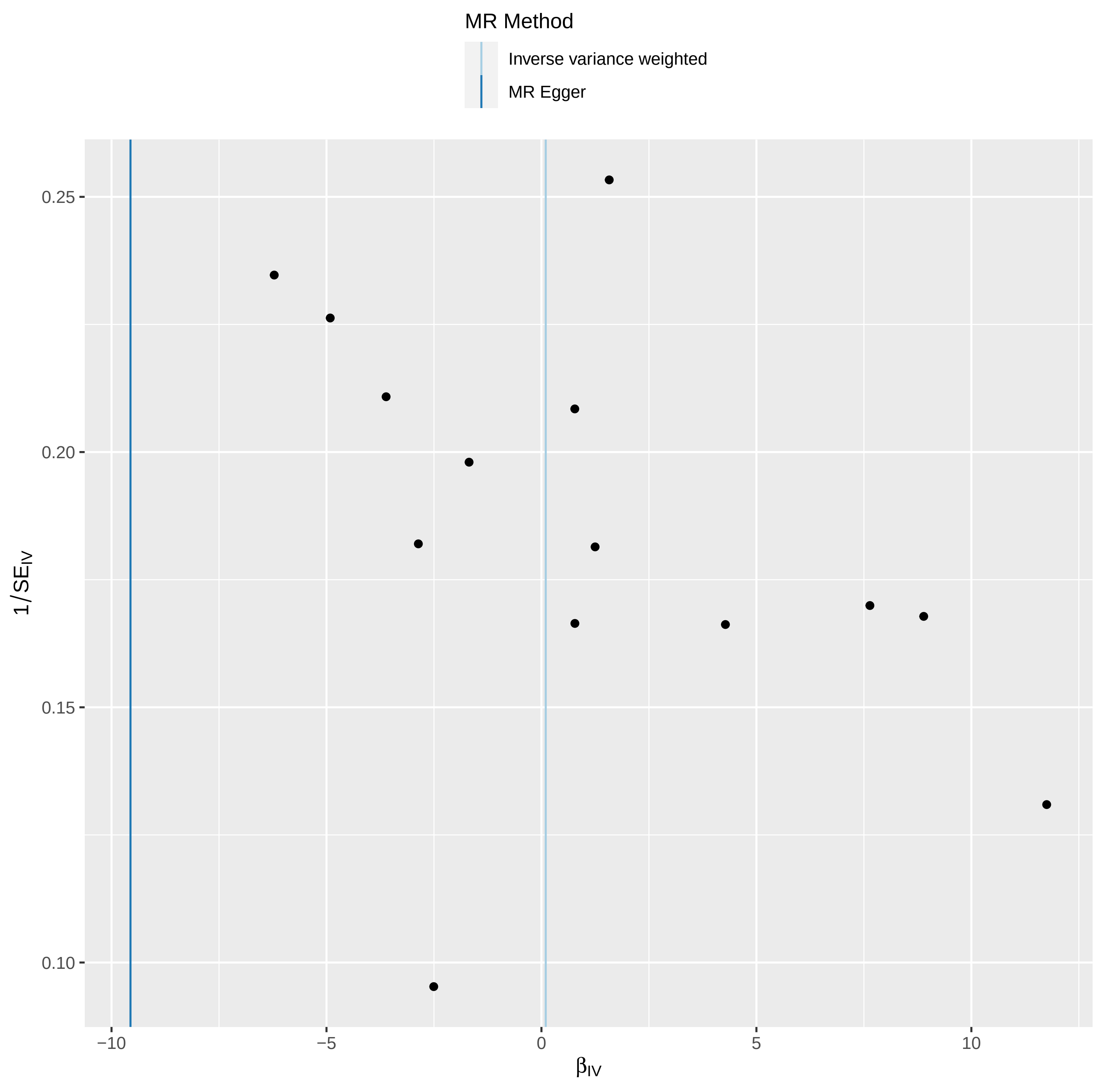
MR Method
Inverse variance weighted
MR Egger
0.25
0.20
0.15
0.10
1/SE_IV
−10
−5
0
5
10
β_IV

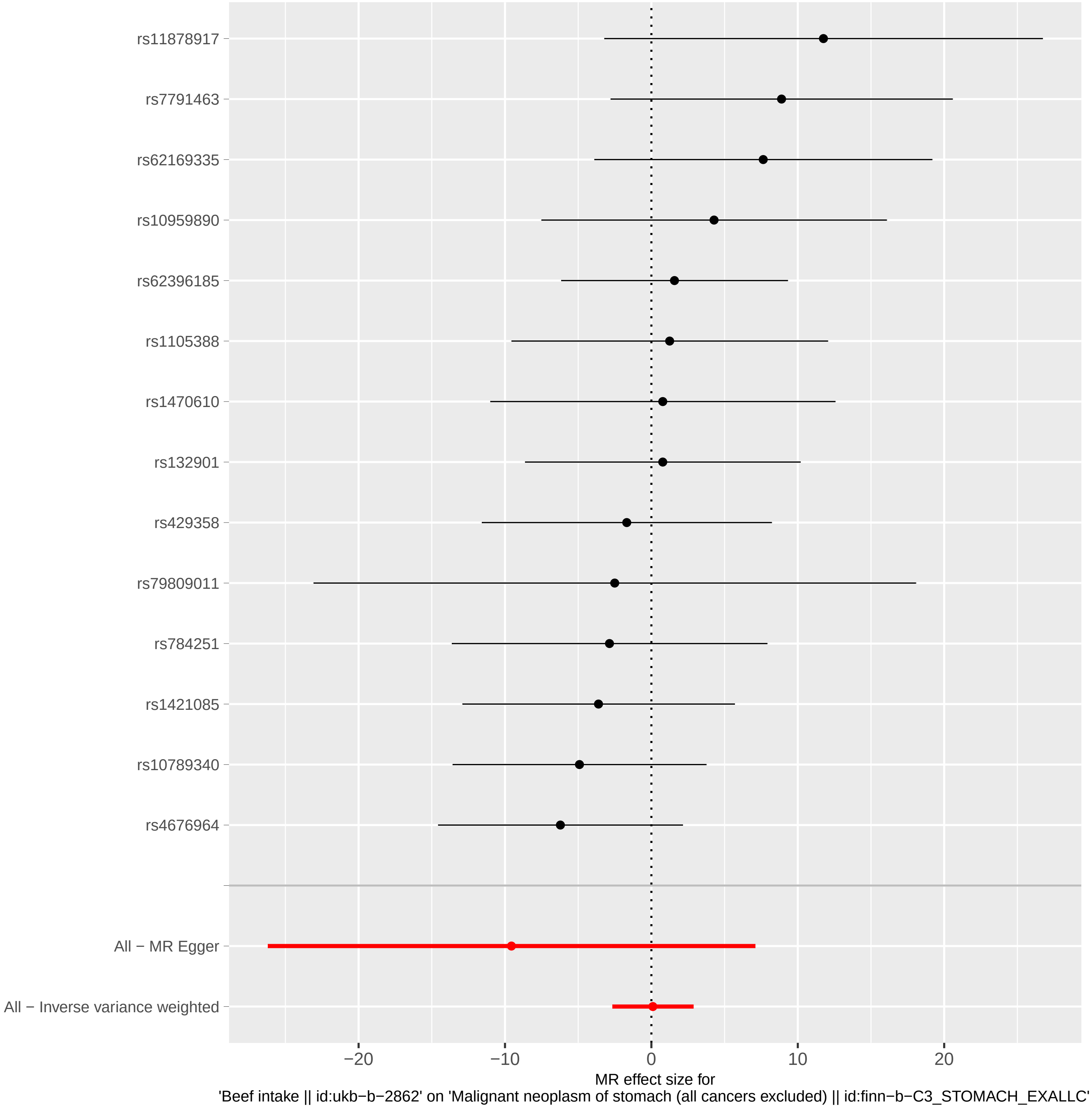
rs11878917
rs7791463
rs62169335
rs10959890
rs62396185
rs1105388
rs1470610
rs132901
rs429358
rs79809011
rs784251
rs1421085
rs10789340
rs4676964
All − MR Egger
All − Inverse variance weighted
−20
−10
0
10
20
MR effect size for
'Beef intake || id:ukb−b−2862' on 'Malignant neoplasm of stomach (all cancers excluded) || id:finn−b−C3_STOMACH_EXALLC'

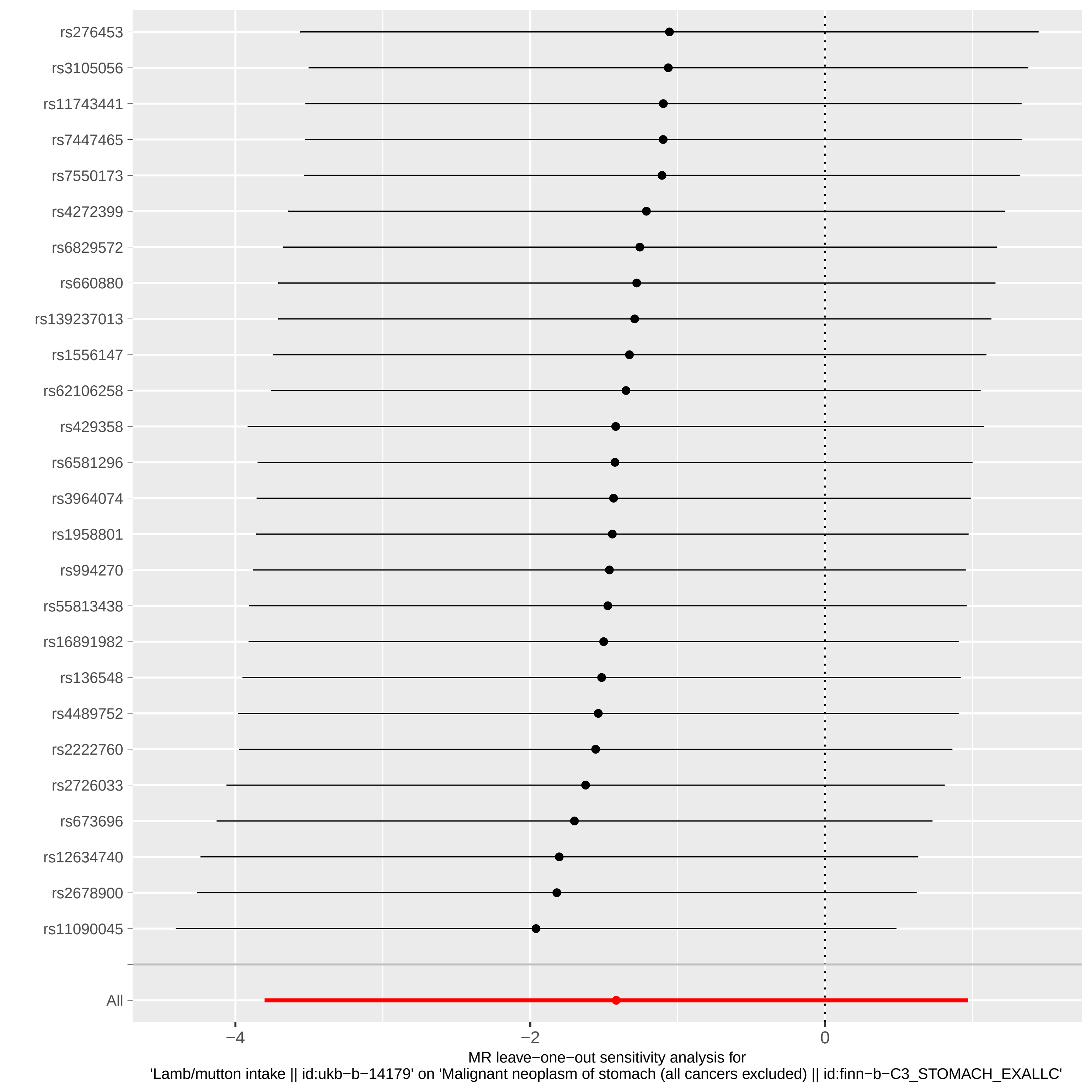
rs276453
rs3105056
rs11743441
rs7447465
rs7550173
rs4272399
rs6829572
rs660880
rs139237013
rs1556147
rs62106258
rs429358
rs6581296
rs3964074
rs1958801
rs994270
rs55813438
rs16891982
rs136548
rs4489752
rs2222760
rs2726033
rs673696
rs12634740
rs2678900
rs11090045
All
−4
−2
0
MR leave−one−out sensitivity analysis for
'Lamb/mutton intake || id:ukb−b−14179' on 'Malignant neoplasm of stomach (all cancers excluded) || id:finn−b−C3_STOMACH_EXALLC'

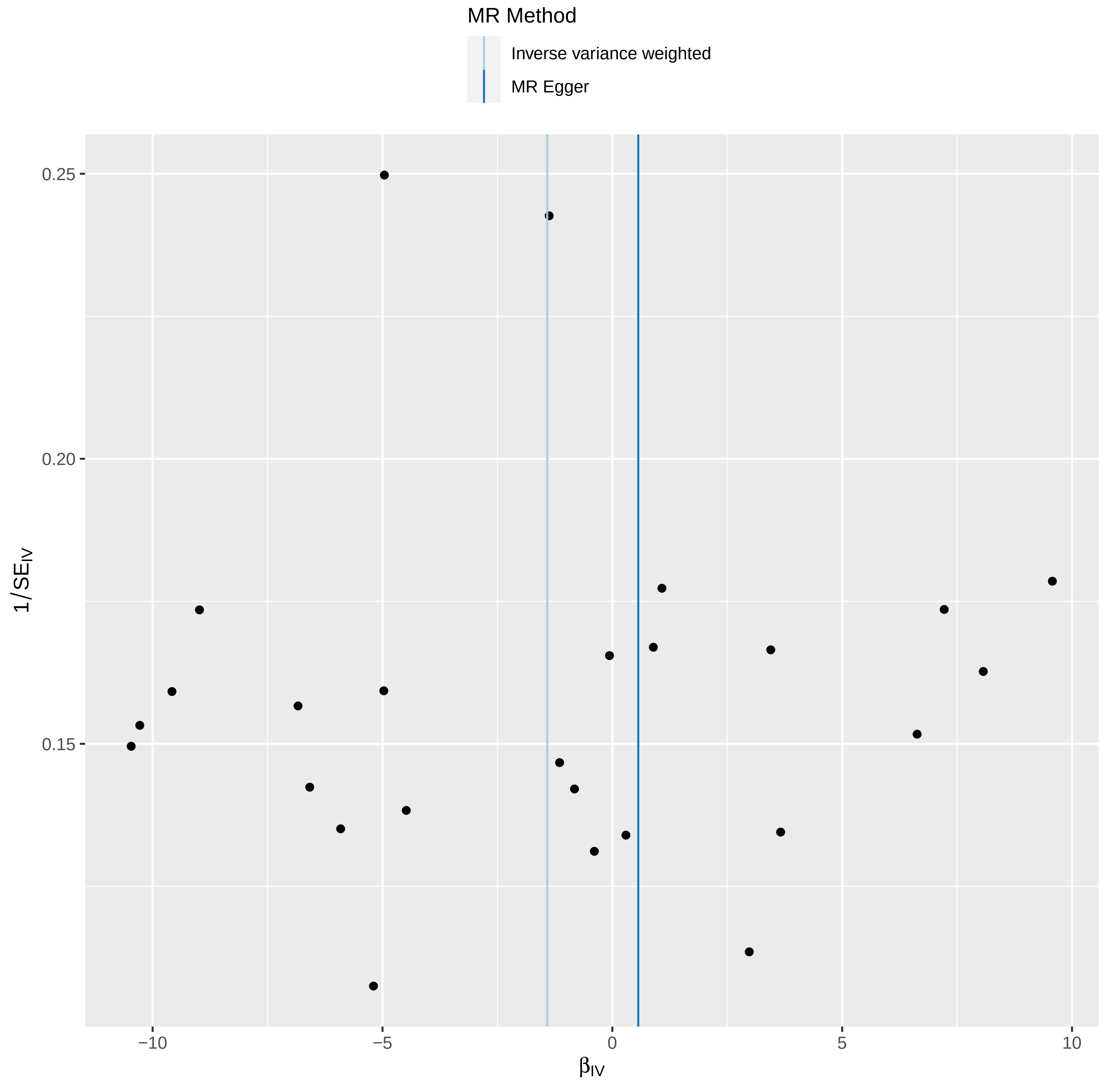
MR Method
Inverse variance weighted
MR Egger
0.25
0.20
0.15
1/SE$_{IV}$
−10
−5
0
5
10
β$_{IV}$

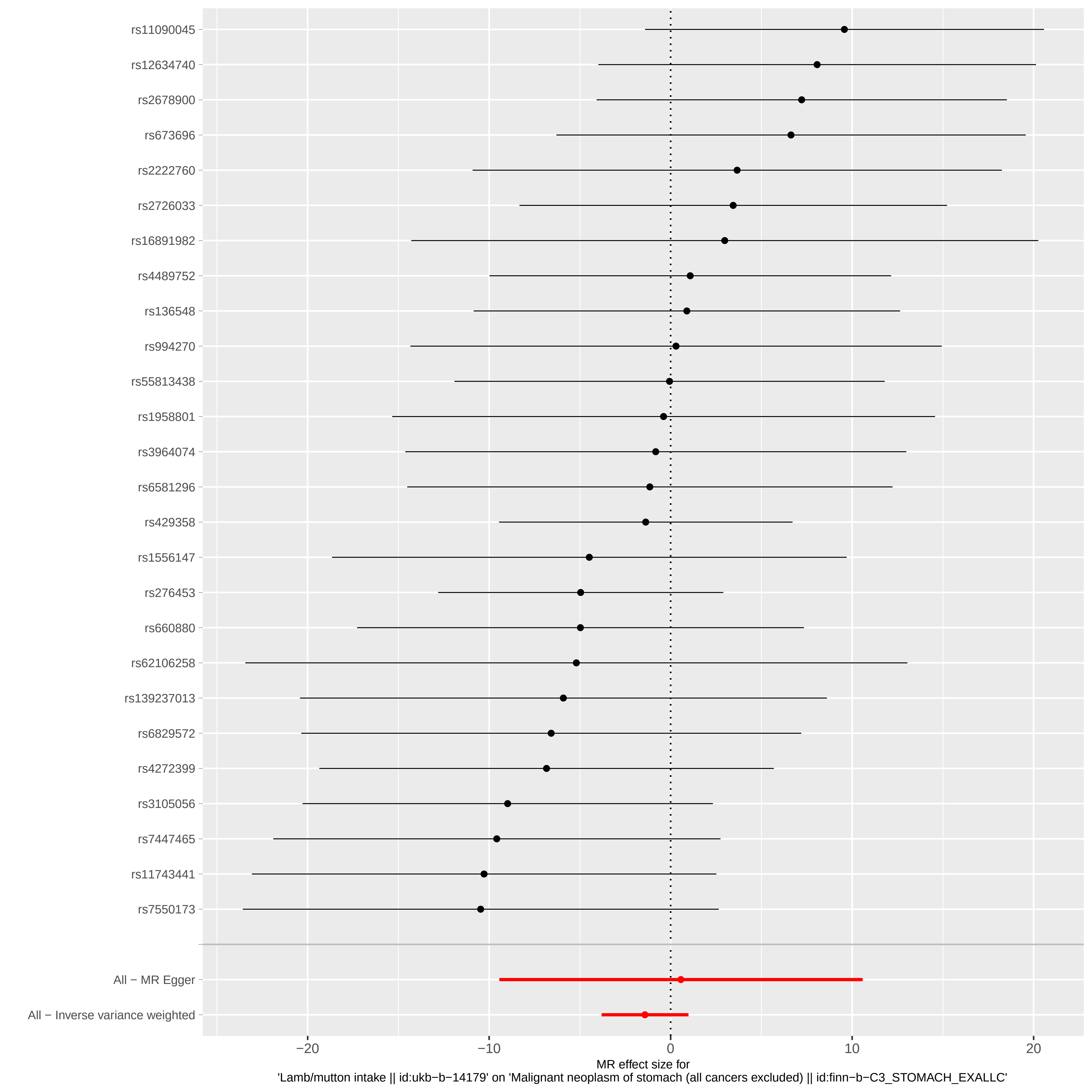
rs11090045
rs12634740
rs2678900
rs673696
rs2222760
rs2726033
rs16891982
rs4489752
rs136548
rs994270
rs55813438
rs1958801
rs3964074
rs6581296
rs429358
rs1556147
rs276453
rs660880
rs62106258
rs139237013
rs6829572
rs4272399
rs3105056
rs7447465
rs11743441
rs7550173
All − MR Egger
All − Inverse variance weighted
−20
−10
0
10
20
MR effect size for
'Lamb/mutton intake || id:ukb−b−14179' on 'Malignant neoplasm of stomach (all cancers excluded) || id:finn−b−C3_STOMACH_EXALLC'

Figure S7. Leave-one-out analysis, funnel plot and MR effect size for processed meat, pork, beef and mutton intake on prostate cancer.

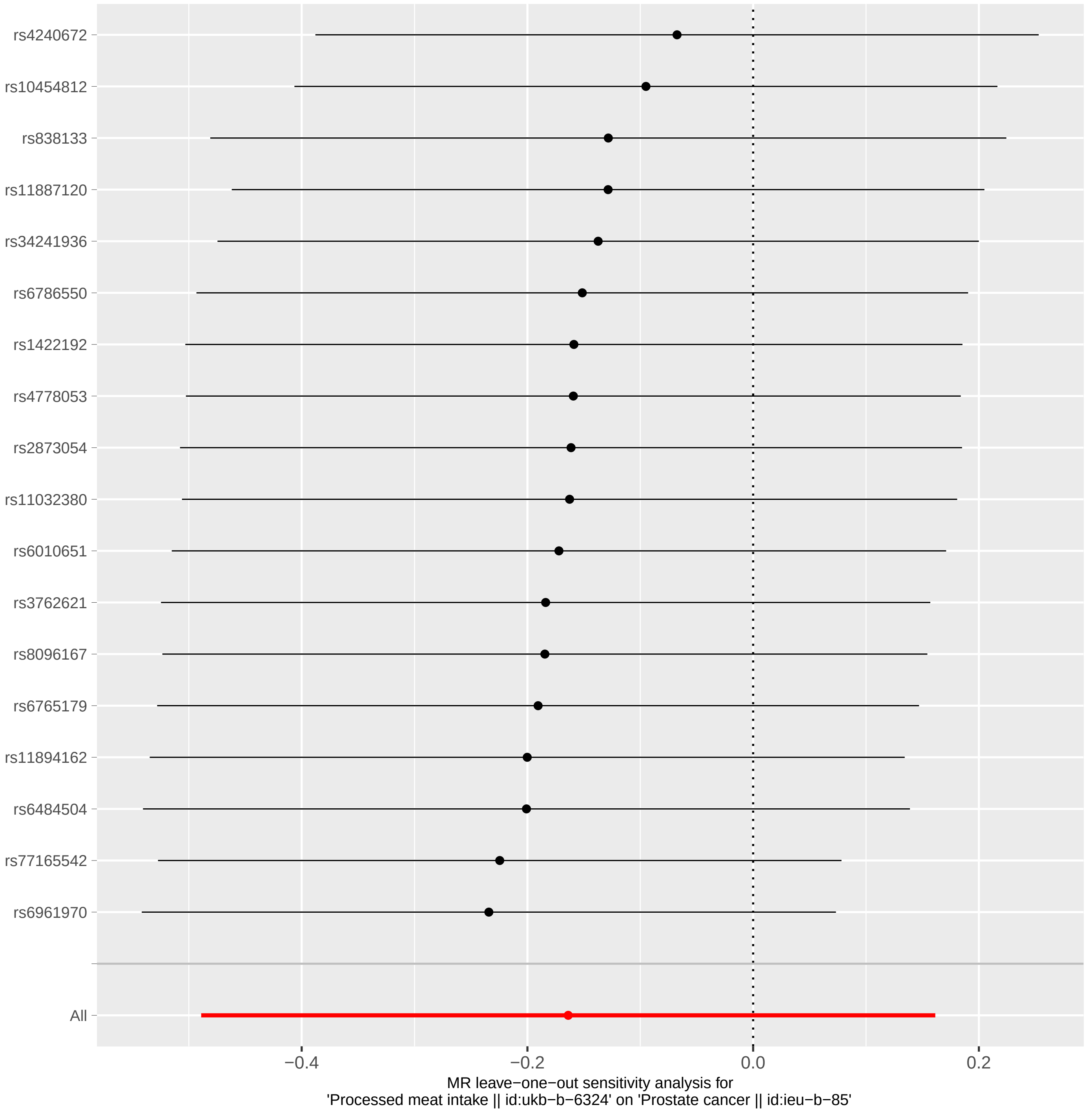

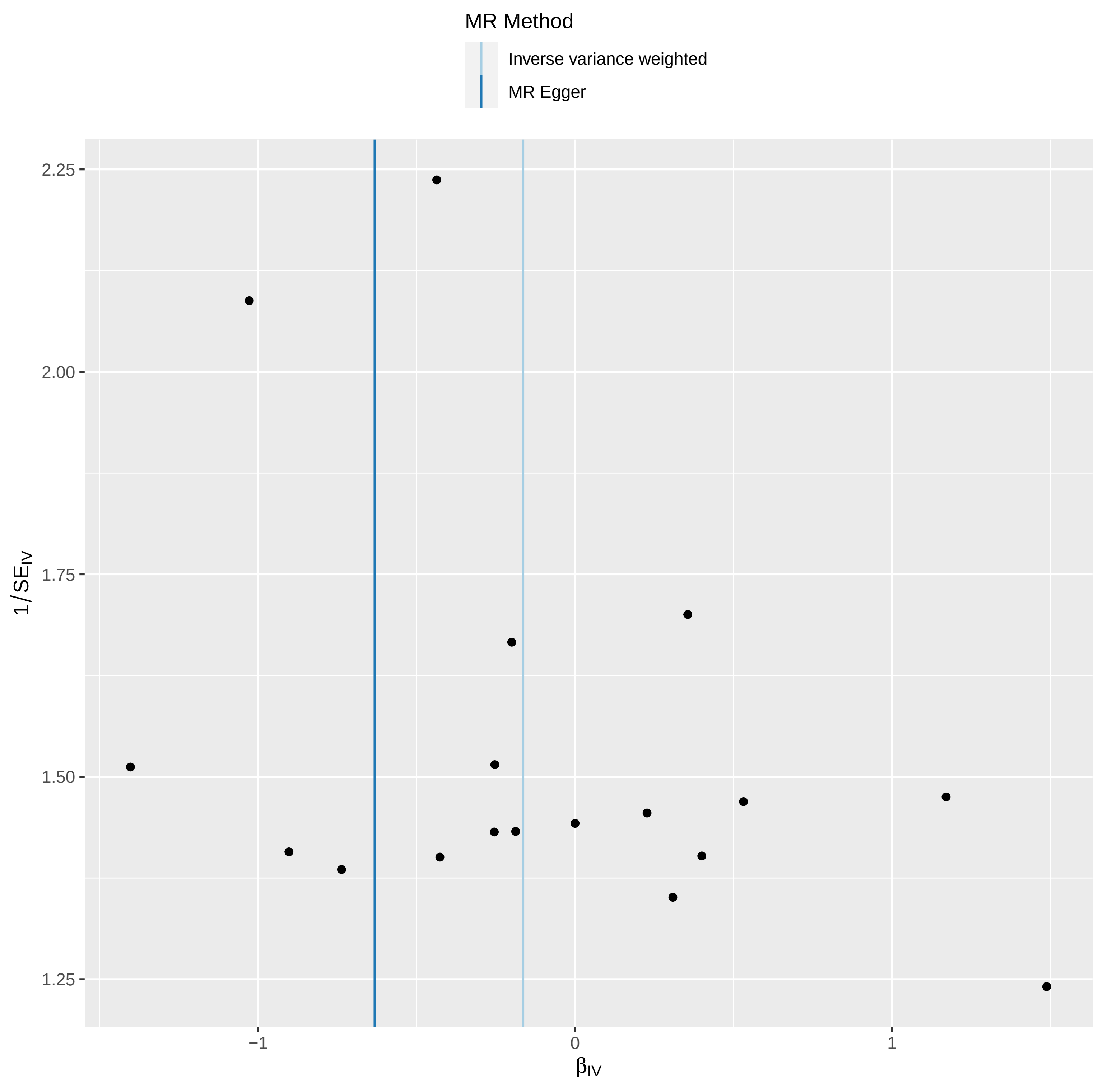
MR Method
Inverse variance weighted
MR Egger
2.25
2.00
1.75
1.50
1.25
1/SE_IV
−1
0
1
β_IV

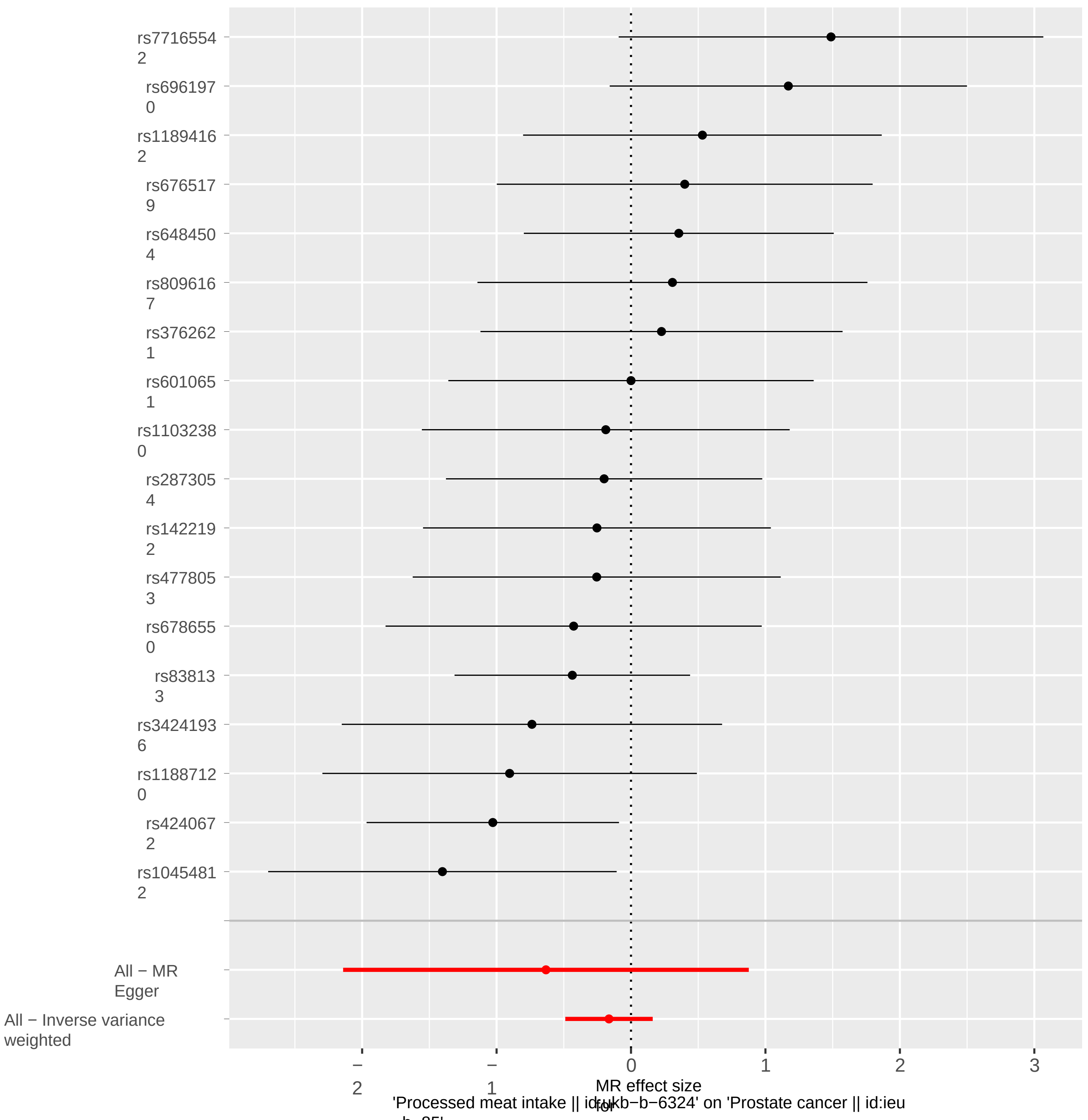

rs77165542
rs6961970
rs11894162
rs6765179
rs6484504
rs8096167
rs3762621
rs6010651
rs11032380
rs2873054
rs1422192
rs4778053
rs6786550
rs838133
rs34241936
rs11887120
rs4240672
rs10454812
All − MR Egger
All − Inverse variance weighted
−2
−1
0
1
2
3
MR effect size for
'Processed meat intake || id:ukb−b−6324' on 'Prostate cancer || id:ieu

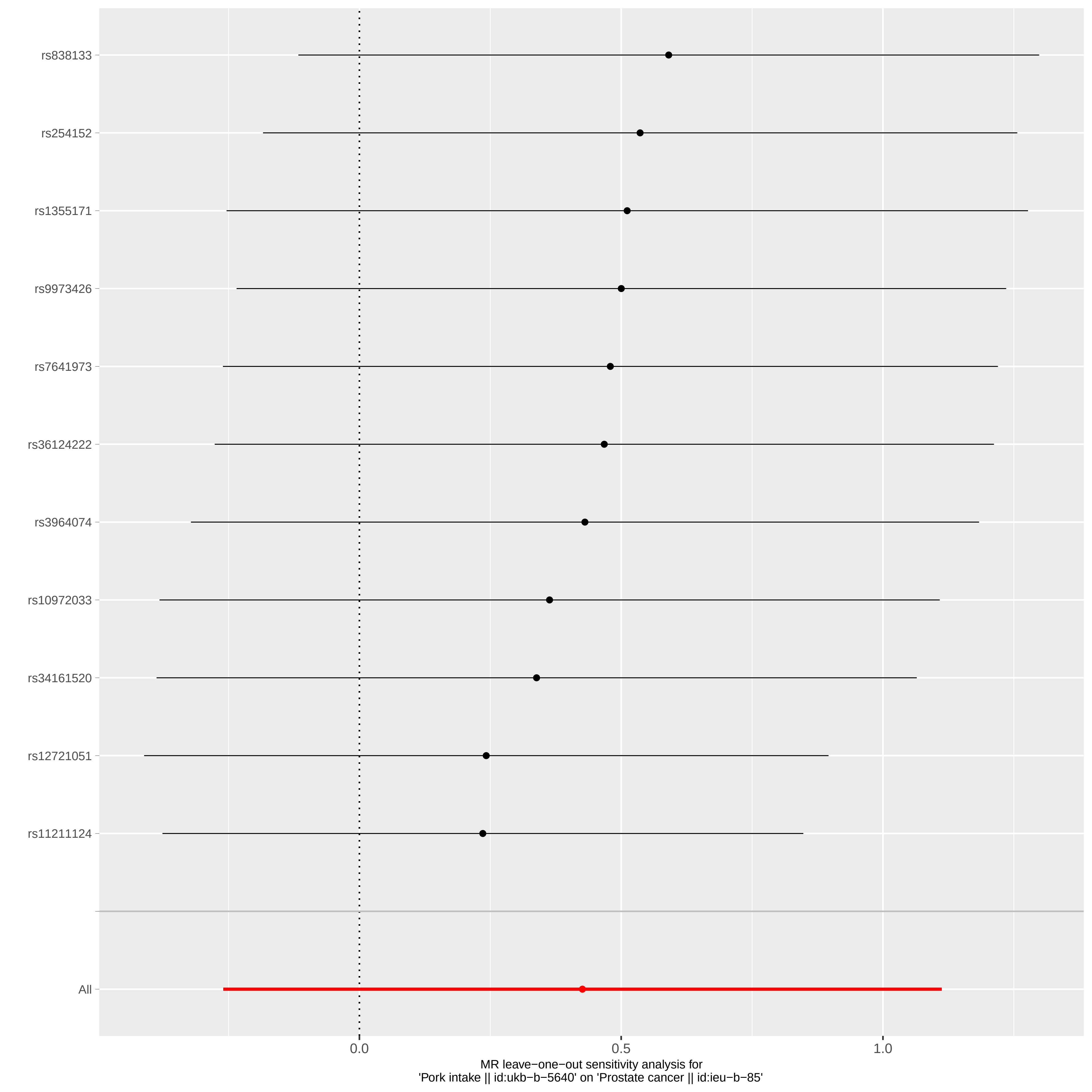

rs838133
rs254152
rs1355171
rs9973426
rs7641973
rs36124222
rs3964074
rs10972033
rs34161520
rs12721051
rs11211124
All
0.0
0.5
1.0
MR leave−one−out sensitivity analysis for
'Pork intake || id:ukb−b−5640' on 'Prostate cancer || id:ieu−b−85'

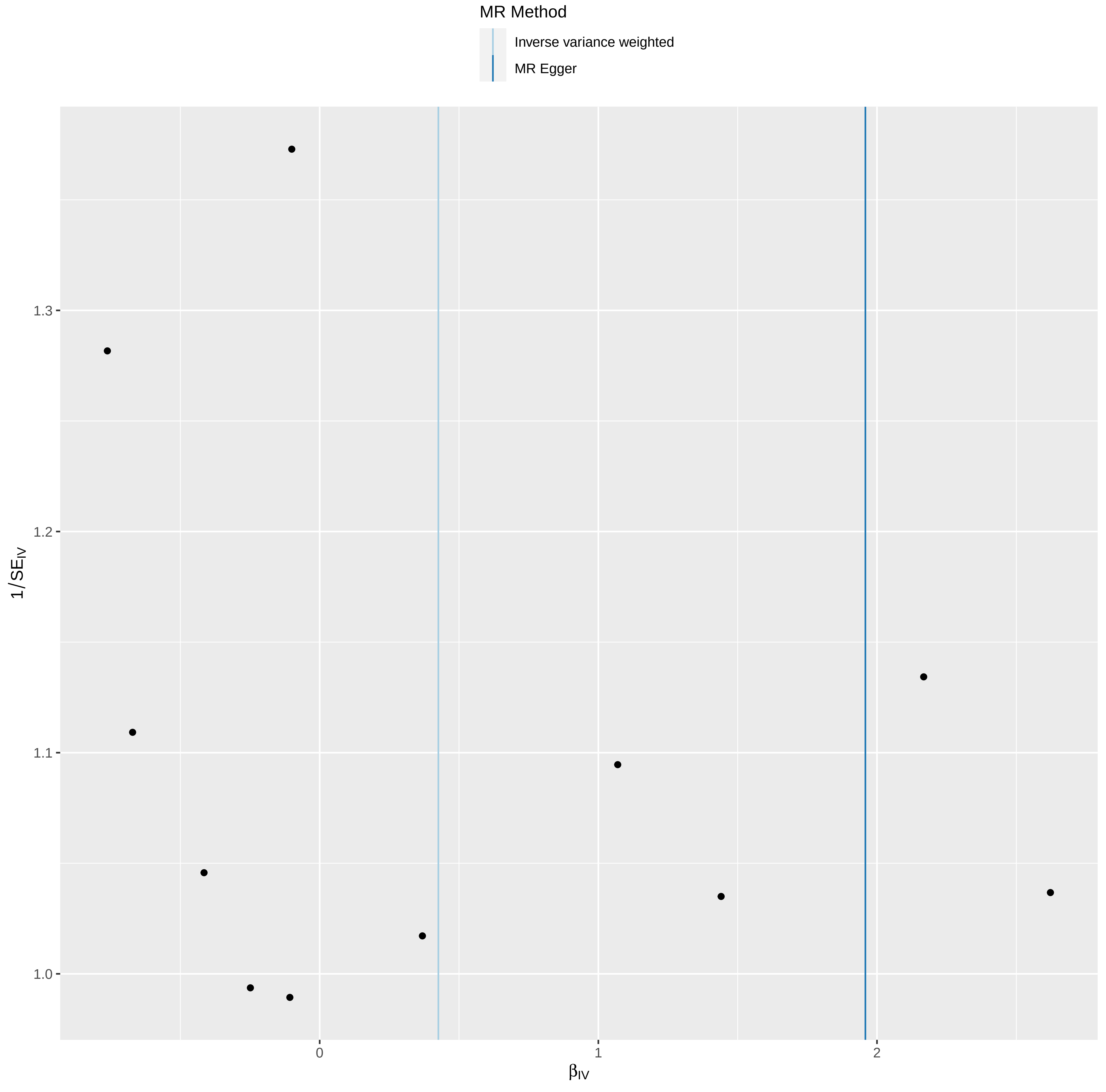

MR Method
Inverse variance weighted
MR Egger
1.3
1.2
1.1
1.0
1/SE_IV
0
1
2
β_IV

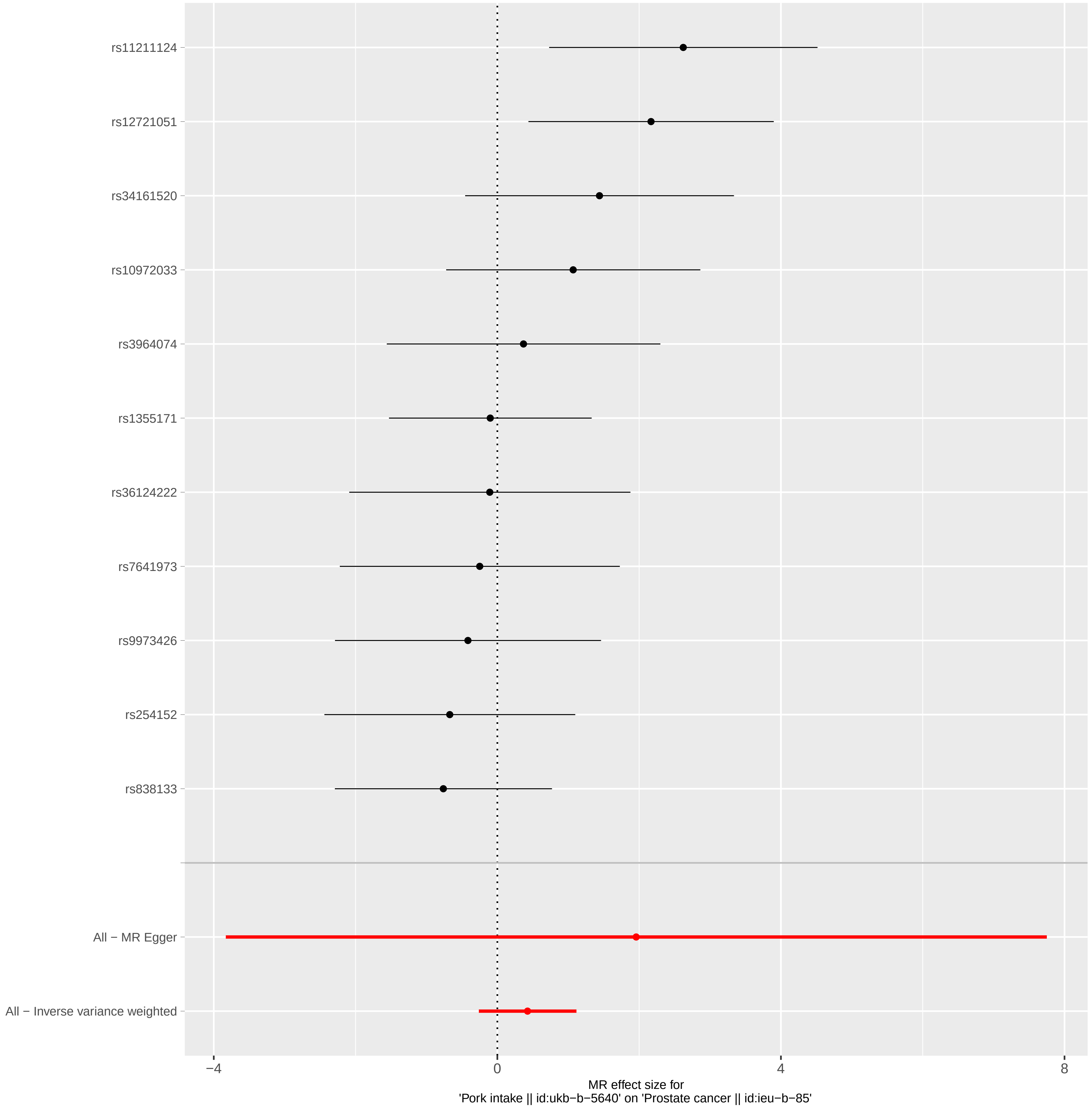
rs11211124
rs12721051
rs34161520
rs10972033
rs3964074
rs1355171
rs36124222
rs7641973
rs9973426
rs254152
rs838133
All − MR Egger
All − Inverse variance weighted
−4
0
4
8
MR effect size for
'Pork intake || id:ukb−b−5640' on 'Prostate cancer || id:ieu−b−85'

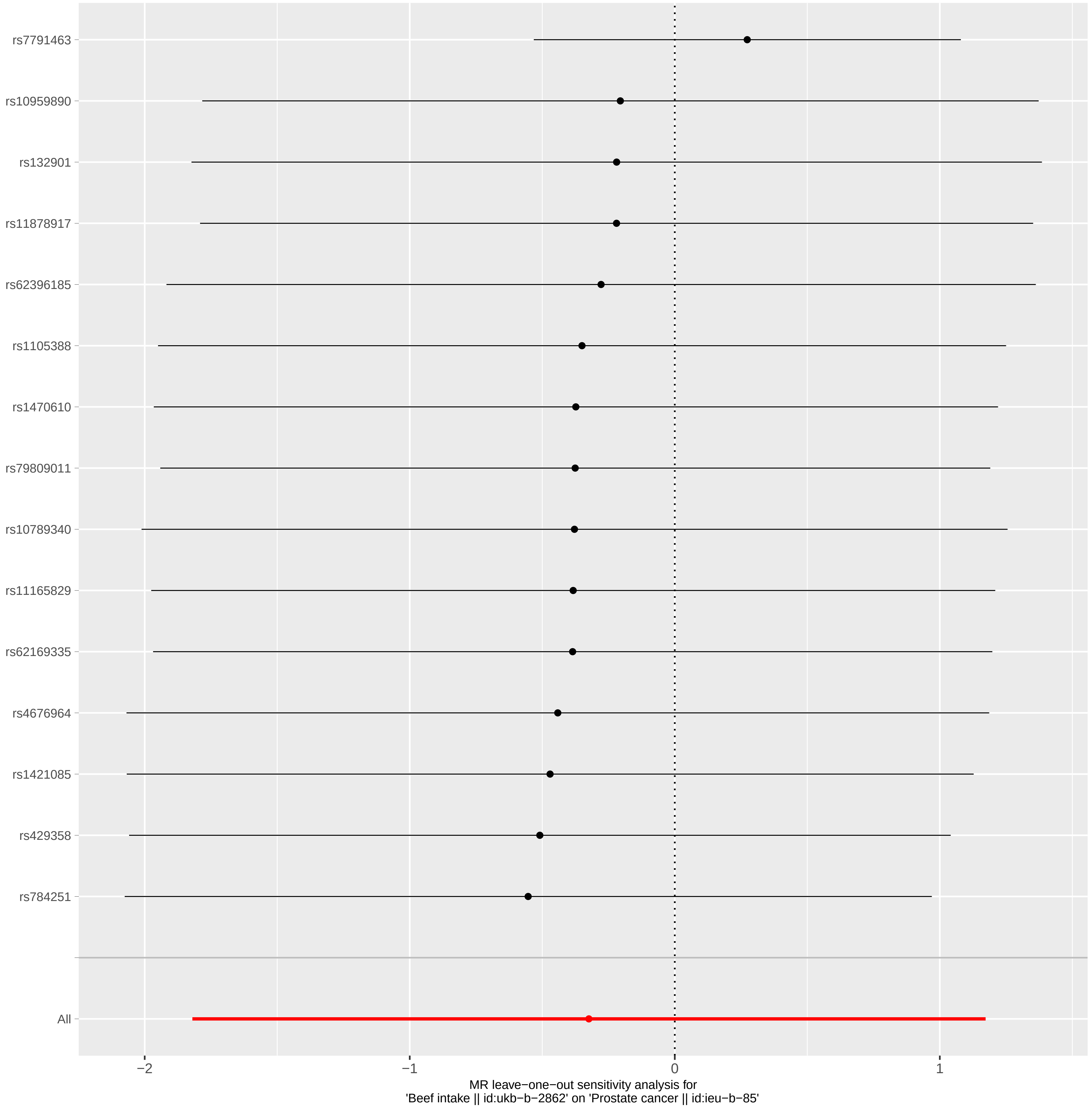
rs7791463
rs10959890
rs132901
rs11878917
rs62396185
rs1105388
rs1470610
rs79809011
rs10789340
rs11165829
rs62169335
rs4676964
rs1421085
rs429358
rs784251
All
−2
−1
0
1
MR leave−one−out sensitivity analysis for
'Beef intake || id:ukb−b−2862' on 'Prostate cancer || id:ieu−b−85'

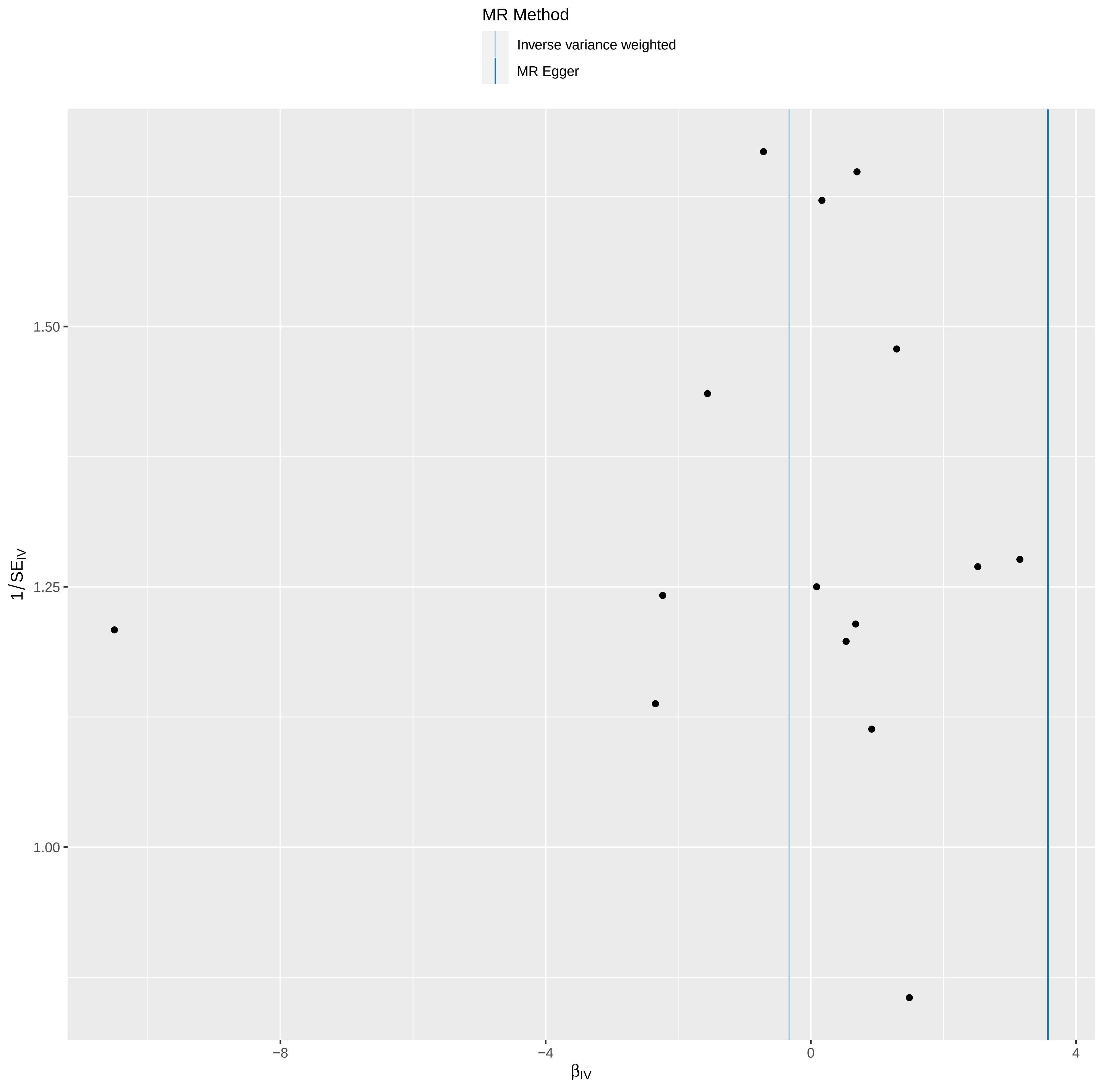

MR Method
Inverse variance weighted
MR Egger
1.50
1.25
1.00
1/SE$_{IV}$
−8
−4
0
4
β$_{IV}$

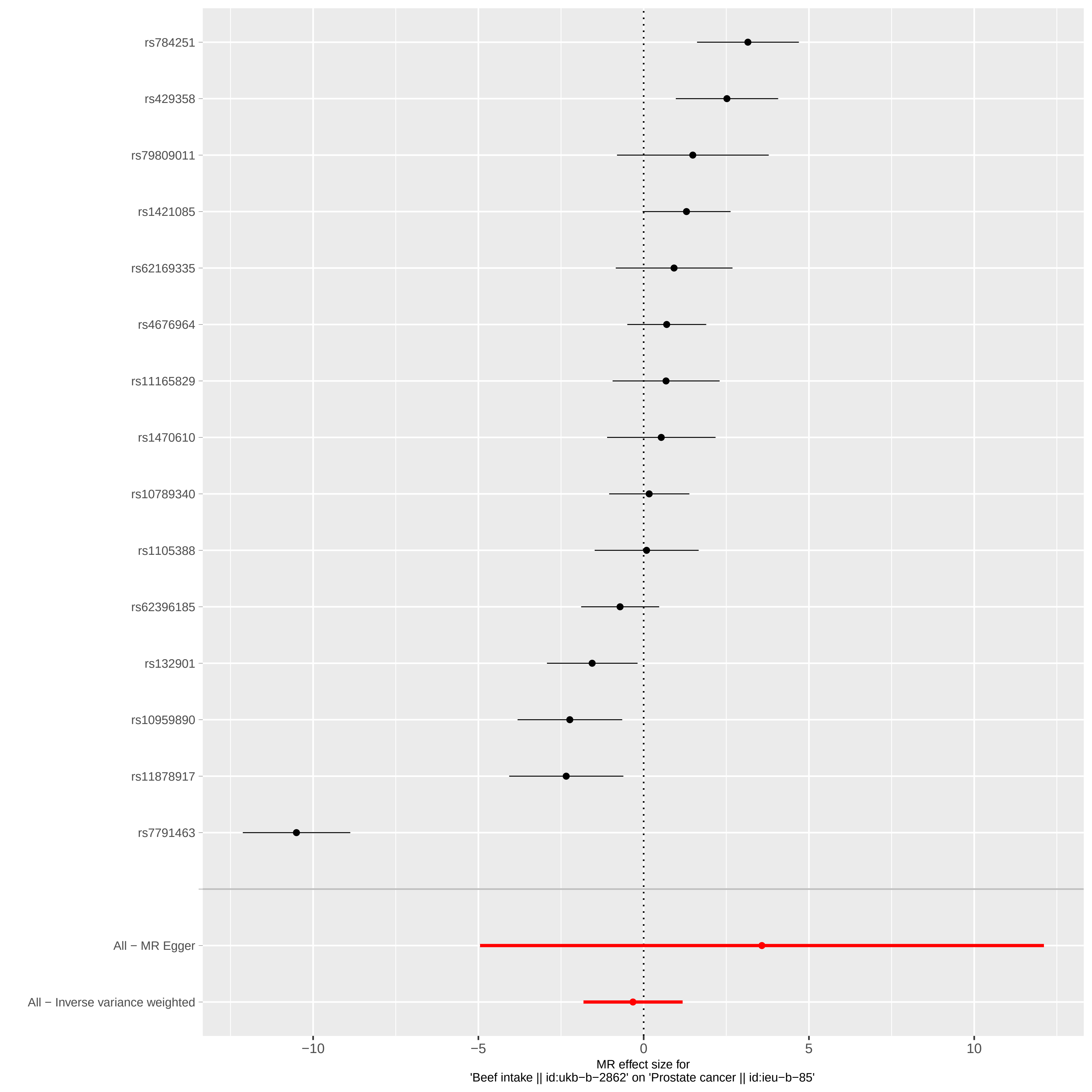
rs784251
rs429358
rs79809011
rs1421085
rs62169335
rs4676964
rs11165829
rs1470610
rs10789340
rs1105388
rs62396185
rs132901
rs10959890
rs11878917
rs7791463
All − MR Egger
All − Inverse variance weighted
−10
−5
0
5
10
MR effect size for
'Beef intake || id:ukb−b−2862' on 'Prostate cancer || id:ieu−b−85'

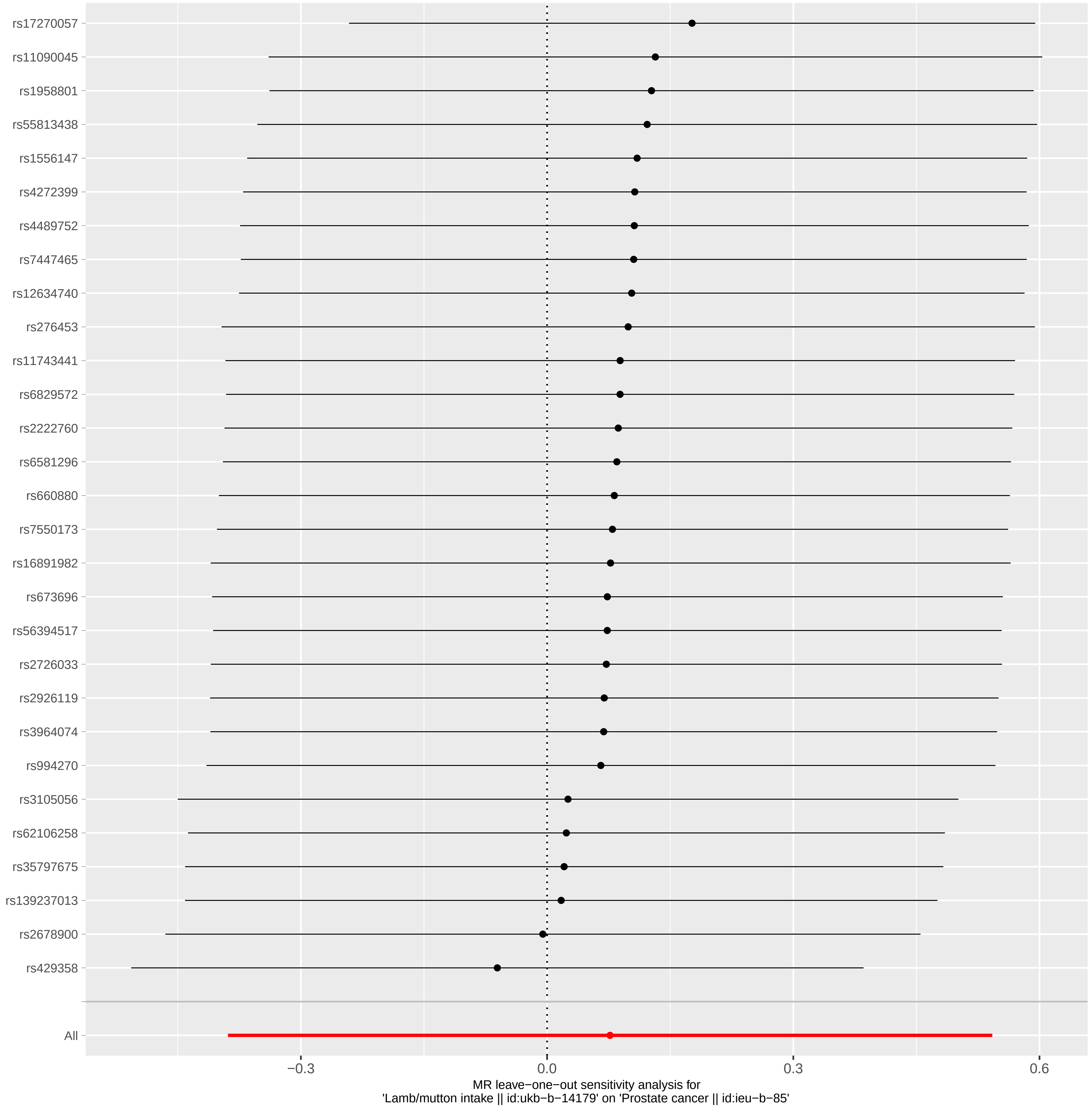
rs17270057
rs11090045
rs1958801
rs55813438
rs1556147
rs4272399
rs4489752
rs7447465
rs12634740
rs276453
rs11743441
rs6829572
rs2222760
rs6581296
rs660880
rs7550173
rs16891982
rs673696
rs56394517
rs2726033
rs2926119
rs3964074
rs994270
rs3105056
rs62106258
rs35797675
rs139237013
rs2678900
rs429358
All
−0.3
0.0
0.3
0.6
MR leave−one−out sensitivity analysis for
'Lamb/mutton intake || id:ukb−b−14179' on 'Prostate cancer || id:ieu−b−85'

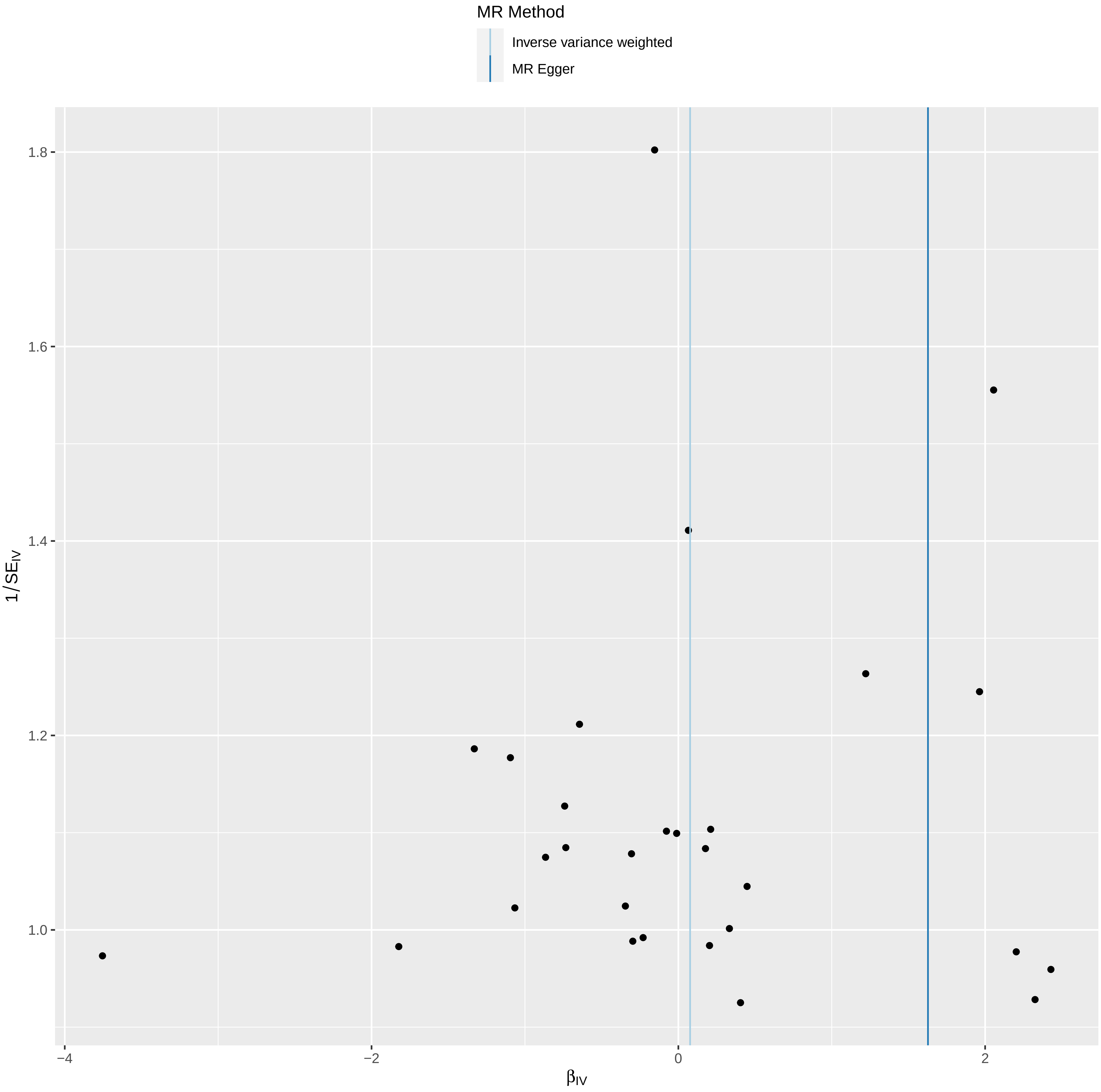

MR Method
Inverse variance weighted
MR Egger
1.8
1.6
1.4
1.2
1.0
1/SE_IV
−4
−2
0
2
β_IV

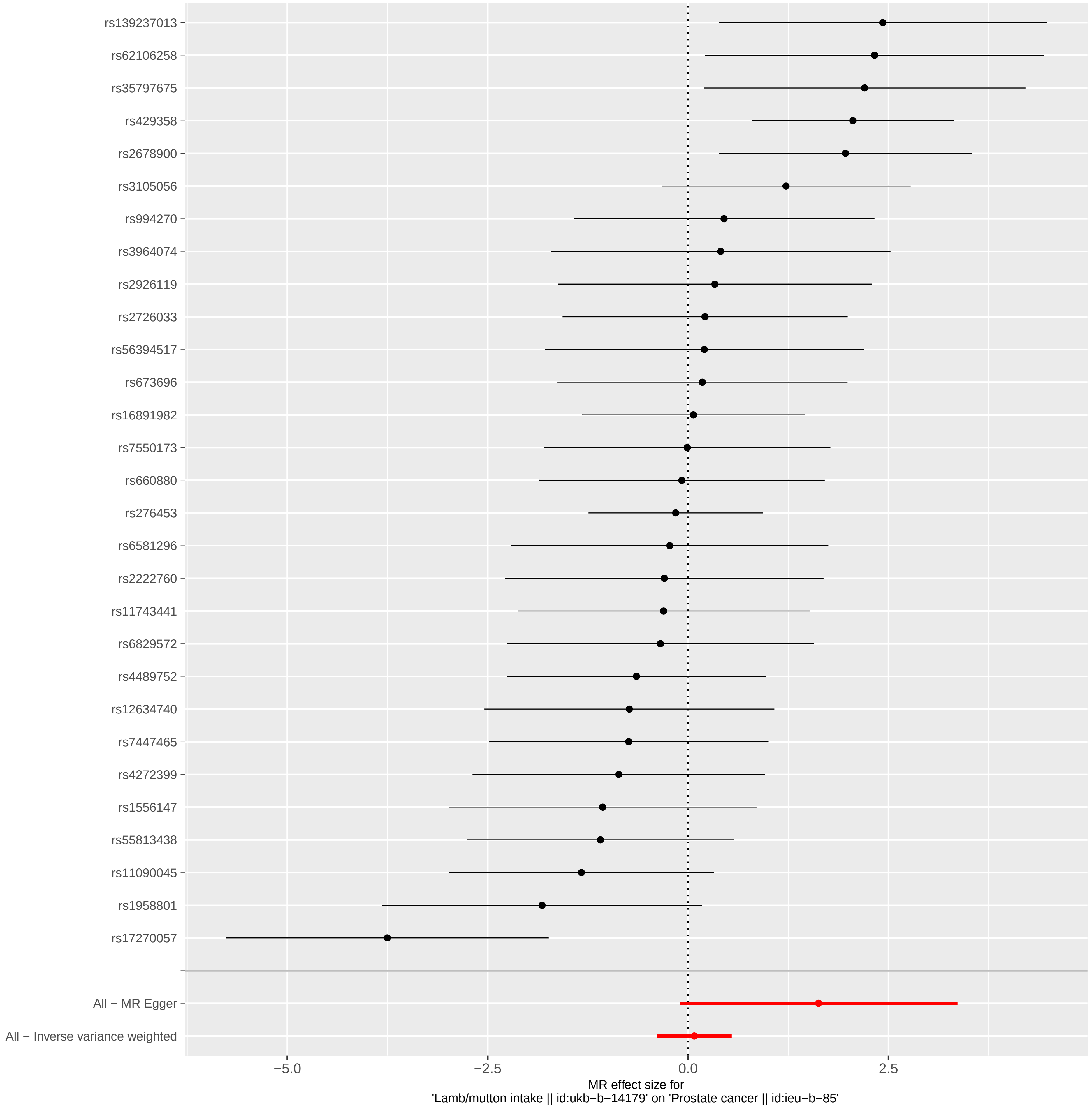
rs139237013
rs62106258
rs35797675
rs429358
rs2678900
rs3105056
rs994270
rs3964074
rs2926119
rs2726033
rs56394517
rs673696
rs16891982
rs7550173
rs660880
rs276453
rs6581296
rs2222760
rs11743441
rs6829572
rs4489752
rs12634740
rs7447465
rs4272399
rs1556147
rs55813438
rs11090045
rs1958801
rs17270057
All − MR Egger
All − Inverse variance weighted
−5.0
−2.5
0.0
2.5
MR effect size for
'Lamb/mutton intake || id:ukb−b−14179' on 'Prostate cancer || id:ieu−b−85'

Figure S8. Leave-one-out analysis, funnel plot and MR effect size for processed meat, pork, beef and mutton intake on skin malignancy.

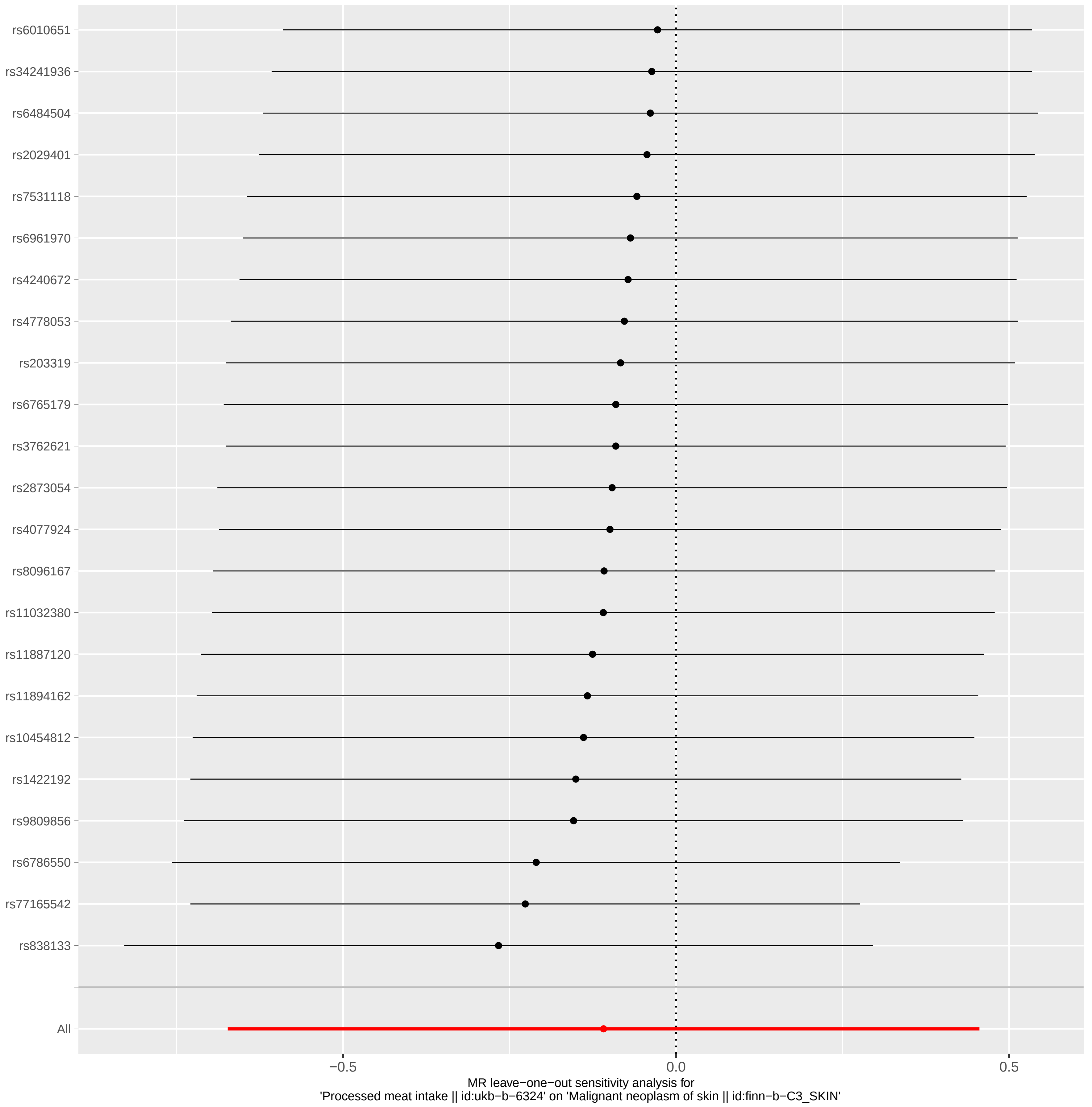

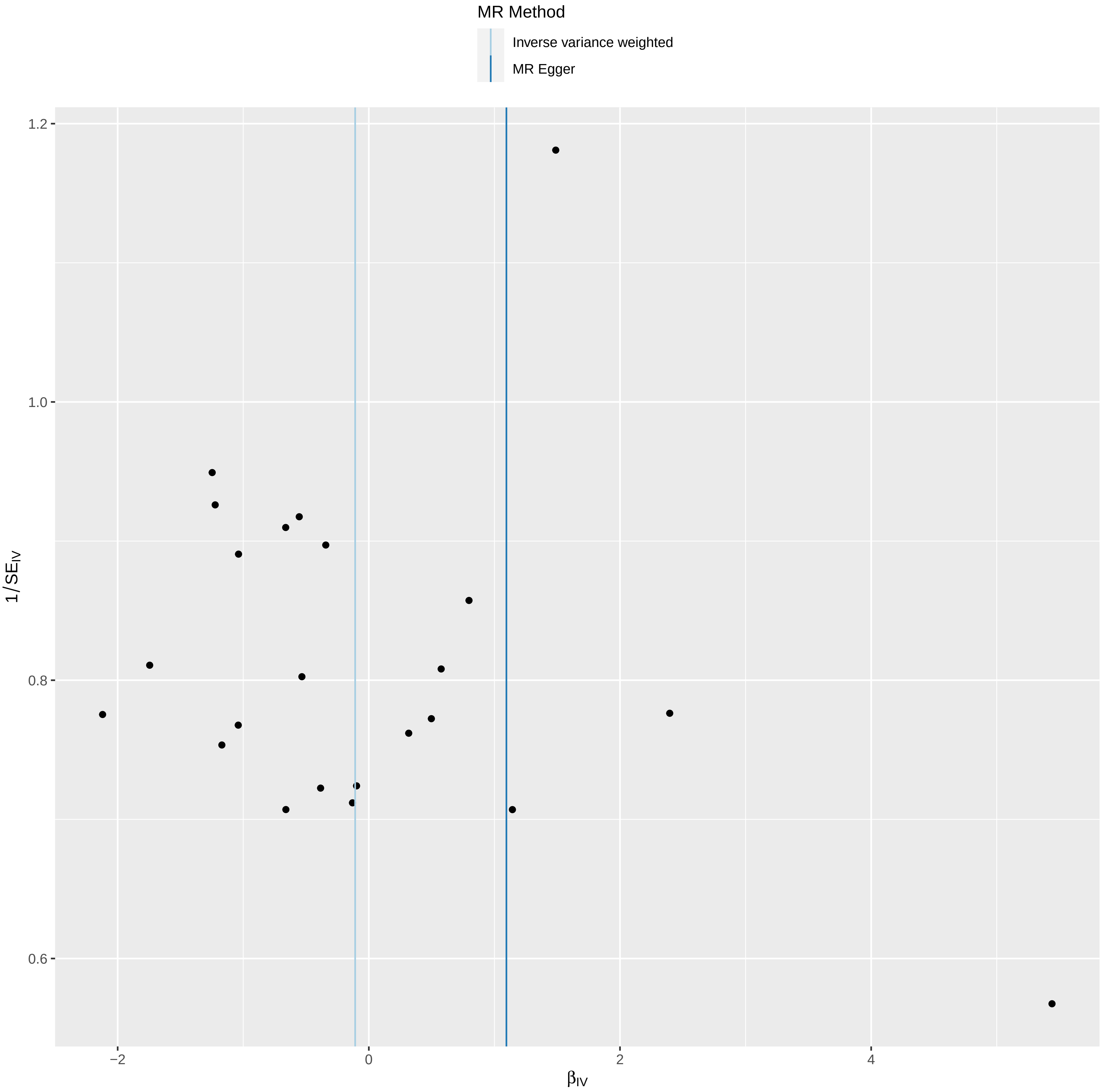

MR Method
Inverse variance weighted
MR Egger
1.2
1.0
0.8
0.6
1/SE$_{IV}$
−2
0
2
4
β$_{IV}$

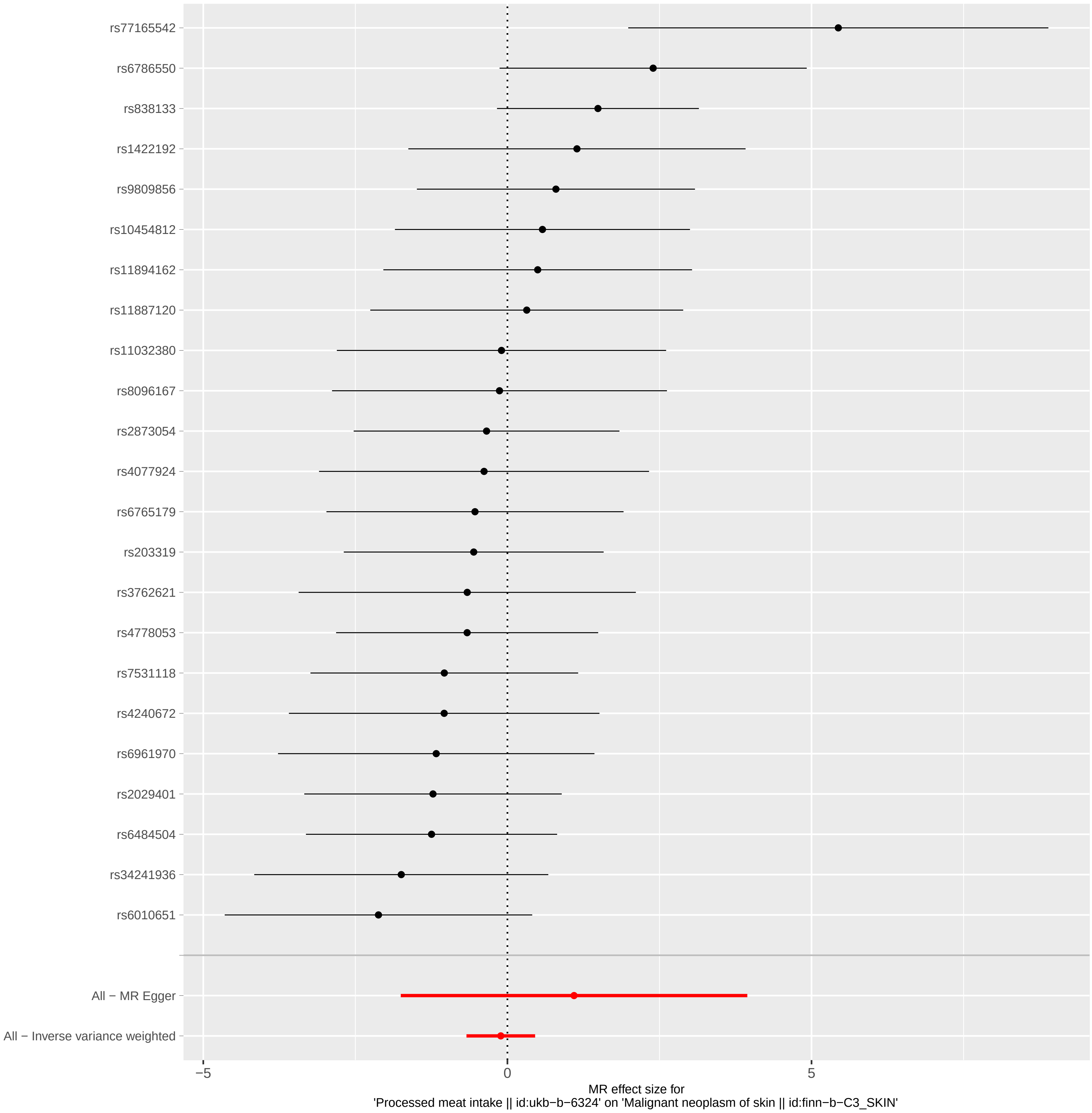

rs77165542
rs6786550
rs838133
rs1422192
rs9809856
rs10454812
rs11894162
rs11887120
rs11032380
rs8096167
rs2873054
rs4077924
rs6765179
rs203319
rs3762621
rs4778053
rs7531118
rs4240672
rs6961970
rs2029401
rs6484504
rs34241936
rs6010651
All − MR Egger
All − Inverse variance weighted
−5
0
5
MR effect size for
'Processed meat intake || id:ukb−b−6324' on 'Malignant neoplasm of skin || id:finn−b−C3_SKIN'

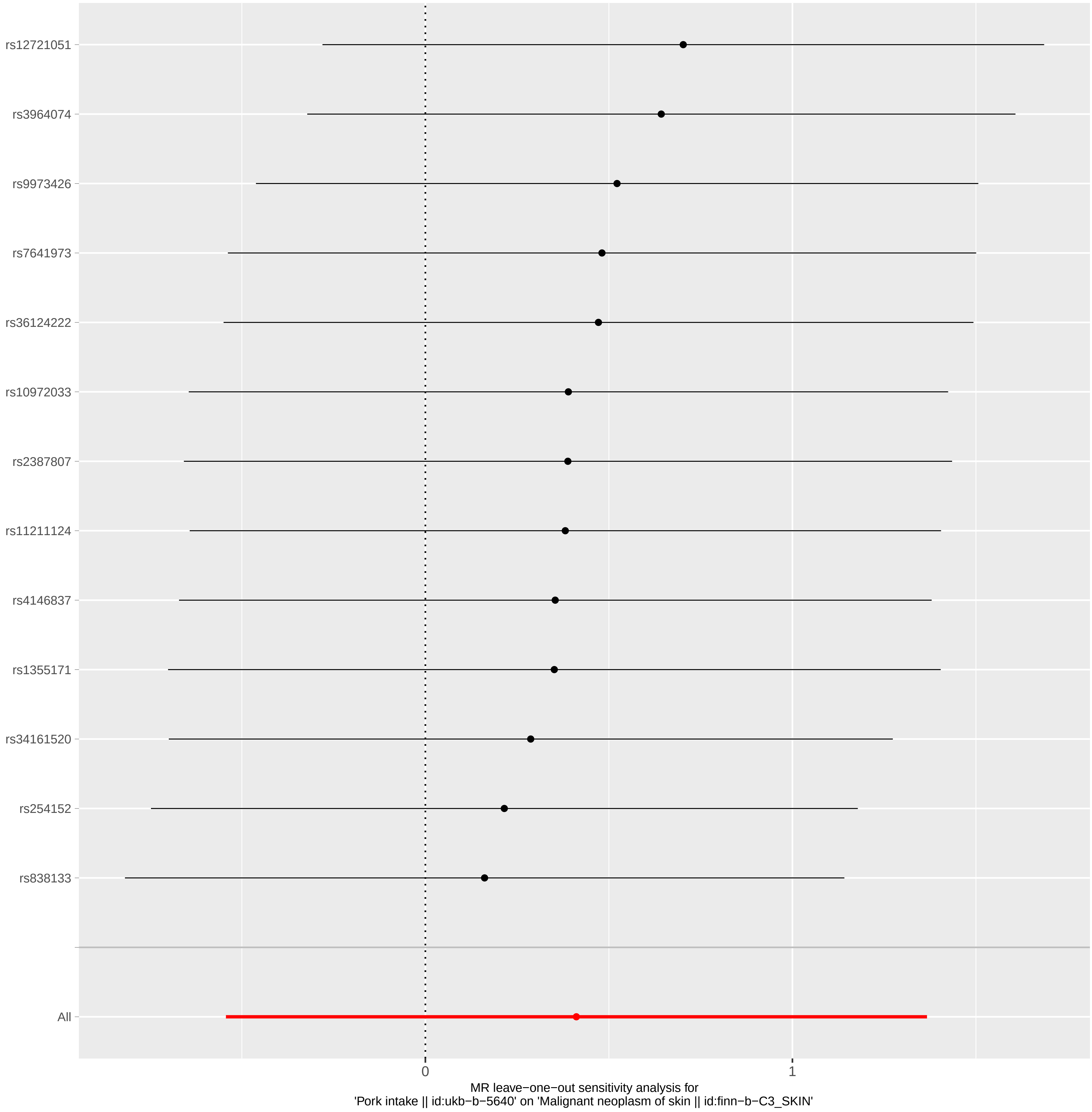

rs12721051
rs3964074
rs9973426
rs7641973
rs36124222
rs10972033
rs2387807
rs11211124
rs4146837
rs1355171
rs34161520
rs254152
rs838133
All
0
1
MR leave−one−out sensitivity analysis for
'Pork intake || id:ukb−b−5640' on 'Malignant neoplasm of skin || id:finn−b−C3_SKIN'

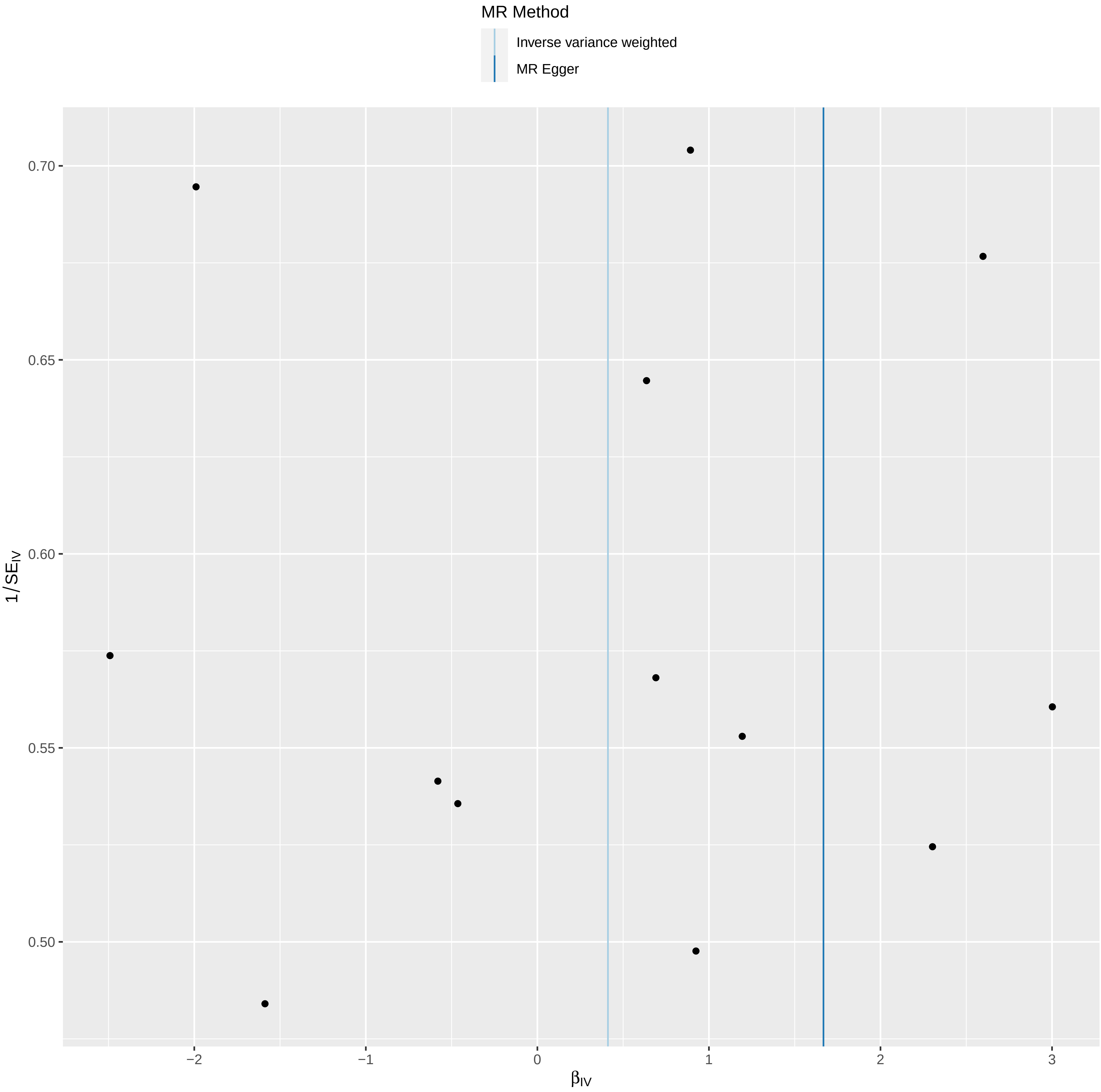
MR Method
Inverse variance weighted
MR Egger
0.70
0.65
0.60
0.55
0.50
1/SE$_{IV}$
−2
−1
0
1
2
3
β$_{IV}$

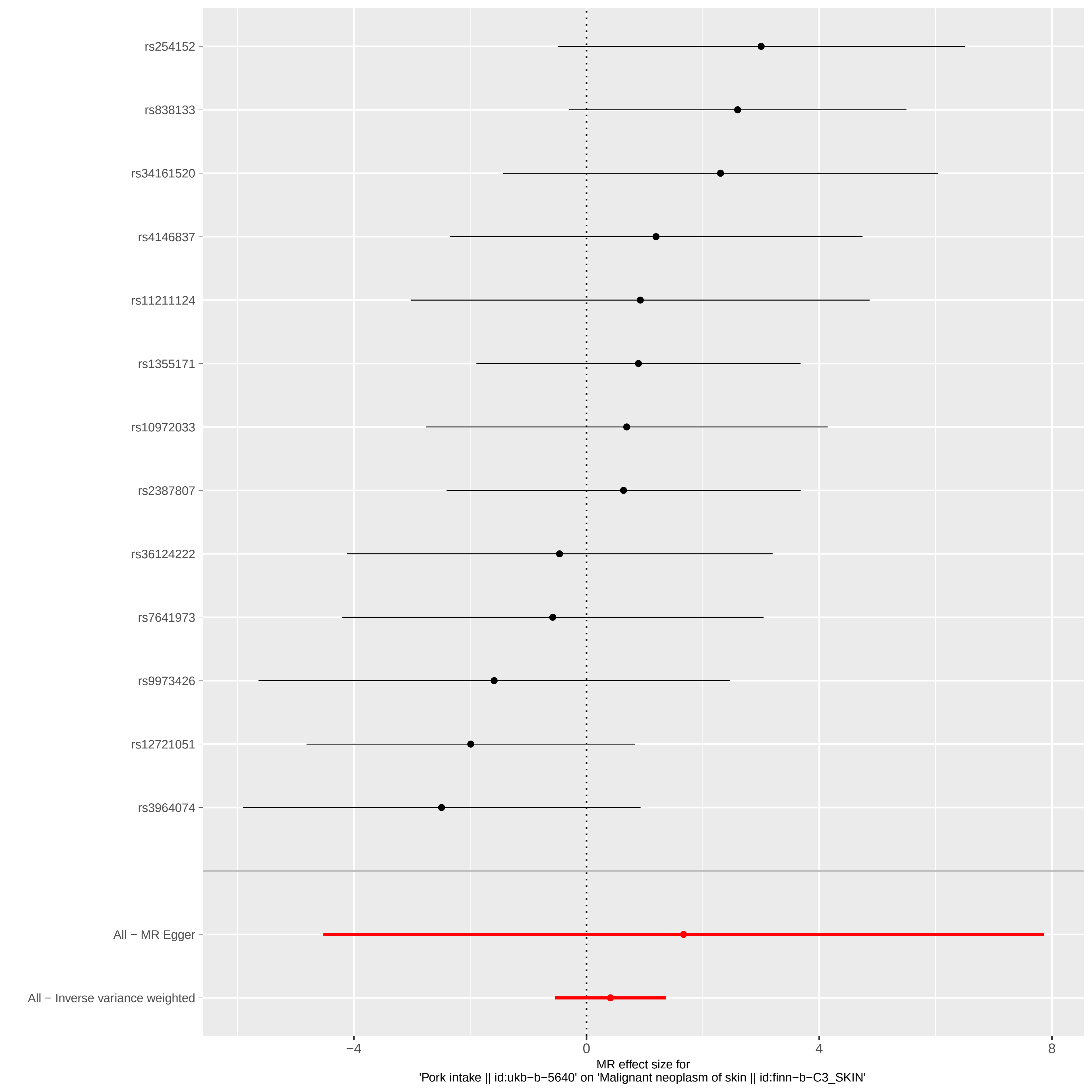
rs254152
rs838133
rs34161520
rs4146837
rs11211124
rs1355171
rs10972033
rs2387807
rs36124222
rs7641973
rs9973426
rs12721051
rs3964074
All − MR Egger
All − Inverse variance weighted
−4
0
4
8
MR effect size for
'Pork intake || id:ukb−b−5640' on 'Malignant neoplasm of skin || id:finn−b−C3_SKIN'

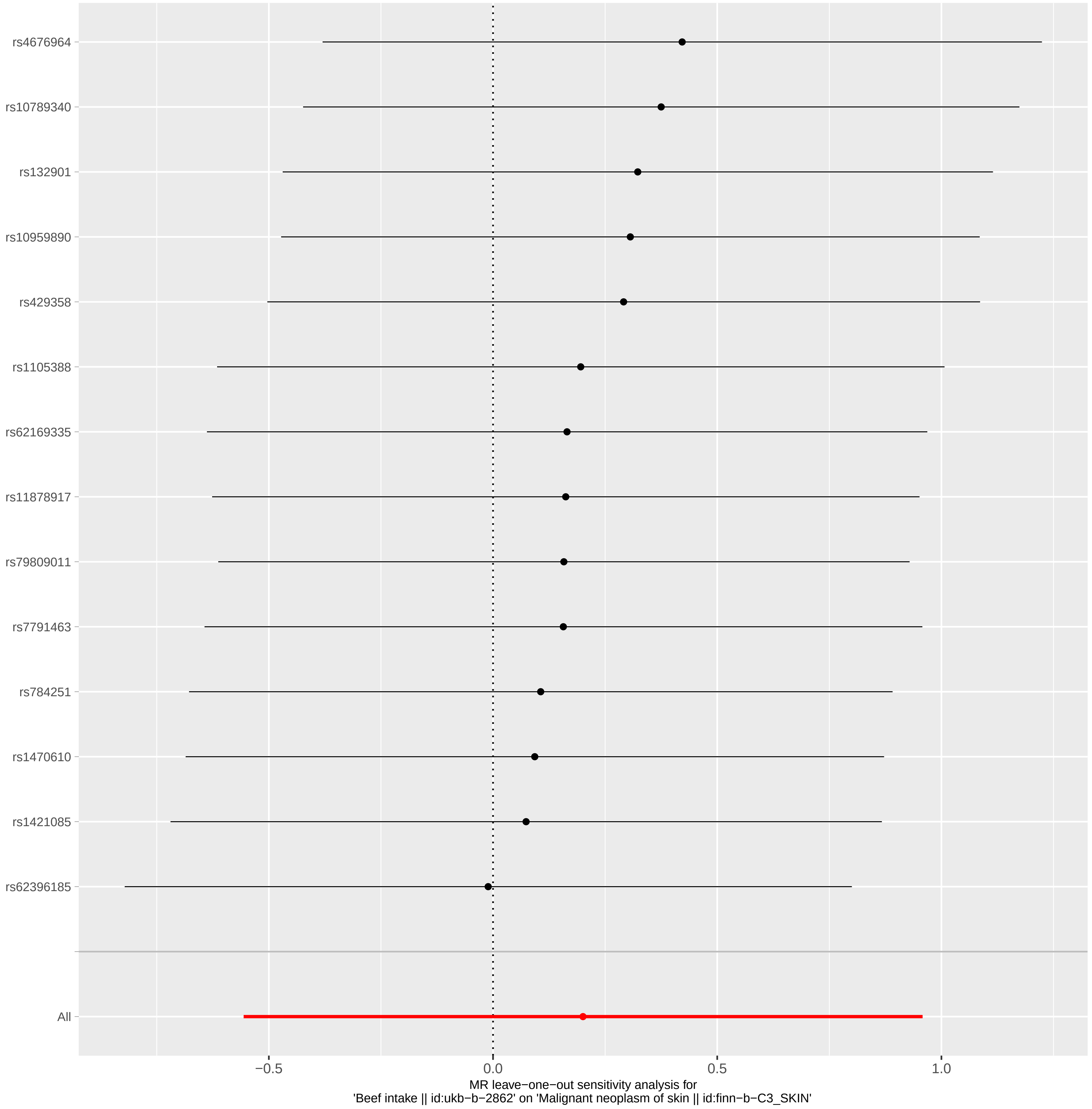
rs4676964
rs10789340
rs132901
rs10959890
rs429358
rs1105388
rs62169335
rs11878917
rs79809011
rs7791463
rs784251
rs1470610
rs1421085
rs62396185
All
−0.5
0.0
0.5
1.0
MR leave−one−out sensitivity analysis for
'Beef intake || id:ukb−b−2862' on 'Malignant neoplasm of skin || id:finn−b−C3_SKIN'

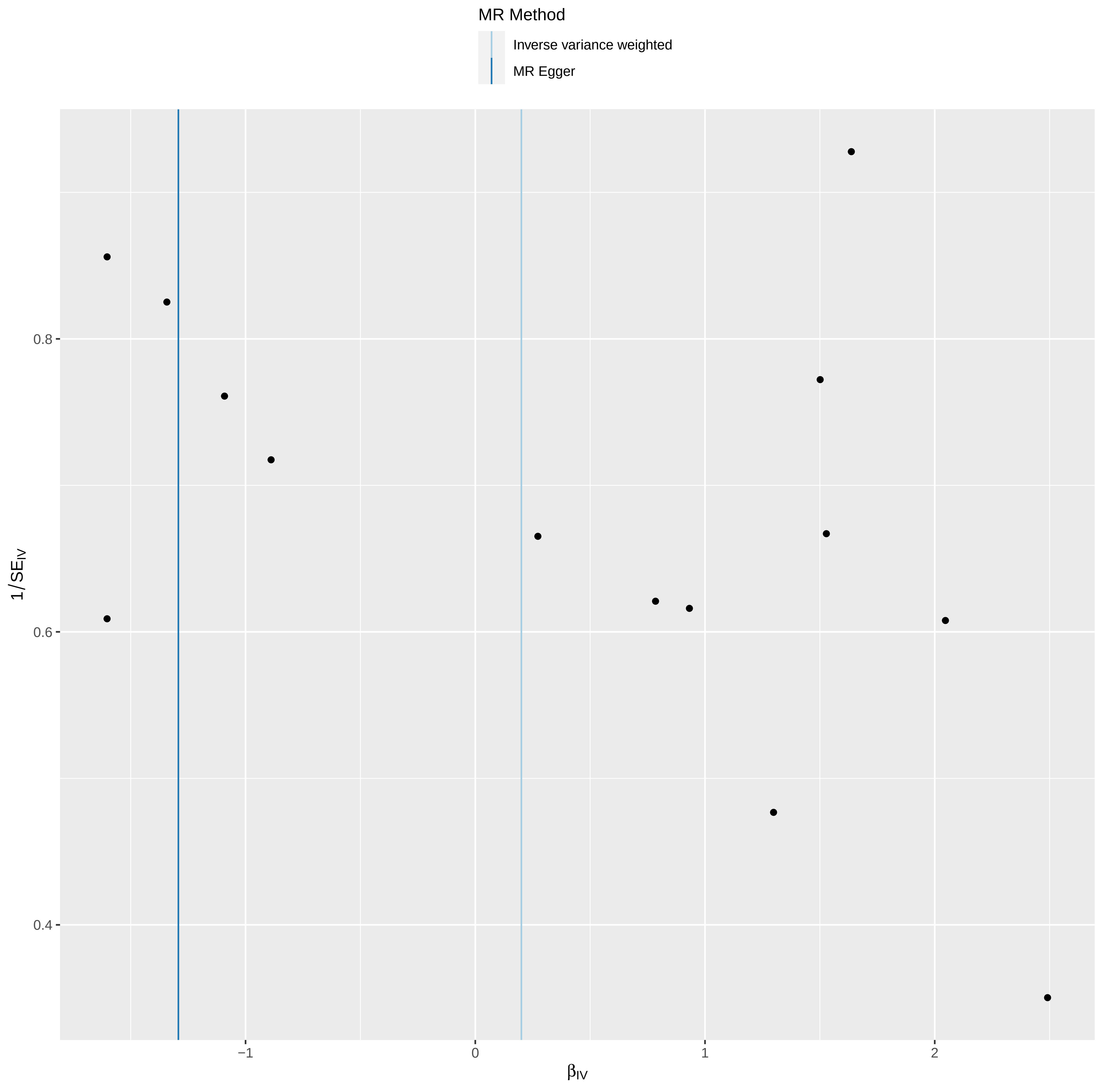
MR Method
Inverse variance weighted
MR Egger
0.4
0.6
0.8
1/SE$_{IV}$
−1
0
1
2
β$_{IV}$

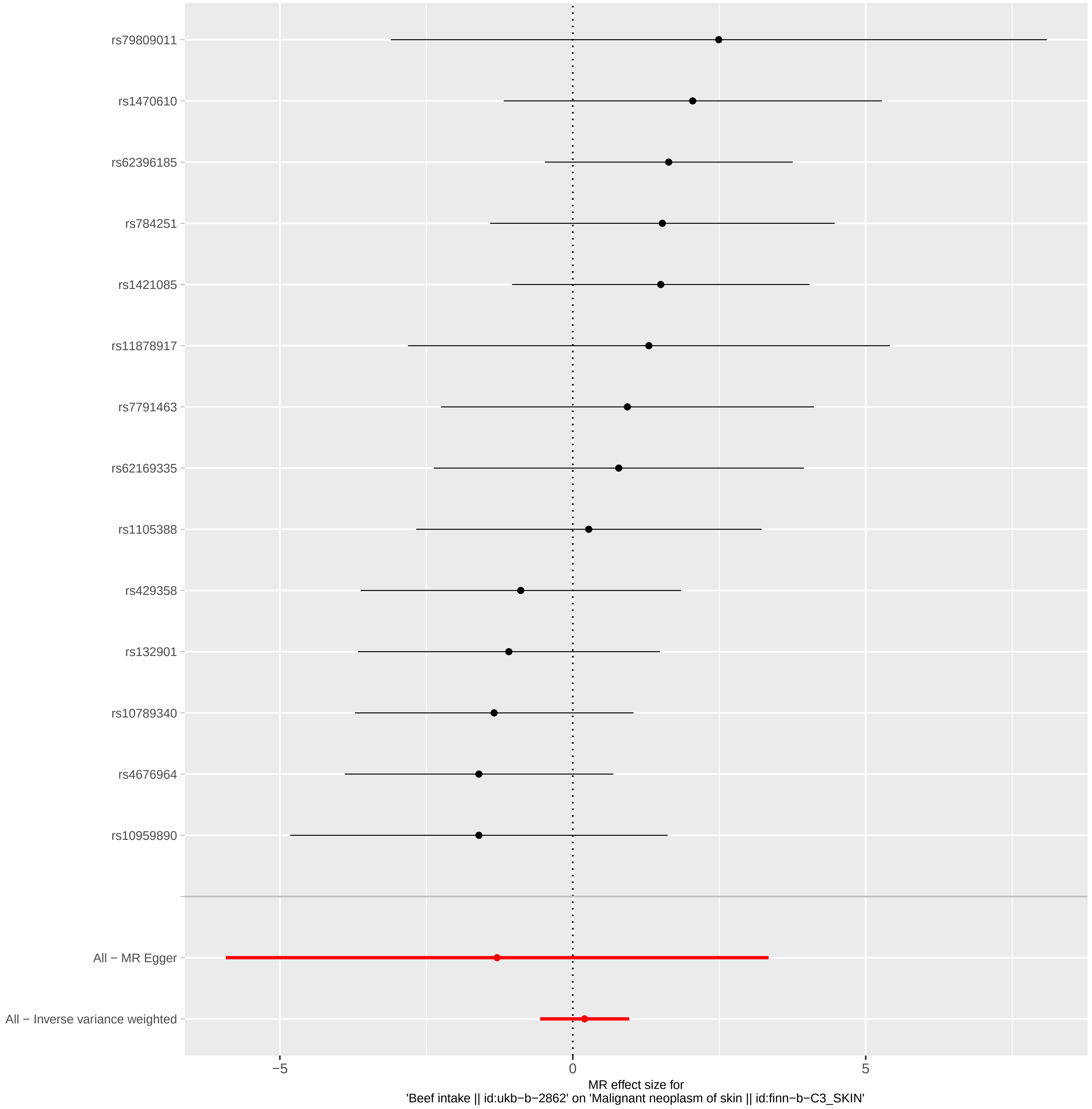

rs79809011
rs1470610
rs62396185
rs784251
rs1421085
rs11878917
rs7791463
rs62169335
rs1105388
rs429358
rs132901
rs10789340
rs4676964
rs10959890
All − MR Egger
All − Inverse variance weighted
−5
0
5
MR effect size for
'Beef intake || id:ukb−b−2862' on 'Malignant neoplasm of skin || id:finn−b−C3_SKIN'

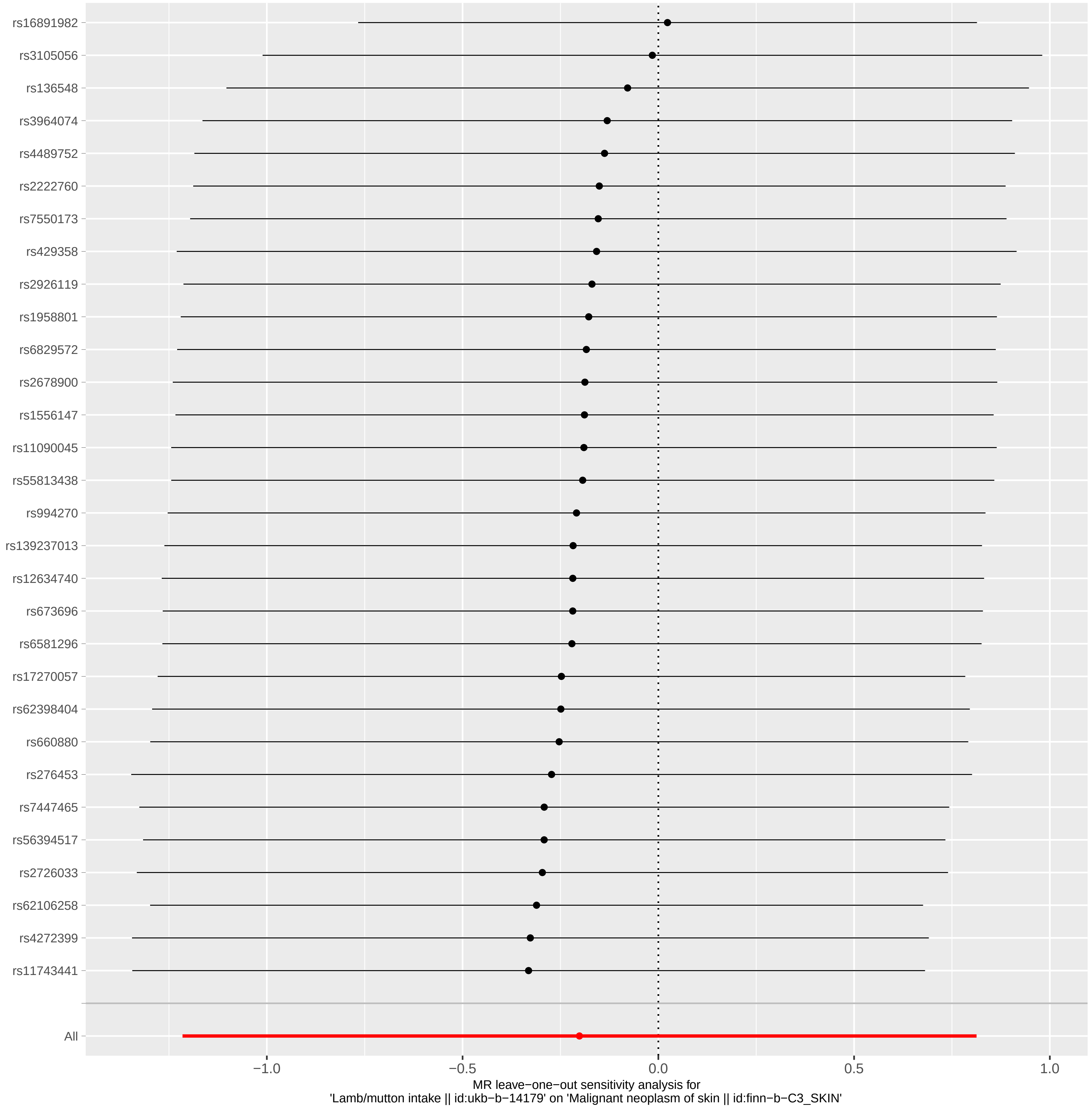
rs16891982
rs3105056
rs136548
rs3964074
rs4489752
rs2222760
rs7550173
rs429358
rs2926119
rs1958801
rs6829572
rs2678900
rs1556147
rs11090045
rs55813438
rs994270
rs139237013
rs12634740
rs673696
rs6581296
rs17270057
rs62398404
rs660880
rs276453
rs7447465
rs56394517
rs2726033
rs62106258
rs4272399
rs11743441
All
−1.0
−0.5
0.0
0.5
1.0
MR leave−one−out sensitivity analysis for
'Lamb/mutton intake || id:ukb−b−14179' on 'Malignant neoplasm of skin || id:finn−b−C3_SKIN'

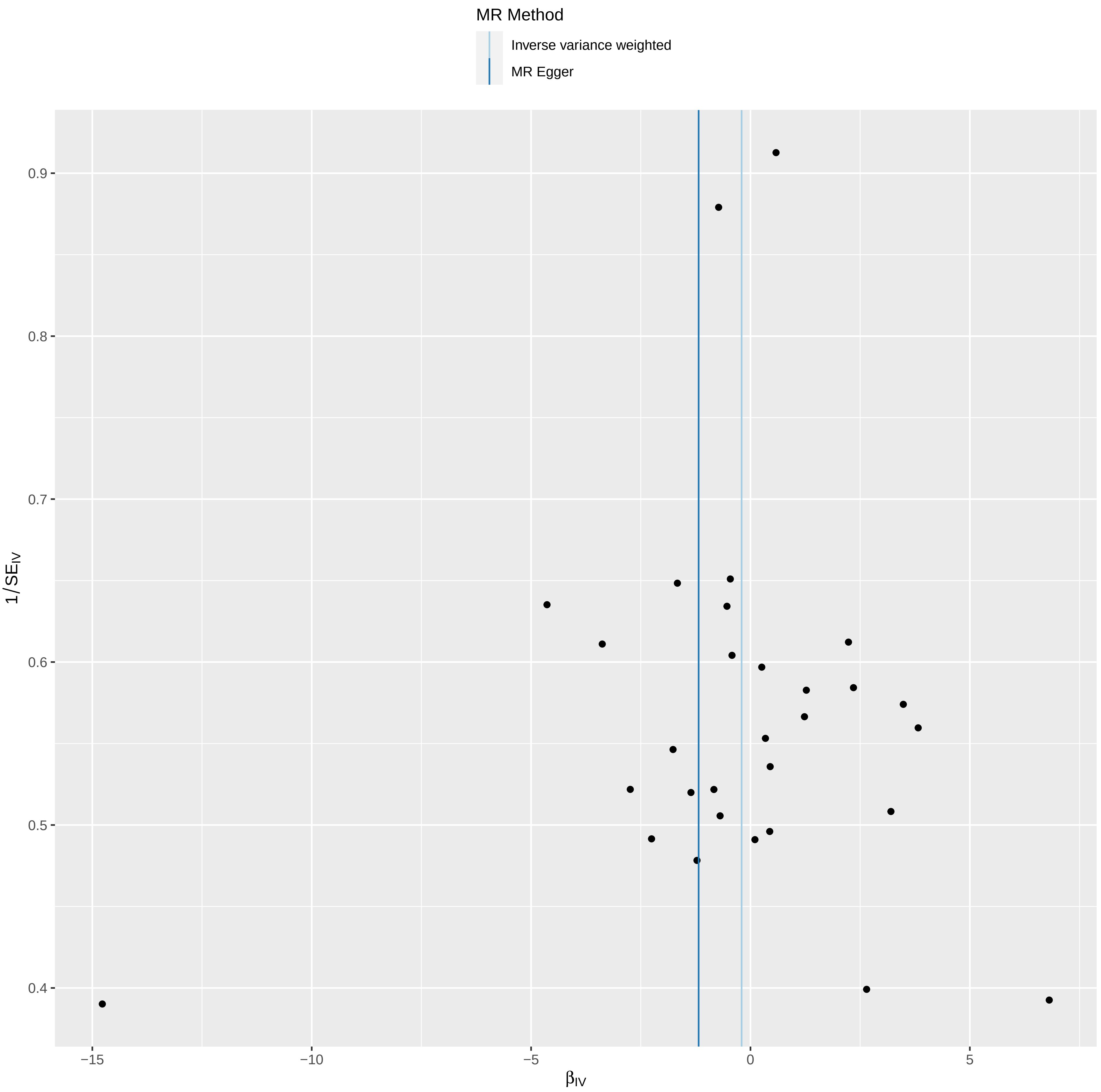
MR Method
Inverse variance weighted
MR Egger
0.9
0.8
0.7
0.6
0.5
0.4
1/SE_IV
−15
−10
−5
0
5
β_IV

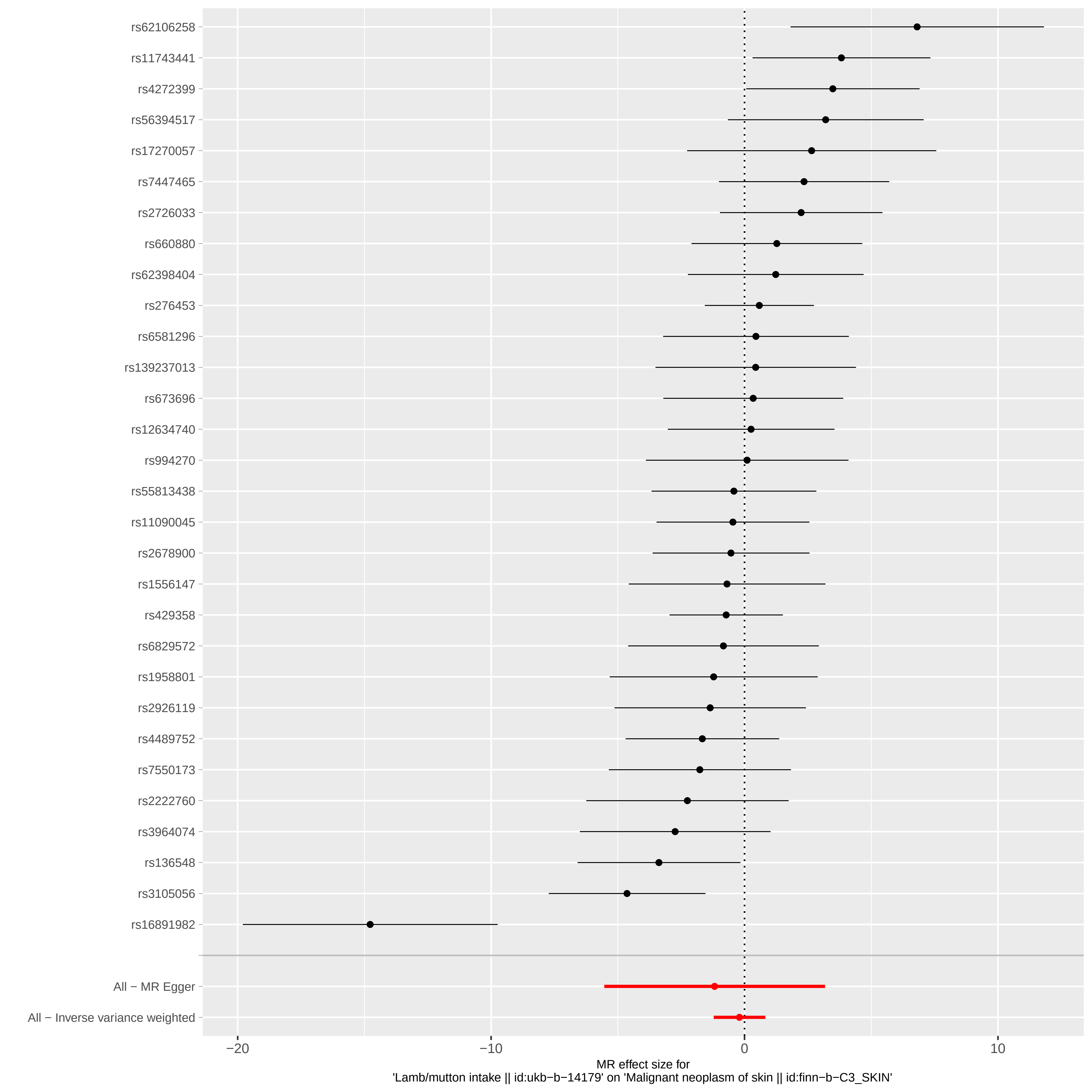

rs62106258
rs11743441
rs4272399
rs56394517
rs17270057
rs7447465
rs2726033
rs660880
rs62398404
rs276453
rs6581296
rs139237013
rs673696
rs12634740
rs994270
rs55813438
rs11090045
rs2678900
rs1556147
rs429358
rs6829572
rs1958801
rs2926119
rs4489752
rs7550173
rs2222760
rs3964074
rs136548
rs3105056
rs16891982
All − MR Egger
All − Inverse variance weighted
−20
−10
0
10
MR effect size for
'Lamb/mutton intake || id:ukb−b−14179' on 'Malignant neoplasm of skin || id:finn−b−C3_SKIN'

Figure S9. Leave-one-out analysis, funnel plot and MR effect size for processed meat, pork, beef and mutton intake on oropharyngeal cancer.

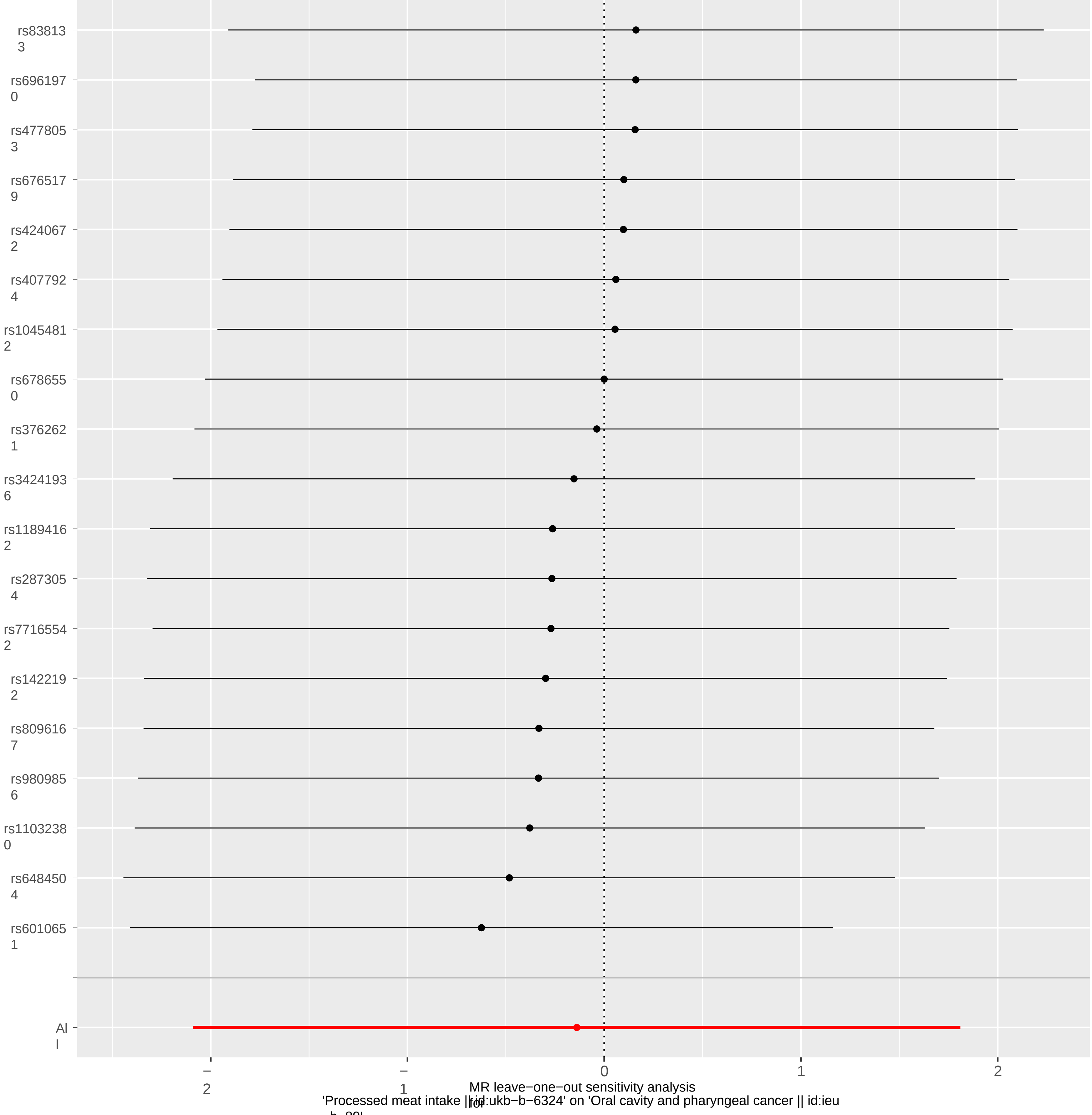

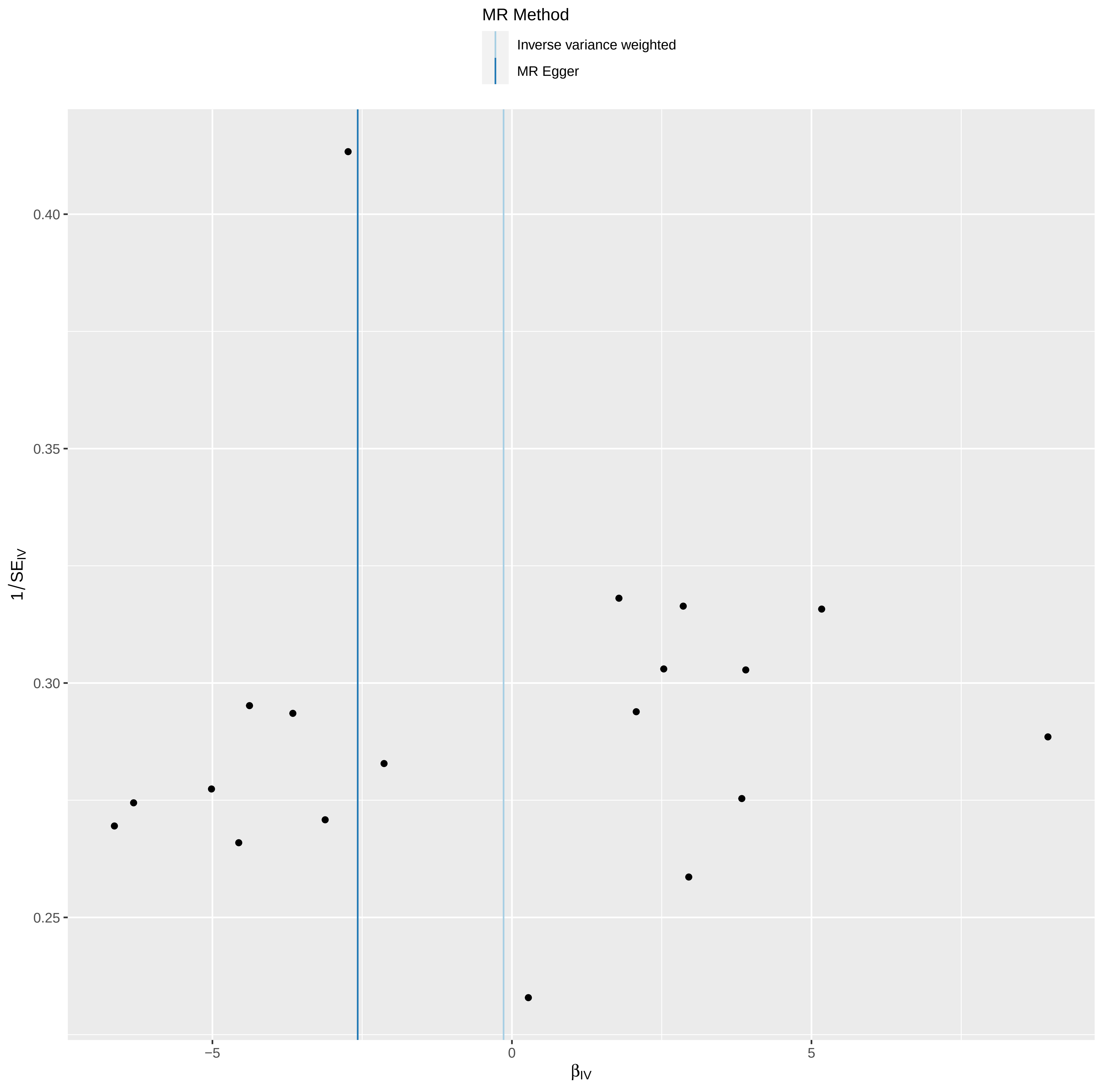
MR Method
Inverse variance weighted
MR Egger
0.40
0.35
0.30
0.25
1/SE_IV
−5
0
5
β_IV

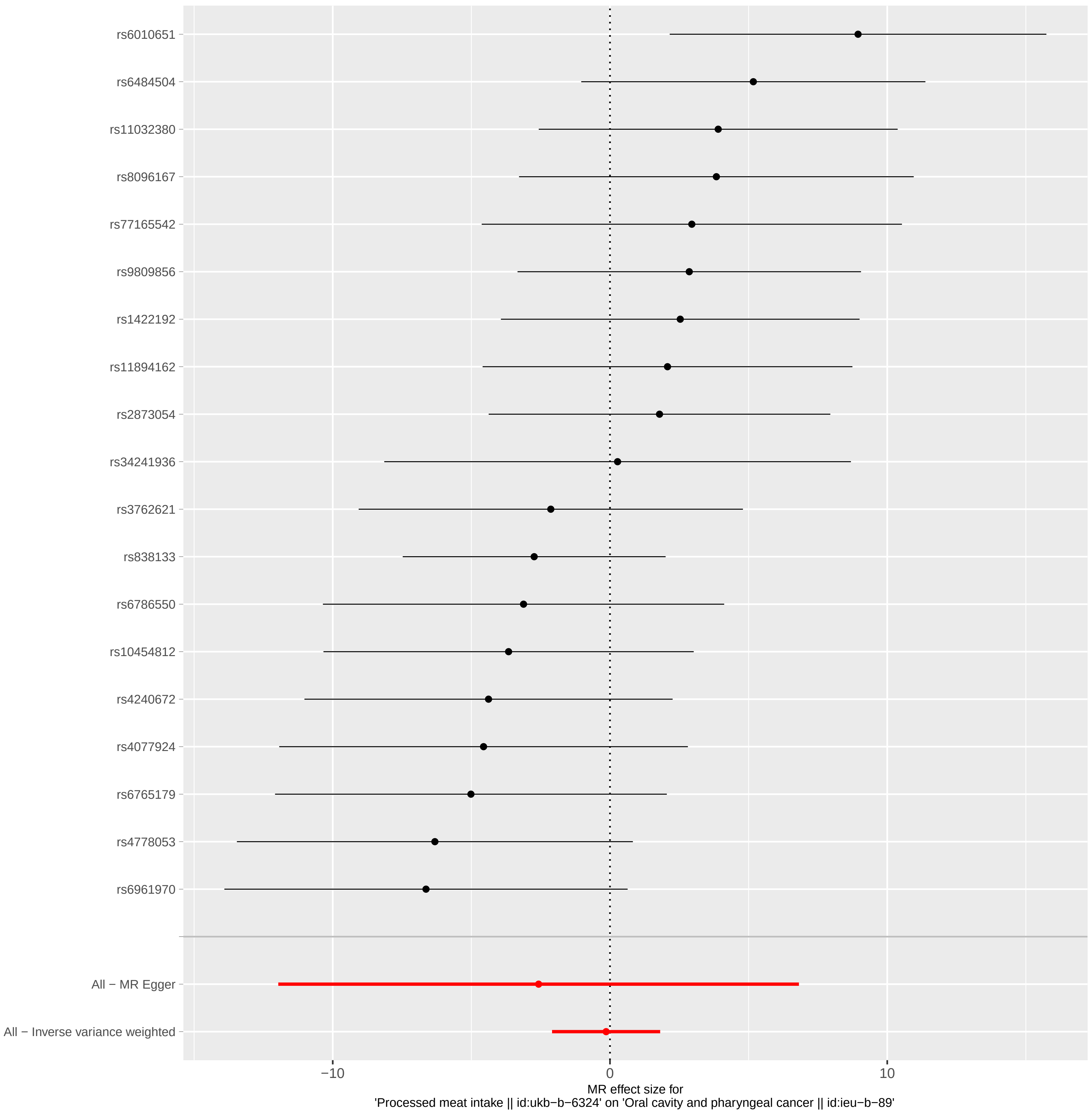
rs6010651
rs6484504
rs11032380
rs8096167
rs77165542
rs9809856
rs1422192
rs11894162
rs2873054
rs34241936
rs3762621
rs838133
rs6786550
rs10454812
rs4240672
rs4077924
rs6765179
rs4778053
rs6961970
All − MR Egger
All − Inverse variance weighted
−10
0
10
MR effect size for
'Processed meat intake || id:ukb−b−6324' on 'Oral cavity and pharyngeal cancer || id:ieu−b−89'

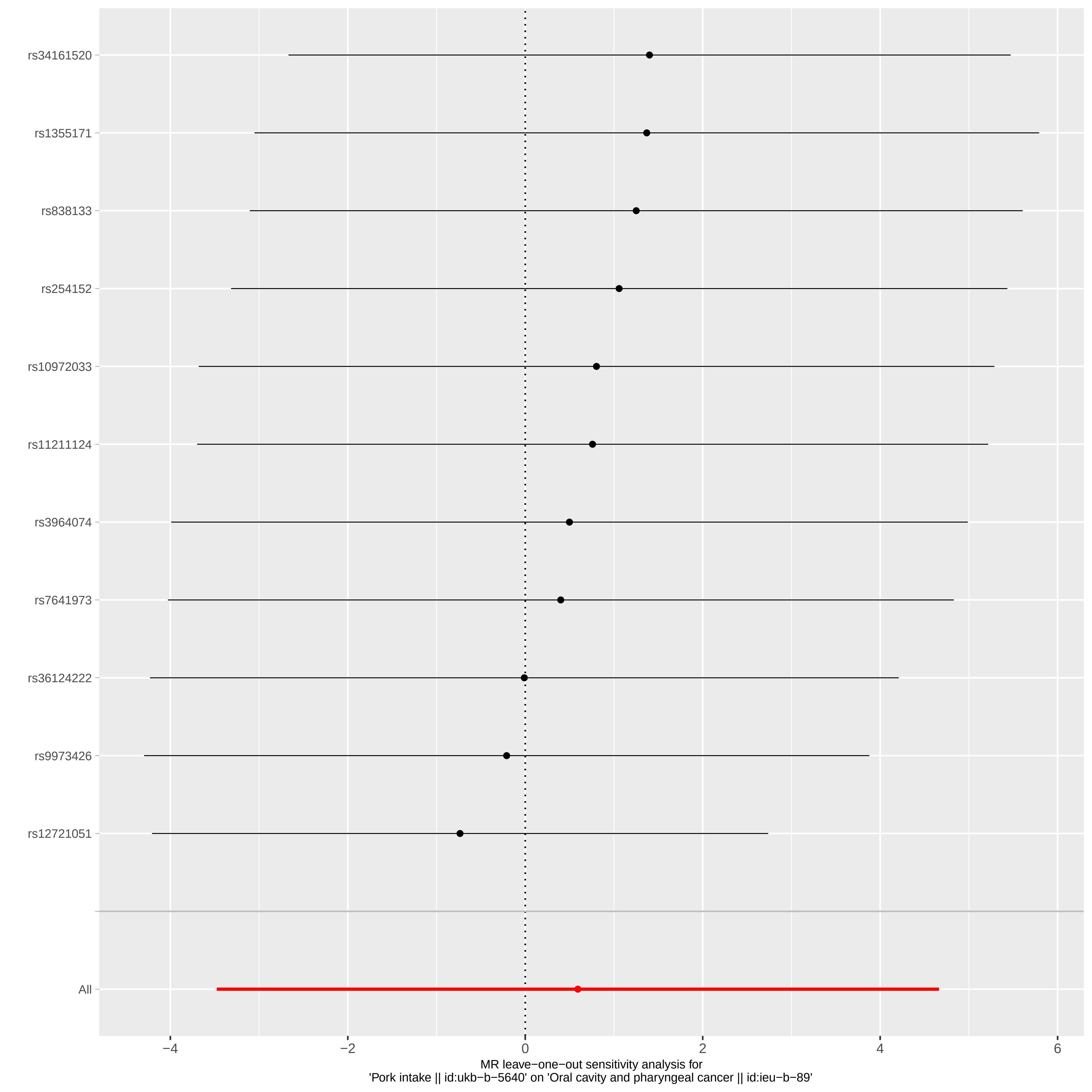
rs34161520
rs1355171
rs838133
rs254152
rs10972033
rs11211124
rs3964074
rs7641973
rs36124222
rs9973426
rs12721051
All
−4
−2
0
2
4
6
MR leave−one−out sensitivity analysis for
'Pork intake || id:ukb−b−5640' on 'Oral cavity and pharyngeal cancer || id:ieu−b−89'

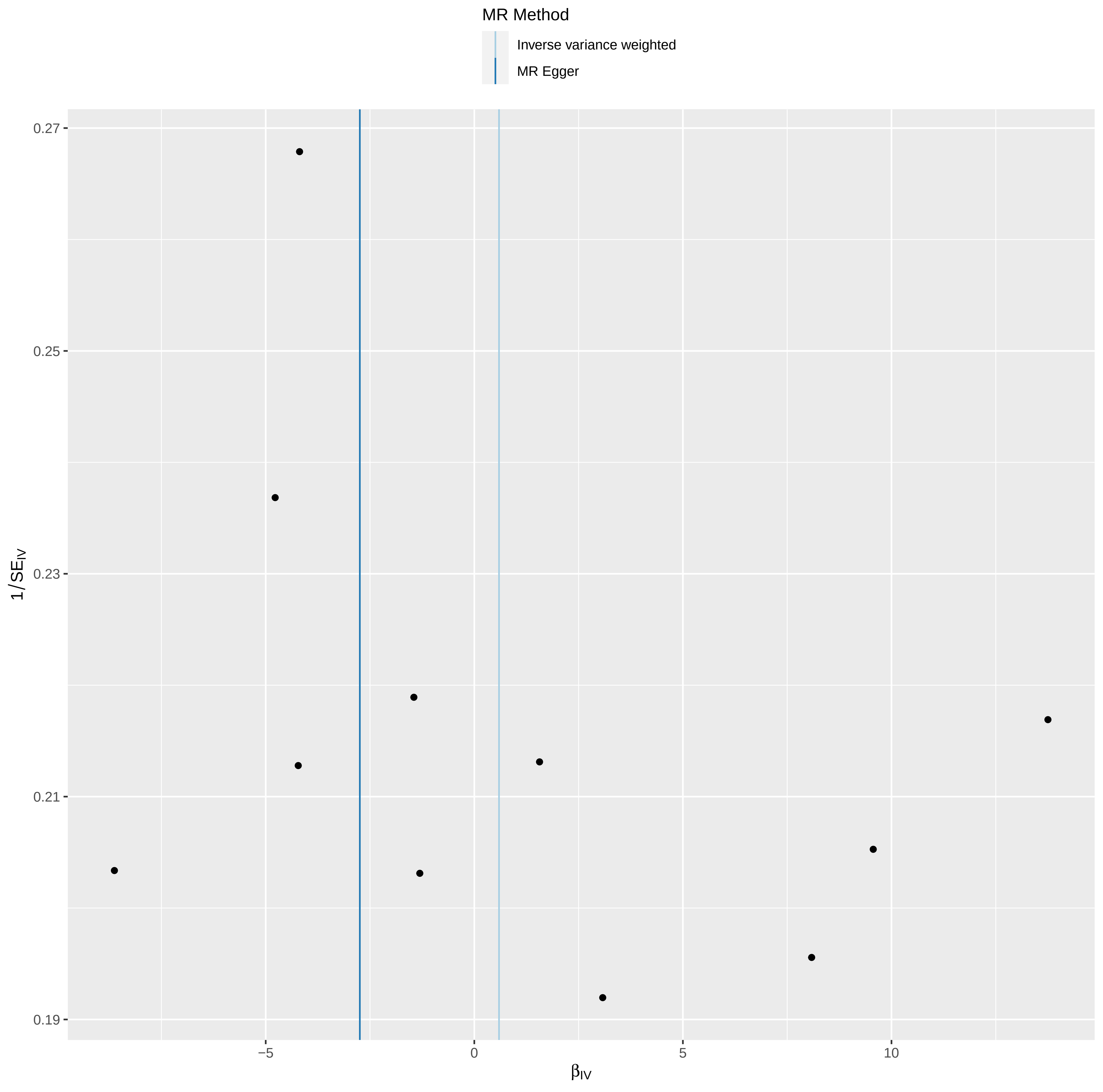
MR Method
Inverse variance weighted
MR Egger
0.27
0.25
0.23
0.21
0.19
$1/SE_{IV}$
−5
0
5
10
β_{IV}

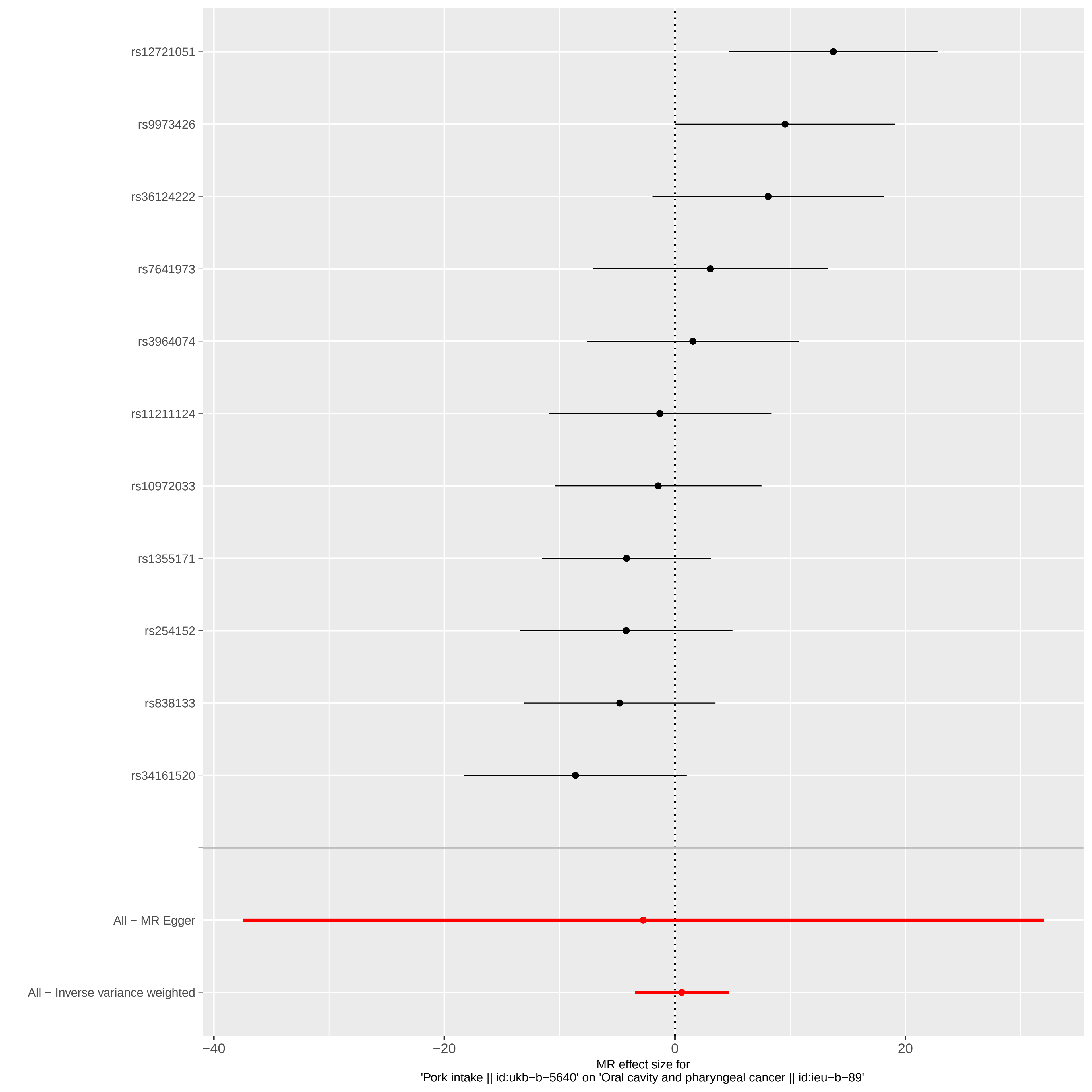
rs12721051
rs9973426
rs36124222
rs7641973
rs3964074
rs11211124
rs10972033
rs1355171
rs254152
rs838133
rs34161520
All − MR Egger
All − Inverse variance weighted
−40
−20
0
20
MR effect size for
'Pork intake || id:ukb−b−5640' on 'Oral cavity and pharyngeal cancer || id:ieu−b−89'

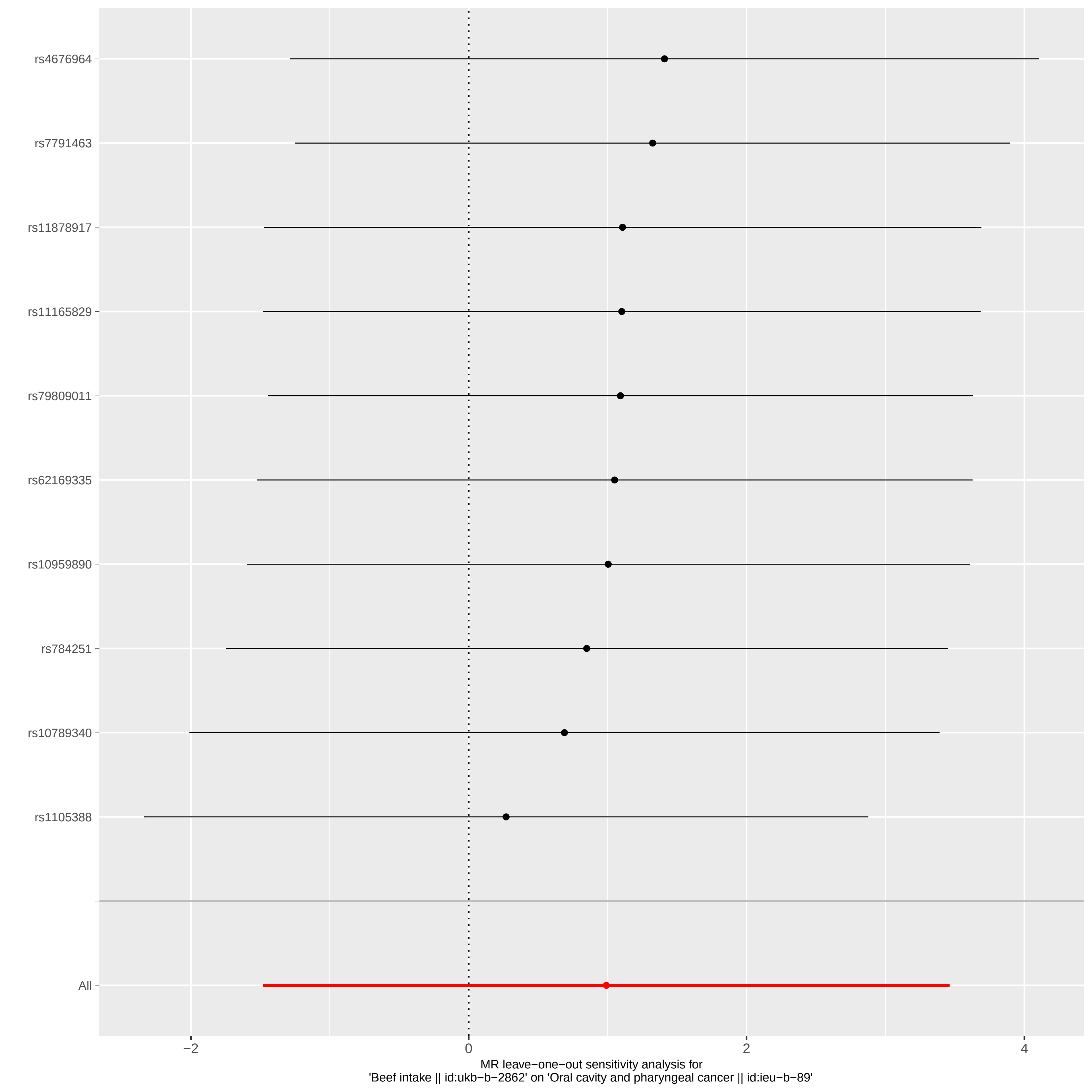
rs4676964
rs7791463
rs11878917
rs11165829
rs79809011
rs62169335
rs10959890
rs784251
rs10789340
rs1105388
All
−2
0
2
4
MR leave−one−out sensitivity analysis for
'Beef intake || id:ukb−b−2862' on 'Oral cavity and pharyngeal cancer || id:ieu−b−89'

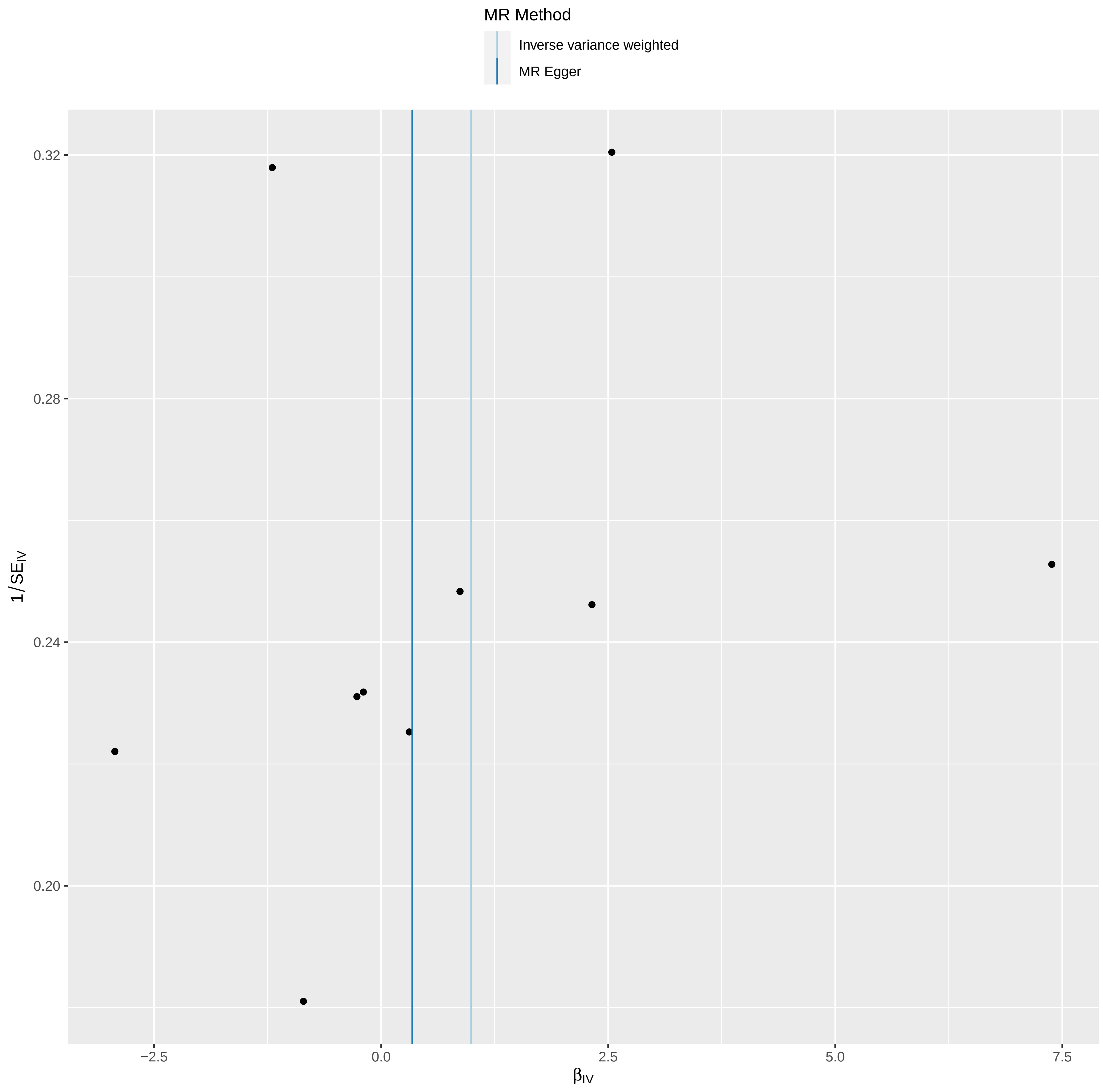
MR Method
Inverse variance weighted
MR Egger
0.32
0.28
0.24
0.20
1/SE$_{IV}$
−2.5
0.0
2.5
5.0
7.5
β$_{IV}$

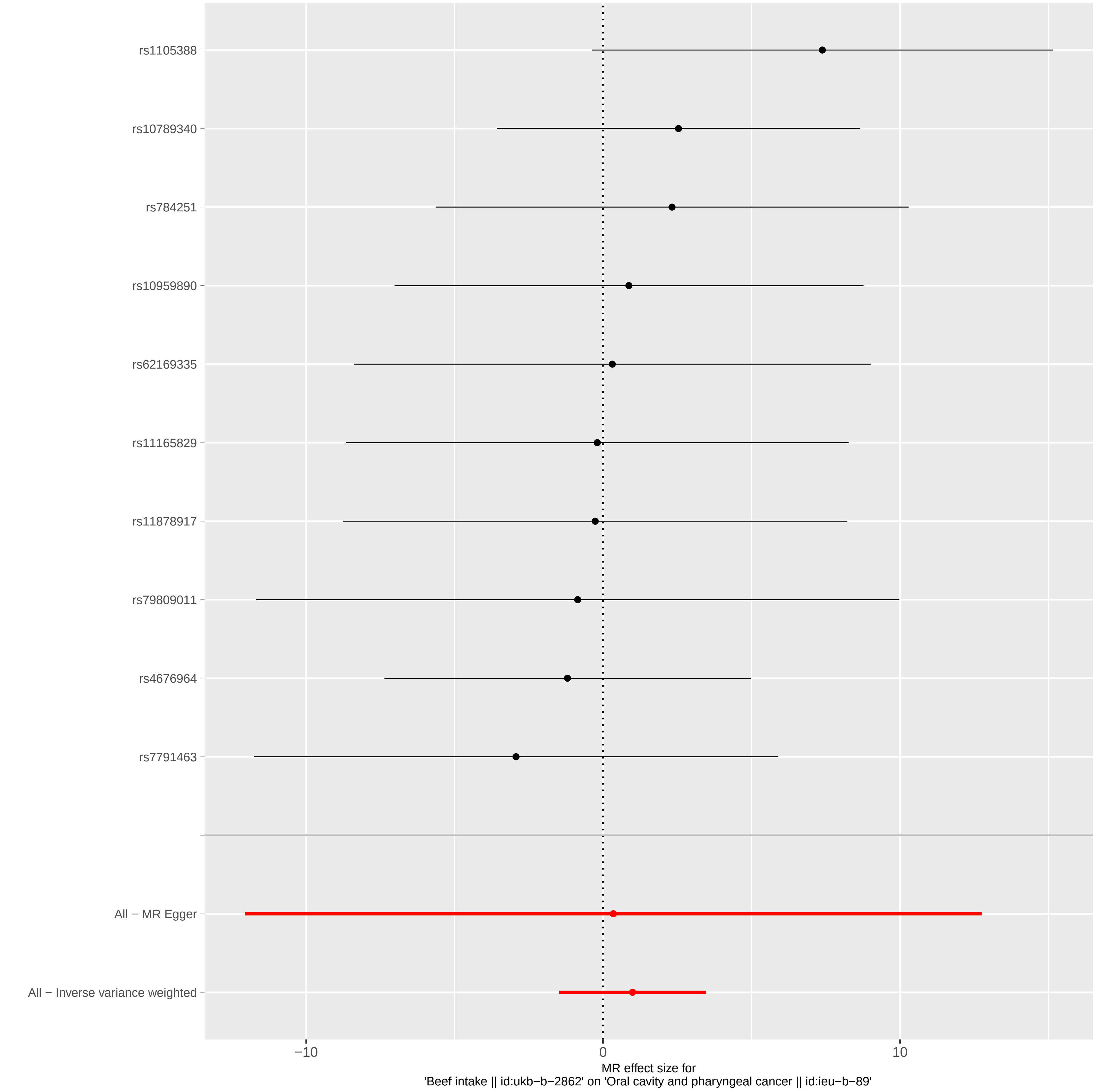

rs1105388
rs10789340
rs784251
rs10959890
rs62169335
rs11165829
rs11878917
rs79809011
rs4676964
rs7791463
All − MR Egger
All − Inverse variance weighted
−10
0
10
MR effect size for
'Beef intake || id:ukb−b−2862' on 'Oral cavity and pharyngeal cancer || id:ieu−b−89'

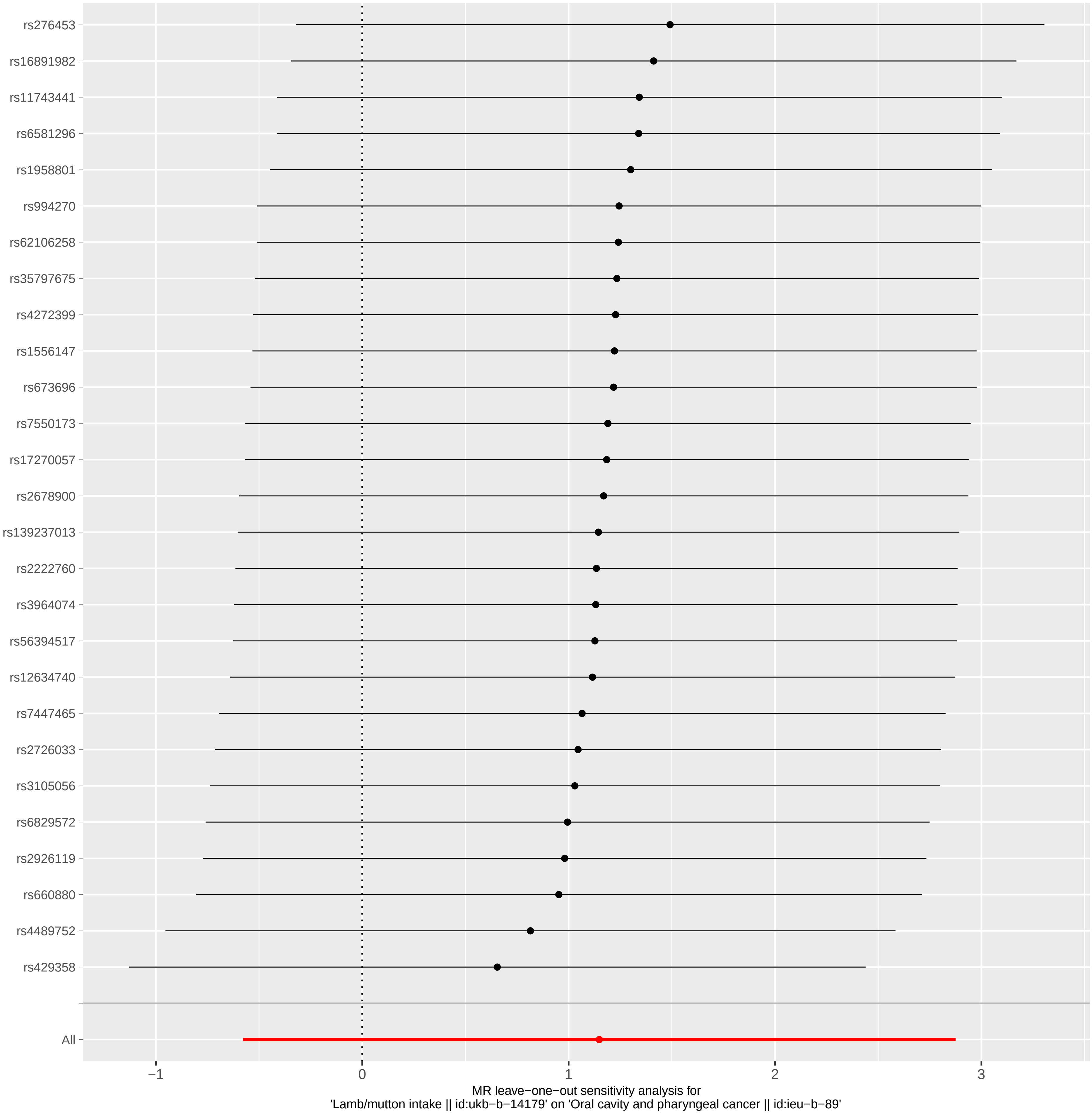
rs276453
rs16891982
rs11743441
rs6581296
rs1958801
rs994270
rs62106258
rs35797675
rs4272399
rs1556147
rs673696
rs7550173
rs17270057
rs2678900
rs139237013
rs2222760
rs3964074
rs56394517
rs12634740
rs7447465
rs2726033
rs3105056
rs6829572
rs2926119
rs660880
rs4489752
rs429358
All
−1
0
1
2
3
MR leave−one−out sensitivity analysis for
'Lamb/mutton intake || id:ukb−b−14179' on 'Oral cavity and pharyngeal cancer || id:ieu−b−89'

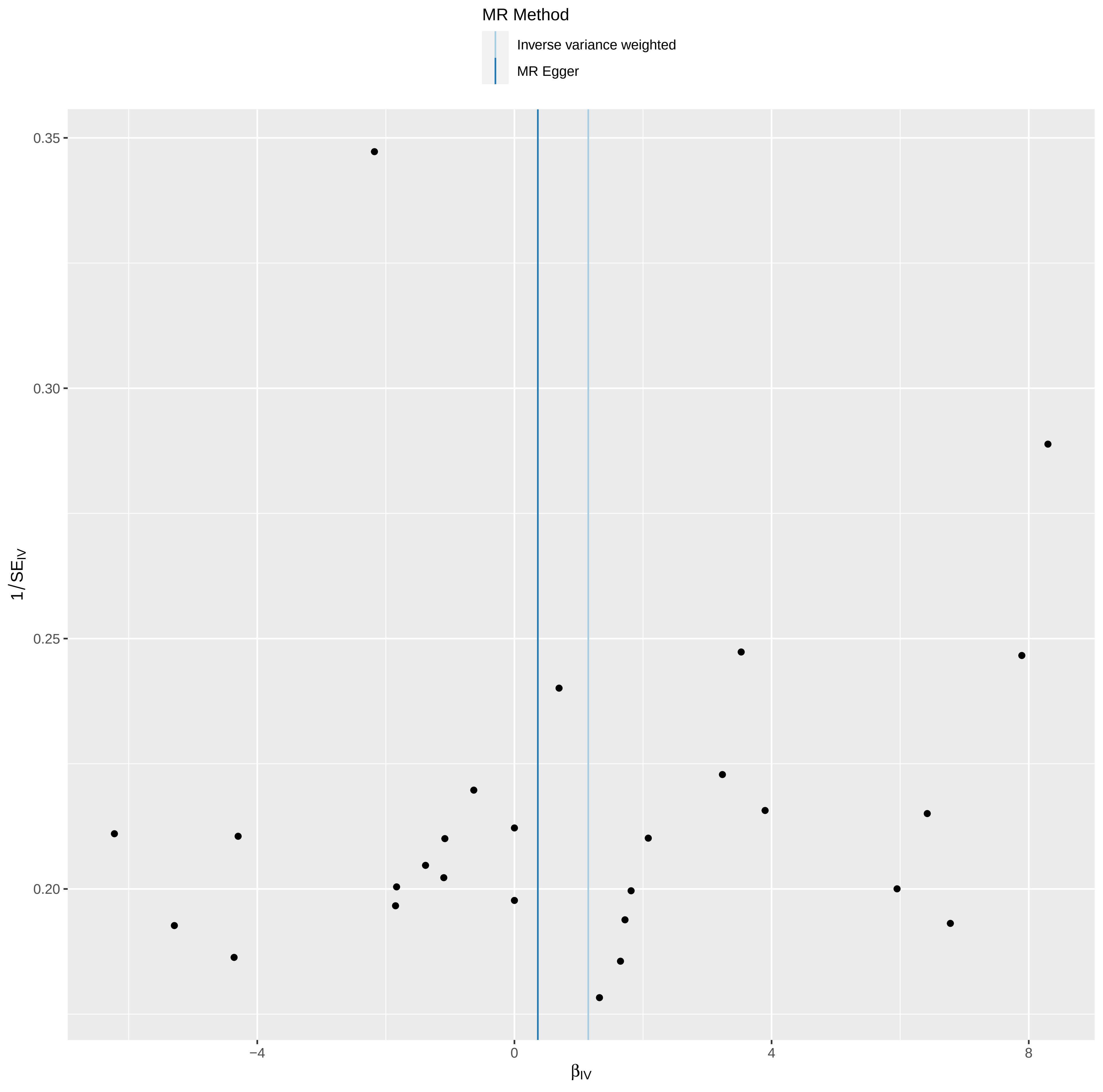
MR Method
Inverse variance weighted
MR Egger
0.35
0.30
0.25
0.20
1/SE$_{IV}$
−4
0
4
8
β$_{IV}$

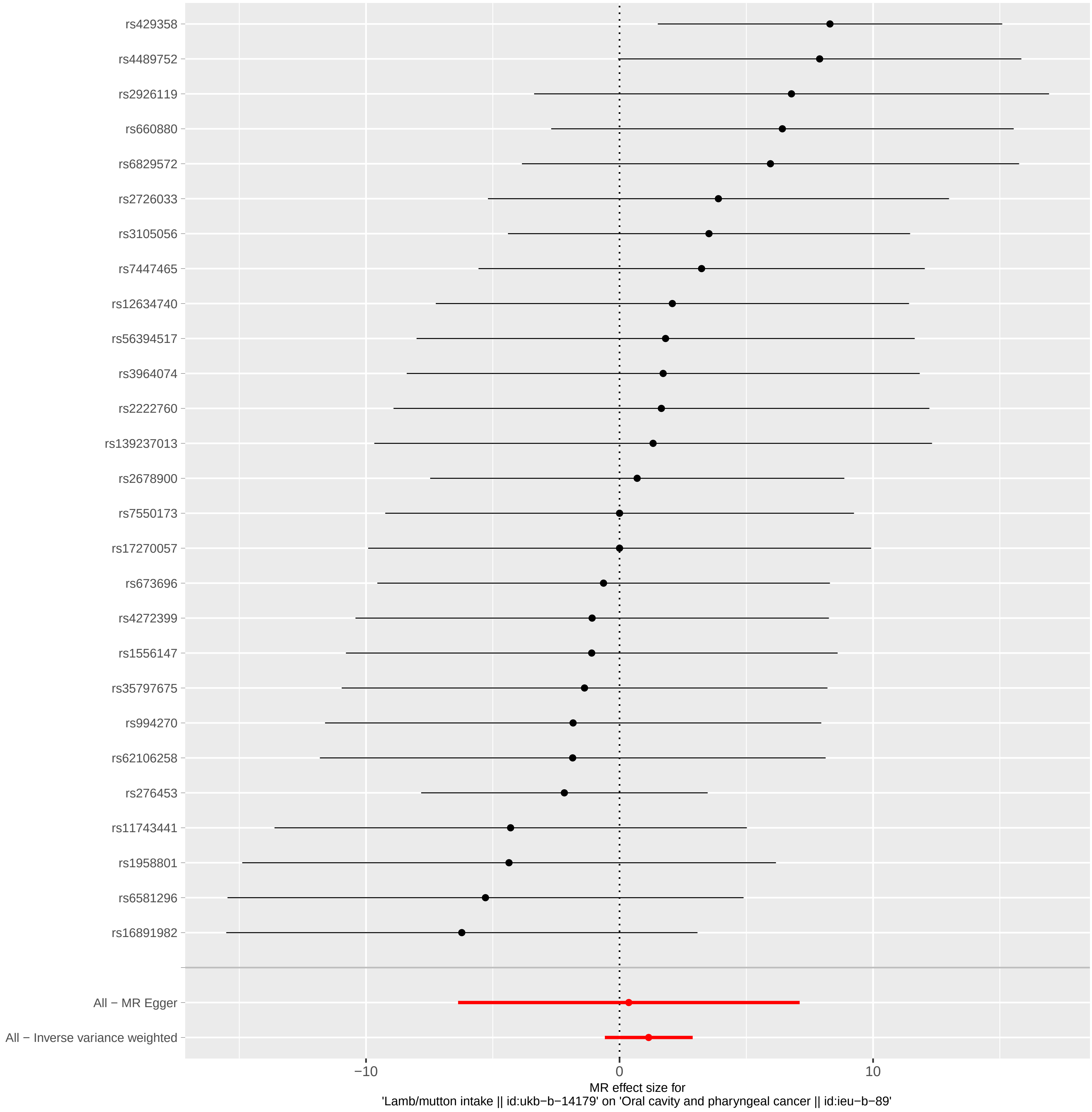
rs429358
rs4489752
rs2926119
rs660880
rs6829572
rs2726033
rs3105056
rs7447465
rs12634740
rs56394517
rs3964074
rs2222760
rs139237013
rs2678900
rs7550173
rs17270057
rs673696
rs4272399
rs1556147
rs35797675
rs994270
rs62106258
rs276453
rs11743441
rs1958801
rs6581296
rs16891982
All − MR Egger
All − Inverse variance weighted
−10
0
10
MR effect size for
'Lamb/mutton intake || id:ukb−b−14179' on 'Oral cavity and pharyngeal cancer || id:ieu−b−89'
